# The Burden of Motor Neuron Diseases in the United States, 1990–2021: A Systematic Analysis of the Global Burden of Disease Study 2021

**DOI:** 10.1002/mus.70023

**Published:** 2025-09-10

**Authors:** Yun‐Seo Oh, Raon Jung, Dong Keon Yon, Min‐Seo Kim, Joon‐Ho Shin, Jae Il Shin, Tae‐Jin Song

**Affiliations:** ^1^ Department of Medicine Ewha Womans University College of Medicine Seoul South Korea; ^2^ Center for Digital Health, Medical Science Research Institute, Kyung Hee University, Medical Center, Kyung Hee University, College of Medicine Seoul South Korea; ^3^ Medical and Population Genetics and Cardiovascular Disease Initiative Broad Institute of MIT and Harvard Cambridge Massachusetts USA; ^4^ Department of Rehabilitation Medicine National Rehabilitation Center, Ministry of Health and Welfare Seoul South Korea; ^5^ Department of Pediatrics Yonsei University College of Medicine Seoul South Korea; ^6^ Department of Neurology, Seoul Hospital Ewha Womans University College of Medicine Seoul South Korea; ^7^ Graduate Program in System Health Science and Engineering Ewha Womans University Seoul South Korea

**Keywords:** amyotrophic lateral sclerosis, disease burden, incidence, motor neuron diseases, prevalence

## Abstract

**Introduction/Aims:**

There is a lack of up‐to‐date information on the burden of motor neuron diseases (MNDs) in the United States (US). This study aimed to estimate trends in the prevalence, incidence, mortality, and disability‐adjusted life years (DALYs) for MNDs in the US from 1990 to 2021.

**Methods:**

We performed a secondary analysis of MNDs in the US using estimates of prevalence, incidence, and mortality obtained from analyses of the Global Burden of Disease 2021 dataset. These data were generated using DisMod‐MR 2.1, a Bayesian meta‐regression tool. Estimates were analyzed by age group, sex, region, and sociodemographic index (SDI).

**Results:**

In 2021, the age‐standardized prevalence rate of MNDs in the US was 8.82 (95% uncertainty interval, 7.96–9.74) per 100,000, a 12.89% (3.10–23.66) increase from 1990 (7.82 per 100,000). Age‐standardized MND‐related DALY and mortality rates in 2021 were 41.36 (39.47–42.94) and 1.49 (1.38–1.56) per 100,000, respectively, increases of 4.14% (0.41%–7.68%) and 18.34% (13.86%–22.70%) compared to 1990. Geographic disparities were observed, with the West North Central reporting the highest DALY rates and the Middle Atlantic showing the lowest. The burden of MNDs was consistently greater in males across all metrics, with a male‐to‐female ratio of approximately 1.4:1. SDI was negatively correlated with age‐standardized DALYs, years of life lost, and mortality rates.

**Discussion:**

The observed burden of MNDs in the US highlights the necessity for targeted public health interventions; equitable resource distribution; and further research into environmental, genetic, and sociodemographic factors that contribute to MNDs.

## Graphical abstract

This analysis of Global Burden of Disease data reveals a rising burden of motor neuron diseases in the United States from 1990 to 2021, with increasing prevalence and mortality. Geographic and sociodemographic disparities highlight the need for public health interventions and equitable resource distribution.

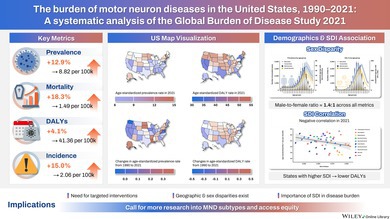

**Abbreviations**
ALS: amyotrophic lateral sclerosis
CODEm: Cause of Death Ensemble modeling
CSMR: cause‐specific mortality rate
DALYs: disability‐adjusted life years
GBD: Global Burden of Disease
GHDx: Global Health Data Exchange
ICD: International Classification of Diseases
IHME: Institute for Health Metrics and Evaluation
MNDs: motor neuron diseases
SDI: sociodemographic index
UIs: uncertainty intervals
US: United States
YLDs: years lived with disability
YLLs: years of life lost

## 1 Introduction

The global prevalence of motor neuron diseases (MNDs) and associated deaths was higher in 2019 than in 1990, without a significant change in incidence [1]. Population aging is considered the main driver of this trend, but it does not fully explain the increase [2, 3]. Furthermore, the burden of MNDs tends to be higher in more developed countries, as evidenced by the robust positive correlation between the sociodemographic index (SDI) and both the age‐standardized prevalence and disability‐adjusted life year (DALY) rates of MNDs [4]. In the United States (US), DALY rates of the MNDs increased by 20.9% between 1990 and 2017 [5].

Global Burden of Disease (GBD) studies primarily examine the burden of diseases, injuries, and risk factors, providing global, regional, and country‐specific estimates [1, 5, 6]. However, little research has focused specifically on MNDs in the US [7]. Accurate data on the incidence, prevalence, mortality, and disability caused by MNDs and their trends are crucial for evidence‐based healthcare planning and resource allocation. Therefore, national and state‐level epidemiological data in the US need to be gathered and synthesized for healthcare planners. This study aimed to present trends in the MND burden in the US from 1990 to 2021 by geographic unit, sex, age group, and SDI, based on data from the GBD 2021 study.

## 2 Methods

### 2.1 Overview and Data Source

The GBD study is a comprehensive analysis of global diseases. Using estimates from the GBD study, current global, regional, and national disease burdens can be compared and assessed [8]. The GBD study adheres to the Guidelines for Accurate and Transparent Health Estimates Reporting [9] and is operated by the Institute for Health Metrics and Evaluation (IHME) at the University of Washington using anonymized data, for which informed consent is not required. The data analyzed in this study were sourced from GBD 2021, which offers the most recent epidemiological estimates for 371 diseases and injuries across 21 GBD regions and 204 countries and territories from 1990 to 2021. All data are freely available through the Global Health Data Exchange (GHDx) (https://ghdx.healthdata.org.) [10], and comprehensive information on data collection, methodologies, and statistical modeling are available in previous reports [11, 12]. As GBD collaborators, we performed a secondary analysis regarding the GBD 2021 estimates for MNDs to investigate the burden in the US. These estimates were part of an open‐access data resource available through the GHDx upon approval of a study proposal submitted to the IHME. Our institutional review board reviewed and approved a waiver of informed consent for this study (EUMC 2023‐10‐040). This manuscript was produced as part of the GBD Collaborator Network in accordance with the GBD Protocol.

### 2.2 Case Definition

MNDs are defined as a set of chronic, degenerative, and progressive neurological conditions typified by destruction of upper and lower motor neurons and the subsequent deterioration of voluntary muscle activity. The IHME investigated estimates of disease burden by conducting a systematic review and collecting claims data using the International Classification of Disease (ICD) codes, specifically ICD‐9 codes ranging from 335 to 335.9, and ICD‐10 codes ranging from G12 to G12.9. However, the GBD dataset reports only aggregated estimates for overall MNDs, without detailed information on specific subcodes, such as amyotrophic lateral sclerosis (ALS), spinal muscular atrophy, post‐polio syndrome, and non‐ALS MNDs. That is, the estimates represent the combined disease burden of all these subtypes under a single category of MNDs.

### 2.3 Input Data and Estimates

For the GBD study, a systematic review of published studies and claims data were used as input data. A detailed list of data sources is available in GHDx (https://ghdx.healthdata.org/gbd‐2021/sources). A flowchart illustrating the steps used to derive the final burden estimation and the search terms used to gather the systematic review is provided in the Supporting Information S1. Methods for gathering data on non‐fatal outcomes and mortality have been detailed in previous studies [10, 12].

To investigate the burden of MNDs, their prevalence, incidence, DALYs, years of life lost (YLLs), years lived with disability (YLDs), and mortality were estimated. DALYs are a measure of the loss of health in a population. One DALY represents the loss of 1 year of full health and was calculated as the sum of the YLLs and YLDs. We obtained MND‐related estimates at the global, regional, and national levels, including all 50 states and the District of Columbia. Estimates were stratified by sex and age groups. For the geographic analysis within the US, divisions and regions were defined according to the classifications provided by the US Census Bureau [13]. Figures and tables were constructed by sex, age, and SDI for clear interpretation of observed trends.

### 2.4 Mortality and Severity

Mortality from MNDs was estimated using data from the Cause of Death database, including vital registration and surveillance data. Sources for this database include national mortality systems such as the United States National Vital Statistics System. The standard Cause of Death Ensemble modeling (CODEm) [11, 12] approach, commonly used in GBD study, was used to estimate deaths from MNDs. CODEm uses an ensemble of statistical models while systematically testing combinations of covariates based on their out‐of‐sample predictive validity. Separate models were conducted for male and female mortality, and the age for both models ranged from 0 days to 95+ years. Unadjusted death estimates were adjusted using CoDCorrect to produce the final YLLs estimates. Body mass index, serum total cholesterol, latitude, fasting plasma glucose, fruit consumption, and SDI score were selected as covariates in the model. The covariates used in MND mortality modeling are accessible online at GHDx (https://www.healthdata.org/gbd/methods‐appendices‐2021/motor‐neuron‐disease). With regard to the severity of MNDs, since ALS accounts for the majority of MND cases [14], the methodology used to assess the severity of ALS from the GBD dataset is described in the Supporting Information S1.

### 2.5 SDI

The SDI, introduced by the IHME in 2015, is a comprehensive metric used to assess the development level of countries or regions to investigate relationships between social development and population health outcomes. It was calculated as the geometric mean of three normalized indicators: the total fertility rate for individuals younger than 25 years, the mean education level for individuals aged 15 years and older, and the lag‐distributed income per capita. For the GBD 2021 study, SDI values ranged from 0 to 1, with 0 representing the lowest education and income levels and the highest fertility rate and 1 representing the highest education and income levels and the lowest fertility rate. Detailed information regarding SDI values is available online at GHDx (https://ghdx.healthdata.org/record/global‐burden‐disease‐study‐2021‐gbd‐2021‐socio‐demographic‐index‐sdi‐1950%E2%80%932021). The 204 countries and territories were divided into five SDI quintiles: low (< 0.46), low‐middle (0.46–0.60), middle (0.61–0.69), high‐middle (0.70–0.81), and high (> 0.81) [11, 12].

### 2.6 Statistical Analysis

In the GBD 2021 study, DisMod‐MR 2.1, a Bayesian meta‐regression tool designed for disease modeling, was used as the main analytical tool for MND estimation. Input data included prevalence, incidence, cause‐specific mortality rates (CSMR) from the GBD causes of death analysis, and excess mortality rate, calculated by dividing the CSMR by prevalence. Pre‐specified modeling assumptions included zero remission across all ages and a maximum incidence rate of 0.0004. The prevalence and incidence of super‐region random effects were constrained to −0.5 and 0.5, respectively, to account for spurious inflation of regional differences. Further details of the modeling strategy are provided in the Supplementary Methods. Results are presented as total counts and all‐ages or age‐standardized rates per 100,000 population with 95% uncertainty intervals (UIs). Spearman's correlation and linear regression analyses were conducted to assess the relationship between SDI and the burden of MNDs, using R version 4.4.2 (https://www.r‐project.org/).

## 3 Results

### 3.1 Changes in the Burden of Motor Neuron Diseases

In 2021, the age‐standardized DALY rate for MNDs was 41.36 per 100,000, representing a 4.14% increase from 1990. The age‐standardized YLD rate increased by 12.80% and the age‐standardized YLL rate by a not significant amount of 3.76% during the same period (Table S1). The relative contributions of YLDs and YLLs to DALYs are presented in Figure S1. The age‐standardized prevalence rate for MNDs in 2021 was 8.82 per 100,000, reflecting a 12.89% increase from 1990. The age‐standardized MND mortality rate in 2021 was 1.49 per 100,000, an increase of 18.34% from 1990. Furthermore, the age‐standardized incidence rate in 2021 was 2.06 per 100,000, an increase of 15.03% from 1990 (Table 1 and Table S1).

**TABLE 1:** Numbers, age‐standardized rates, percentage changes of prevalence, DALYs, and deaths in the US.

	Prevalence (95% UI)			DALYs (95% UI)			Deaths (95% UI)		
	Absolute number, thousands, 2021	Age‐standardized rate, per 100,000 people, 2021	Percentage change, 1990–2021	Absolute number, thousands, 2021	Age‐standardized rate, per 100,000 people, 2021	Percentage change, 1990–2021	Absolute number, thousands, 2021	Age‐standardized rate, per 100,000 people, 2021	Percentage change, 1990–2021
United States of America	40495.43 (37645.36–43576.27)	8.82 (8.26–9.51)	12.89% (3.10%–23.66%)	210559.45 (199250.06–218996.64)	41.36 (39.47–42.94)	4.14% (0.41%–7.68%)	8465.56 (7799.92–8898.99)	1.49 (1.38–1.56)	18.34% (13.86%–22.70%)
Northeast	8183.64 (7585.57–8850.32)	10.18 (9.34–11.14)	17.66% (6.48%–30.05%)	35531.92 (30086.29–41220.94)	40.24 (34.42–46.48)	−1.67% (−16.04% to 13.94%)	1448.54 (1210.87–1687.98)	1.46 (1.24–1.71)	9.55% (−7.31% to 28.16%)
Midwest	8739.43 (8027.03–9522.62)	9.25 (8.42–10.16)	15.17% (4.99%–27.17%)	49542.83 (42262.27–57480.71)	46.88 (40.36–53.89)	13.48% (−2.52% to 30.87%)	2015.52 (1706.17–2350.38)	1.69 (1.44–1.97)	25.72% (6.89%–46.42%)
South	14388.31 (13313.62–15571.30)	8.37 (7.69–9.13)	20.43% (8.80%–34.01%)	78767.27 (67435.83–90938.09)	44.11 (38.10–50.68)	14.67% (−1.52% to 32.67%)	3127.04 (2649.51–3635.53)	1.55 (1.32–1.79)	28.56% (9.47%–49.93%)
West	9184.04 (8481.42–10011.31)	9.12 (8.27–10.11)	12.89% (2.35%–24.99%)	46717.43 (40086.34–53761.52)	43.41 (37.51–49.86)	5.10% (−9.34% to 21.39%)	1874.45 (1593.66–2175.82)	1.56 (1.32–1.81)	17.75% (0.45%–37.41%)

Abbreviations: DALYs, disability‐adjusted life years; UI, uncertainty interval; US, United States.

**FIGURE 1:**
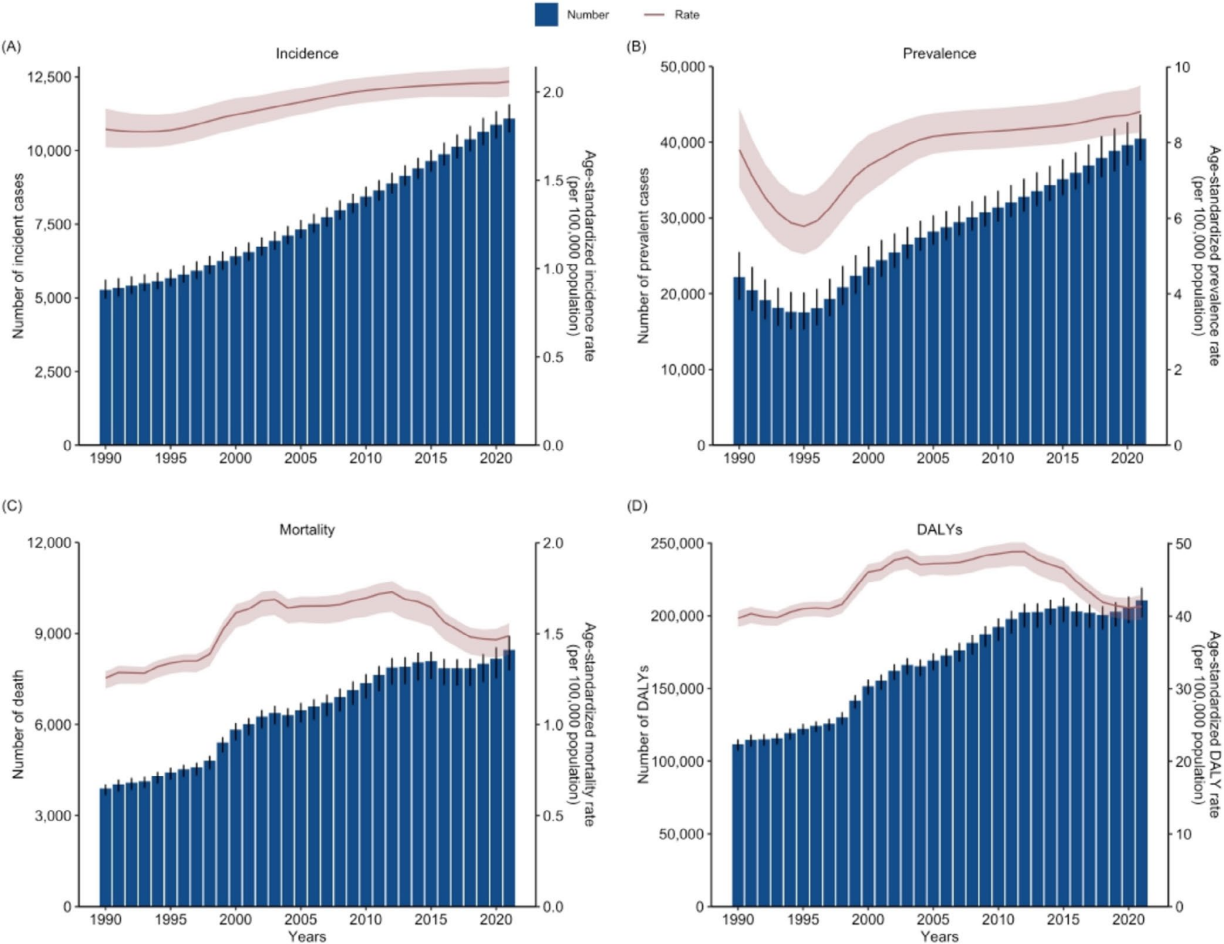
Annual trends in the numbers and age‐standardized rates of motor neuron disease in the US from 1990 to 2021 for (A) incidence, (B) prevalence, (C) deaths, and (D) DALYs. DALYs, disability‐adjusted life years; US, United States.

**FIGURE 2:**
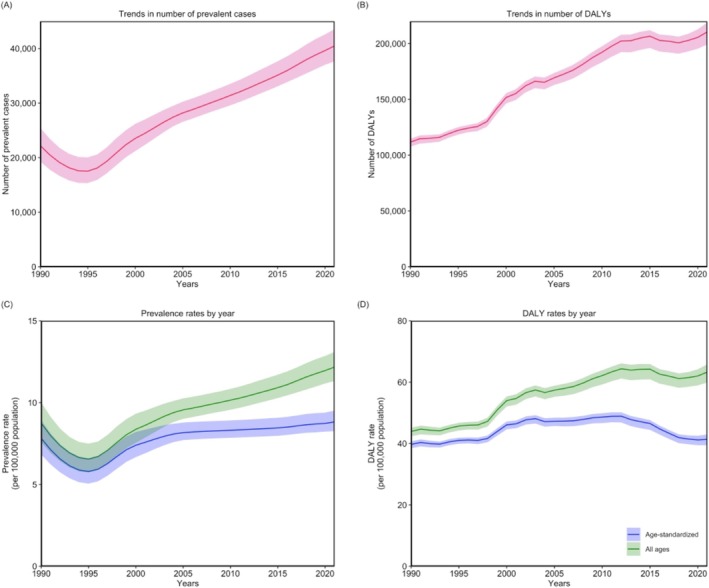
Annual trends in (A) the number of prevalent cases, (B) the number of DALYs, (C) prevalence rate and age‐standardized prevalence rate, and (D) DALY rate and age‐standardized DALY rate in the US from 1990 to 2021. DALYs, disability‐adjusted life years; US, United States.

From 1990 to 2003, both males and females experienced increasing trends in age‐standardized death, DALY, and YLL rates for MNDs. After 2003, those rates showed a slight decrease, followed by an increase until 2012, with a notable decrease thereafter. In contrast, age‐standardized YLD and prevalence rates decreased steadily until 1995 and began to increase afterward. Those rates rose rapidly until 2005, after which their growth became more gradual. The age‐standardized incidence rate, however, showed a slight decline until 1993, followed by a sharp increase (Table S2 and Figures 1 and 2).

### 3.2 Comparison of US, Europe, Asia, and Global Data

Among Asia, Europe, US, and global data, the US exhibited the highest age‐standardized rates of DALYs, prevalence, mortality, and incidence for MNDs in both 1990 and 2021, followed by Europe, global, and Asia. Between 1990 and 2021, the US showed the second‐largest increase in prevalence and incidence rates, following Europe. Meanwhile, the increases in DALY and mortality rates ranked third and fourth, respectively, among the US, Europe, Asia, and global (Table S1).

### 3.3 Geographic Variation in Motor Neuron Diseases

**FIGURE 3:**
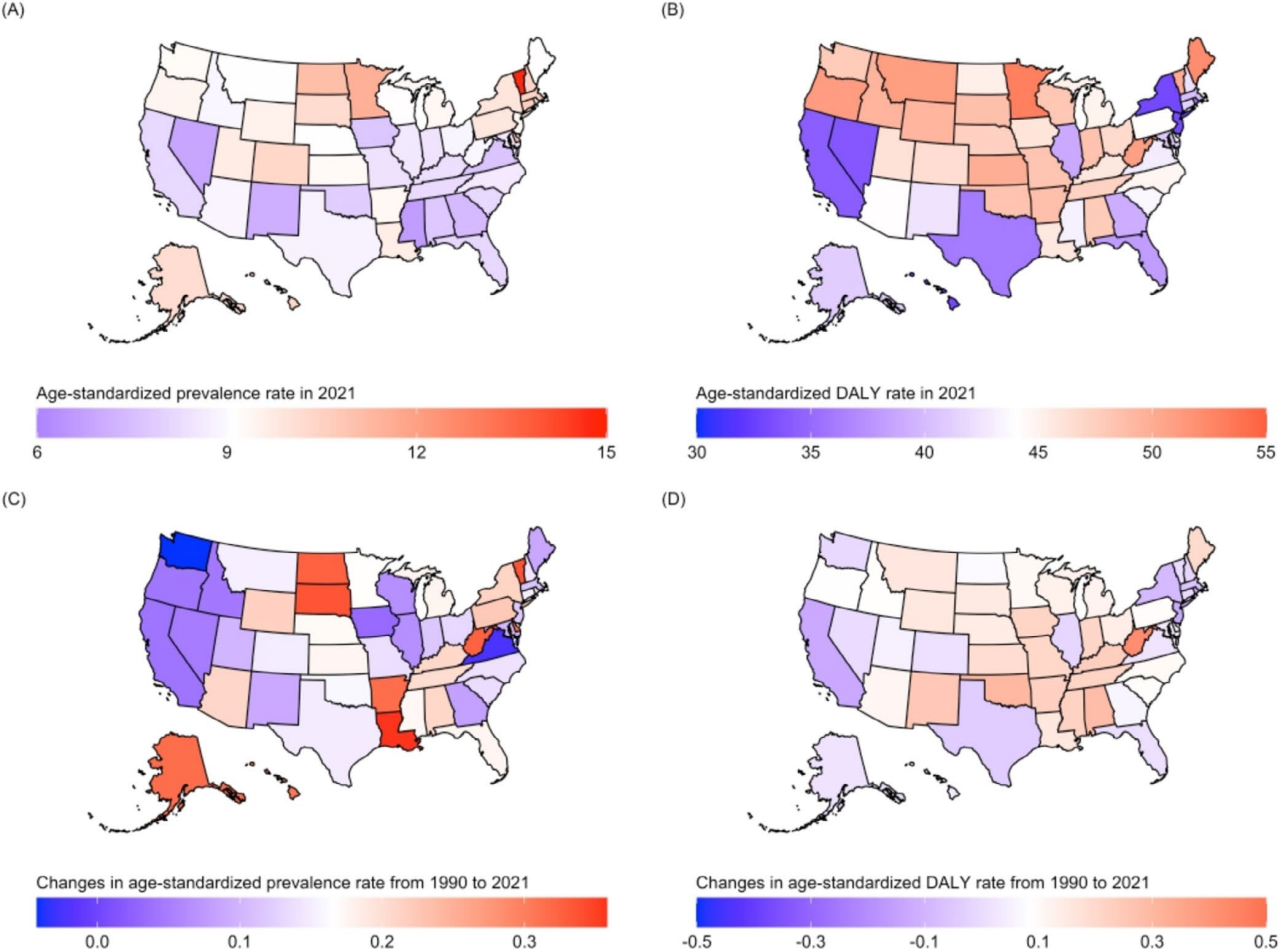
Age‐standardized rates of (A) prevalence and (B) DALYs in 2021, and percentage changes in age‐standardized (C) prevalence and (D) DALY rates from 1990 to 2021 across US states. DALYs, disability‐adjusted life years; US, United States.

The burden of MNDs exhibited substantial geographic variation across the US. Age‐standardized rates of MND prevalence, DALYs, and deaths for the 50 states and the District of Columbia in 2021 are presented in Table S3 and Figure 3. Minnesota, Maine, and Oregon recorded the highest age‐standardized DALY rates. In contrast, the District of Columbia, Hawaii, and New York exhibited the lowest DALY rates (Figure S2). West Virginia, Oklahoma, Alabama, and New Mexico showed the largest increases in age‐standardized DALY rates from 1990 to 2021, with each state reporting more than a 25% growth. Conversely, the District of Columbia, California, and New Jersey showed a decrease in the DALY rate (Figure S3). Decadal trends in age‐standardized DALY rates from 2000 to 2010 and from 2010 to 2021 are illustrated in Figure S4. Further state‐level changes during the pandemic period (2019–2021) are presented in Figure S5.

**FIGURE 4:**
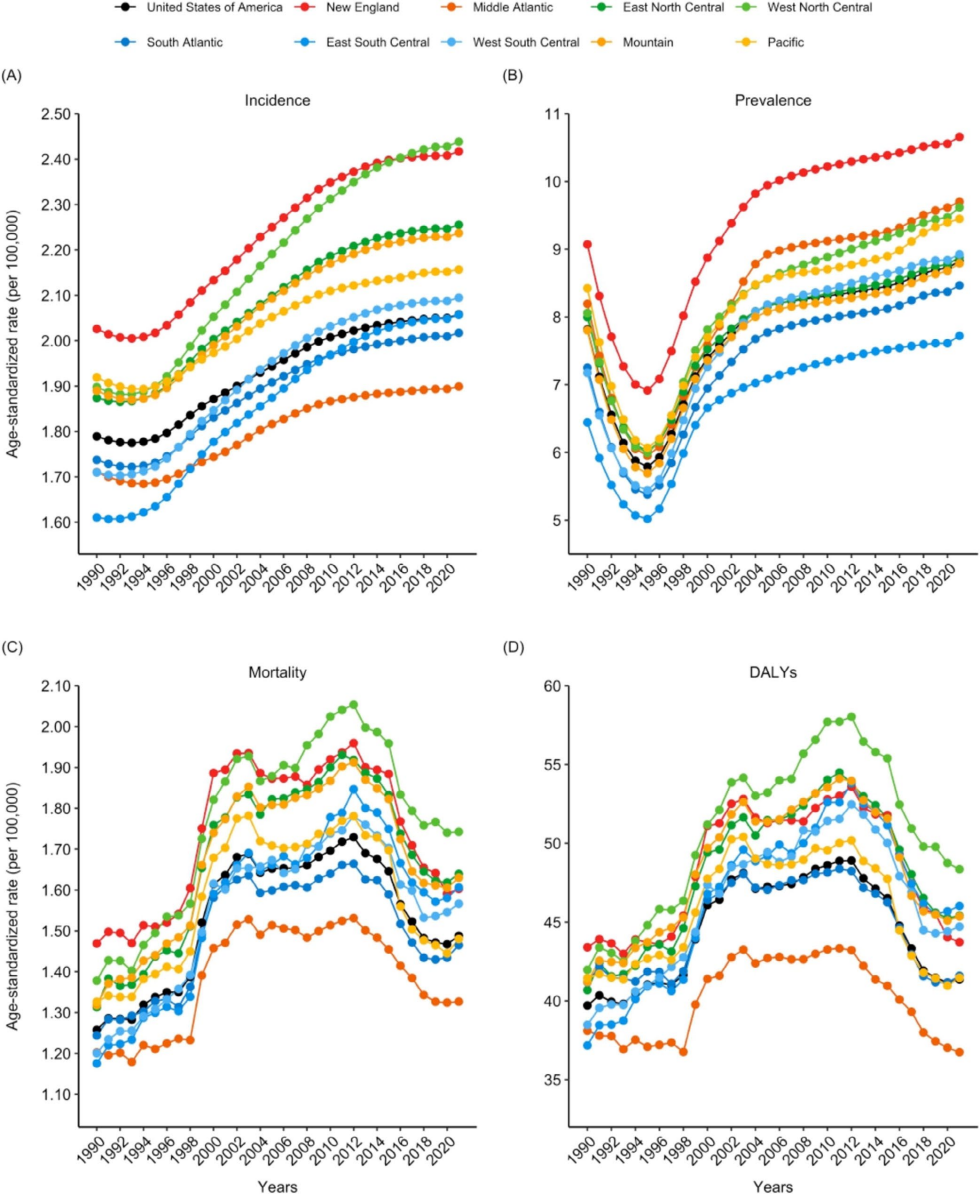
Annual trends in age‐standardized (A) incidence, (B) prevalence, (C) mortality, and (D) DALY rates of motor neuron disease from 1990 to 2021 across nine Census divisions of the US. DALY, disability‐adjusted life year; US, United States.

An examination of trends across the four regions of the US (Northeast, Midwest, South, and West) showed that the Midwest had the highest age‐standardized DALY rate in 2021, followed by the South, West, and Northeast (Table 1). Age‐standardized rates of incidence, prevalence, mortality, and DALYs from 1990 to 2021 across the nine US Census divisions (New England, Middle Atlantic, East North Central, West North Central, South Atlantic, East South Central, West South Central, Mountain, and Pacific) are presented in Figure 4. All nine Census divisions showed increasing incidence and prevalence rates since 1995, whereas DALY and mortality rates exhibited a biphasic pattern, with local peaks around 2003 and 2012. In 2021, the West North Central and Middle Atlantic divisions both exhibited relatively high prevalence rates, ranking 3rd and 2nd, but showed the highest and lowest rates of incidence, DALYs, and mortality, respectively. Changes in age‐standardized rates during the pre‐pandemic (1990–2019) and pandemic periods (2020–2021) for the divisions are illustrated in Figure S6.

### 3.4 Sex and Age Differences in Motor Neuron Diseases

**FIGURE 5:**
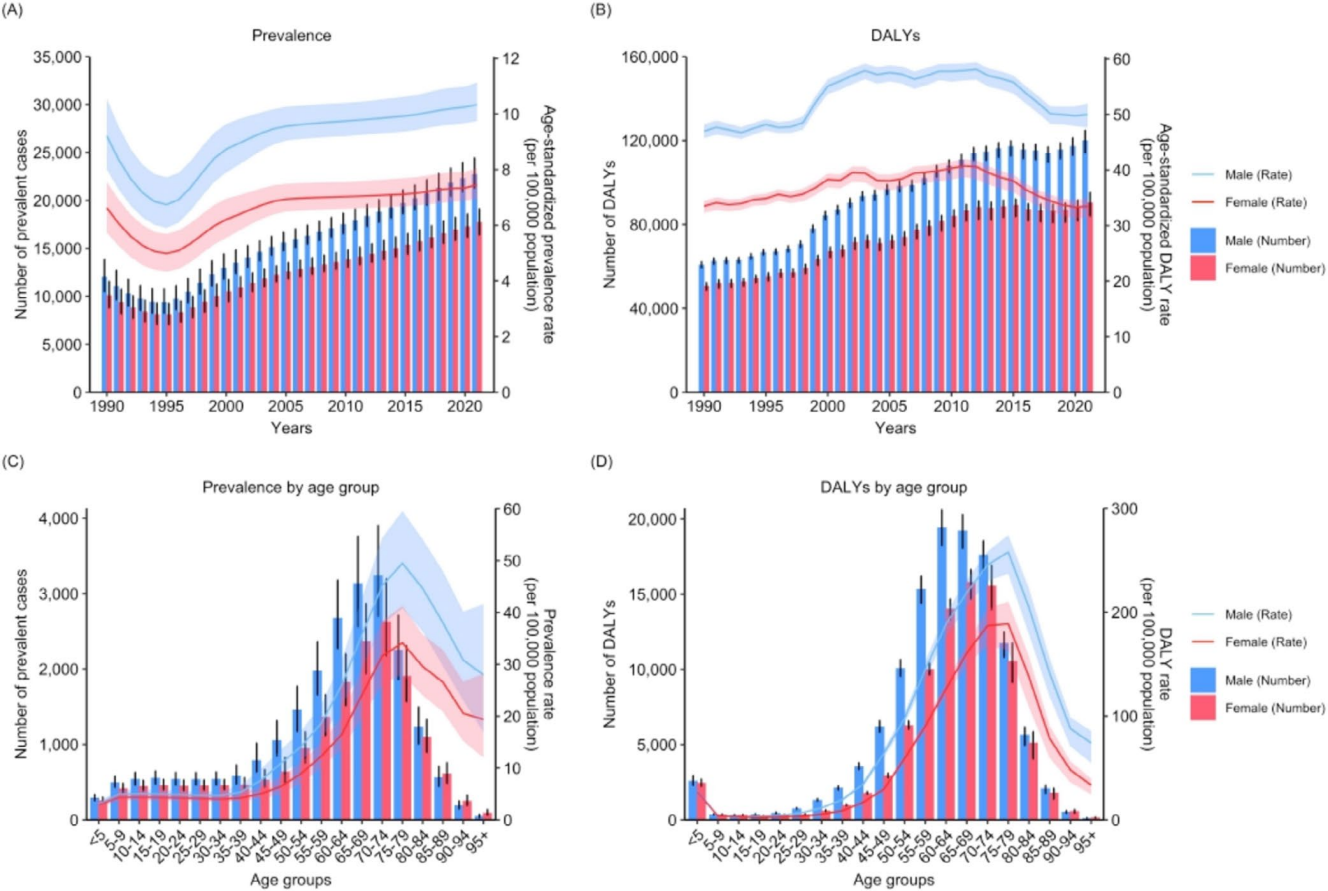
Numbers and age‐standardized rates of prevalence and DALYs from motor neuron disease by year, age group, and sex in the US. (A) Annual trends in the number of prevalent cases and age‐standardized prevalence rate from 1990 to 2021, by sex (B) Annual trends in the number of DALYs and age‐standardized DALY rate from 1990 to 2021, by sex. (C) Numbers and rates of prevalence in 2021, by age group and sex (D) Numbers and rates of DALYs in 2021, by age group and sex. DALYs, disability‐adjusted life years; US, United States.

In 2021, the age‐standardized rates for prevalence, DALYs, incidence, and mortality in males were approximately 1.4 times higher than those in females in the US (Table S4 and Figure S7). The prevalence and YLD rates appeared higher in males across all age groups, with the disparity becoming more pronounced in individuals older than 40 years. Similarly, the incidence rates appeared higher in males across all age groups, with the greatest disparity observed among individuals aged 20–60 years and those older than 85 years. DALY, YLL, and mortality rates were also higher in males than in females, except in children aged 5–14 years, where the rates were numerically higher in females. Further details are provided in Figure 5 and Table S5.

For both sexes, age‐specific rates of prevalence, incidence, mortality, DALYs, YLDs, and YLLs increased with age, with the highest rates per 100,000 observed in the 75–79‐year age group (Table S6). Sex‐specific analyses revealed comparable trends in both sexes, except for the mortality rate in males, which peaked in the 80–84 year age group (Table S5). Similarly, across the nine Census divisions, the 75–79‐year age group generally exhibited the highest rates, except for mortality in New England, East North Central, and Pacific, and incidence in Mountain, which peaked at 80–84 years (Figure S8). The number of deaths, incident cases, and YLLs was highest among individuals aged 70–74 years. The number of DALYs and YLLs was highest in the 65–69‐year age group for both sexes, whereas both metrics peaked in the 60–64‐year age group among males (Figure 5 and Figure S9). In the two age groups divided at 70 years, the rates of most metrics increased over time (Figure S10).

During the pandemic period (2019–2021), prevalence rates increased across all age groups. However, DALY and mortality rates declined among younger age groups (i.e., < 5 years and 5–9 years). Notably, although DALY and mortality rates in the 5–9‐year age group had been increasing prior to the pandemic, both rates decreased during the pandemic, particularly among females (Figure S6).

### 3.5 Association Between the SDI and Motor Neuron Disease

**FIGURE 6:**
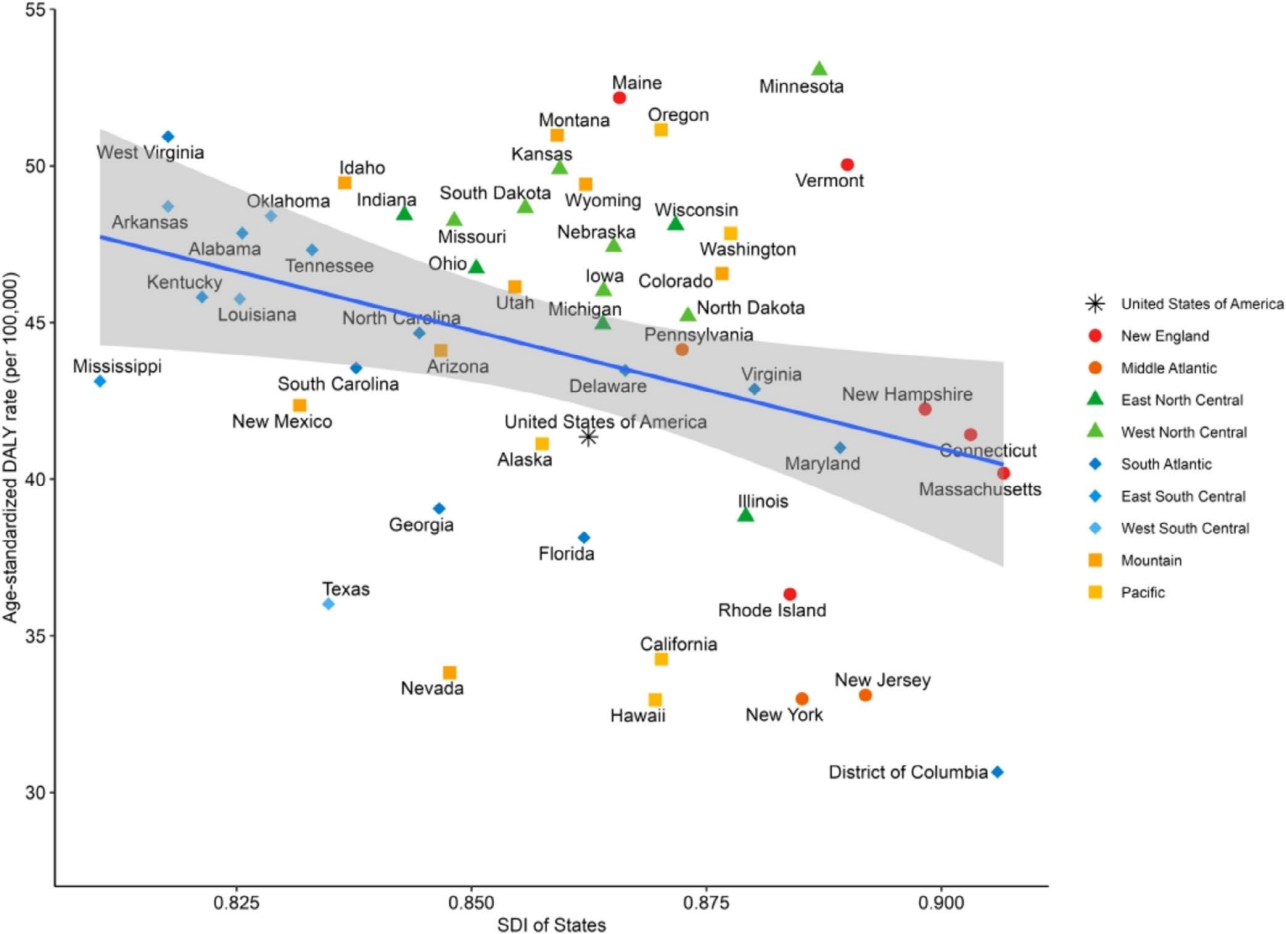
Age‐standardized DALY rates for motor neuron disease according to the sociodemographic index in 2021. DALY, disability‐adjusted life year; US, United States.

A significant correlation was observed between the SDI and age‐standardized DALYs for MNDs across the US. Specifically, regions with lower SDI tended to exhibit higher DALY rates in 2021, as demonstrated by linear regression analysis (β = −75.564, *p* = 0.019) (Figure 6). Spearman's correlation coefficient (𝜌) was −0.291, indicating a weak but significant negative correlation (*p* = 0.037) between the SDI and DALYs (Table S7). Notably, the correlation shifted from a positive value in 1990 (𝜌 = 0.481) to a negative value in 2021 (𝜌 = −0.291), reflecting a reversal in the relationship between the SDI and DALYs over time. From 1990 to 2021 across the nine divisions, SDI and the age‐standardized DALY rate showed an inverted U shape (Figure S11). For YLDs, a positive correlation with the SDI was observed in both 1990 (𝜌 = 0.702, *p* < 0.001) and 2021 (𝜌 = 0.468, p < 0.001). In contrast, YLLs exhibited a notable shift in correlation over time, changing from a positive correlation in 1990 (𝜌 = 0.459, *p* < 0.001) to a negative correlation in 2021 (𝜌 = −0.308, *p* = 0.027). Further details are provided in Figure S12 and Table S7.

## 4 Discussion

This study provided a comprehensive assessment of the burden of MNDs between 1990 and 2021 in the US at subnational levels by analyzing data from the GBD 2021. The age‐standardized prevalence rate was 8.82 per 100,000, which represented an arithmetic increase of 12.89% compared to 1990. The age‐standardized DALY rate was 41.36 per 100,000, a 4.14% increase from 1990. As MNDs may be age‐dependent conditions, the number of prevalent cases and DALYs exhibited greater increases than the corresponding age‐standardized rates.

### 4.1 Comparison With Global Trends

In the US, the age‐standardized incidence rate of MNDs increased from 1990 to 2021, diverging from the relatively stable global trend. Similar patterns have been observed in other high‐SDI countries, including Sweden [15], England [16], and Scotland [17]. While population aging and improved survival from other chronic conditions may have expanded the pool of individuals at risk [18], the increase in age‐standardized rates suggests a broader shift. Enhanced access to neurological care, increased neurologist density, and greater diagnostic awareness have likely facilitated earlier and more sensitive detection of overall MNDs.

The age‐standardized prevalence rate in the US remained consistently higher than the global rate from 1990 to 2021, which fluctuated between 3.1 and 3.5 during the same period. This trend could be attributed to advances in ALS treatment and management, including the introduction of riluzole [19], improvements in supportive care such as non‐invasive ventilation [20], and the increasing adoption of multidisciplinary care, which has been shown to prolong survival [21, 22]. In addition, the greater accessibility and lower costs of current genetic testing might have contributed to an increased apparent prevalence of genetically diagnosable conditions such as Kennedy's disease, spinal muscular atrophy, and distal hereditary motor neuropathy. Furthermore, the introduction of disease‐modifying therapies has improved survival among patients with spinal muscular atrophy, particularly in children, increasing the prevalence of the condition [23]. However, as the current GBD framework did not differentiate MND subtypes in the publicly available estimates, the trends reported in this study might have reflected overlapping contributions from multiple conditions. For instance, the prevalence of post‐polio syndrome might have declined during the study period, as some cases develop decades after polio infection (so likely many some cases were diagnosed), but also a number of individuals previously diagnosed are likely to have died during the course of this data collection. The implementation of routine polio vaccination in the US may have also contributed to the decline in the prevalence of post‐polio syndrome by preventing new cases of polio. As a result, trends in less common subtypes might have been masked.

### 4.2 Comparison Among the US, Europe, and Asia

The US had the highest age‐standardized burden of MNDs in both 1990 and 2021 compared to Asia and Europe. This disparity can be partly explained by environmental and genetic factors, as ALS incidence has been relatively uniform among Caucasian populations in Europe and North America but lower in African, Asian, and Hispanic populations [24]. Recent US data reported higher age‐adjusted mortality of MNDs in White individuals compared to other racial groups [25]. However, racial composition alone does not fully explain the elevated burden in the US, warranting further investigation.

### 4.3 Geographic Variation Within the US

In 2021, the burden of MNDs varied substantially across the US states. The mean density of neurologists varies by as much as fourfold [26]. Therefore, differences in MND burden observed across states—and similarly across broader regions and divisions—might reflect disparities in neurologist availability and diagnostic capacity rather than true differences in disease occurrence. The geographic heterogeneity in MND burden might also reflect differences in racial composition, diagnostic access, survival duration, disease management, and social support systems.

### 4.4 Sex and Age Differences in Motor Neuron Disease

Age‐specific analysis showed that MND burden was consistently greater in males than females across all age groups, particularly among middle‐aged and older adults. These findings were consistent with previous studies [27] and might be explained by sex‐specific biological differences, such as hormonal influences, or higher exposure to occupational or environmental risk factors among males [28]. This sex‐related disparity emphasizes the need for tailored public health interventions and further research to explore the underlying mechanisms.

### 4.5 Association Between the SDI and Motor Neuron Disease

The relationships between the SDI and MND burden in this study were complex. Globally, higher SDI was associated with a greater MND burden. In contrast, in the US, states with higher SDI exhibited lower DALY rates. This might be because a more equitable distribution of healthcare resources in more advantaged states reduced premature deaths and shifted the impact of MNDs from early mortality to long‐term disability. As a result, the overall burden may have been lower, despite a greater number of individuals living with the condition. Nevertheless, as the SDI of US states exhibited a narrower range (0.810–0.907) than the global distribution (0.078–0.946), the SDI–DALY relationship observed in the US may reflect only a limited portion of the global pattern—one that may not align with trends observed across the entire global SDI range.

### 4.6 Public Health Implications

The findings of this study have public health implications for addressing the burden of MNDs in the US. Sharing best practices and advancing research on modifiable risk factors for MNDs could help reduce the overall burden. Integrating these findings into national health strategies could support equitable resource distribution, improve health outcomes, and ultimately enhance the quality of life for individuals with MNDs in the US.

## 5 Limitations

This study shared the general limitations associated with the design of GBD studies [11]. First, diagnosing MND is clinically challenging, and certain groups, such as older adults and ethnic minorities, may be missed. Second, the potential impact of subtypes of MNDs on our findings could not be evaluated, as the IHME provides only aggregate estimates for all subtypes combined. Third, as our dataset was not based on cluster random sampling but derived from ICD‐coded health claims data and systematic reviews, statistically valid state‐by‐state comparisons could not be performed. Fourth, within the GBD framework, major changes over time—such as the transition from ICD‐9 to ICD‐10, the approval of riluzole and edaravone, and inclusion of ALS as a service‐related condition by the Veterans Affairs Administration—were not incorporated, which might have influenced case reporting and introduced potential bias in case ascertainment over time. Also, data derived from diagnostic‐code‐based databases are subject to potential coding errors. In addition, although previous studies have supported the reliability of using ICD codes to identify MNDs [29, 30], reporting a positive predictive value of 92% (95% CI: 89%–95%) for ICD‐9 codes in the US hospital admission data, and a positive predictive value of 65% (95% CI: 59%–70%) with a sensitivity of 85% (95% CI: 80%–89%) for ICD‐10 codes in the US death certificate data [30], validation steps to confirm diagnostic accuracy could not be implemented as individual medical records were not available for systematic review. Fifth, although the GBD 2021 study defined the El Escorial criteria as the main criteria for ALS diagnosis, ICD codes could be recorded based on clinical suspicion, even in the absence of an exact match to the diagnostic criteria. Therefore, a mismatch between ICD codes and diagnostic criteria may have occurred. Finally, the severity analysis in the GBD 2021 study was based solely on ALS patients, as no severity data were available for other MND subtypes. Consequently, these estimates might not fully represent disability across all MND subtypes.

## 6 Conclusions

Our study may provide insights into the burden of MNDs in the US, highlighting temporal, geographic, and sociodemographic trends. These findings suggest a potential need for targeted public health interventions, enhanced research efforts, and equitable distribution of healthcare resources for individuals with MNDs.

## Author Contributions

**Yun‐Seo Oh:** data curation, formal analysis, investigation, software, validation, visualization, writing – original draft, writing – review and editing. **Raon Jung:** data curation, formal analysis, investigation, software, validation, writing – original draft, writing – review and editing. **Dong Keon Yon:** conceptualization, investigation, funding acquisition, methodology, project administration, resources, supervision, writing – review and editing. **Min‐Seo Kim:** data curation, formal analysis, software, investigation, writing – review and editing. **Joon‐Ho Shin:** funding acquisition, investigation, writing – review and editing. **Jae Il Shin:** conceptualization, investigation, funding acquisition, methodology, resources, supervision, writing – review and editing. **Tae‐Jin Song:** conceptualization, investigation, funding acquisition, methodology, project administration, resources, supervision, validation, writing – review and editing.

## Ethics Statement

We confirm that we have read the *Muscle & Nerve* position on issues involved in ethical publication and affirm that this report is consistent with those guidelines.

## Conflicts of Interest

The authors declare no conflicts of interest.

## Supporting information


**Figure S1:** Percent contribution of YLLs and YLDs in all‐age DALYs in the US, from 1990 to 2021. Abbreviations: DALYs, disability‐adjusted life years; US, United States; YLDs, years lived with disability; YLLs, years of life lost
**Figure S2:** Age‐standardized rates of (A) prevalence, (B) DALYs, (C) deaths, (D) incidence, (E) YLDs, (F) YLLs in 1990 and 2021, by states and sex. Abbreviations: DALYs, disability‐adjusted life years; YLDs, years lived with disability; YLLs, years of life lost
**Figure S3:** Percentage change of (A) prevalence, (B) DALY, (C) death, (D) incidence, (E) YLDs, (F) YLLs, from 1990 to 2021. Abbreviations: DALYs, disability‐adjusted life years; YLDs, years lived with disability; YLLs, years of life lost
**Figure S4:** Percentage change in age‐standardized rates of (A) prevalence, (B) DALYs, (C) deaths, (D) incidence, (E) YLDs, (F) YLLs, from 2000 to 2010, and from 2010 to 2021. Abbreviations: DALYs, disability‐adjusted life years; YLDs, years lived with disability; YLLs, years of life lost
**Figure S5:** Percentage change in age‐standardized rates of (A) prevalence, (B) DALYs, (C) deaths, (D) incidence, (E) YLDs, (F) YLLs in pre‐pandemic (2010–2019) and pandemic (2019–2021) periods. Abbreviations: DALYs, disability‐adjusted life years; YLDs, years lived with disability; YLLs, years of life lost
**Figure S6:** Changes in rates of (A) prevalence, (B) DALYs, (C) deaths, (D) incidence, (E) YLDs, (F) YLLs in pre‐pandemic (2010–2019) and pandemic (2019–2021) periods, by age groups, sex and divisions. Abbreviations: DALYs, disability‐adjusted life years; YLDs, years lived with disability; YLLs, years of life lost
**Figure S7:** Annual trends of age‐standardized rate and numbers of (A) prevalence, DALYs, and deaths, (B) DALYs, YLDs, and YLLs from 1990 to 2021, by sex (shaded areas represent 95% uncertainty intervals). Abbreviations: DALYs, disability‐adjusted life years; YLDs, years lived with disability; YLLs, years of life lost
**Figure S8:** Rates of (A) prevalence, (B) DALYs, (C) deaths, (D) incidence, (E) YLDs, (F) YLLs in 2021, by age groups and divisions. Abbreviations: DALYs, disability‐adjusted life years; YLDs, years lived with disability; YLLs, years of life lost
**Figure S9:** Numbers and rates (per 100,000 population) of (A) incidence and deaths, (B) YLDs and YLLs according to year, age groups, and sex in the US. Abbreviations: US, United States; YLDs, years lived with disability; YLLs, years of life lost
**Figure S10:** Annual trends of rates for (A) prevalence, (B) DALYs, (C) deaths, (D) incidence, (E) YLDs, (F) YLLs from 1990 to 2021, stratified by two age groups (younger than 70 years and 70 years and older) (shaded areas are 95% uncertainty intervals). Abbreviations: DALYs, disability‐adjusted life years; YLDs, years lived with disability; YLLs, years of life lost
**Figure S11:** Age‐standardized (A) prevalence, (B) DALY, (C) death, (D) incidence, (E) YLD, (F) YLL rates for motor neuron disease from 1990 to 2021 in nine divisions of the US according to the sociodemographic index. Abbreviations: DALYs, disability‐adjusted life years; SDI, sociodemographic index; US, United States; YLDs, years lived with disability; YLLs, years of life lost
**Figure S12:** Age‐standardized rates of (A) prevalence, (B) deaths, (C) incidence, (D) YLDs, (E) YLLs according to the sociodemographic index in 2021. The line represents the linear regression model, and the shaded area indicates the 95% confidence interval. Abbreviations: SDI, sociodemographic index; YLDs, years lived with disability; YLLs, years of life lost.

**Table S1:** Numbers, age‐standardized rates, and percentage changes of incidence, prevalence, deaths, DALYs, YLDs, and YLLs, from 1990 to 2021, in US, global, Asia, and Europe
**Table S2a:** Annual age‐standardized prevalence rates and percentage changes from 1990 to 2021, by sex
**Table S2b:** Annual age‐standardized disability‐adjusted life year (DALY) rates and percentage changes from 1990 to 2021, by sex
**Table S2c:** Annual age‐standardized mortality rates and percentage changes from 1990 to 2021, by sex
**Table S2d:** Annual age‐standardized incidence rates and percentage changes from 1990 to 2021, by sex
**Table S2e:** Annual age‐standardized years lived with disability (YLD) rates and percentage changes from 1990 to 2021, by sex
**Table S2f:** Annual age‐standardized years of life lost (YLL) rates and percentage changes from 1990 to 2021, by sex
**Table S3:** Numbers and age‐standardized rates, and percentage changes of Prevalence, DALYs, and deaths in the US.
**Table S4a:** Numbers and age‐standardized rates of prevalence in 2021, and percentage changes from 1990 to 2021, by sex
**Table S4b:** Numbers and age‐standardized rates of disability‐adjusted life years (DALYs) in 2021, and percentage changes from 1990 to 2021, by sex
**Table S4c:** Numbers and age‐standardized rates of deaths in 2021, and percentage changes from 1990 to 2021, by sex
**Table S4d:** Numbers and age‐standardized rates of incidence in 2021, and percentage changes from 1990 to 2021, by sex
**Table S4e:** Numbers and age‐standardized rates of years lived with disability (YLDs) in 2021, and percentage changes from 1990 to 2021, by sex
**Table S4f:** Numbers and age‐standardized rates of years of life lost (YLLs) in 2021, and percentage changes from 1990 to 2021, by sex
**Table S5a:** Prevalence, DALYs, and mortality rates in 2021, by age groups and sex
**Table S5b:** Incidence, YLD, and YLL rates in 2021, by age groups and sex
**Table S6:** Rates of prevalence, DALYs, mortality, incidence, YLD, and YLL in 2021, by age groups
**Table S7a:** Numbers and age‐standardized rates of prevalence, DALYs, and deaths in 2021, and percentage changes from 1990 to 2021, with SDI
**Table S7b:** Numbers and age‐standardized rates of incidence, YLDs, and YLLs in 2021, and percentage changes from 1990 to 2021, with SDI.

## Data Availability

The data that support the findings of this study are available from the GBD 2021 study. All data are freely accessible through the Global Health Data Exchange (https://ghdx.healthdata.org). Data used for this study are available from the corresponding author upon reasonable request.
