# Supplementary material for: The Burden of Motor Neuron Diseases in the United States, 1990–2021: A Systematic Analysis of the Global Burden of Disease Study 2021
Source: Muscle Nerve. 2025 Sep 10;72(5):1130–42. doi: 10.1002/mus.70023 (PMC12529030; doi:10.1002/mus.70023)
Supplement: Supplementary file 1 — Figure S1: Percent contribution of YLLs and YLDs in all‐age DALYs in the US, from 1990 to 2021. Abbreviations: DALYs, disability‐adjusted life years; US, United States; YLDs, years lived with disability; YLLs, years of life lost Figure S2: Age‐standardized rates of (A) prevalence, (B) DALYs, (C) deaths, (D) incidence, (E) YLDs, (F) YLLs in 1990 and 2021, by states and sex. Abbreviations: DALYs, disability‐adjusted life years; YLDs, years lived with disability; YLLs, years of life lost Figure S3: Percentage change of (A) prevalence, (B) DALY, (C) death, (D) incidence, (E) YLDs, (F) YLLs, from 1990 to 2021. Abbreviations: DALYs, disability‐adjusted life years; YLDs, years lived with disability; YLLs, years of life lost Figure S4: Percentage change in age‐standardized rates of (A) prevalence, (B) DALYs, (C) deaths, (D) incidence, (E) YLDs, (F) YLLs, from 2000 to 2010, and from 2010 to 2021. Abbreviations: DALYs, disability‐adjusted life years; YLDs, years lived with disability; YLLs, years of life lost Figure S5: Percentage change in age‐standardized rates of (A) prevalence, (B) DALYs, (C) deaths, (D) incidence, (E) YLDs, (F) YLLs in pre‐pandemic (2010–2019) and pandemic (2019–2021) periods. Abbreviations: DALYs, disability‐adjusted life years; YLDs, years lived with disability; YLLs, years of life lost Figure S6: Changes in rates of (A) prevalence, (B) DALYs, (C) deaths, (D) incidence, (E) YLDs, (F) YLLs in pre‐pandemic (2010–2019) and pandemic (2019–2021) periods, by age groups, sex and divisions. Abbreviations: DALYs, disability‐adjusted life years; YLDs, years lived with disability; YLLs, years of life lost Figure S7: Annual trends of age‐standardized rate and numbers of (A) prevalence, DALYs, and deaths, (B) DALYs, YLDs, and YLLs from 1990 to 2021, by sex (shaded areas represent 95% uncertainty intervals). Abbreviations: DALYs, disability‐adjusted life years; YLDs, years lived with disability; YLLs, years of life lost Figure S8: Rates of (A) prevalence, (B) DALYs, [file MUS-72-1130-s002.pdf]

Figure S1: Percent contribution of YLLs and YLDs in all-age DALYs in the US, from 1990 to 2021.

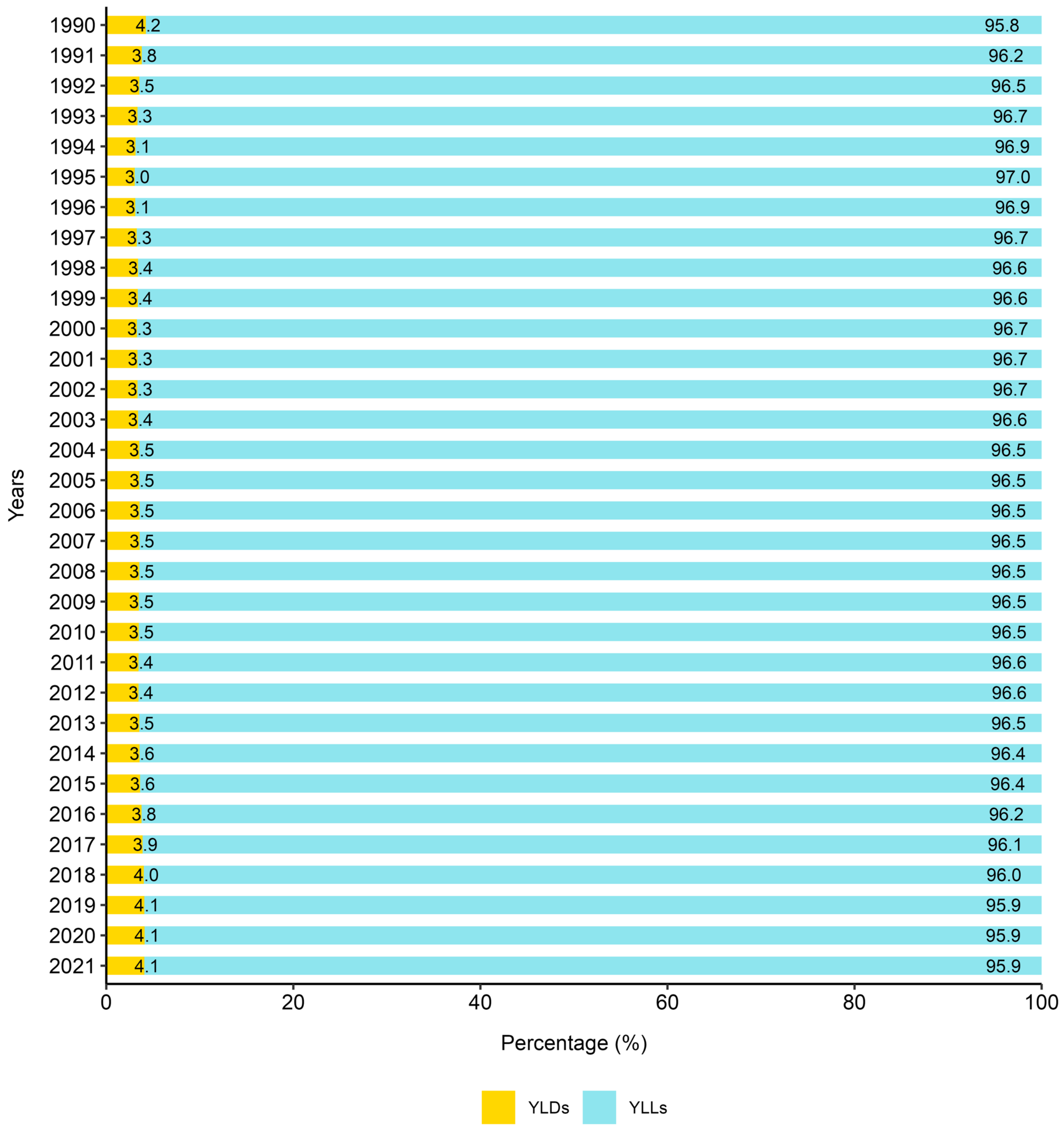

Abbreviations: DALYs, disability-adjusted life years; US, United States; YLDs, years lived with disability; YLLs, years of life lost.

Figure S2: Age-standardized rates of (A) prevalence, (B) DALYs, (C) deaths, (D) incidence, (E) YLDs, (F) YLLs in 1990 and 2021, by states and sex.

(A) prevalence

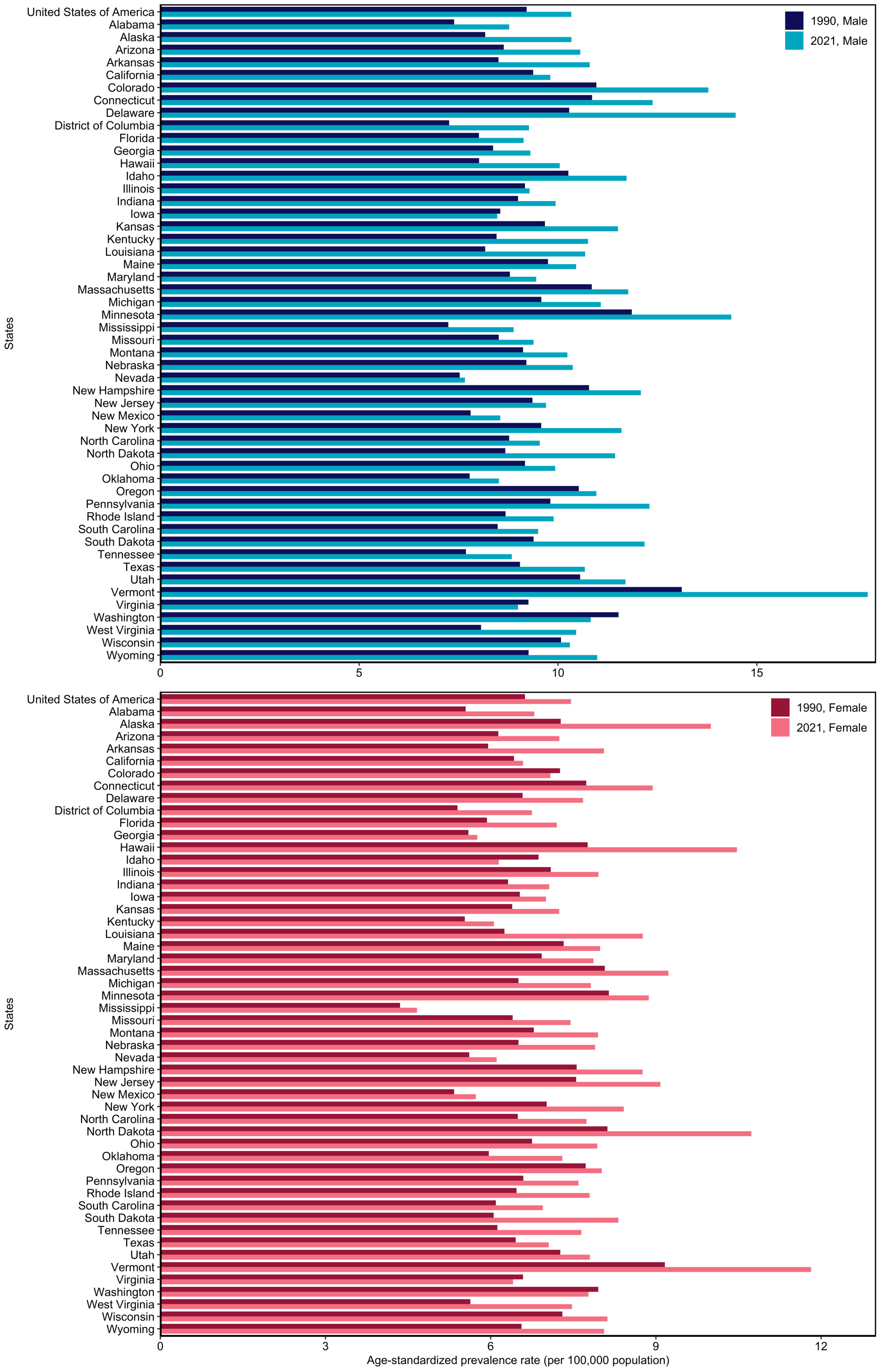

Abbreviations: DALYs, disability-adjusted life years; YLDs, years lived with disability; YLLs, years of life lost.

(B) DALYs

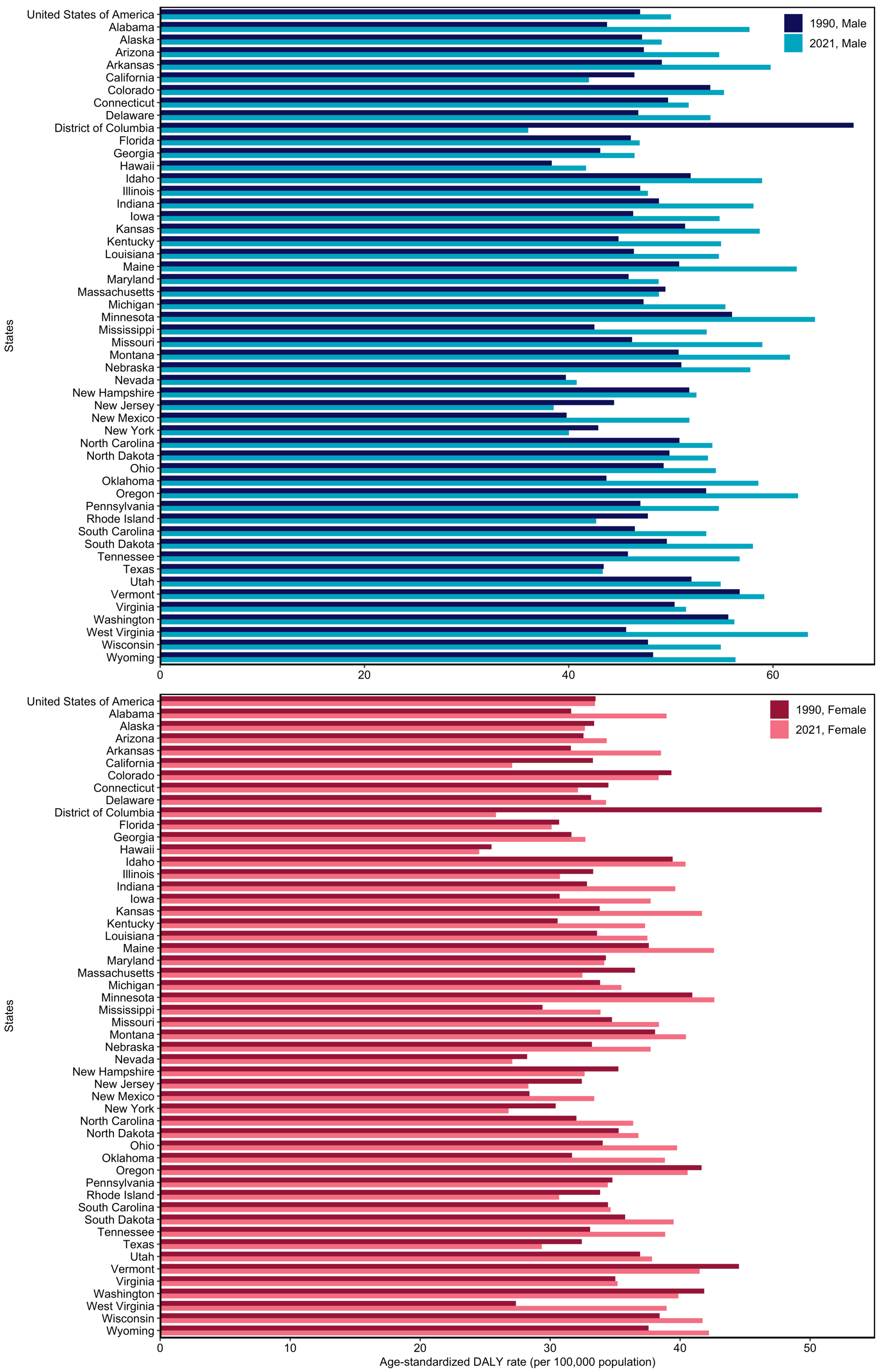

Abbreviations: DALYs, disability-adjusted life years.

(C) deaths

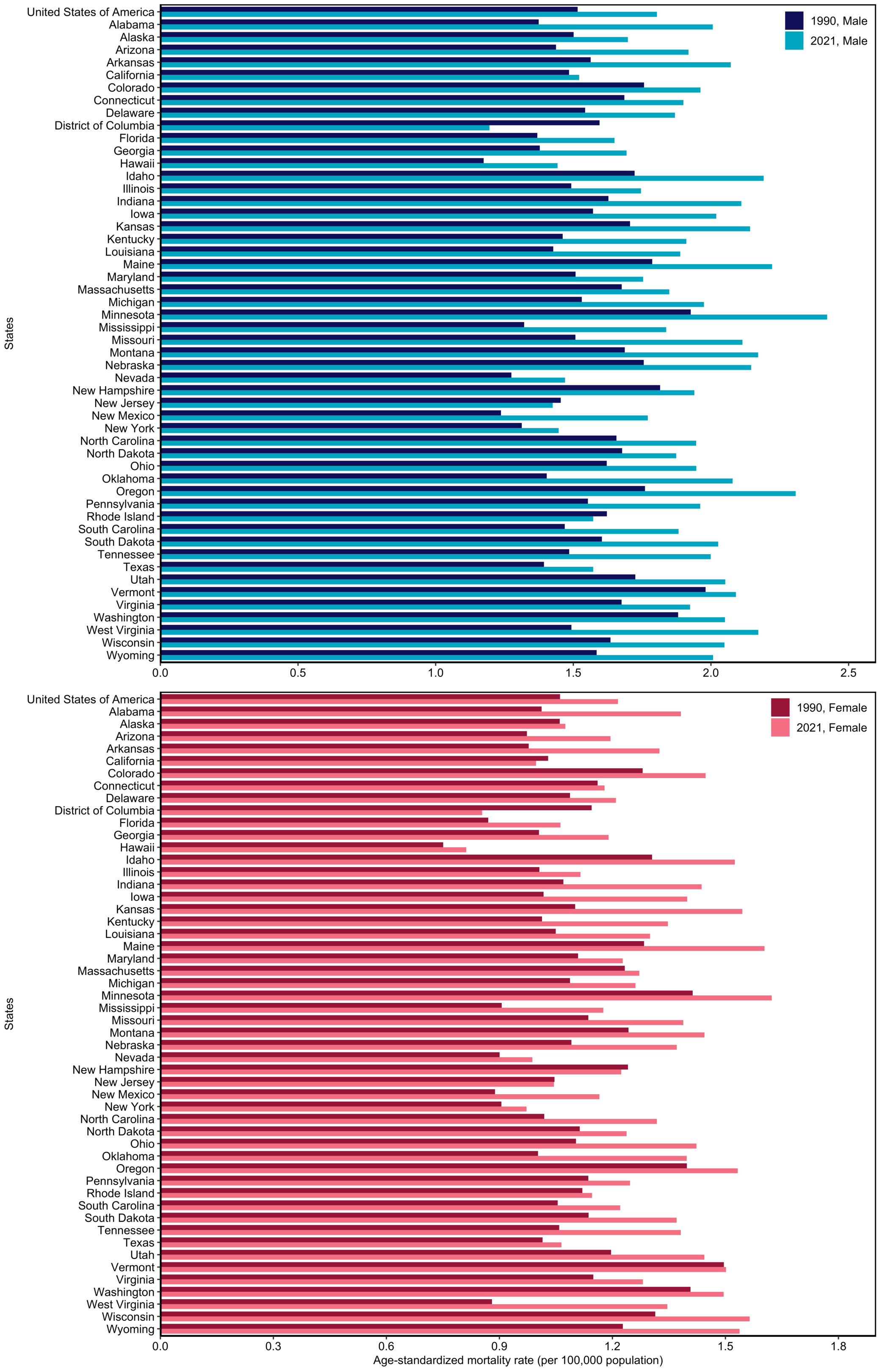

1990, Male
2021, Male
1990, Female
2021, Female
States
United States of America
Alabama
Alaska
Arizona
Arkansas
California
Colorado
Connecticut
Delaware
District of Columbia
Florida
Georgia
Hawaii
Idaho
Illinois
Indiana
Iowa
Kansas
Kentucky
Louisiana
Maine
Maryland
Massachusetts
Michigan
Minnesota
Mississippi
Missouri
Montana
Nebraska
Nevada
New Hampshire
New Jersey
New Mexico
New York
North Carolina
North Dakota
Ohio
Oklahoma
Oregon
Pennsylvania
Rhode Island
South Carolina
South Dakota
Tennessee
Texas
Utah
Vermont
Virginia
Washington
West Virginia
Wisconsin
Wyoming
0.0
0.5
1.0
1.5
2.0
2.5
0.3
0.6
0.9
1.2
1.8
Age-standardized mortality rate (per 100,000 population)

(D) incidence

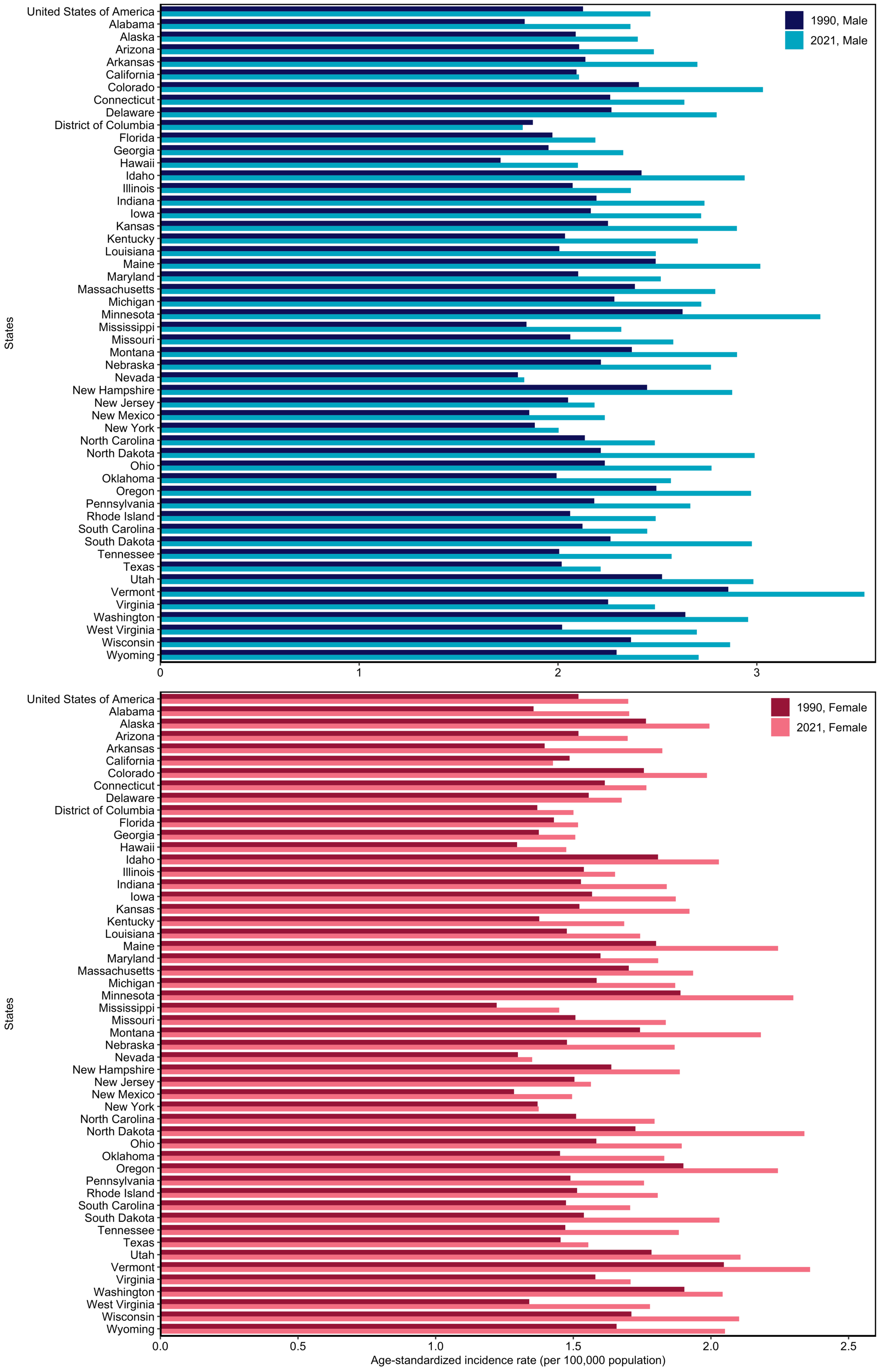

1990, Male
2021, Male
1990, Female
2021, Female
States
United States of America
Alabama
Alaska
Arizona
Arkansas
California
Colorado
Connecticut
Delaware
District of Columbia
Florida
Georgia
Hawaii
Idaho
Illinois
Indiana
Iowa
Kansas
Kentucky
Louisiana
Maine
Maryland
Massachusetts
Michigan
Minnesota
Mississippi
Missouri
Montana
Nebraska
Nevada
New Hampshire
New Jersey
New Mexico
New York
North Carolina
North Dakota
Ohio
Oklahoma
Oregon
Pennsylvania
Rhode Island
South Carolina
South Dakota
Tennessee
Texas
Utah
Vermont
Virginia
Washington
West Virginia
Wisconsin
Wyoming
0
1
2
3
0.0
0.5
1.0
1.5
2.0
2.5
Age-standardized incidence rate (per 100,000 population)

(E) YLDs

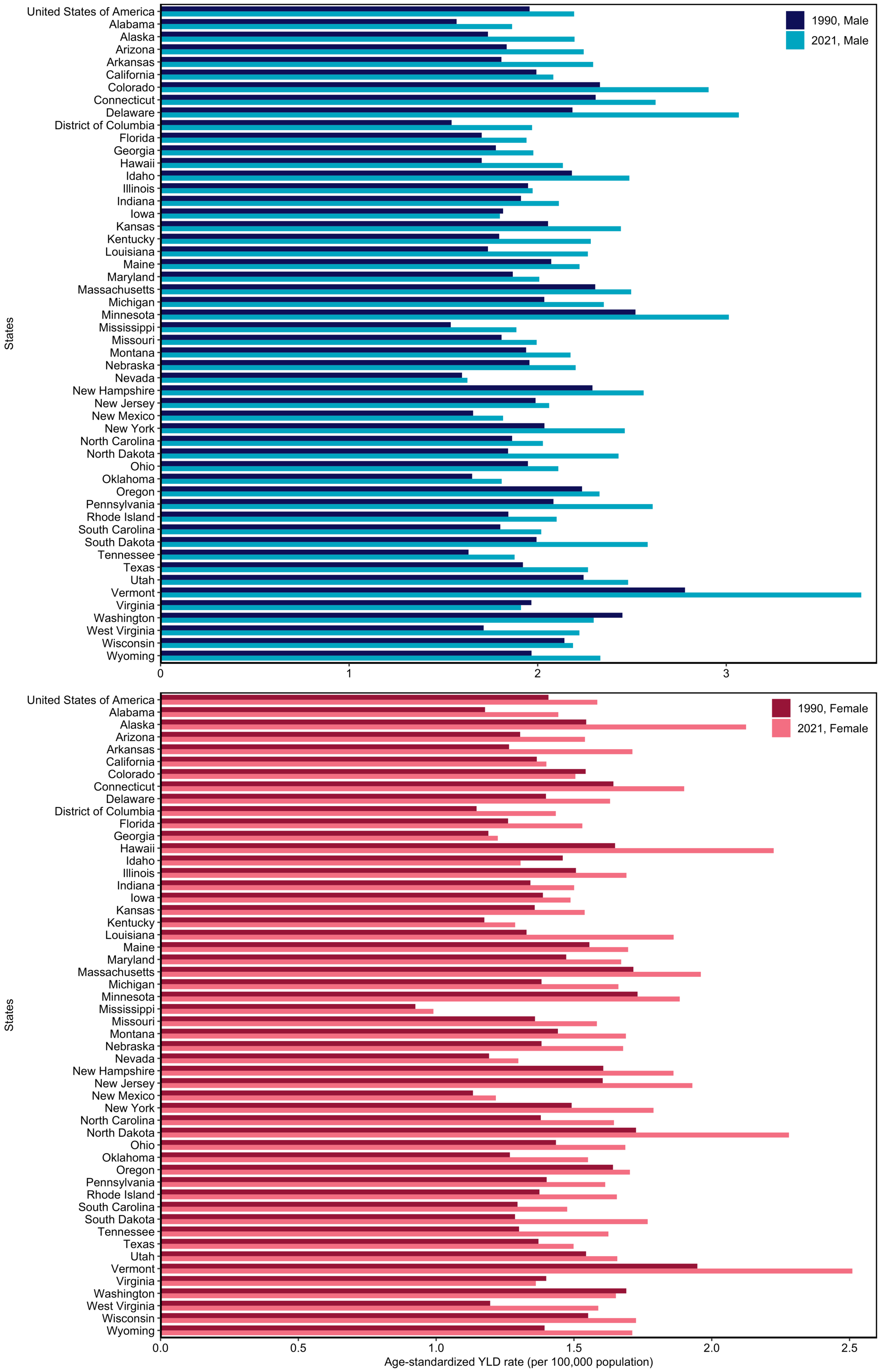

Abbreviations: YLDs, years lived with disability.

(F) YLLs

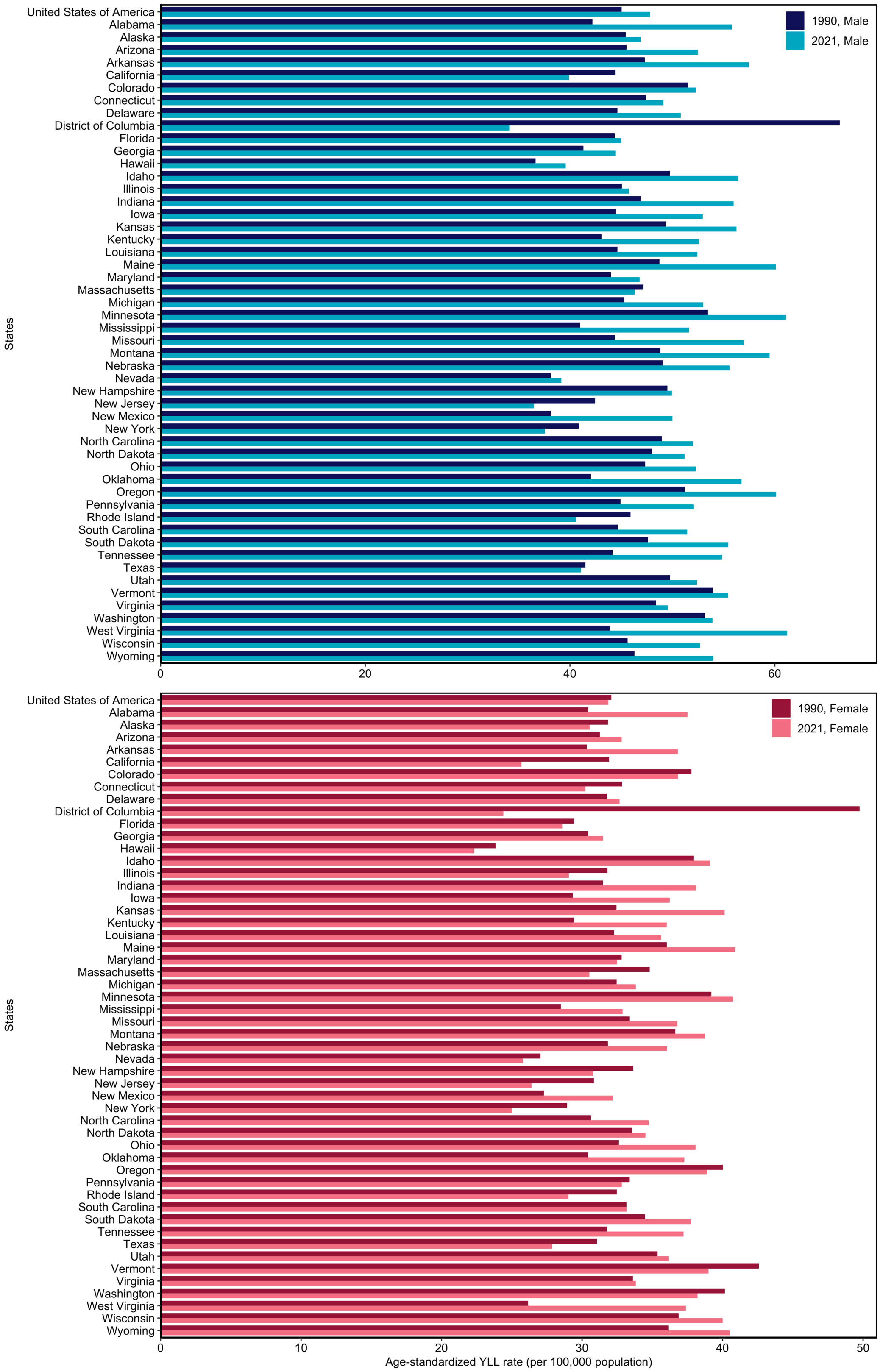

Abbreviations: YLLs, years of life lost.

Figure S3: Percentage change of (A) prevalence, (B) DALY, (C) death, (D) incidence, (E) YLDs, (F) YLLs, from 1990 to 2021.

(A) prevalence

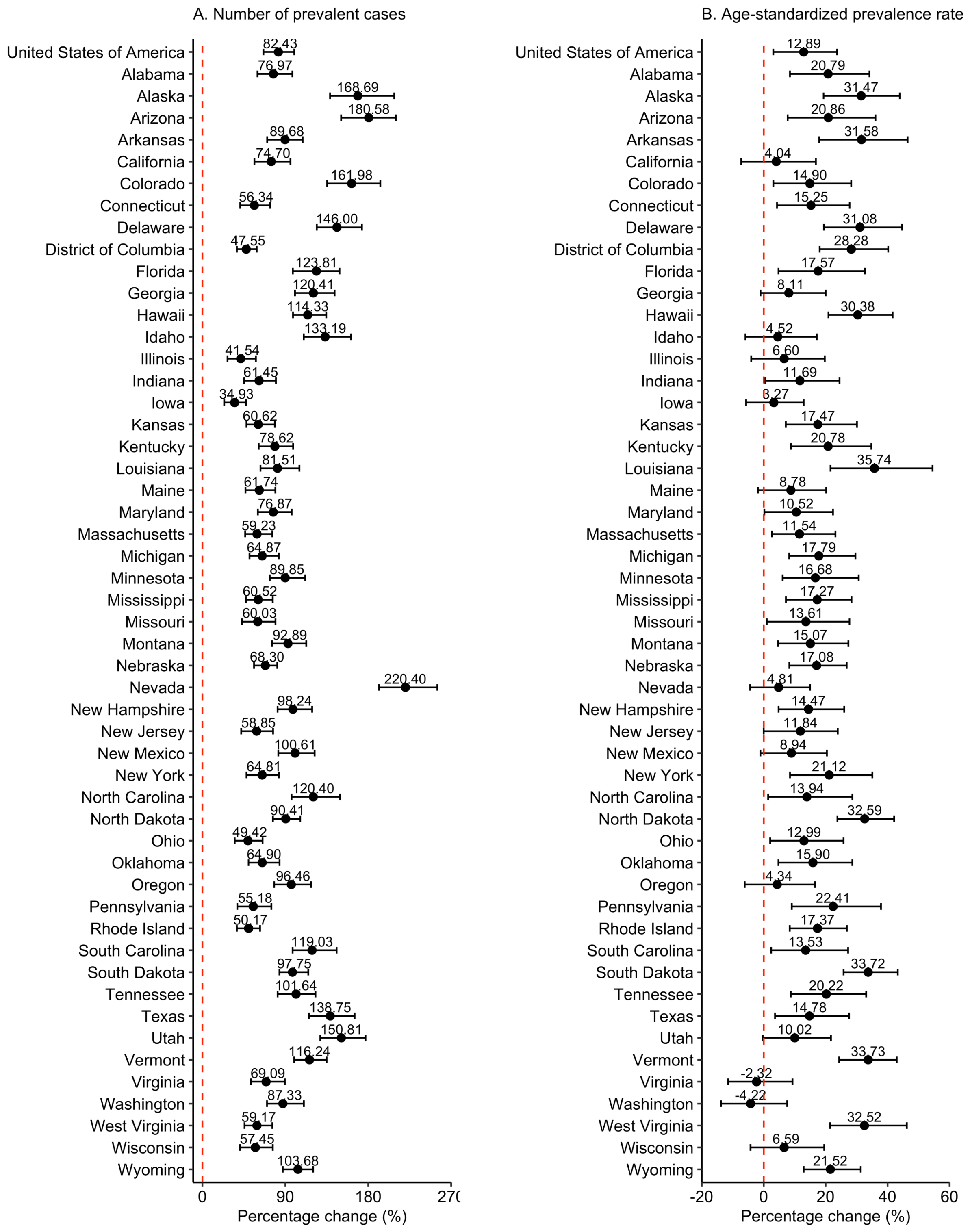

Abbreviations: DALYs, disability-adjusted life years; YLDs, years lived with disability; YLLs, years of life lost.

(B) DALY

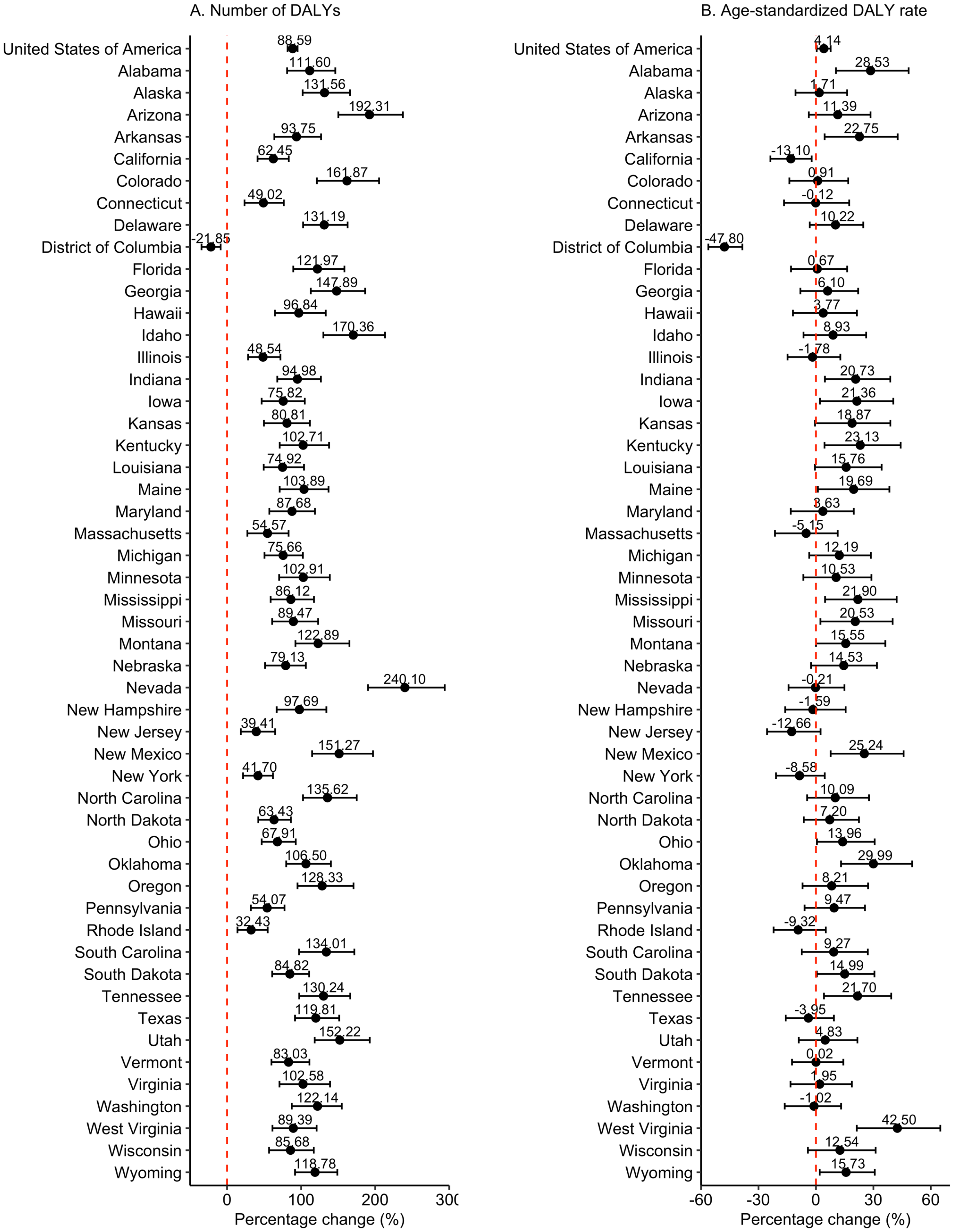

Abbreviations: DALYs, disability-adjusted life years.

(C) death

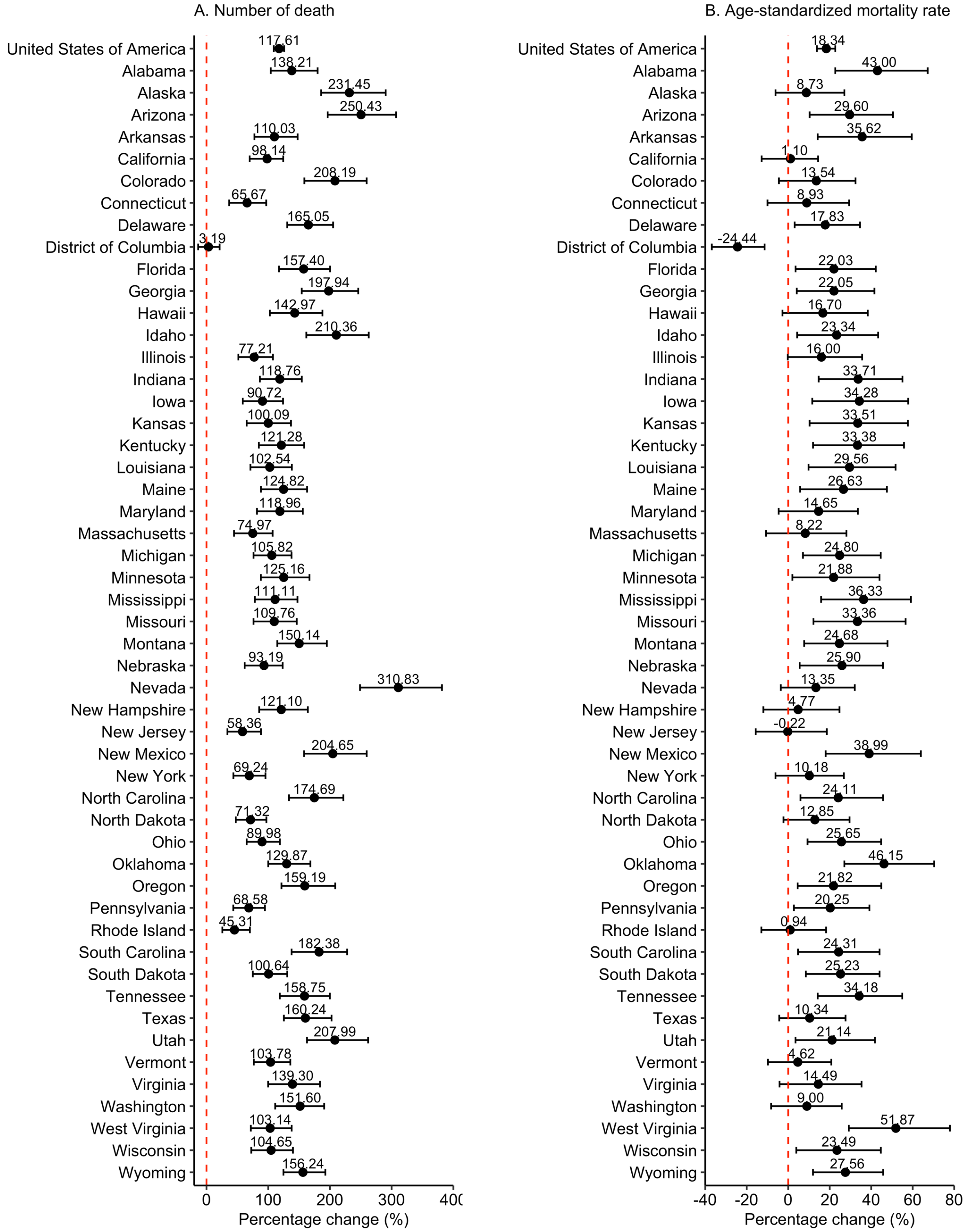
A. Number of death
B. Age-standardized mortality rate
United States of America
Alabama
Alaska
Arizona
Arkansas
California
Colorado
Connecticut
Delaware
District of Columbia
Florida
Georgia
Hawaii
Idaho
Illinois
Indiana
Iowa
Kansas
Kentucky
Louisiana
Maine
Maryland
Massachusetts
Michigan
Minnesota
Mississippi
Missouri
Montana
Nebraska
Nevada
New Hampshire
New Jersey
New Mexico
New York
North Carolina
North Dakota
Ohio
Oklahoma
Oregon
Pennsylvania
Rhode Island
South Carolina
South Dakota
Tennessee
Texas
Utah
Vermont
Virginia
Washington
West Virginia
Wisconsin
Wyoming
117.61
138.21
231.45
250.43
110.03
98.14
208.19
65.67
165.05
3.19
157.40
197.94
142.97
210.36
77.21
118.76
90.72
100.09
121.28
102.54
124.82
118.96
74.97
105.82
125.16
111.11
109.76
150.14
93.19
310.83
121.10
58.36
204.65
69.24
174.69
71.32
89.98
129.87
159.19
68.58
45.31
182.38
100.64
158.75
160.24
207.99
103.78
139.30
151.60
103.14
104.65
156.24
0
100
200
300
400
Percentage change (%)
18.34
43.00
8.73
29.60
35.62
1.10
13.54
8.93
17.83
-24.44
22.03
22.05
16.70
23.34
16.00
33.71
34.28
33.51
33.38
29.56
26.63
14.65
8.22
24.80
21.88
36.33
33.36
24.68
25.90
13.35
4.77
-0.22
38.99
10.18
24.11
12.85
25.65
46.15
21.82
20.25
0.94
24.31
25.23
34.18
10.34
21.14
4.62
14.49
9.00
51.87
23.49
27.56
-40
-20
0
20
40
60
80
Percentage change (%)

(D) incidence

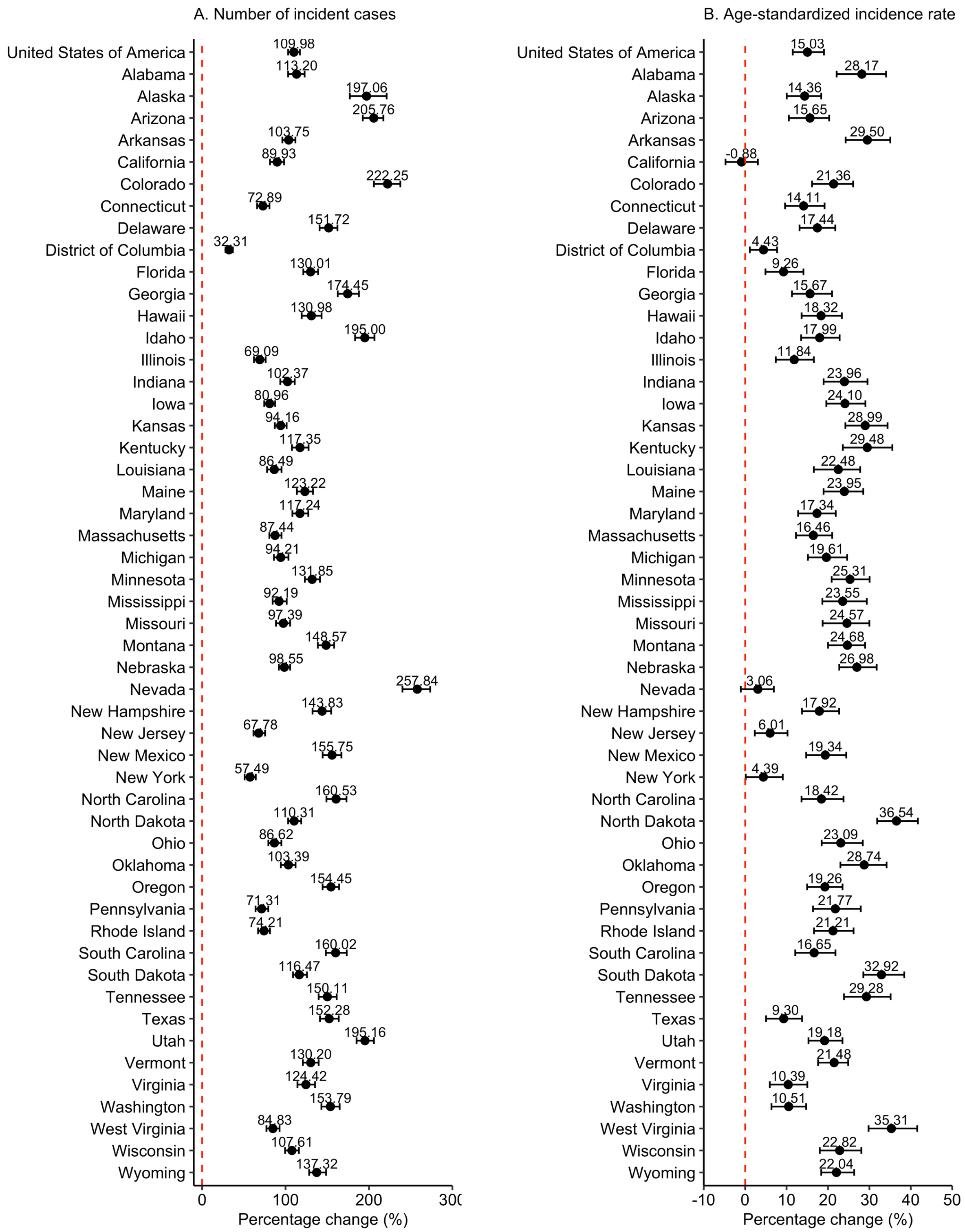
A. Number of incident cases
United States of America 109.98
Alabama 113.20
Alaska 197.06
Arizona 205.76
Arkansas 103.75
California 89.93
Colorado 222.25
Connecticut 72.89
Delaware 151.72
District of Columbia 32.31
Florida 130.01
Georgia 174.45
Hawaii 130.98
Idaho 195.00
Illinois 69.09
Indiana 102.37
Iowa 80.96
Kansas 94.16
Kentucky 117.35
Louisiana 86.49
Maine 123.22
Maryland 117.24
Massachusetts 87.44
Michigan 94.21
Minnesota 131.85
Mississippi 92.19
Missouri 97.39
Montana 148.57
Nebraska 98.55
Nevada 257.84
New Hampshire 143.83
New Jersey 67.78
New Mexico 155.75
New York 57.49
North Carolina 160.53
North Dakota 110.31
Ohio 86.62
Oklahoma 103.39
Oregon 154.45
Pennsylvania 71.31
Rhode Island 74.21
South Carolina 160.02
South Dakota 116.47
Tennessee 150.11
Texas 152.28
Utah 195.16
Vermont 130.20
Virginia 124.42
Washington 153.79
West Virginia 84.83
Wisconsin 107.61
Wyoming 137.32
0
100
200
Percentage change (%)
B. Age-standardized incidence rate
United States of America 15.03
Alabama 28.17
Alaska 14.36
Arizona 15.65
Arkansas 29.50
California -0.88
Colorado 21.36
Connecticut 14.11
Delaware 17.44
District of Columbia 4.43
Florida 9.26
Georgia 15.67
Hawaii 18.32
Idaho 17.99
Illinois 11.84
Indiana 23.96
Iowa 24.10
Kansas 28.99
Kentucky 29.48
Louisiana 22.48
Maine 23.95
Maryland 17.34
Massachusetts 16.46
Michigan 19.61
Minnesota 25.31
Mississippi 23.55
Missouri 24.57
Montana 24.68
Nebraska 26.98
Nevada 3.06
New Hampshire 17.92
New Jersey 6.01
New Mexico 19.34
New York 4.39
North Carolina 18.42
North Dakota 36.54
Ohio 23.09
Oklahoma 28.74
Oregon 19.26
Pennsylvania 21.77
Rhode Island 21.21
South Carolina 16.65
South Dakota 32.92
Tennessee 29.28
Texas 9.30
Utah 19.18
Vermont 21.48
Virginia 10.39
Washington 10.51
West Virginia 35.31
Wisconsin 22.82
Wyoming 22.04
-10
0
10
20
30
40
50
Percentage change (%)

(E) YLDs

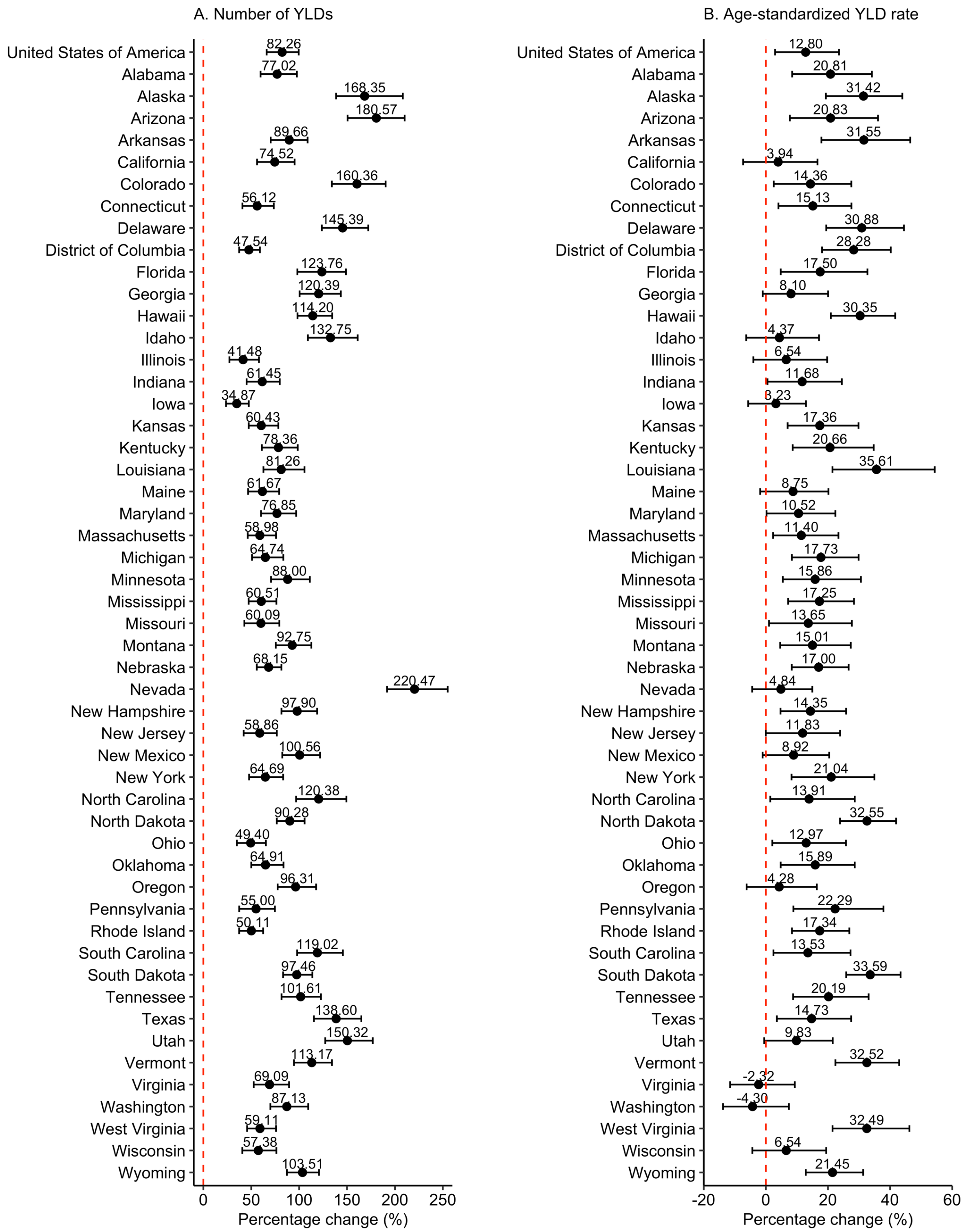

Abbreviations: YLDs, years lived with disability.

(F) YLLs

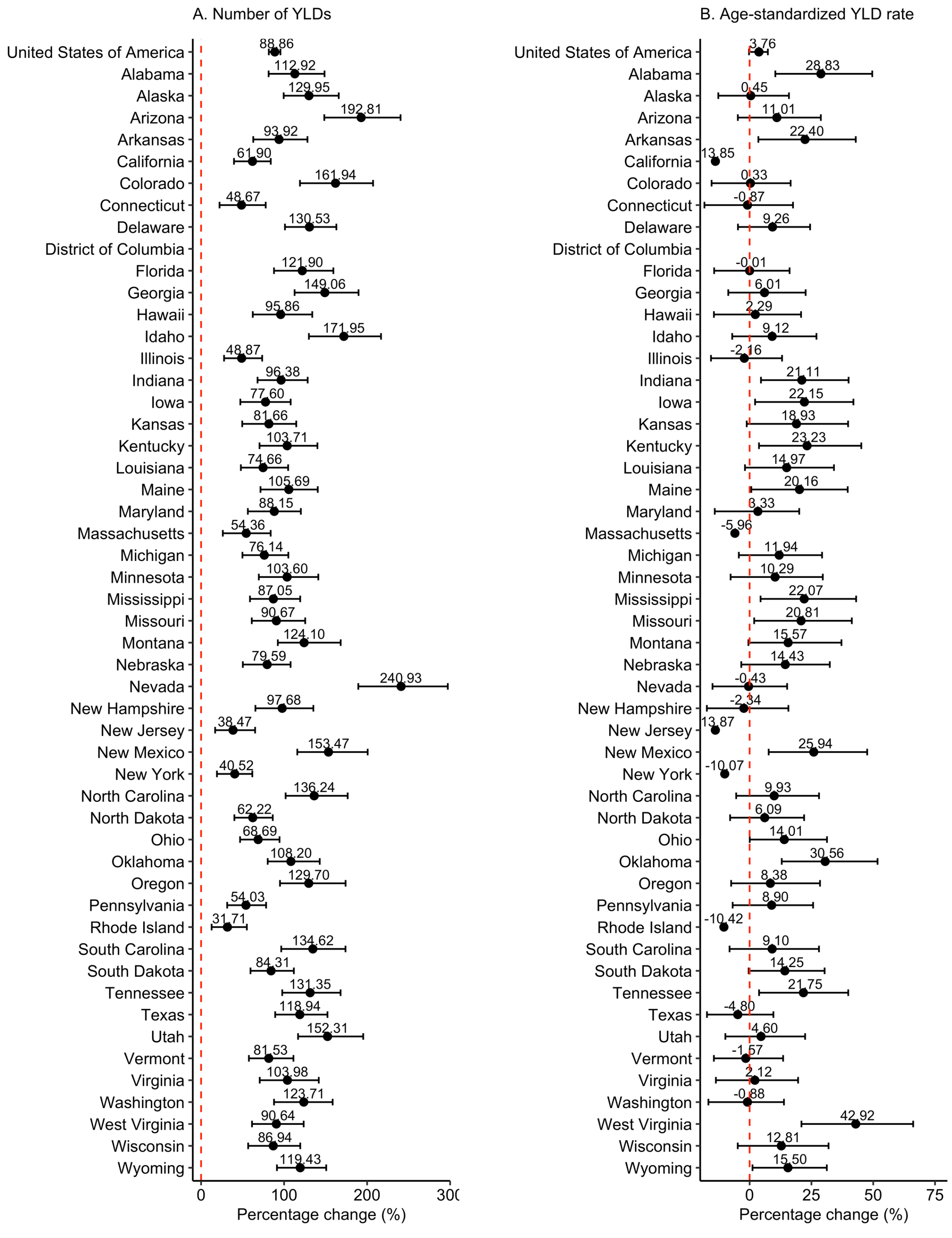

Abbreviations: YLLs, years of life lost.

Figure S4: Percentage change in age-standardized rates of (A) prevalence, (B) DALYs, (C) deaths, (D) incidence, (E) YLDs, (F) YLLs, from 2000 to 2010, and from 2010 to 2021.

(A) prevalence

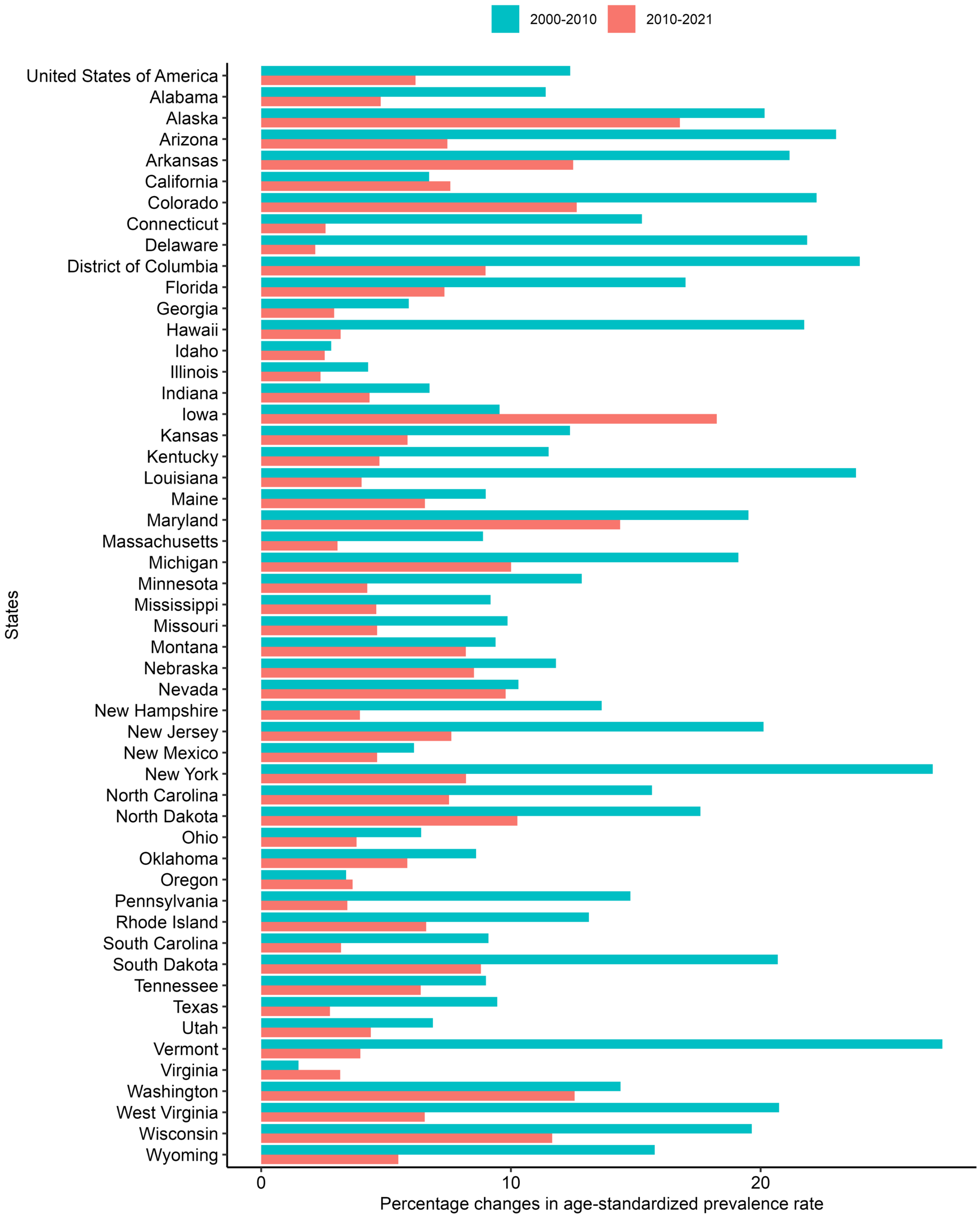

Abbreviations: DALYs, disability-adjusted life years; YLDs, years lived with disability; YLLs, years of life lost.

(B) DALYs

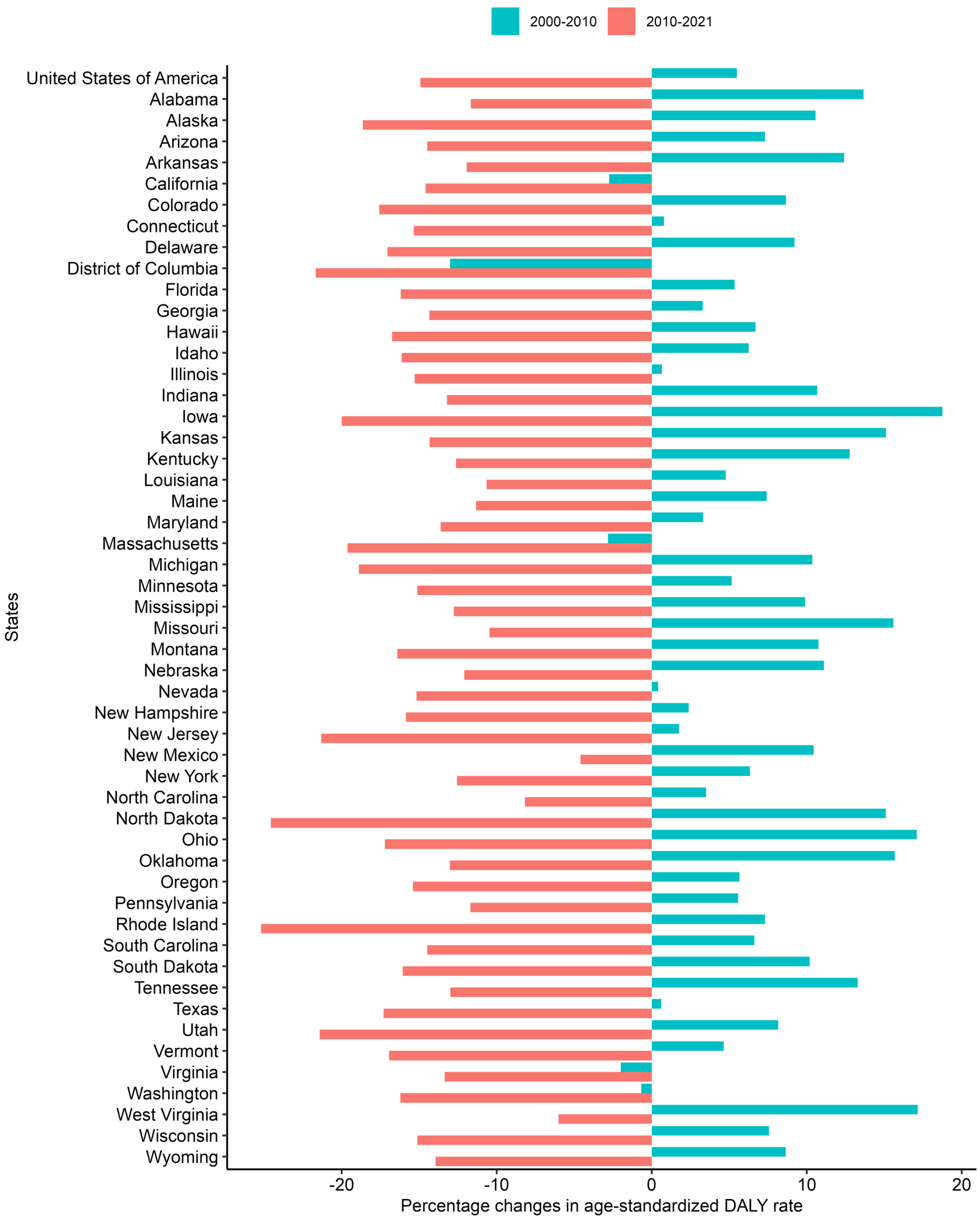

Abbreviations: DALYs, disability-adjusted life years

(C) deaths

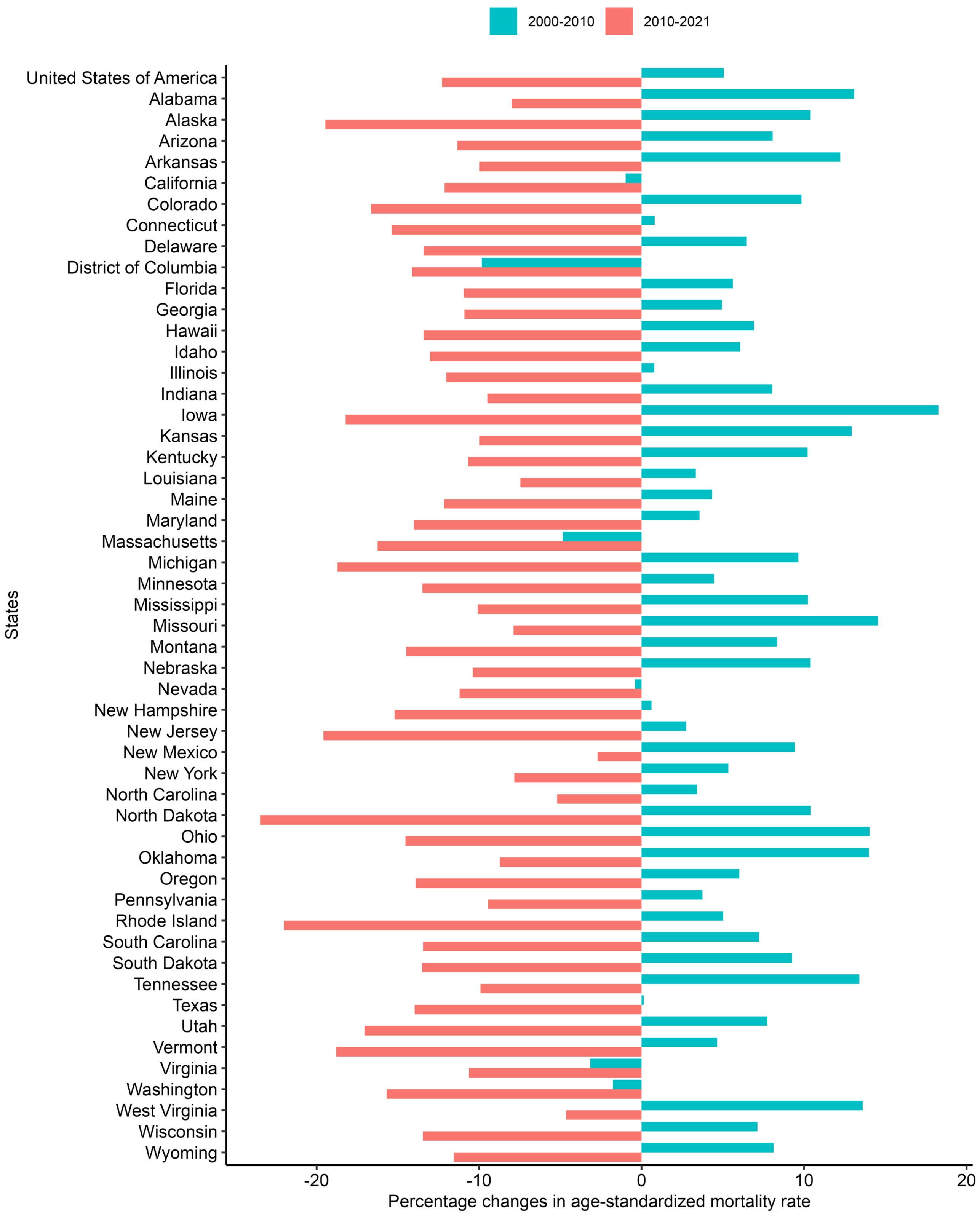
2000-2010
2010-2021
States
United States of America
Alabama
Alaska
Arizona
Arkansas
California
Colorado
Connecticut
Delaware
District of Columbia
Florida
Georgia
Hawaii
Idaho
Illinois
Indiana
Iowa
Kansas
Kentucky
Louisiana
Maine
Maryland
Massachusetts
Michigan
Minnesota
Mississippi
Missouri
Montana
Nebraska
Nevada
New Hampshire
New Jersey
New Mexico
New York
North Carolina
North Dakota
Ohio
Oklahoma
Oregon
Pennsylvania
Rhode Island
South Carolina
South Dakota
Tennessee
Texas
Utah
Vermont
Virginia
Washington
West Virginia
Wisconsin
Wyoming
-20
-10
0
10
20
Percentage changes in age-standardized mortality rate

(D) incidence

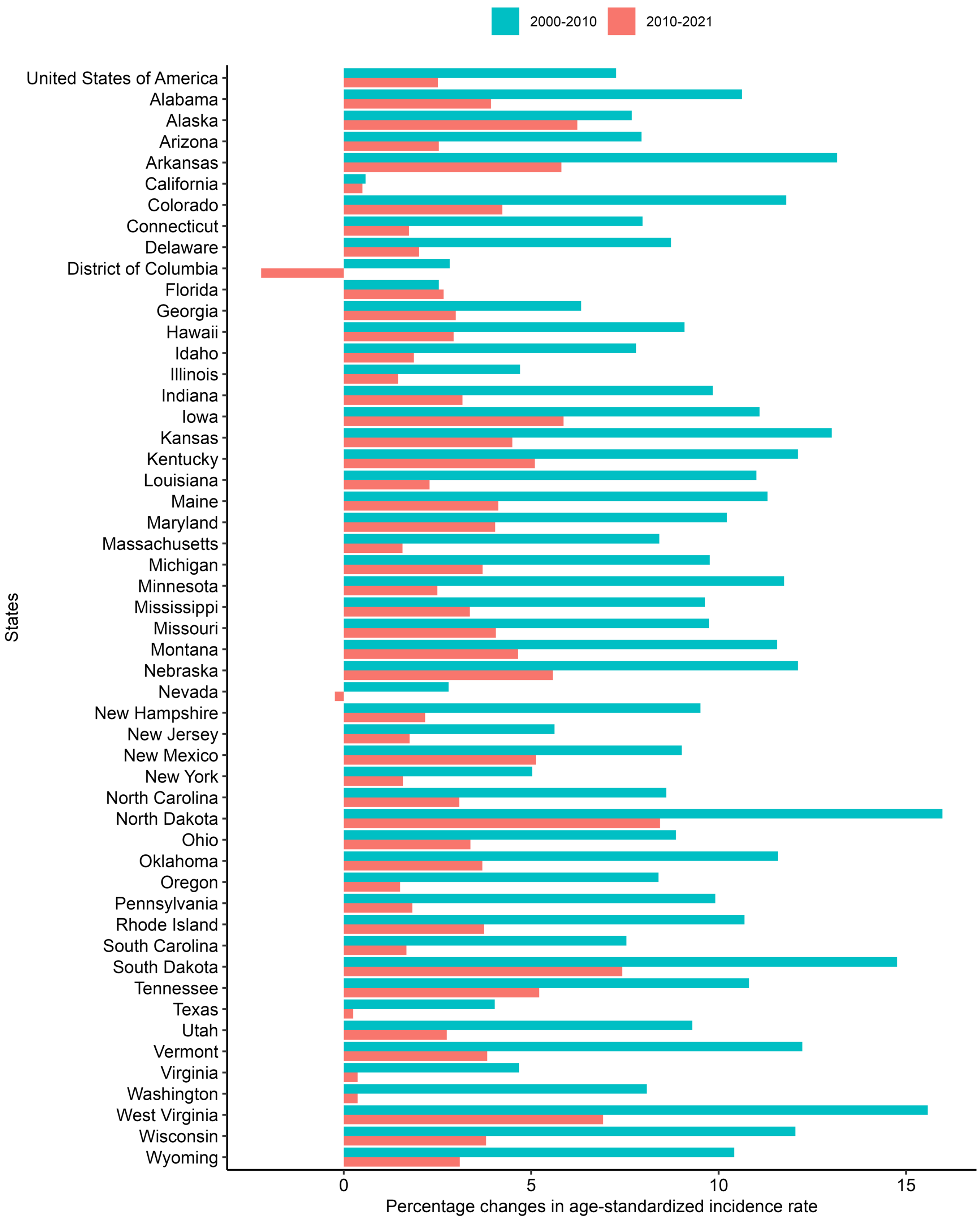

2000-2010
2010-2021
States
United States of America
Alabama
Alaska
Arizona
Arkansas
California
Colorado
Connecticut
Delaware
District of Columbia
Florida
Georgia
Hawaii
Idaho
Illinois
Indiana
Iowa
Kansas
Kentucky
Louisiana
Maine
Maryland
Massachusetts
Michigan
Minnesota
Mississippi
Missouri
Montana
Nebraska
Nevada
New Hampshire
New Jersey
New Mexico
New York
North Carolina
North Dakota
Ohio
Oklahoma
Oregon
Pennsylvania
Rhode Island
South Carolina
South Dakota
Tennessee
Texas
Utah
Vermont
Virginia
Washington
West Virginia
Wisconsin
Wyoming
0
5
10
15
Percentage changes in age-standardized incidence rate

(E) YLDs

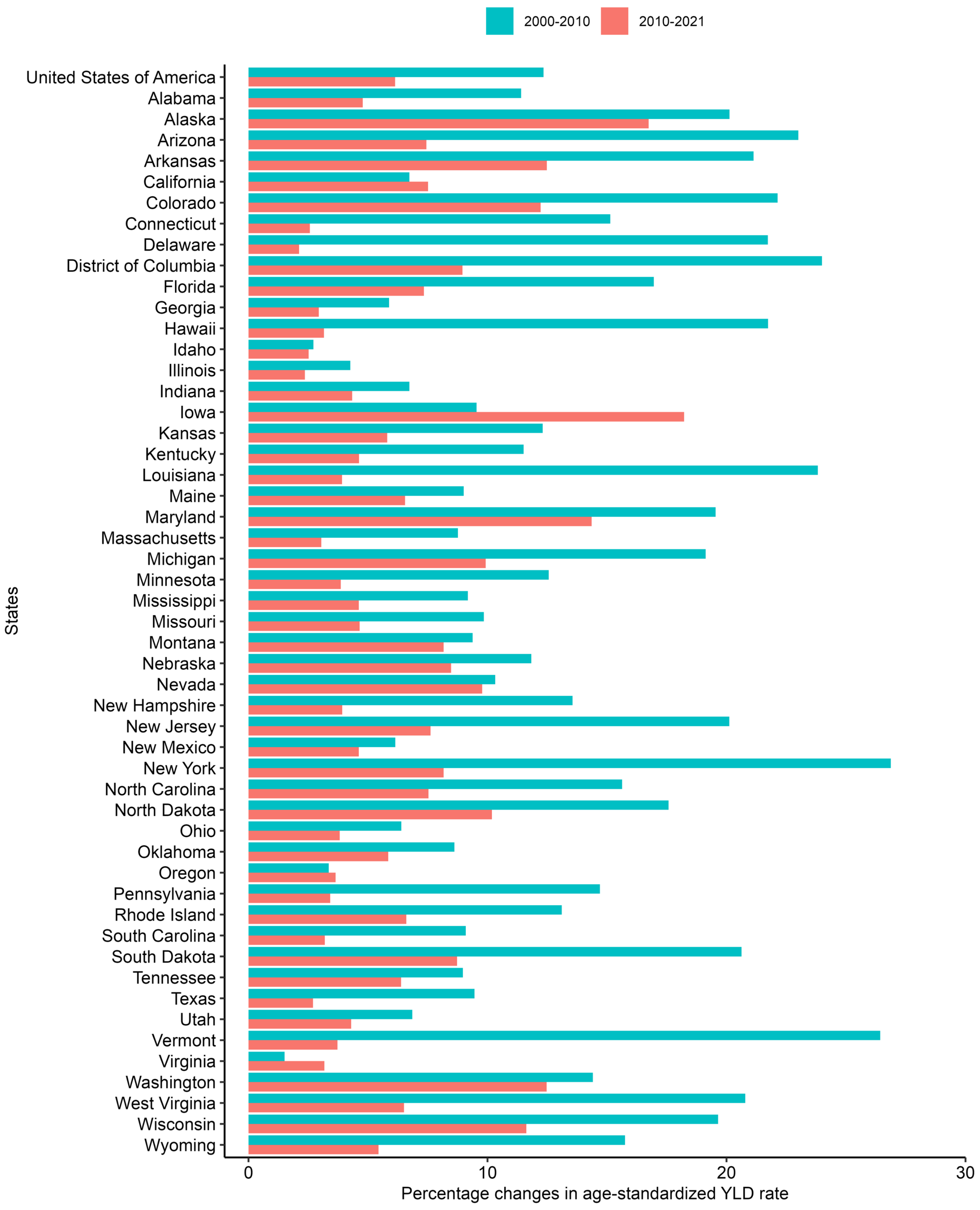

Abbreviations: YLDs, years lived with disability.

(F) YLLs

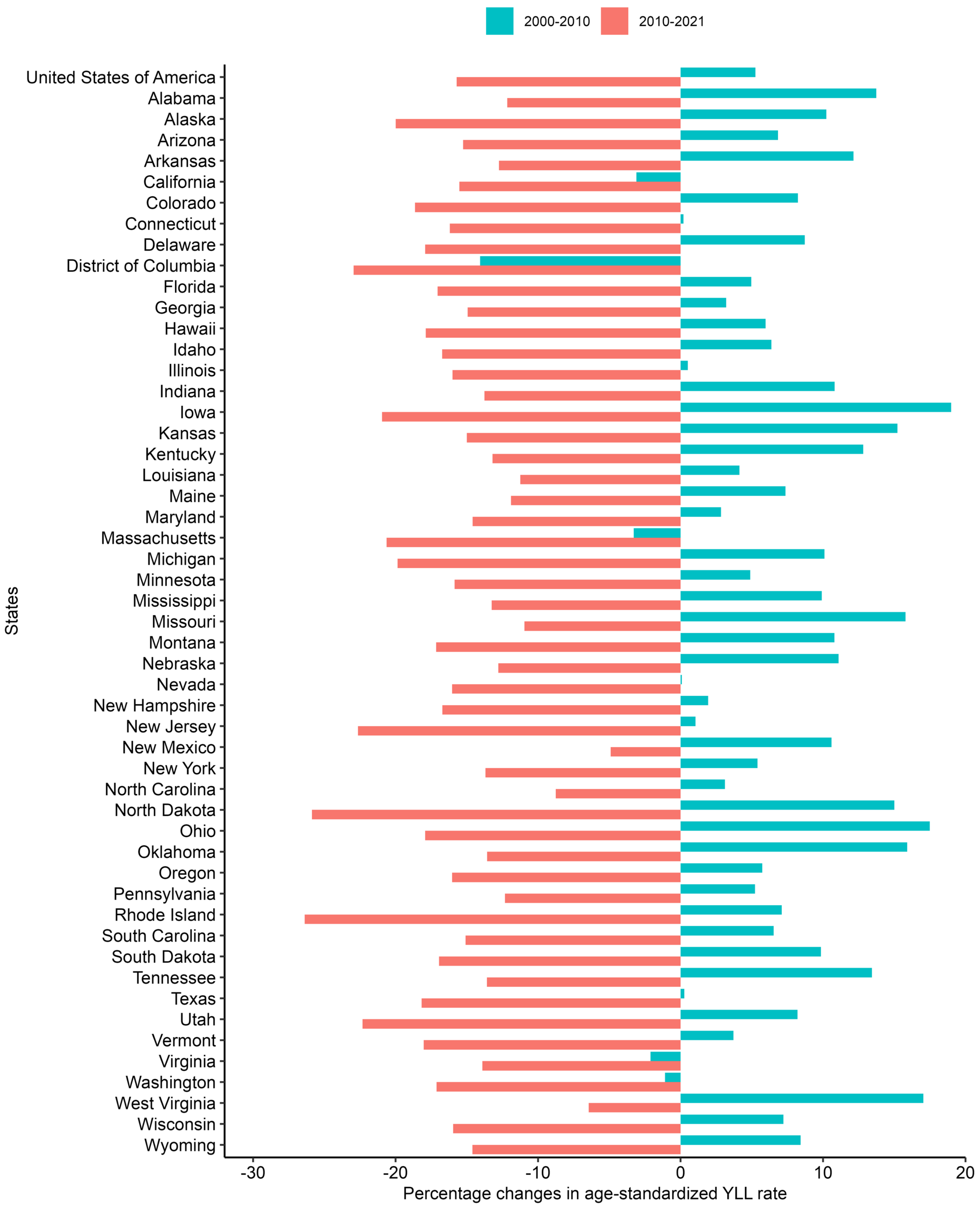

Abbreviations: YLLs, years of life lost.

Figure S5: Percentage change in age-standardized rates of (A) prevalence, (B) DALYs, (C) deaths, (D) incidence, (E) YLDs, (F) YLLs in pre-pandemic (2010-2019) and pandemic (2019-2021) periods.

(A) prevalence

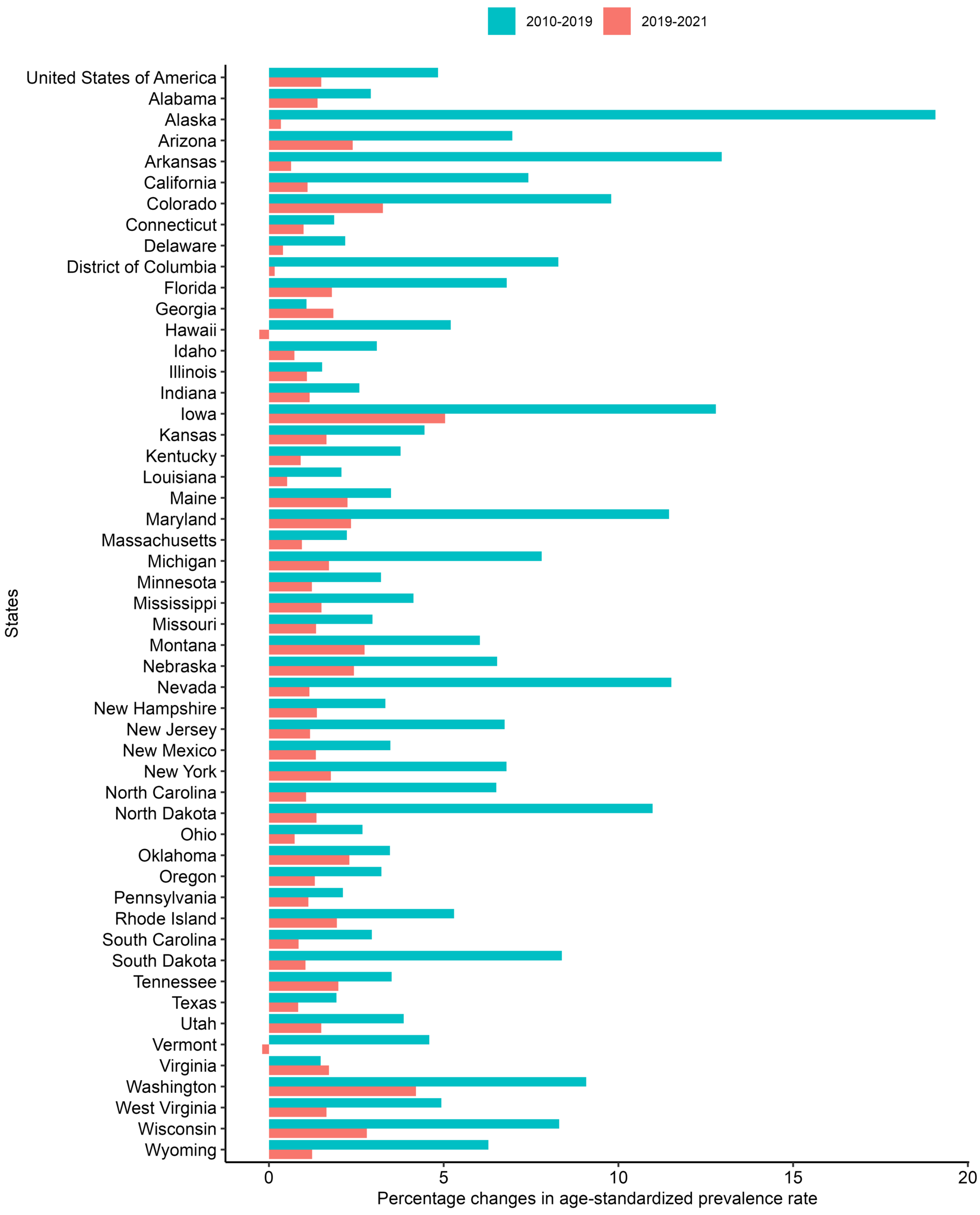

Abbreviations: DALYs, disability-adjusted life years; YLDs, years lived with disability; YLLs, years of life lost.

(B) DALYs

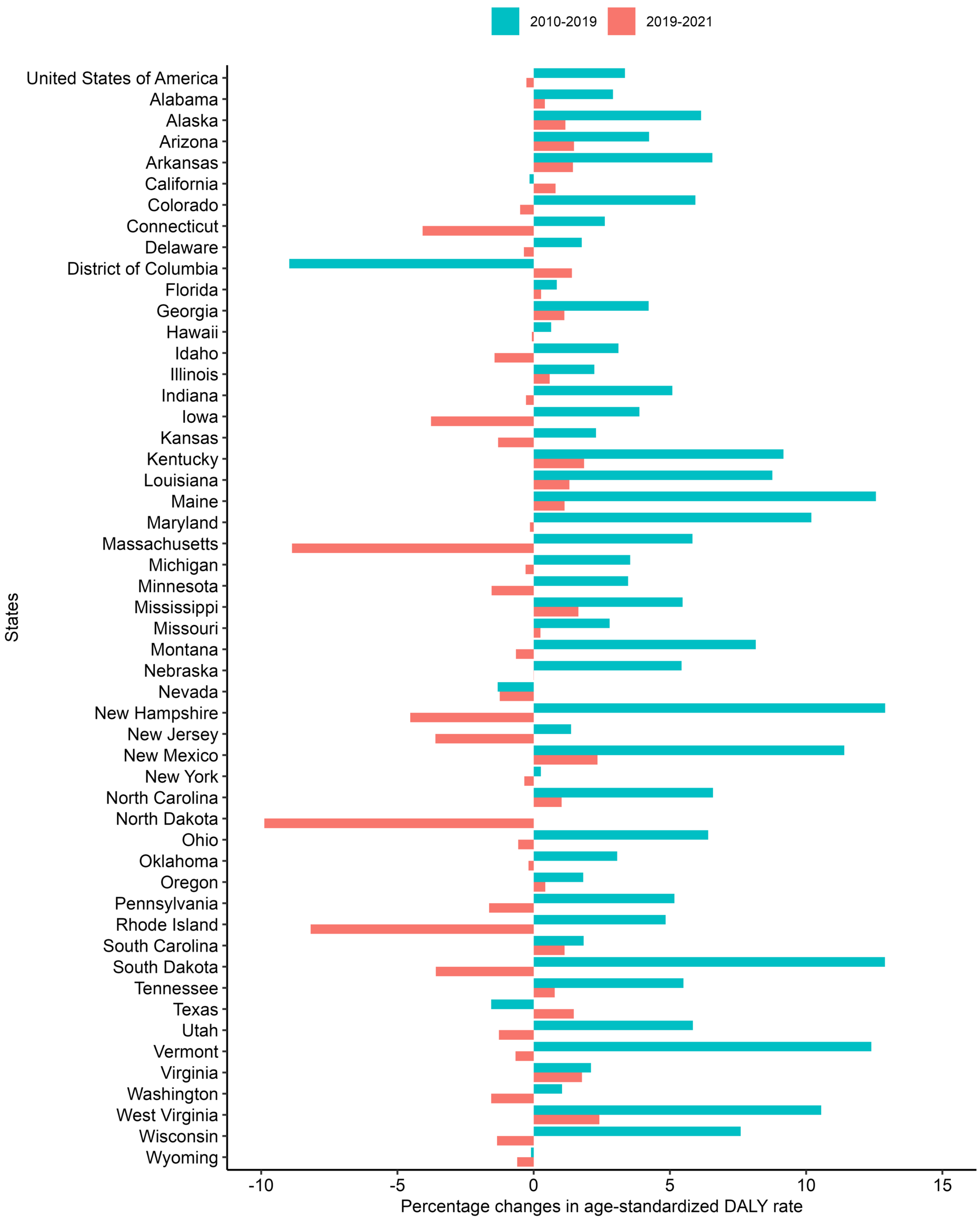

Abbreviations: DALYs, disability-adjusted life years.

(C) deaths

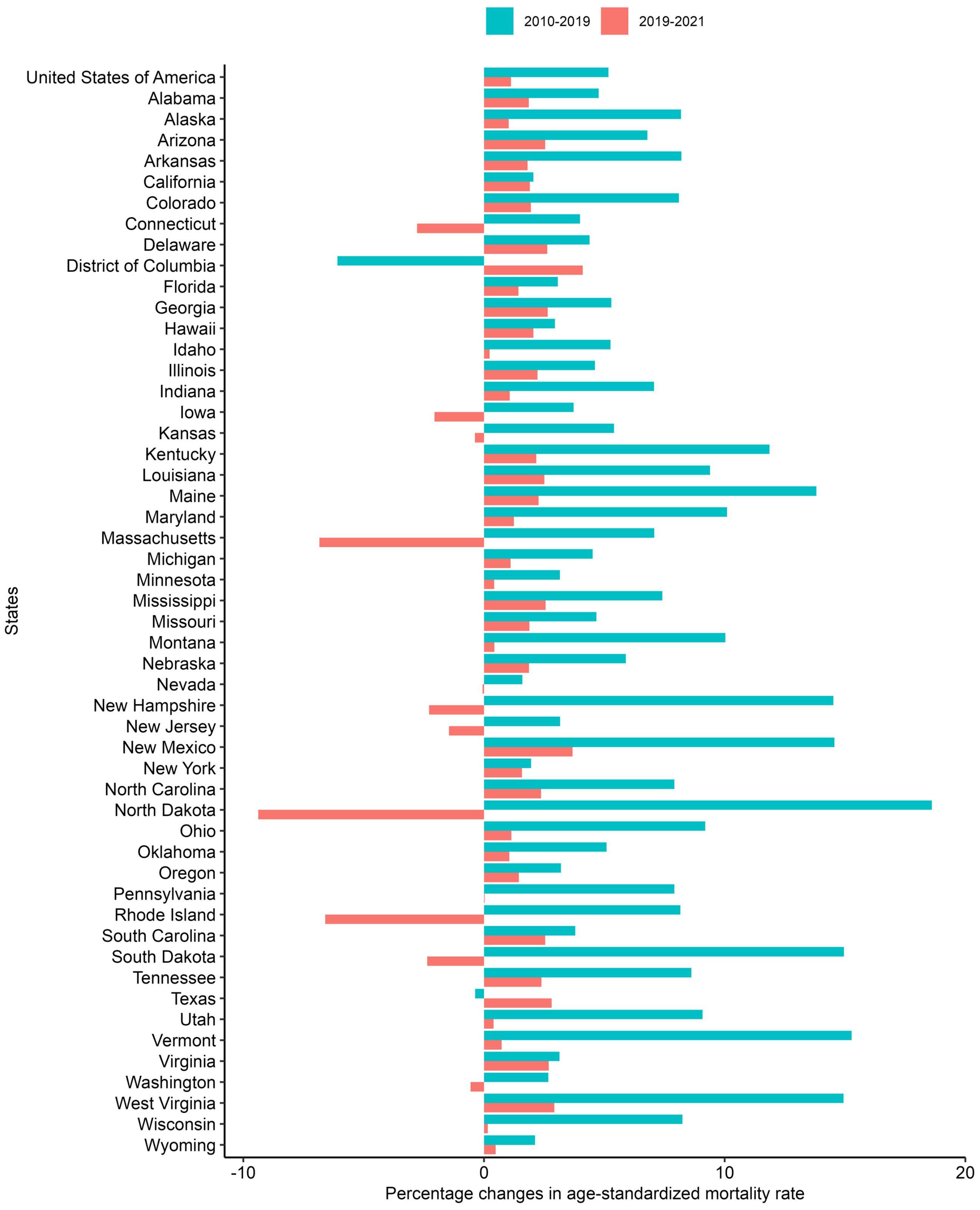

2010-2019
2019-2021
United States of America
Alabama
Alaska
Arizona
Arkansas
California
Colorado
Connecticut
Delaware
District of Columbia
Florida
Georgia
Hawaii
Idaho
Illinois
Indiana
Iowa
Kansas
Kentucky
Louisiana
Maine
Maryland
Massachusetts
Michigan
Minnesota
Mississippi
Missouri
Montana
Nebraska
Nevada
New Hampshire
New Jersey
New Mexico
New York
North Carolina
North Dakota
Ohio
Oklahoma
Oregon
Pennsylvania
Rhode Island
South Carolina
South Dakota
Tennessee
Texas
Utah
Vermont
Virginia
Washington
West Virginia
Wisconsin
Wyoming
States
-10
0
10
20
Percentage changes in age-standardized mortality rate

(D) incidence

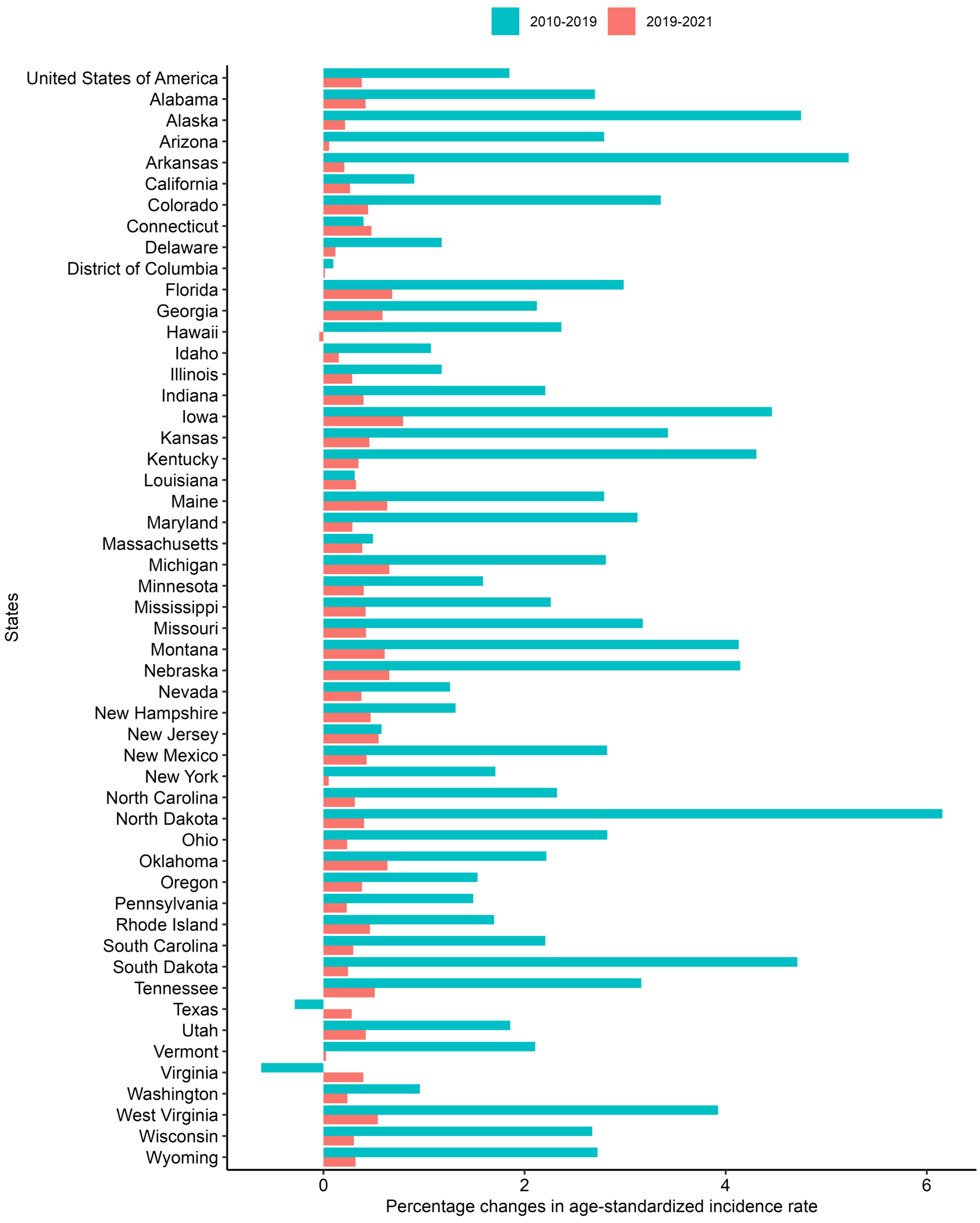

2010-2019
2019-2021
States
United States of America
Alabama
Alaska
Arizona
Arkansas
California
Colorado
Connecticut
Delaware
District of Columbia
Florida
Georgia
Hawaii
Idaho
Illinois
Indiana
Iowa
Kansas
Kentucky
Louisiana
Maine
Maryland
Massachusetts
Michigan
Minnesota
Mississippi
Missouri
Montana
Nebraska
Nevada
New Hampshire
New Jersey
New Mexico
New York
North Carolina
North Dakota
Ohio
Oklahoma
Oregon
Pennsylvania
Rhode Island
South Carolina
South Dakota
Tennessee
Texas
Utah
Vermont
Virginia
Washington
West Virginia
Wisconsin
Wyoming
0
2
4
6
Percentage changes in age-standardized incidence rate

(E) YLDs

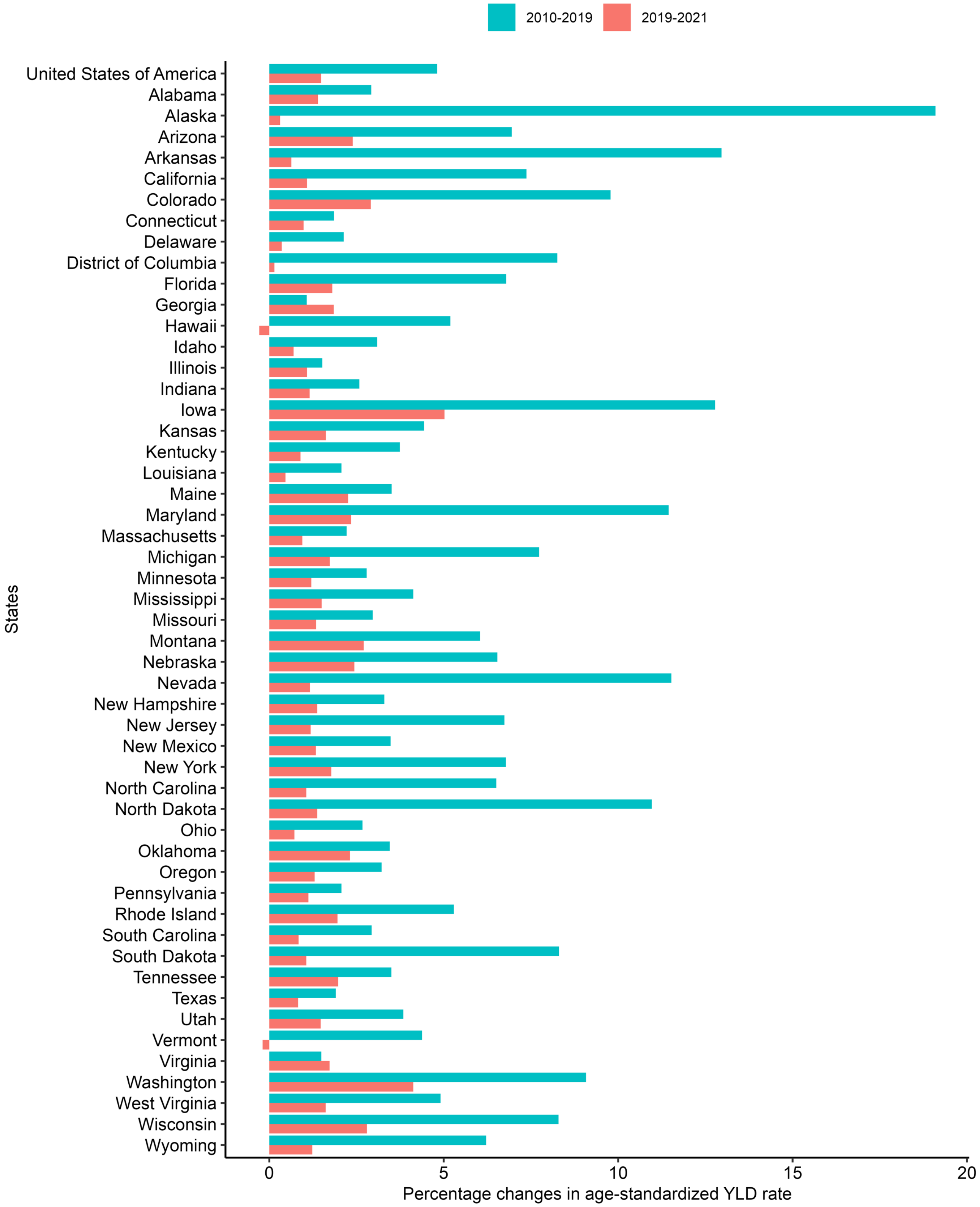

Abbreviations: YLDs, years lived with disability.

(F) YLLs

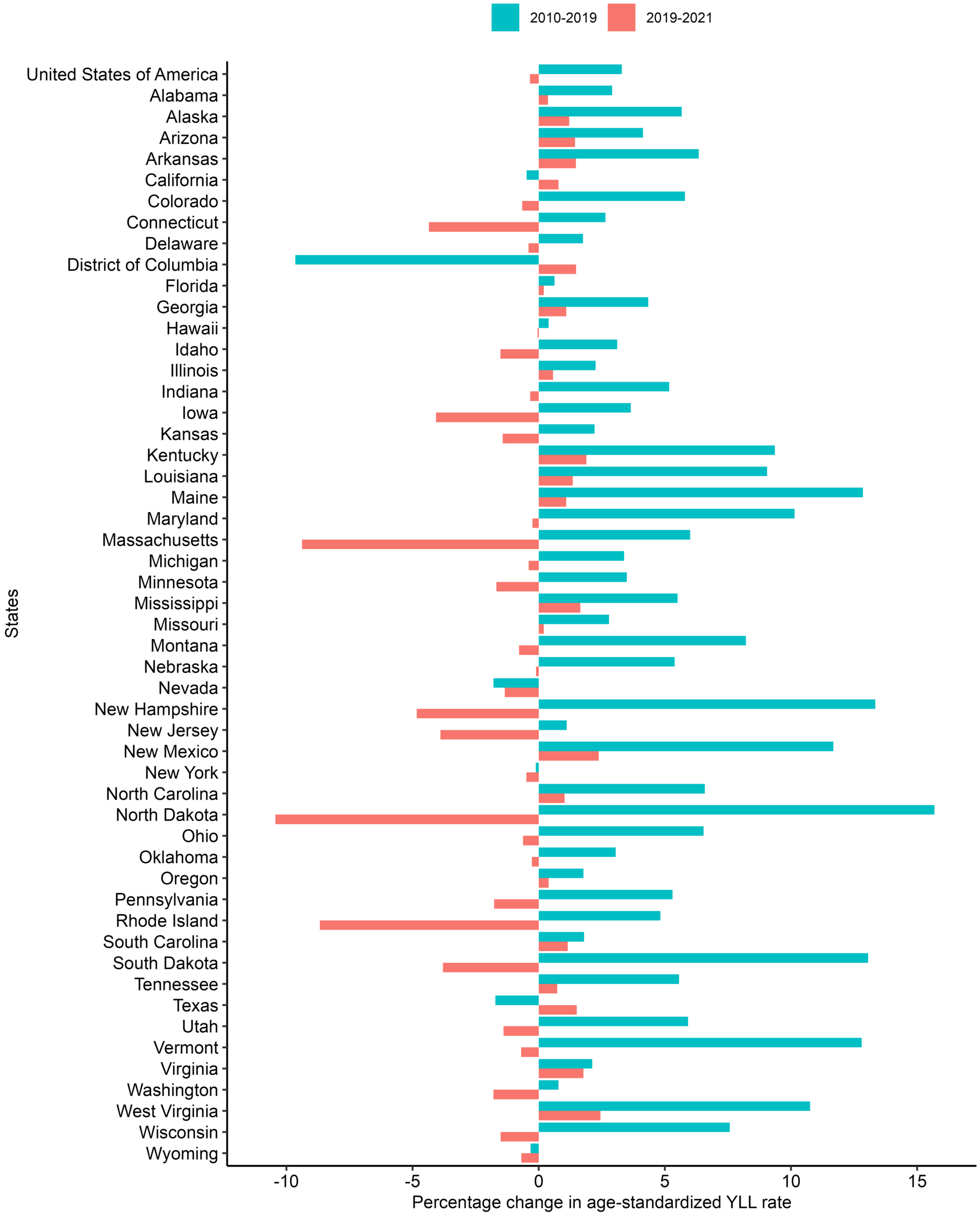

Abbreviations: YLLs, years of life lost.

Figure S6: Changes in rates of (A) prevalence, (B) DALYs, (C) deaths, (D) incidence, (E) YLDs, (F) YLLs in pre-pandemic (2010-2019) and pandemic (2019-2021) periods, by age groups, sex and divisions.

(A) prevalence

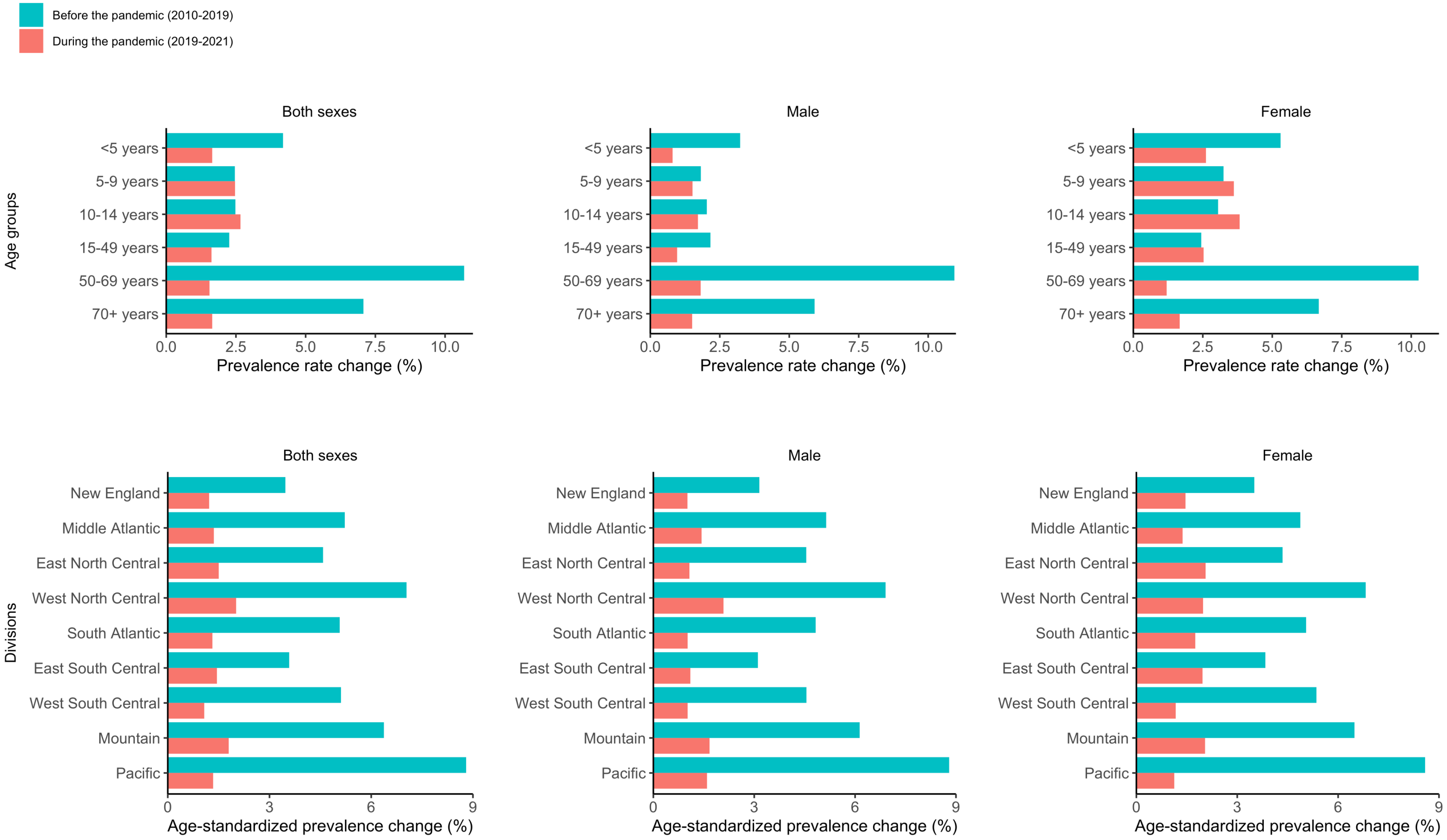

Abbreviations: DALYs, disability-adjusted life years; YLDs, years lived with disability; YLLs, years of life lost.

(B) DALYs

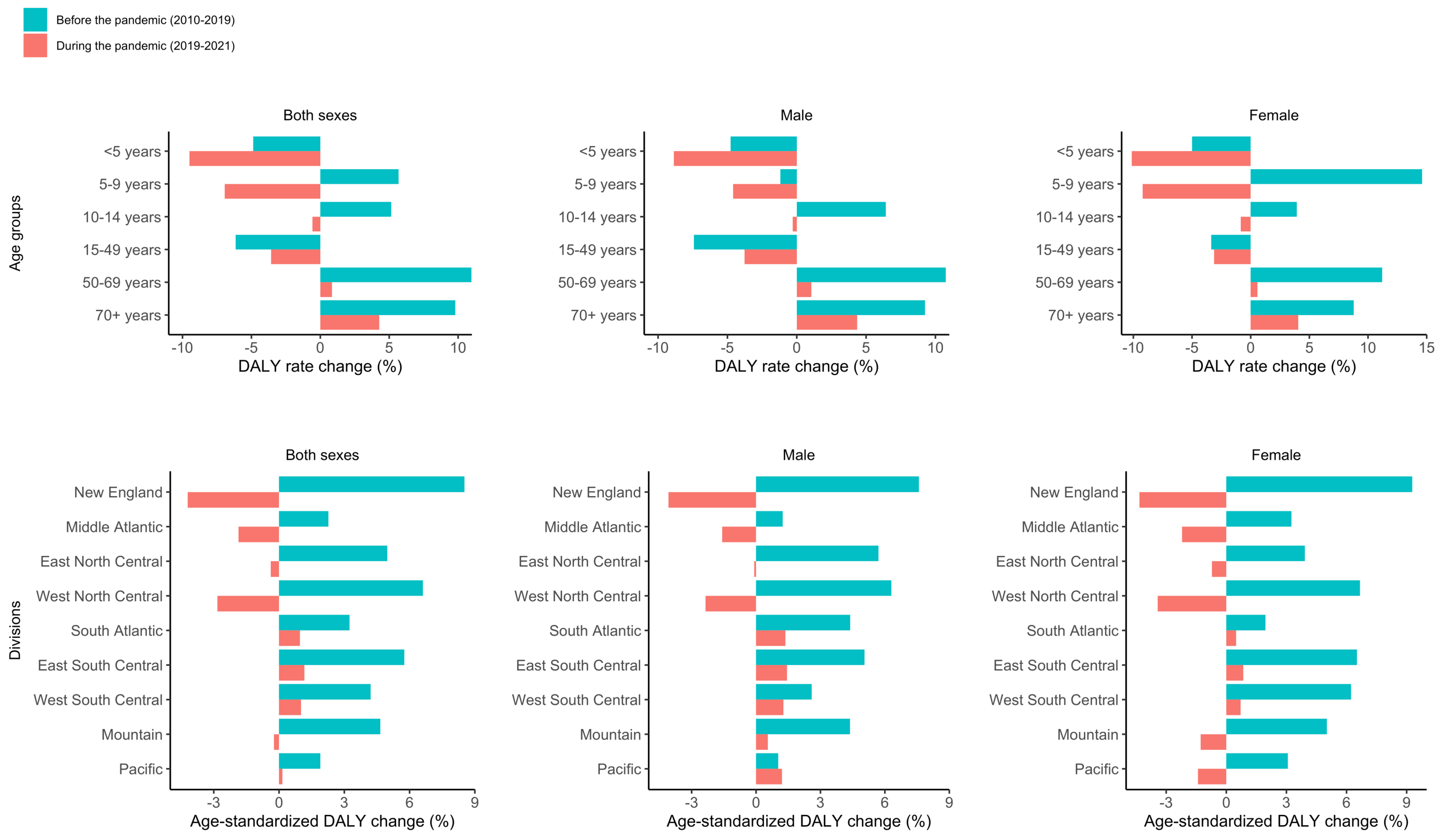

Abbreviations: DALYs, disability-adjusted life years.

(C) deaths

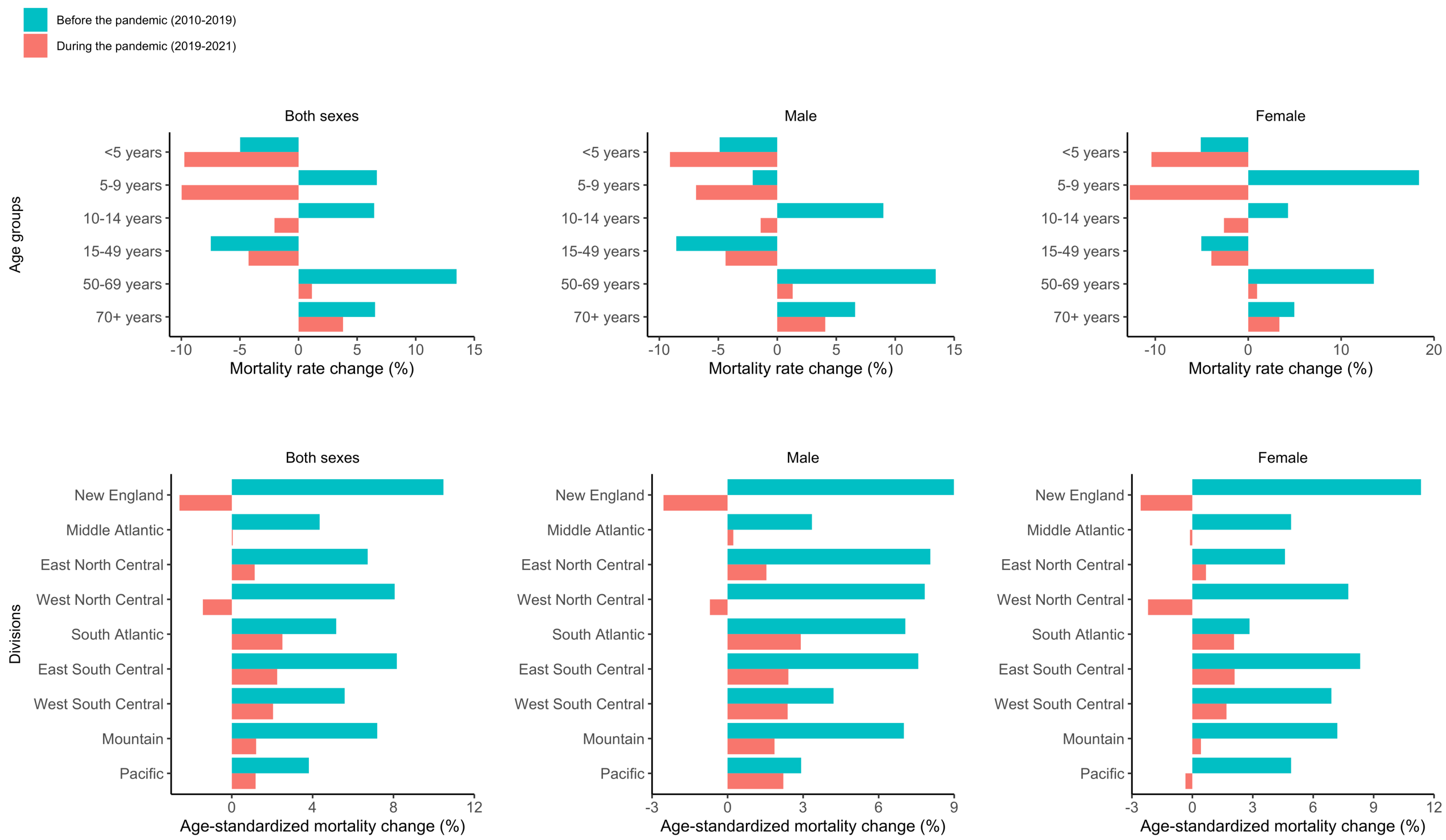

Before the pandemic (2010-2019)
During the pandemic (2019-2021)
Both sexes
Male
Female
Age groups
<5 years
5-9 years
10-14 years
15-49 years
50-69 years
70+ years
Mortality rate change (%)
Divisions
New England
Middle Atlantic
East North Central
West North Central
South Atlantic
East South Central
West South Central
Mountain
Pacific
Age-standardized mortality change (%)

(D) incidence

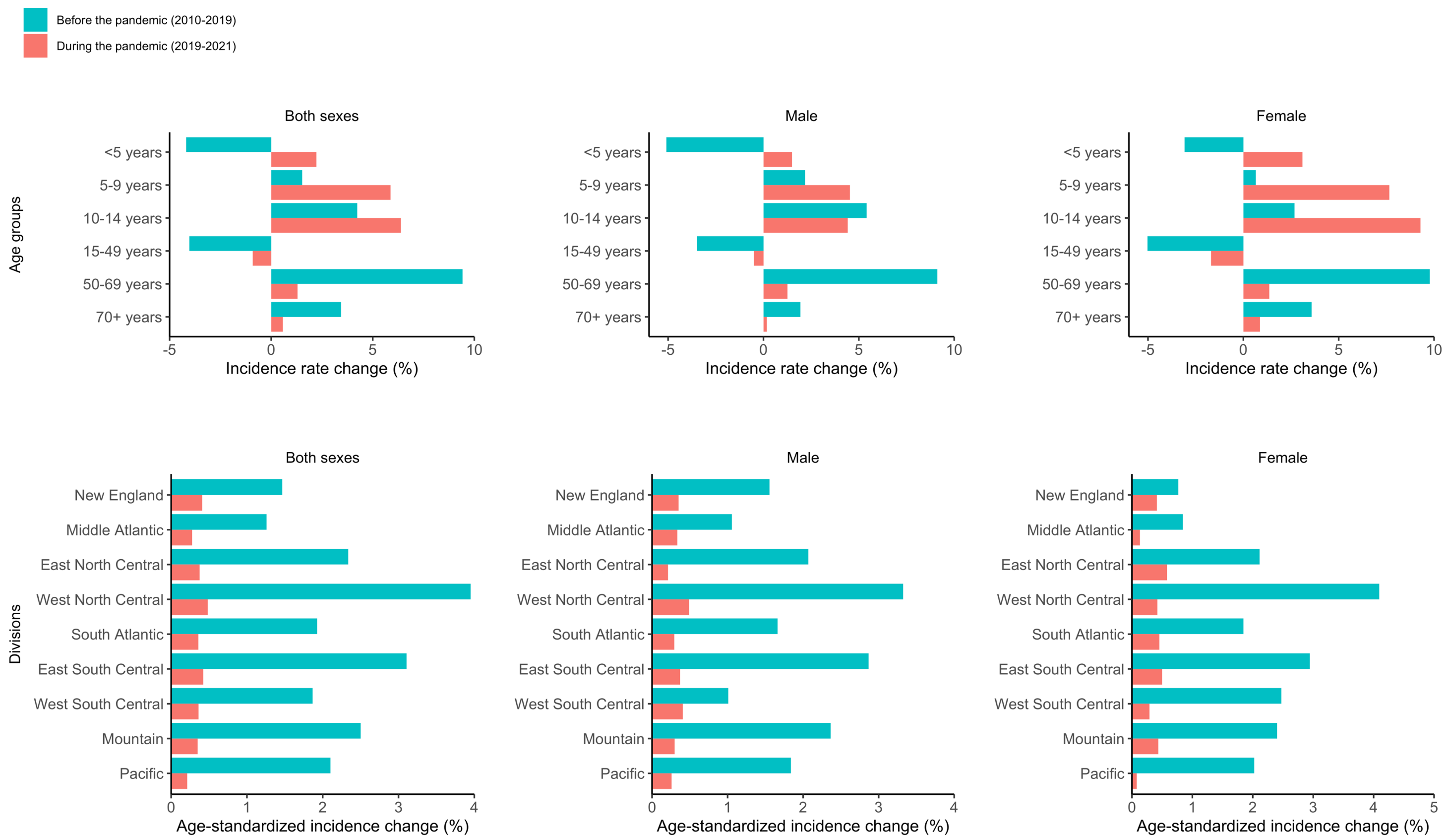
Before the pandemic (2010-2019)
During the pandemic (2019-2021)
Both sexes
Male
Female
Age groups
<5 years
5-9 years
10-14 years
15-49 years
50-69 years
70+ years
-5
0
5
10
Incidence rate change (%)
Divisions
New England
Middle Atlantic
East North Central
West North Central
South Atlantic
East South Central
West South Central
Mountain
Pacific
0
1
2
3
4
5
Age-standardized incidence change (%)

(E) YLDs

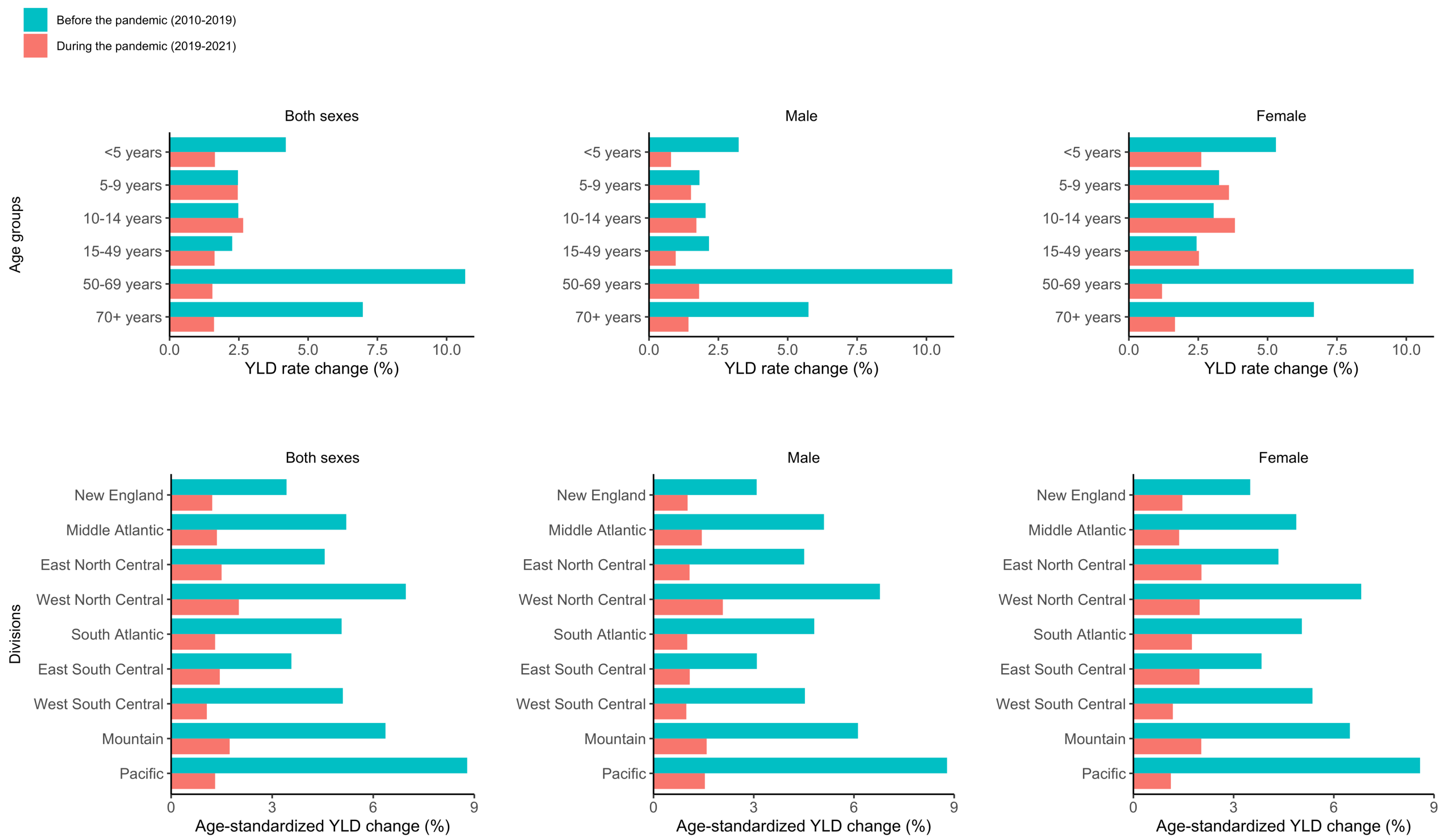

Abbreviations: YLDs, years lived with disability.

(F) YLLs

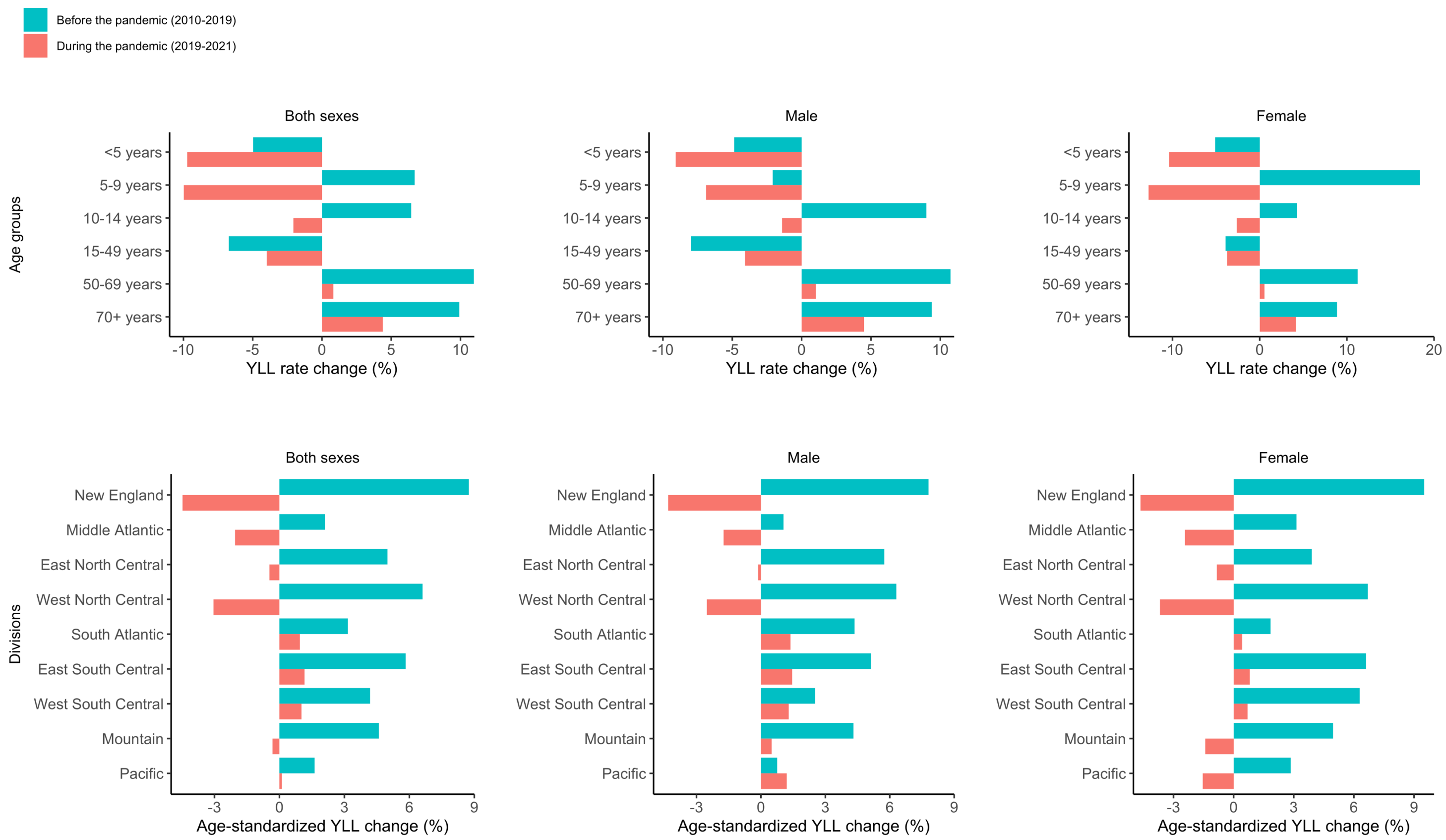

Abbreviations: YLLs, years of life lost.

Figure S7: Annual trends of age-standardized rate and numbers of (A) prevalence, DALYs, and deaths, (B) DALYs, YLDs, and YLLs from 1990 to 2021, by sex (shaded areas represent 95% uncertainty intervals).

(A) prevalence, DALYs, and deaths

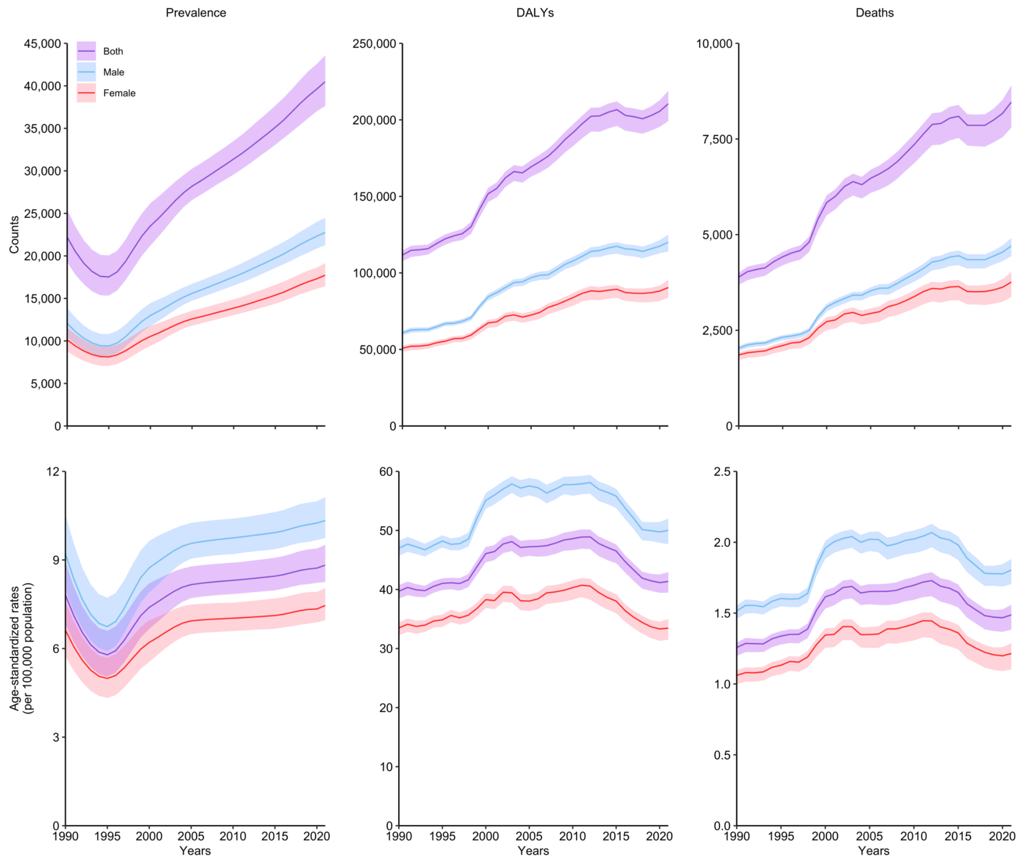

Abbreviations: DALYs, disability-adjusted life years; YLDs, years lived with disability; YLLs, years of life lost.

(B) DALYs, YLDs, and YLLs

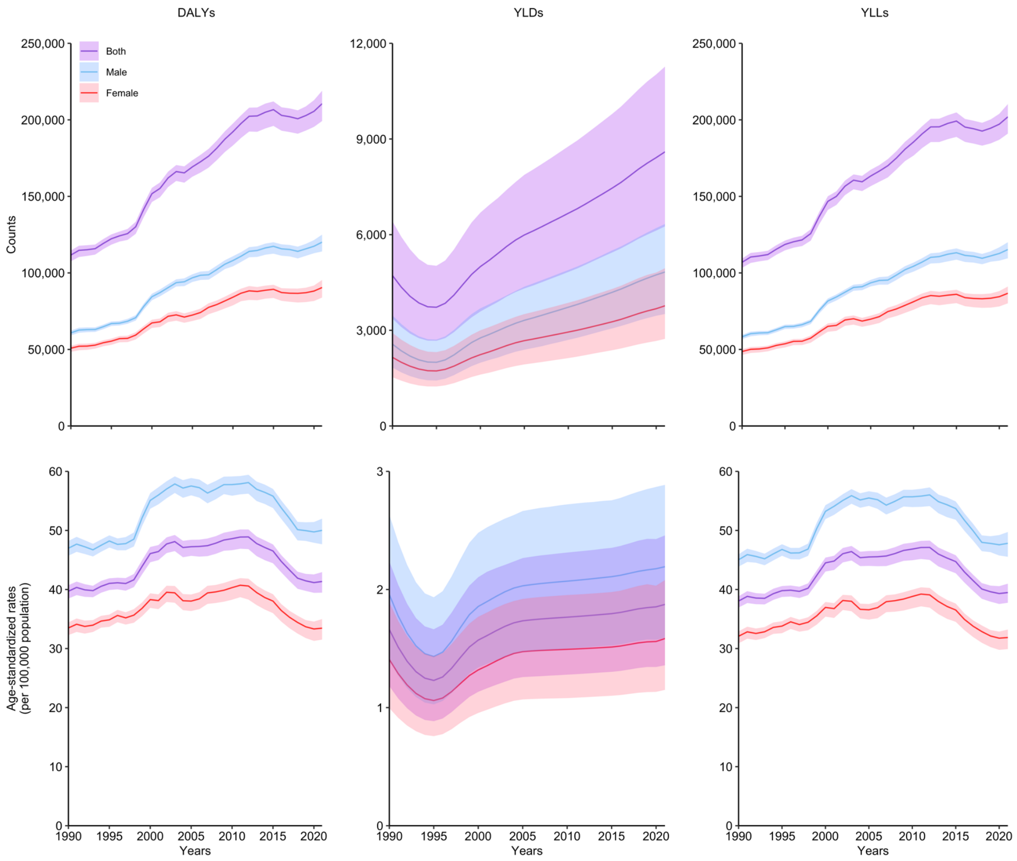

Abbreviations: DALYs, disability-adjusted life years; YLDs, years lived with disability; YLLs, years of life lost.

Figure S8: Rates of (A) prevalence, (B) DALYs, (C) deaths, (D) incidence, (E) YLDs, (F) YLLs in 2021, by age groups and divisions.

(A) prevalence

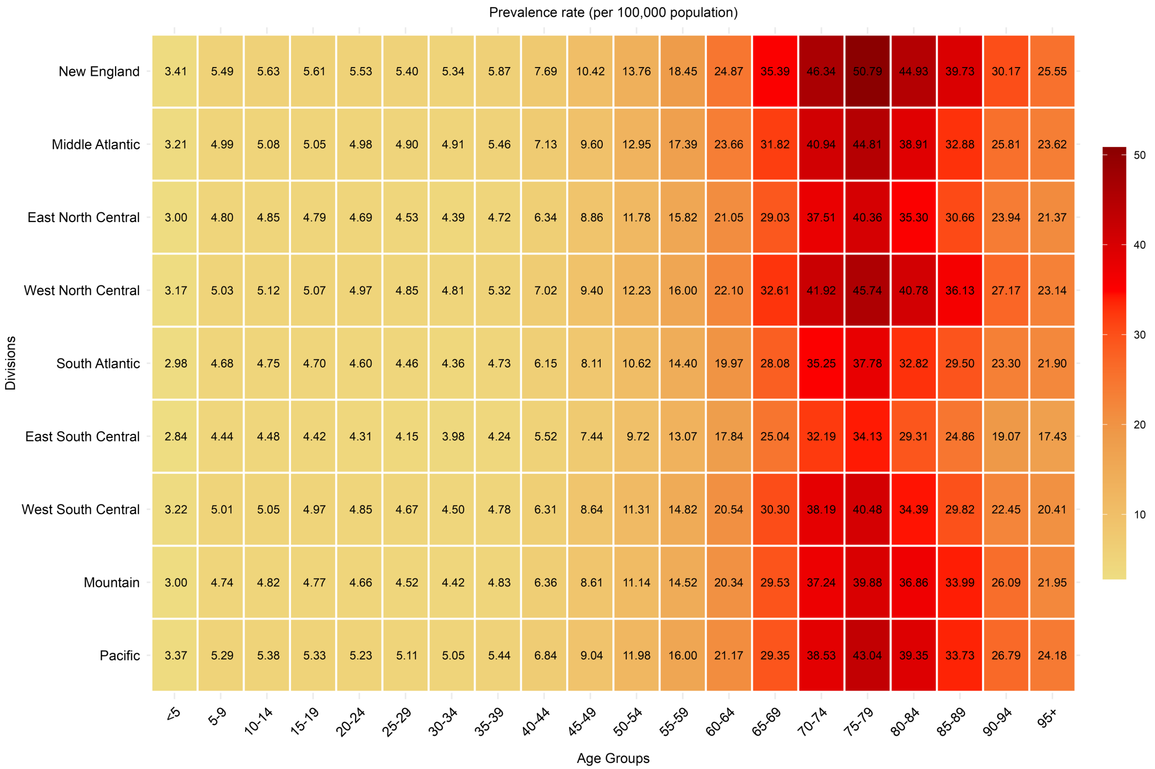

Abbreviations: DALYs, disability-adjusted life years; YLDs, years lived with disability; YLLs, years of life lost.

(B) DALYs

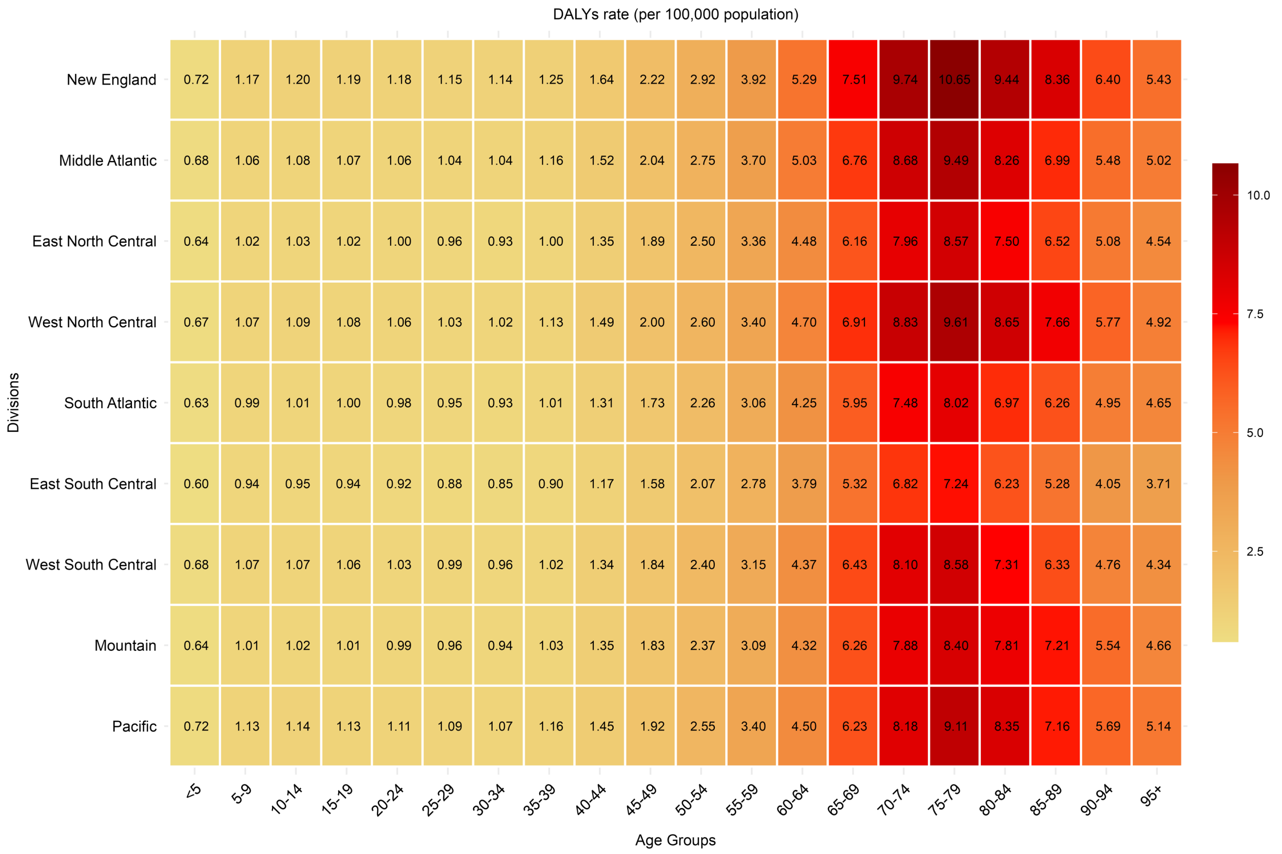

Abbreviations: DALYs, disability-adjusted life years.

(C) deaths

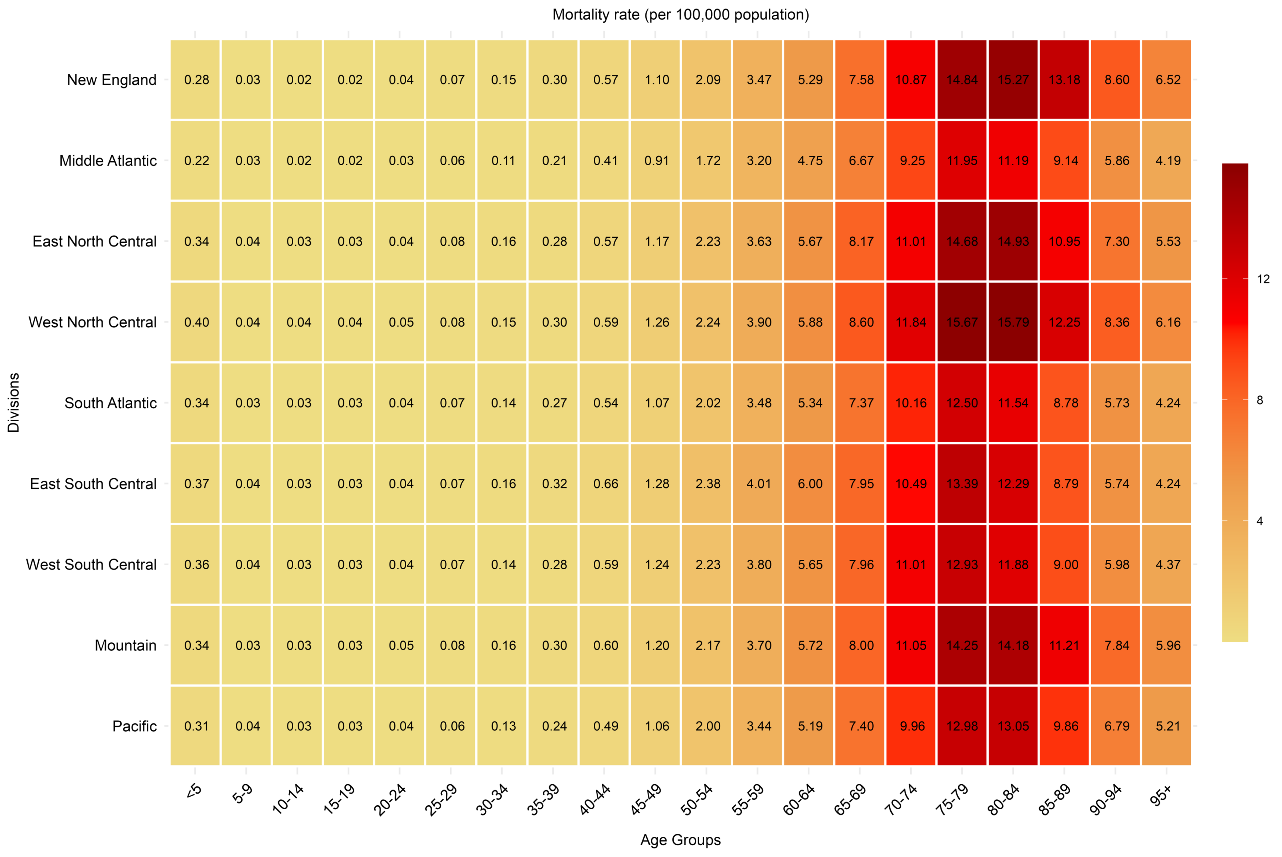
Mortality rate (per 100,000 population)
Divisions
New England
Middle Atlantic
East North Central
West North Central
South Atlantic
East South Central
West South Central
Mountain
Pacific
0.28 0.03 0.02 0.02 0.04 0.07 0.15 0.30 0.57 1.10 2.09 3.47 5.29 7.58 10.87 14.84 15.27 13.18 8.60 6.52
0.22 0.03 0.02 0.02 0.03 0.06 0.11 0.21 0.41 0.91 1.72 3.20 4.75 6.67 9.25 11.95 11.19 9.14 5.86 4.19
0.34 0.04 0.03 0.03 0.04 0.08 0.16 0.28 0.57 1.17 2.23 3.63 5.67 8.17 11.01 14.68 14.93 10.95 7.30 5.53
0.40 0.04 0.04 0.04 0.05 0.08 0.15 0.30 0.59 1.26 2.24 3.90 5.88 8.60 11.84 15.67 15.79 12.25 8.36 6.16
0.34 0.03 0.03 0.03 0.04 0.07 0.14 0.27 0.54 1.07 2.02 3.48 5.34 7.37 10.16 12.50 11.54 8.78 5.73 4.24
0.37 0.04 0.03 0.03 0.04 0.07 0.16 0.32 0.66 1.28 2.38 4.01 6.00 7.95 10.49 13.39 12.29 8.79 5.74 4.24
0.36 0.04 0.03 0.03 0.04 0.07 0.14 0.28 0.59 1.24 2.23 3.80 5.65 7.96 11.01 12.93 11.88 9.00 5.98 4.37
0.34 0.03 0.03 0.03 0.05 0.08 0.16 0.30 0.60 1.20 2.17 3.70 5.72 8.00 11.05 14.25 14.18 11.21 7.84 5.96
0.31 0.04 0.03 0.03 0.04 0.06 0.13 0.24 0.49 1.06 2.00 3.44 5.19 7.40 9.96 12.98 13.05 9.86 6.79 5.21
<5 5-9 10-14 15-19 20-24 25-29 30-34 35-39 40-44 45-49 50-54 55-59 60-64 65-69 70-74 75-79 80-84 85-89 90-94 95+
Age Groups
12
8
4

(D) incidence

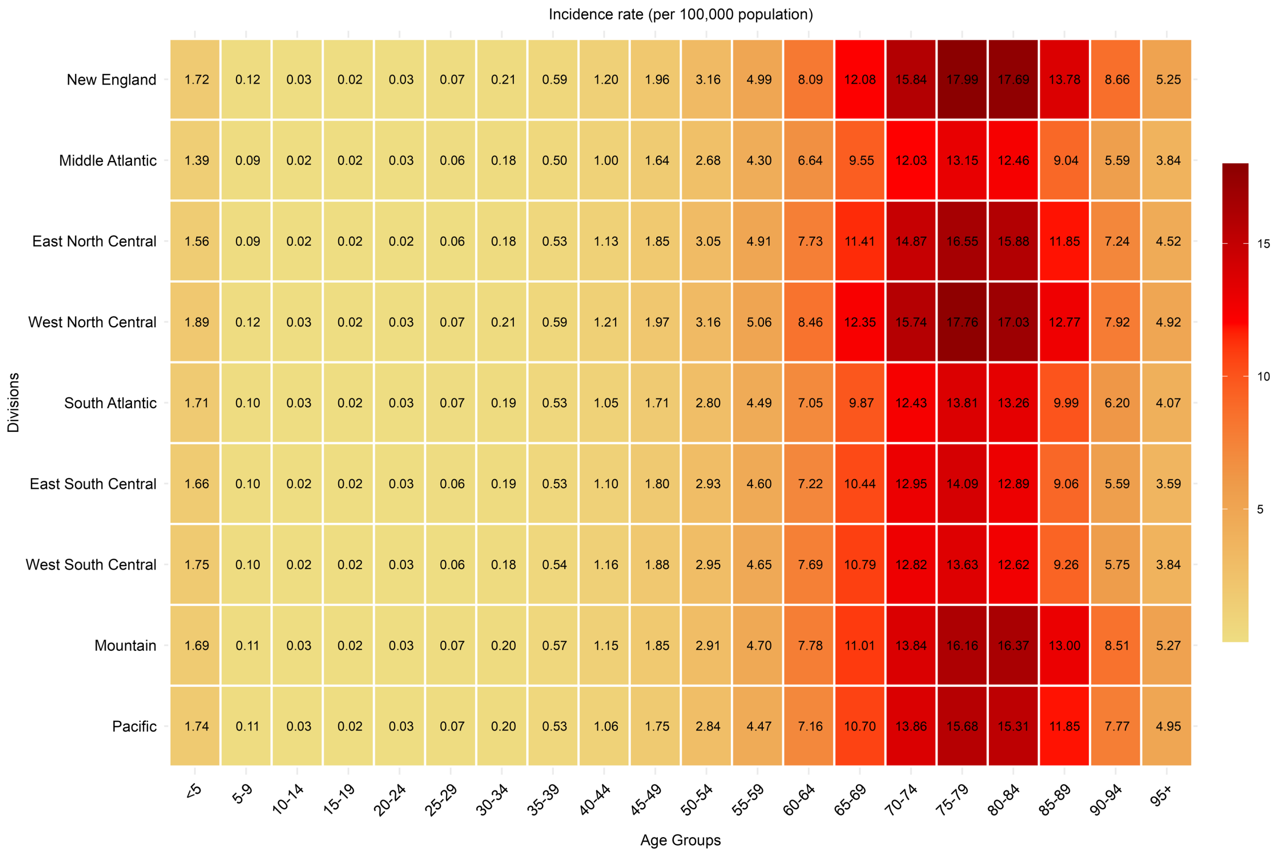
Incidence rate (per 100,000 population)
Divisions
New England 1.72 0.12 0.03 0.02 0.03 0.07 0.21 0.59 1.20 1.96 3.16 4.99 8.09 12.08 15.84 17.99 17.69 13.78 8.66 5.25
Middle Atlantic 1.39 0.09 0.02 0.02 0.03 0.06 0.18 0.50 1.00 1.64 2.68 4.30 6.64 9.55 12.03 13.15 12.46 9.04 5.59 3.84
East North Central 1.56 0.09 0.02 0.02 0.02 0.06 0.18 0.53 1.13 1.85 3.05 4.91 7.73 11.41 14.87 16.55 15.88 11.85 7.24 4.52
West North Central 1.89 0.12 0.03 0.02 0.03 0.07 0.21 0.59 1.21 1.97 3.16 5.06 8.46 12.35 15.74 17.76 17.03 12.77 7.92 4.92
South Atlantic 1.71 0.10 0.03 0.02 0.03 0.07 0.19 0.53 1.05 1.71 2.80 4.49 7.05 9.87 12.43 13.81 13.26 9.99 6.20 4.07
East South Central 1.66 0.10 0.02 0.02 0.03 0.06 0.19 0.53 1.10 1.80 2.93 4.60 7.22 10.44 12.95 14.09 12.89 9.06 5.59 3.59
West South Central 1.75 0.10 0.02 0.02 0.03 0.06 0.18 0.54 1.16 1.88 2.95 4.65 7.69 10.79 12.82 13.63 12.62 9.26 5.75 3.84
Mountain 1.69 0.11 0.03 0.02 0.03 0.07 0.20 0.57 1.15 1.85 2.91 4.70 7.78 11.01 13.84 16.16 16.37 13.00 8.51 5.27
Pacific 1.74 0.11 0.03 0.02 0.03 0.07 0.20 0.53 1.06 1.75 2.84 4.47 7.16 10.70 13.86 15.68 15.31 11.85 7.77 4.95
<5 5-9 10-14 15-19 20-24 25-29 30-34 35-39 40-44 45-49 50-54 55-59 60-64 65-69 70-74 75-79 80-84 85-89 90-94 95+
Age Groups
15
10
5

(E) YLDs

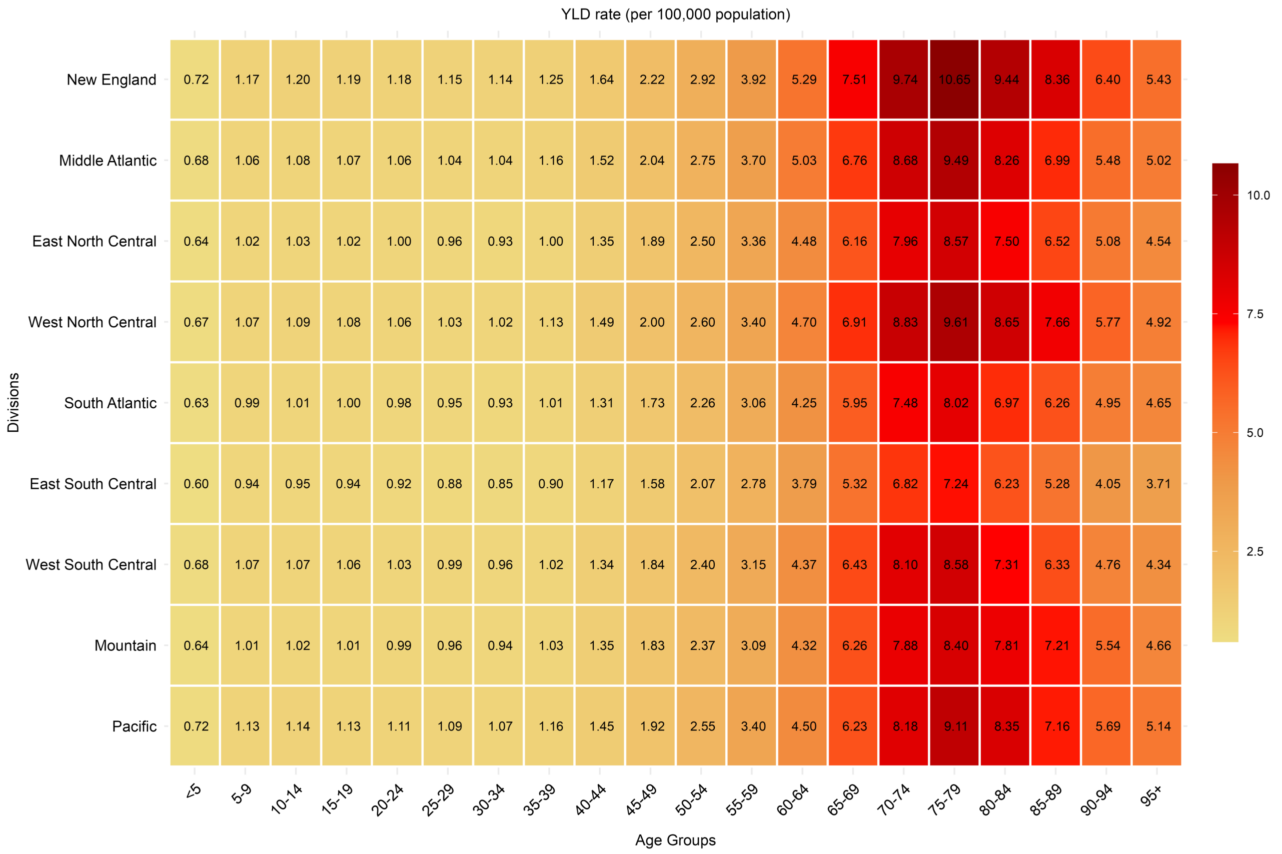

Abbreviations: YLDs, years lived with disability.

(F) YLLs

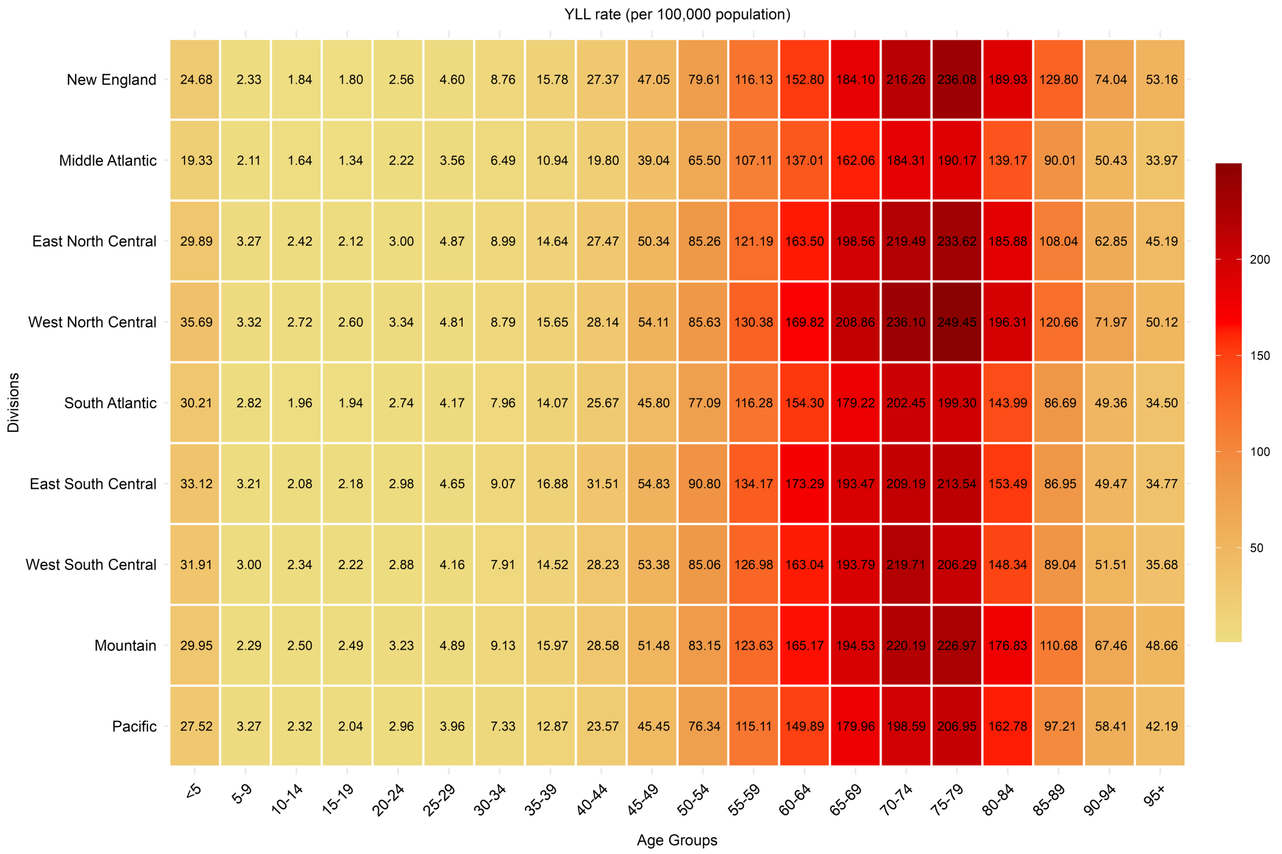

Abbreviations: YLLs, years of life lost.

Figure S9: Numbers and rates (per 100,000 population) of (A) incidence and deaths, (B) YLDs and YLLs according to year, age groups, and sex in the US.

(A) incidence and deaths

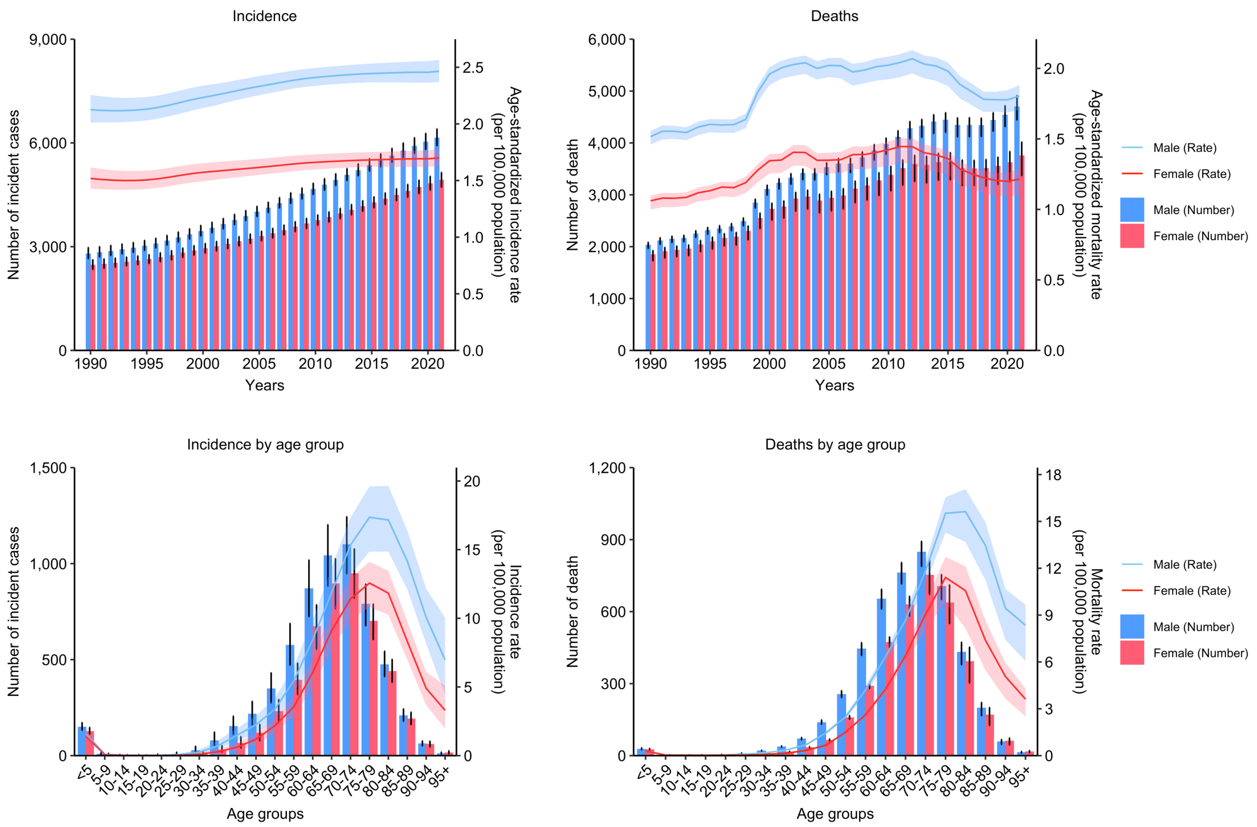

Abbreviations: US, United States; YLDs, years lived with disability; YLLs, years of life lost.

(B) YLDs and YLLs

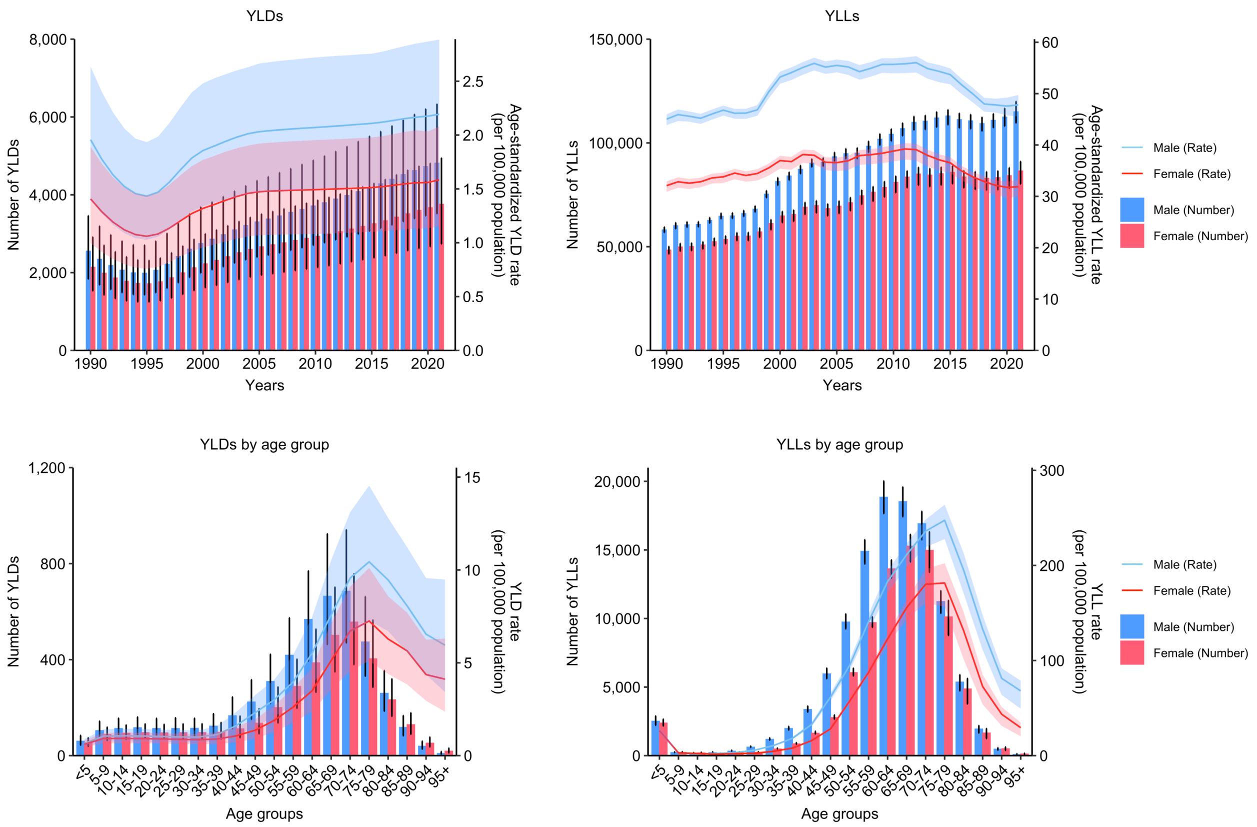

Abbreviations: YLDs, years lived with disability; YLLs, years of life lost.

Figure S10: Annual trends of rates for (A) prevalence, (B) DALYs, (C) deaths, (D) incidence, (E) YLDs, (F) YLLs from 1990 to 2021, stratified by two age groups (younger than 70 years and 70 years and older) (shaded areas are 95% uncertainty intervals).

(A) prevalence

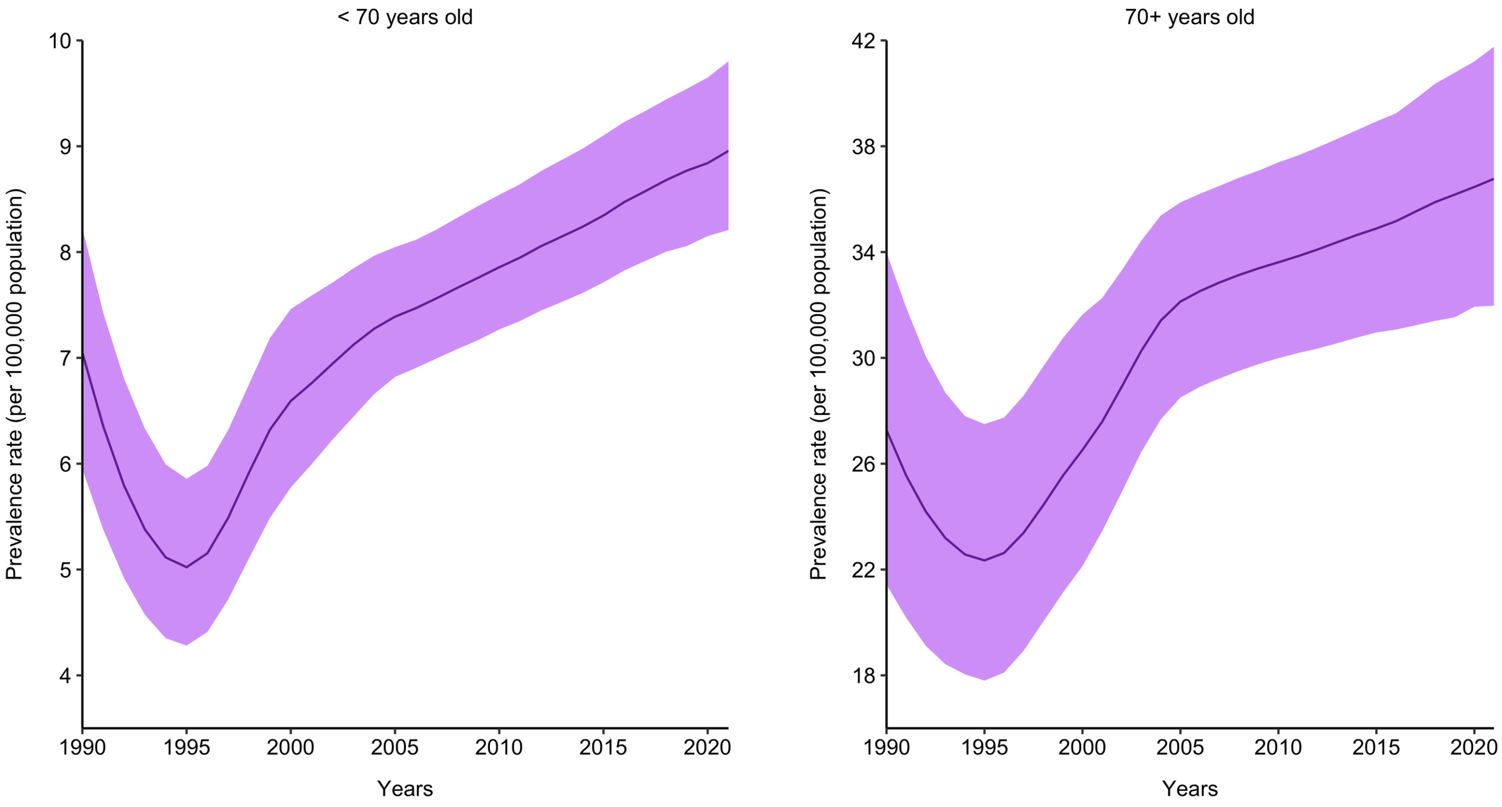

Abbreviations: DALYs, disability-adjusted life years; YLDs, years lived with disability; YLLs, years of life lost.

(B) DALYs

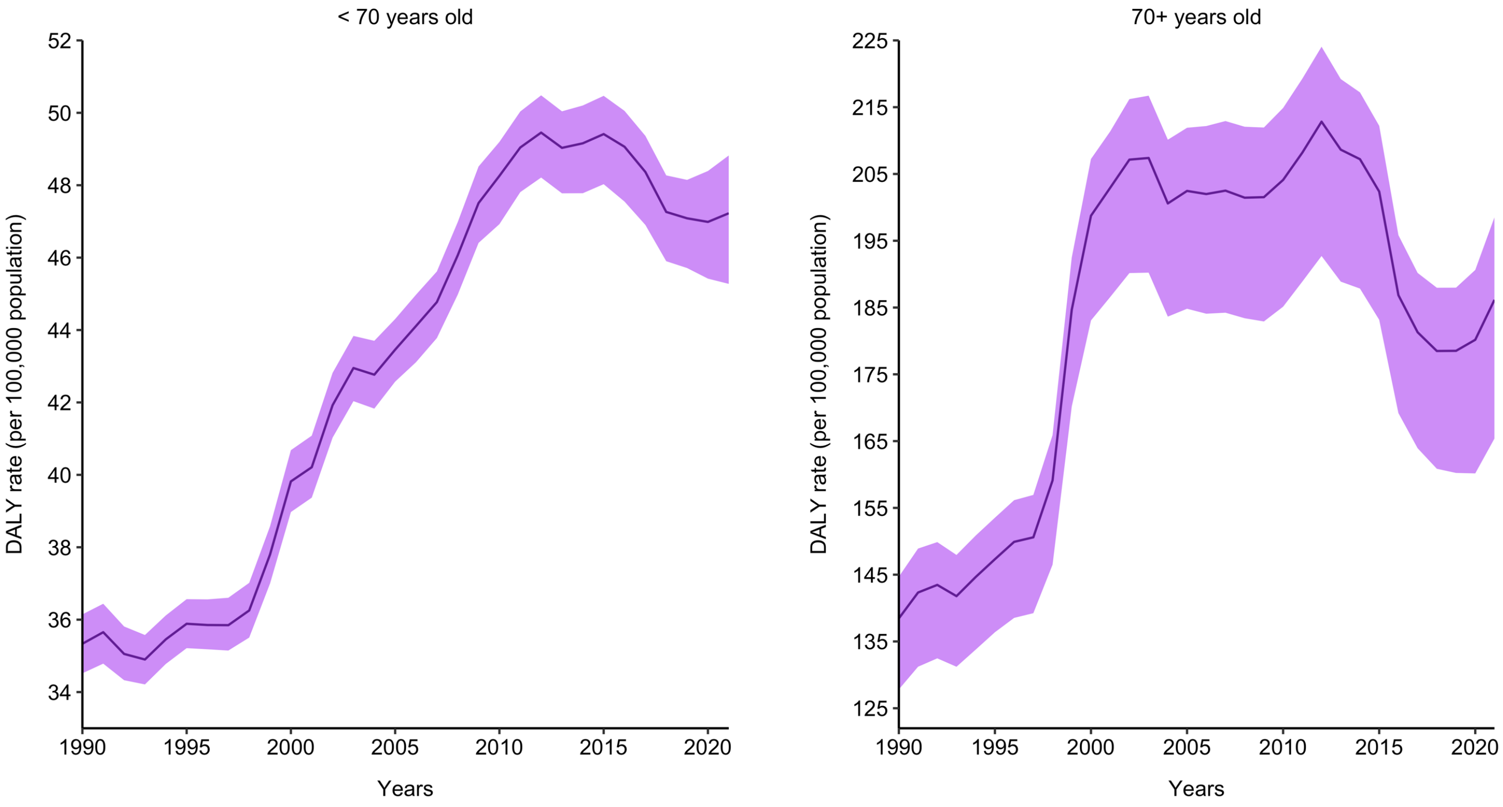

Abbreviations: DALYs, disability-adjusted life years.

(C) deaths

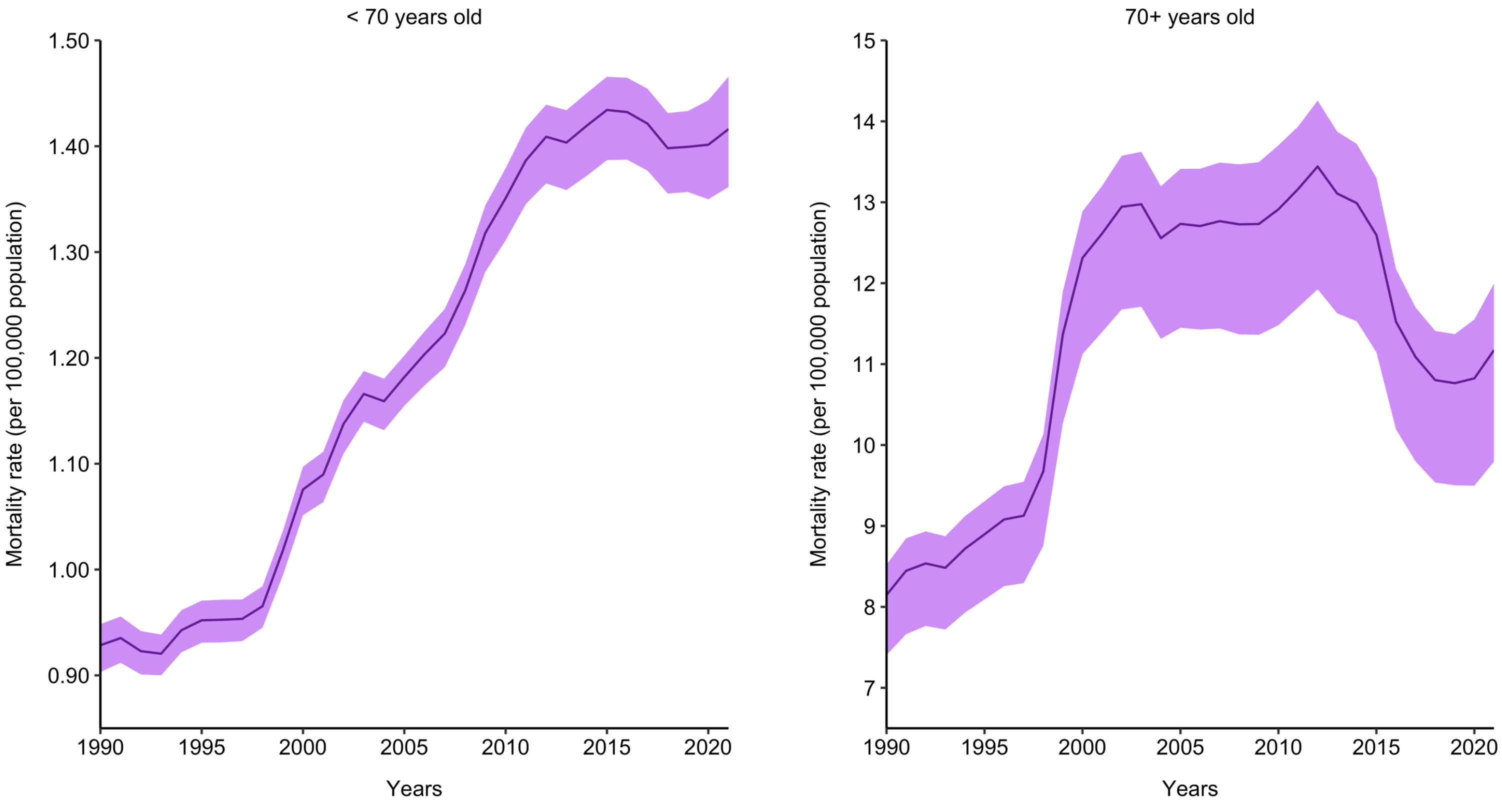

< 70 years old
70+ years old
Mortality rate (per 100,000 population)
1.50
1.40
1.30
1.20
1.10
1.00
0.90
15
14
13
12
11
10
9
8
7
1990
1995
2000
2005
2010
2015
2020
Years

(D) incidence

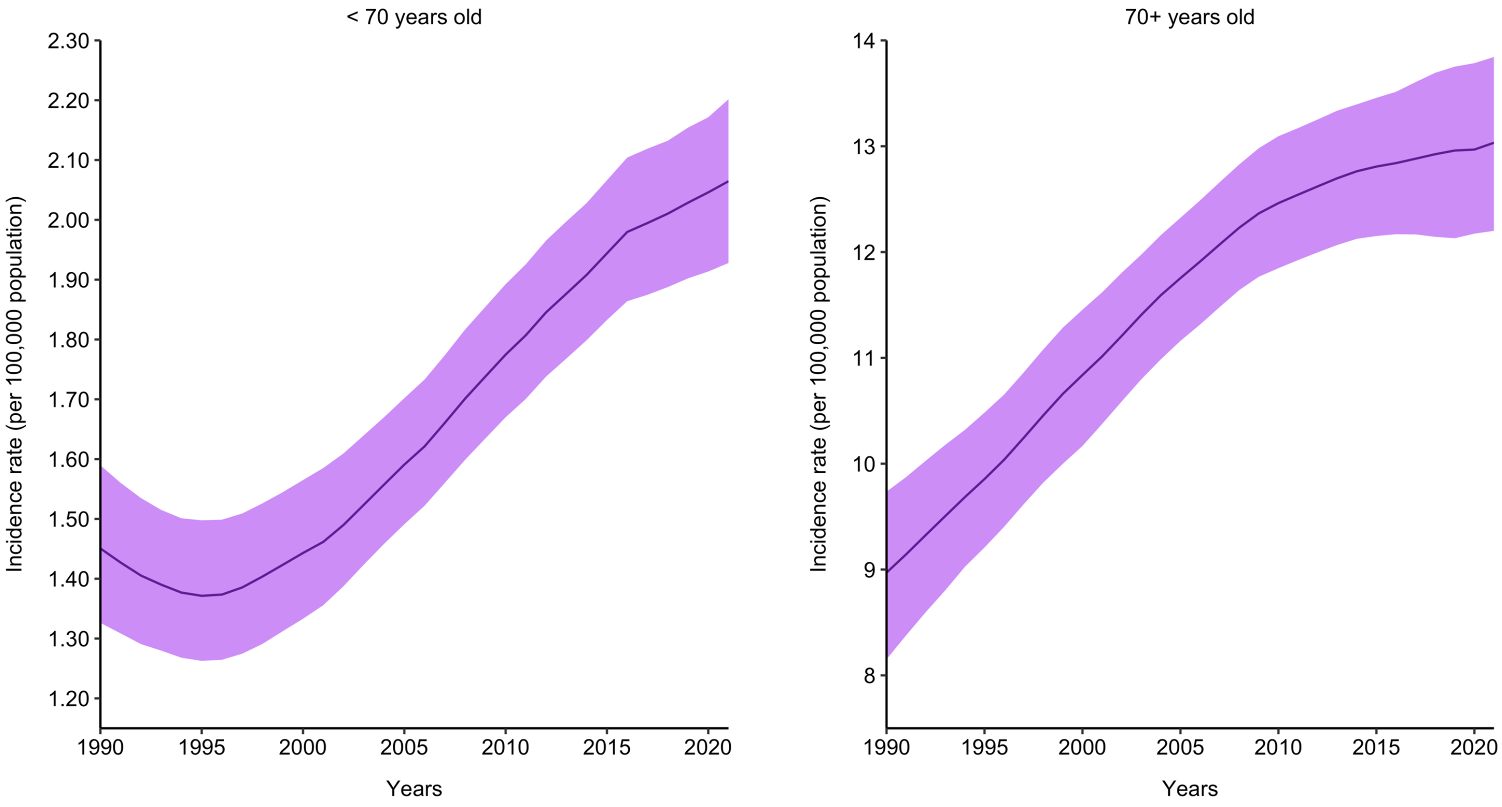

< 70 years old
2.30
2.20
2.10
2.00
1.90
1.80
1.70
1.60
1.50
1.40
1.30
1.20
Incidence rate (per 100,000 population)
1990
1995
2000
2005
2010
2015
2020
Years
70+ years old
14
13
12
11
10
9
8
Incidence rate (per 100,000 population)
1990
1995
2000
2005
2010
2015
2020
Years

(E) YLDs

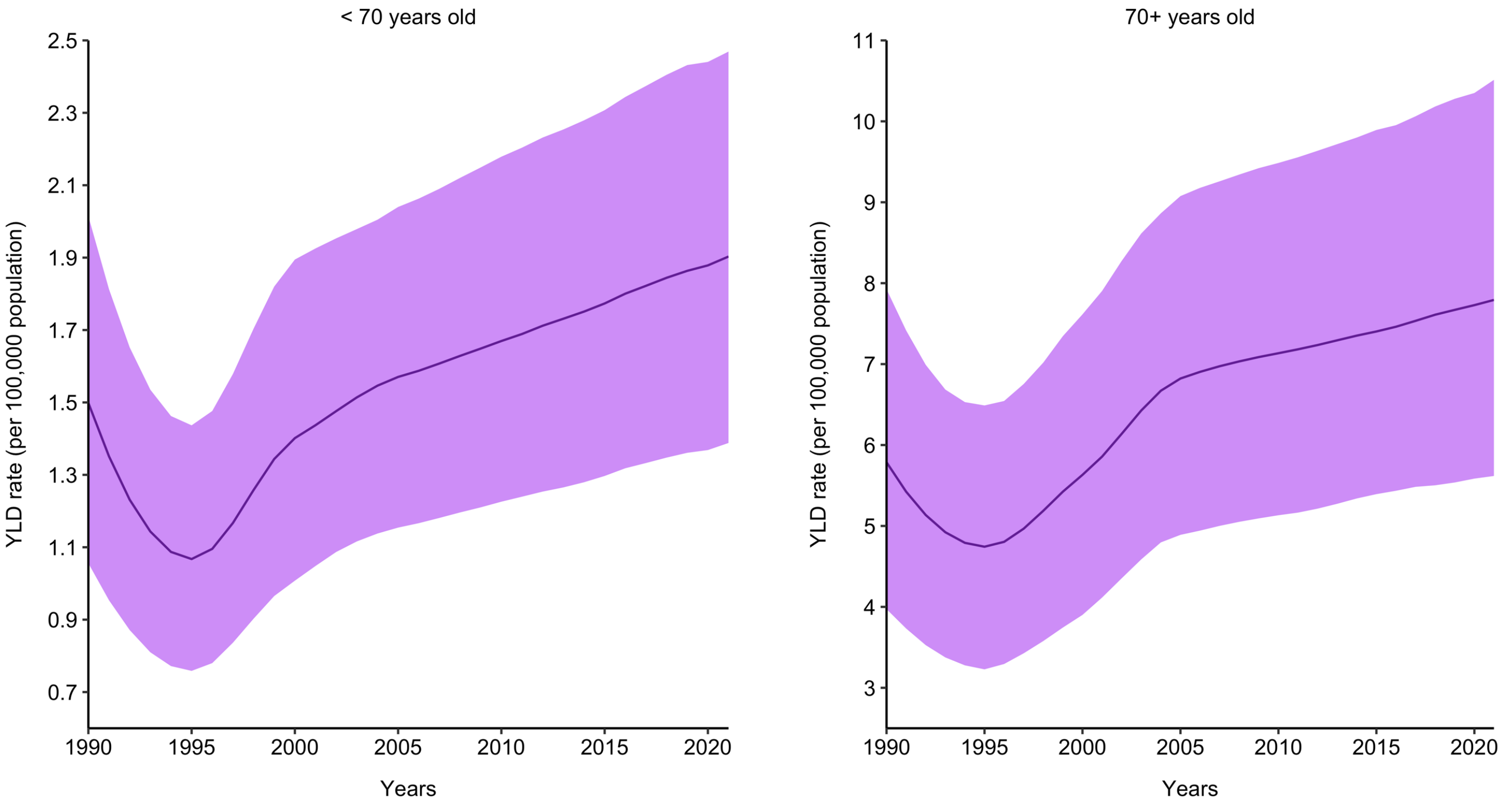

Abbreviations: YLDs, years lived with disability.

(F) YLLs

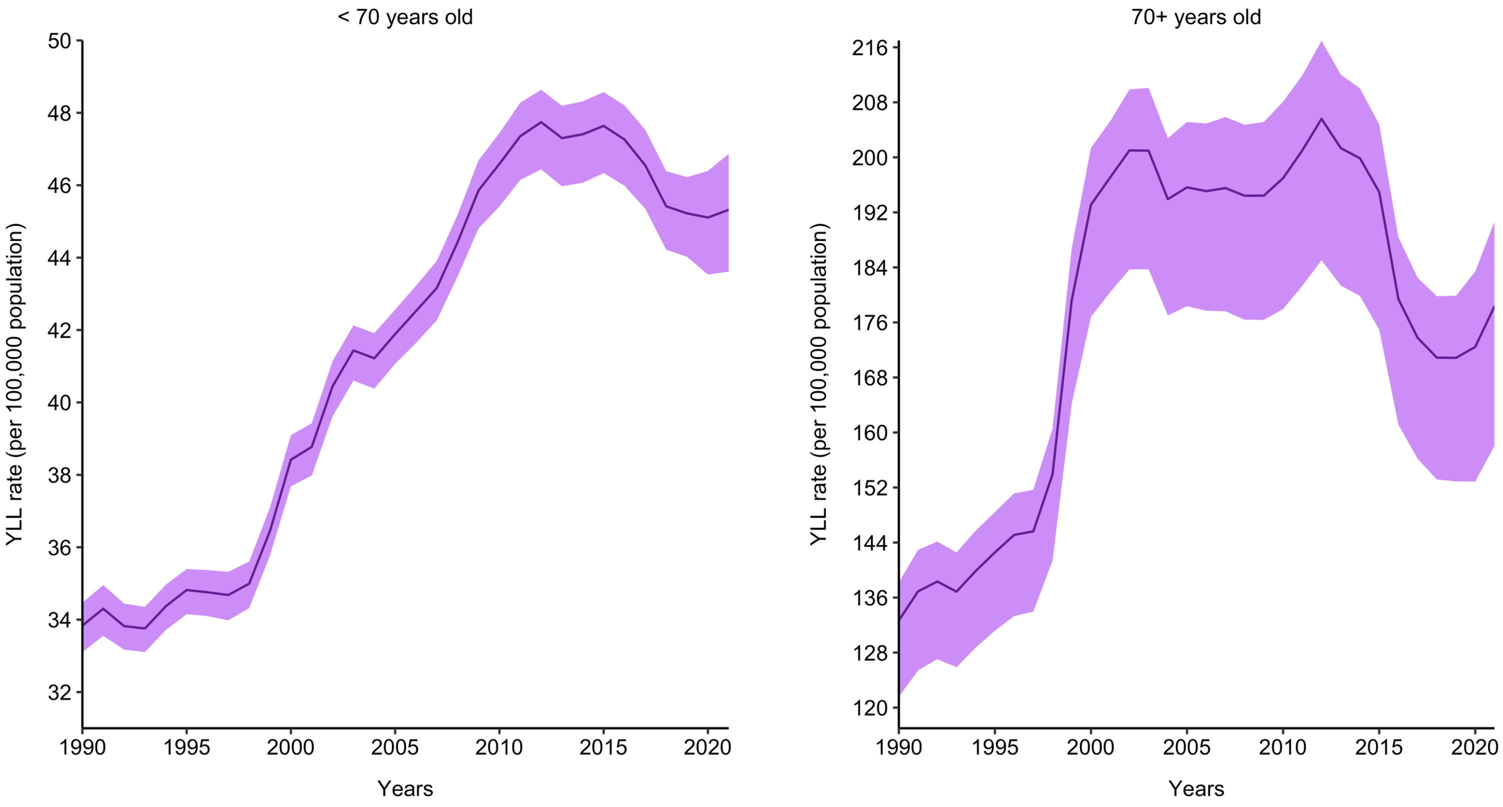

Abbreviations: YLLs, years of life lost.

Figure S11: Age-standardized (A) prevalence, (B) DALY, (C) death, (D) incidence, (E) YLD, (F) YLL rates for motor neuron disease from 1990 to 2021 in nine divisions of the US according to the sociodemographic index.

(A) prevalence

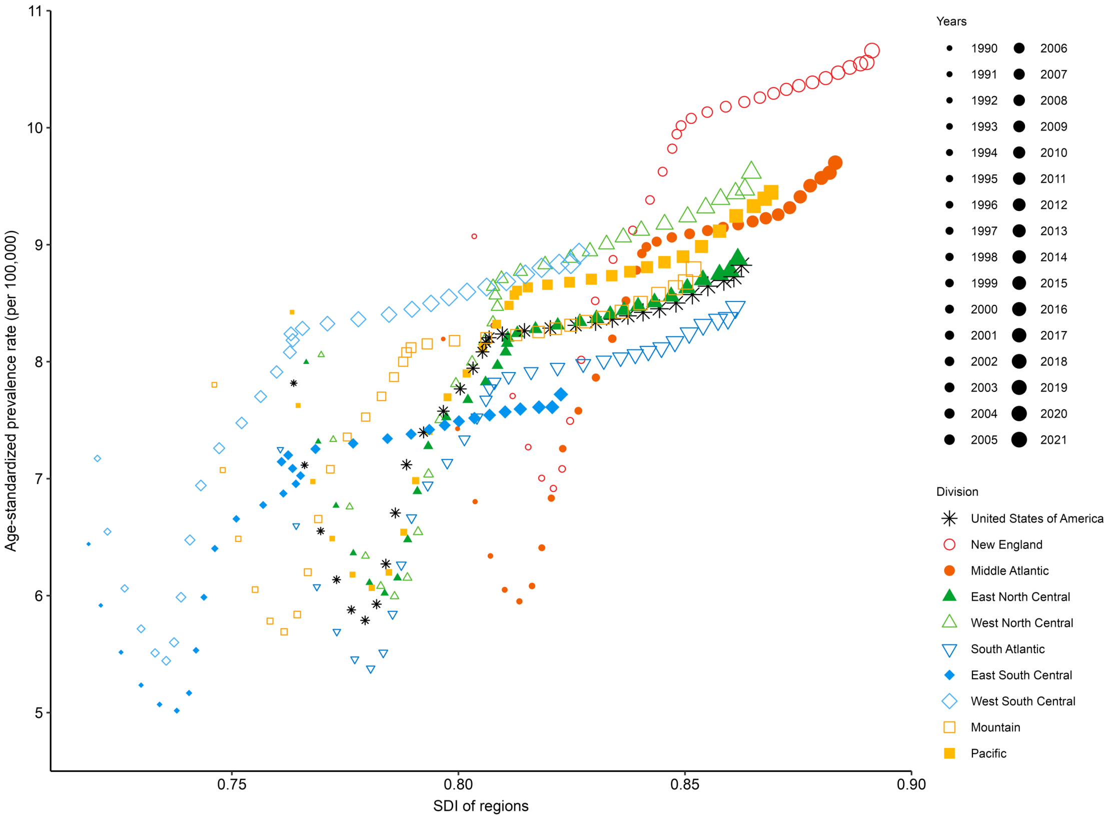

Abbreviations: DALYs, disability-adjusted life years; SDI, sociodemographic index; US, United States; YLDs, years lived with disability; YLLs, years of life lost.

(B) DALY

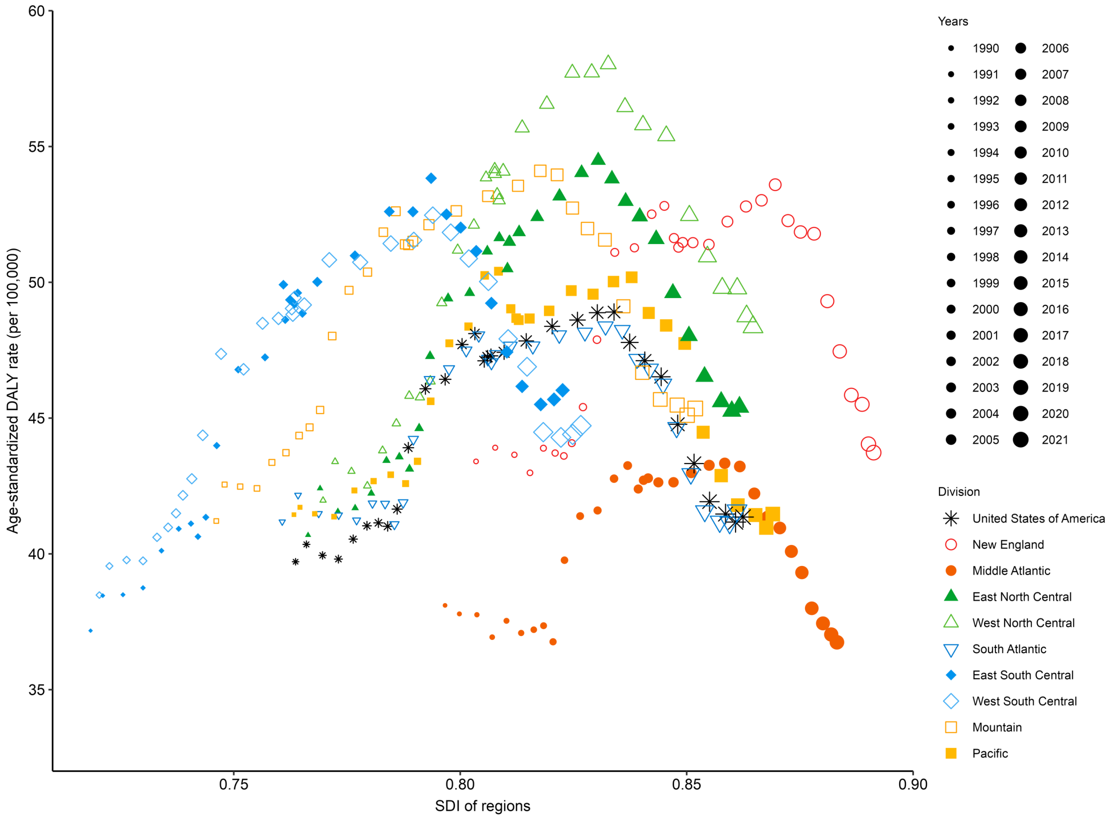

Abbreviations: DALYs, disability-adjusted life years; SDI, sociodemographic index.

(C) death

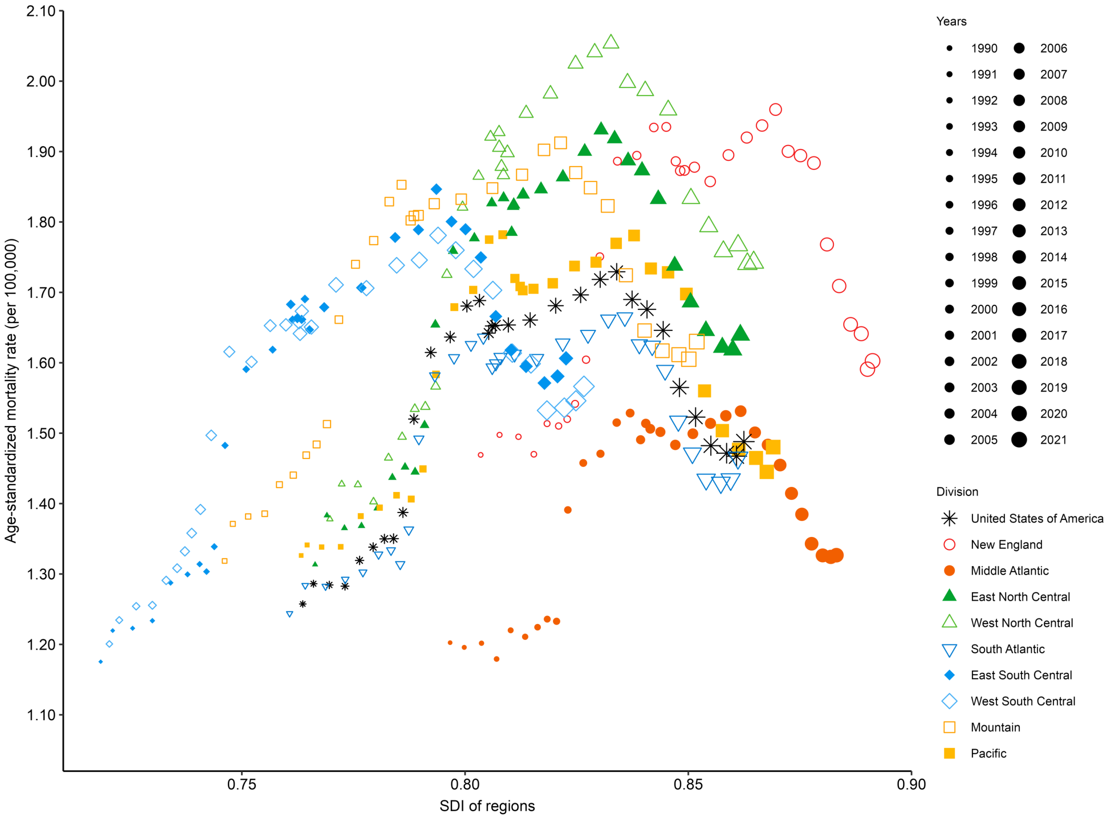

Abbreviations: SDI, sociodemographic index.

(D) incidence

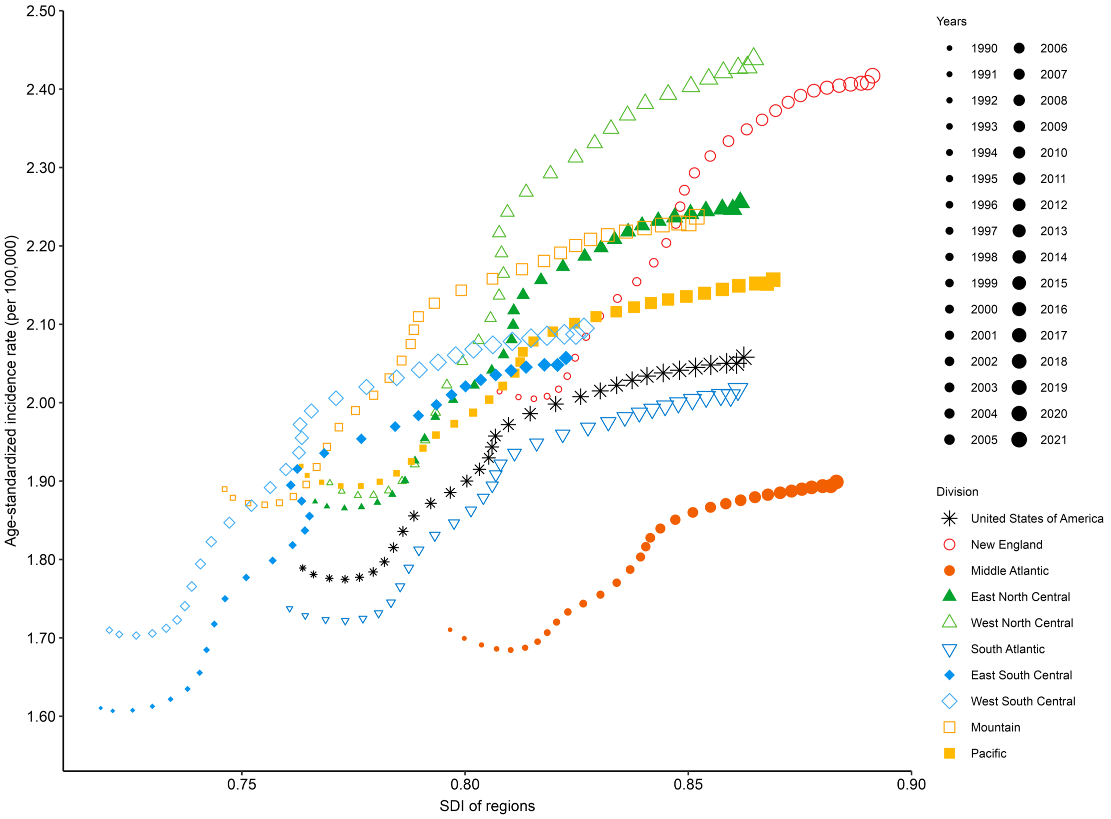

Abbreviations: SDI, sociodemographic index.

(E) YLD

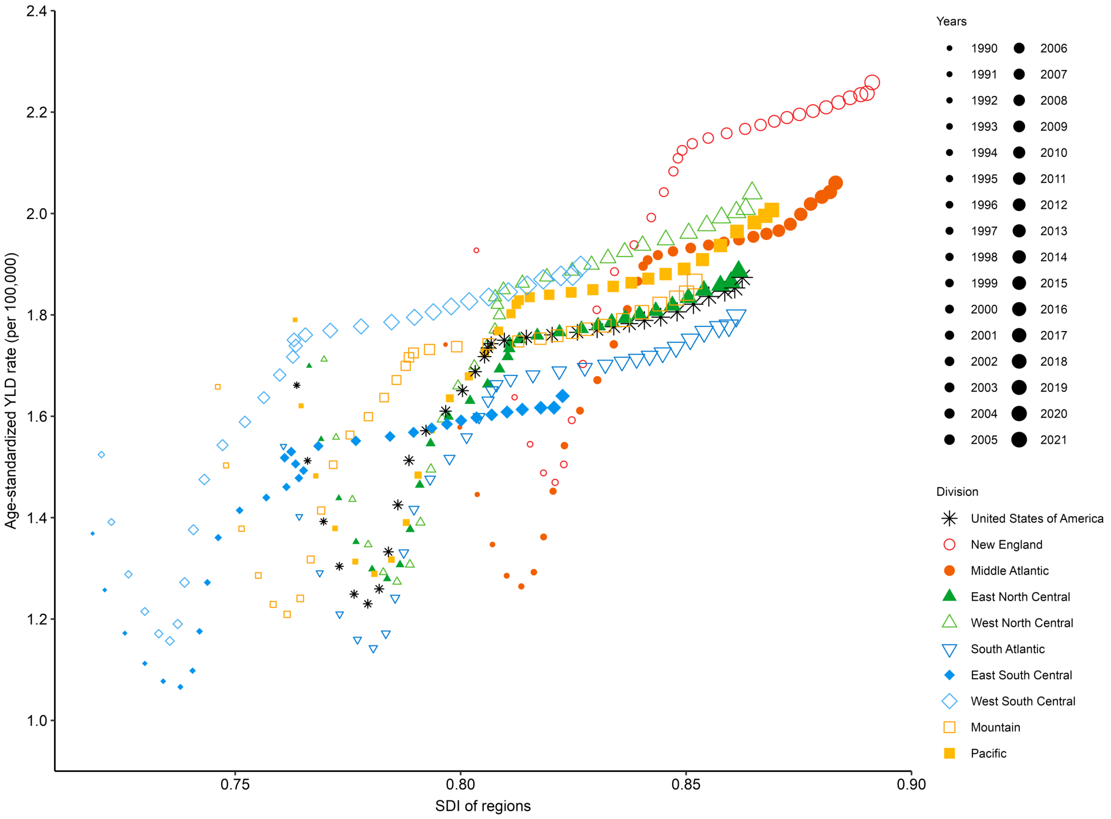

Abbreviations: SDI, sociodemographic index; YLDs, years lived with disability.

(F) YLL

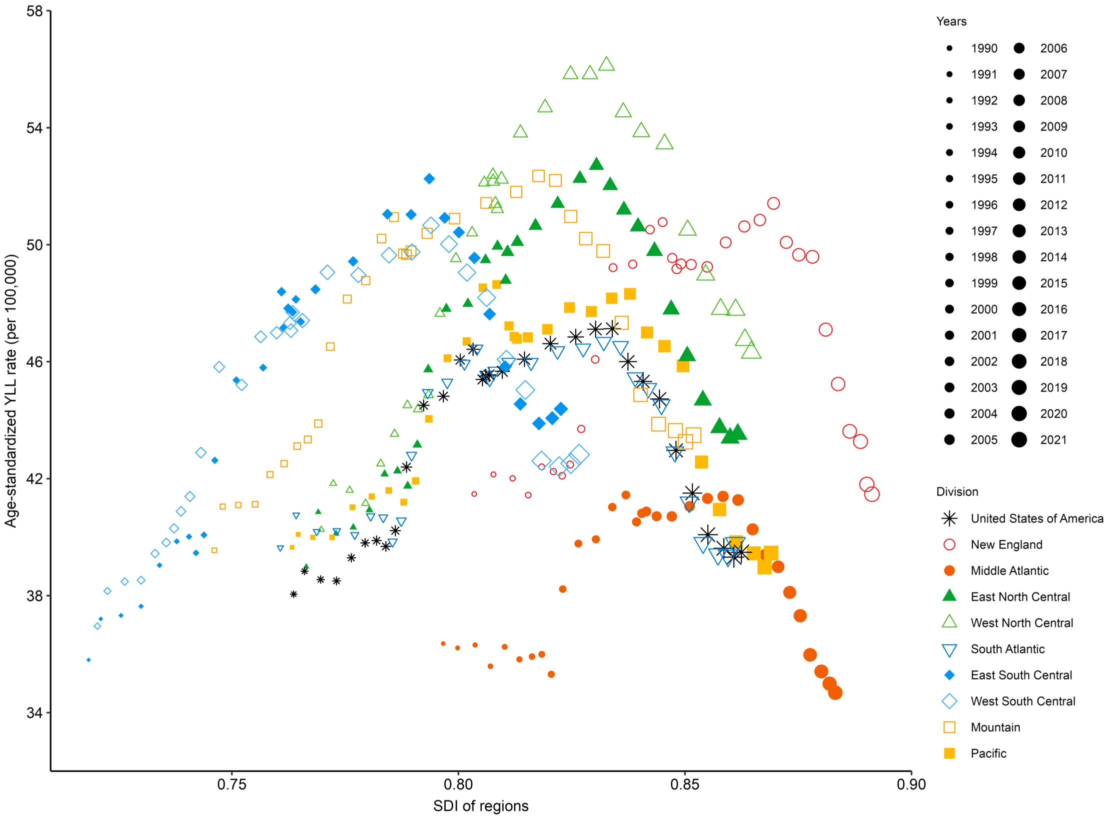

Abbreviations: SDI, sociodemographic index; YLLs, years of life lost.

Figure S12: Age-standardized rates of (A) prevalence, (B) deaths, (C) incidence, (D) YLDs, (E) YLLs according to the sociodemographic index in 2021. The line represents the linear regression model, and the shaded area indicates the 95% confidence interval.

(A) prevalence

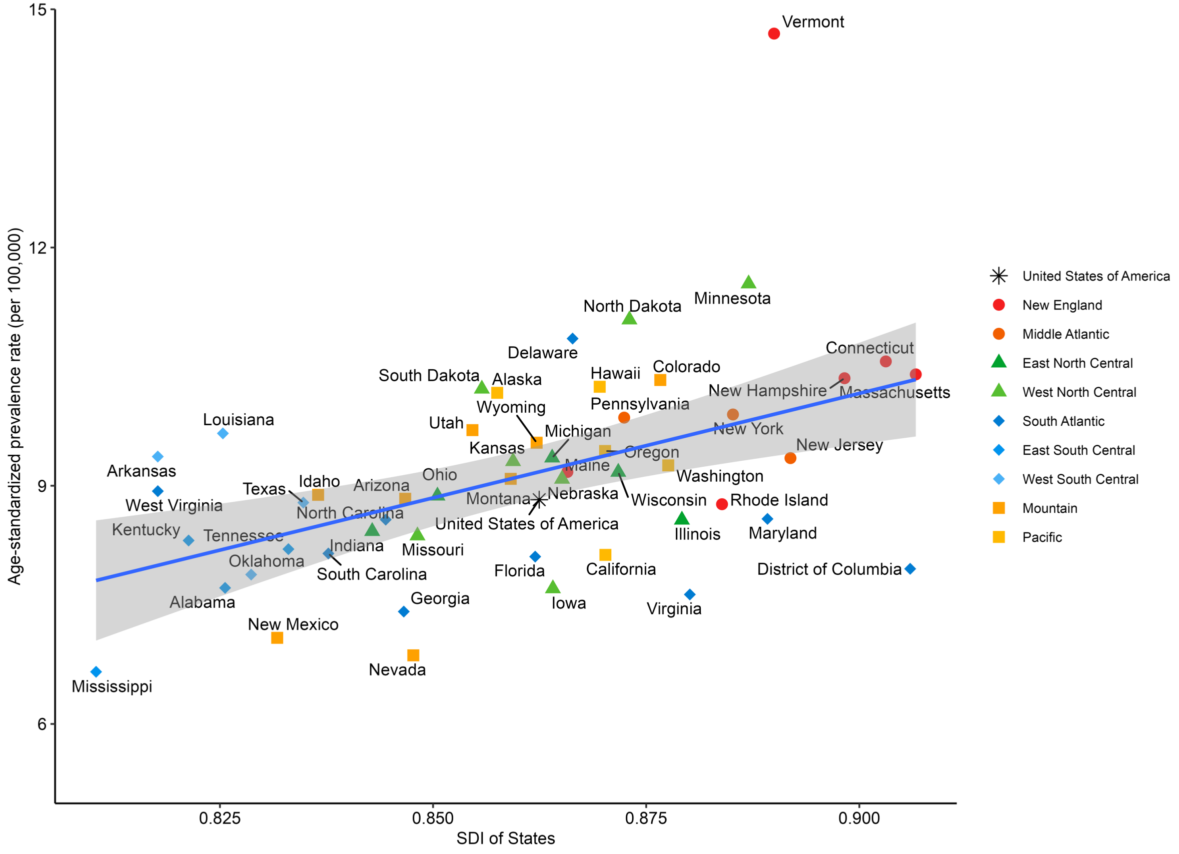

Abbreviations: SDI, sociodemographic index; YLDs, years lived with disability; YLLs, years of life lost.

(B) deaths

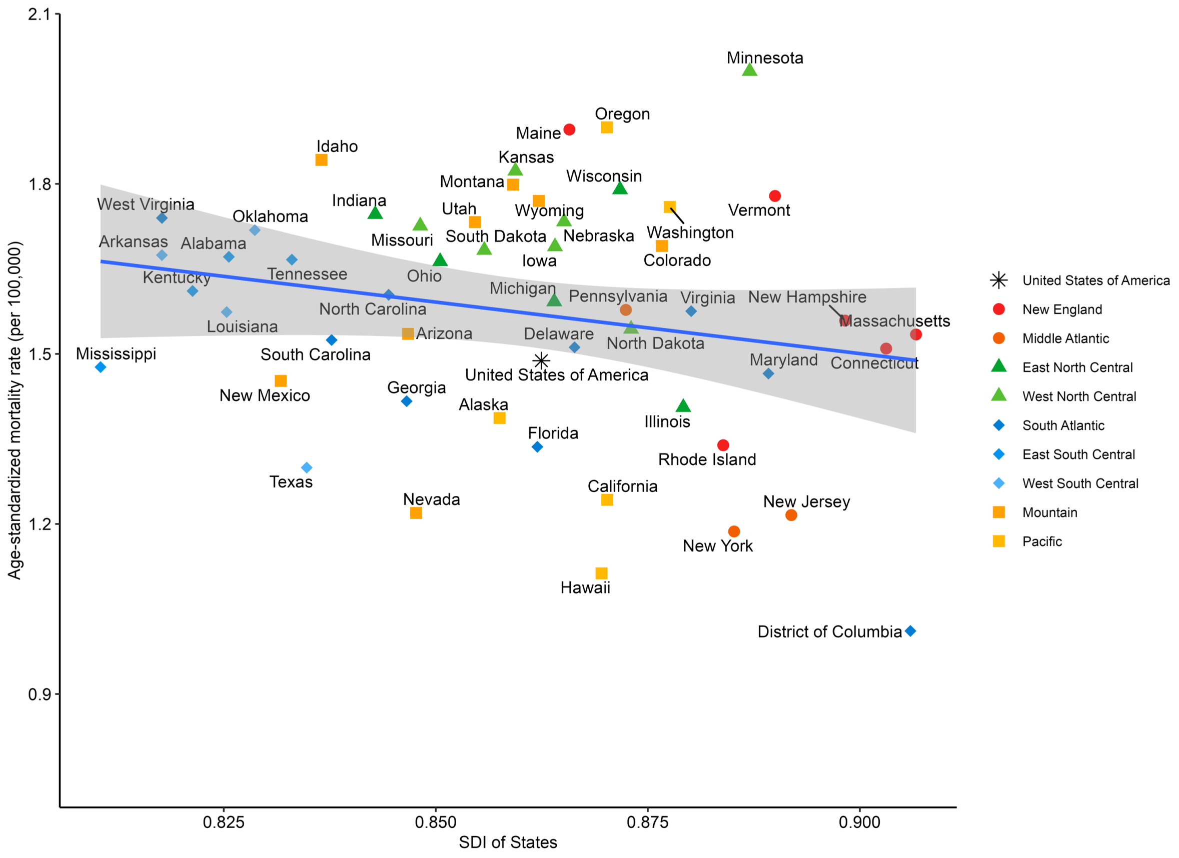

Abbreviations: SDI, sociodemographic index.

(C) incidence

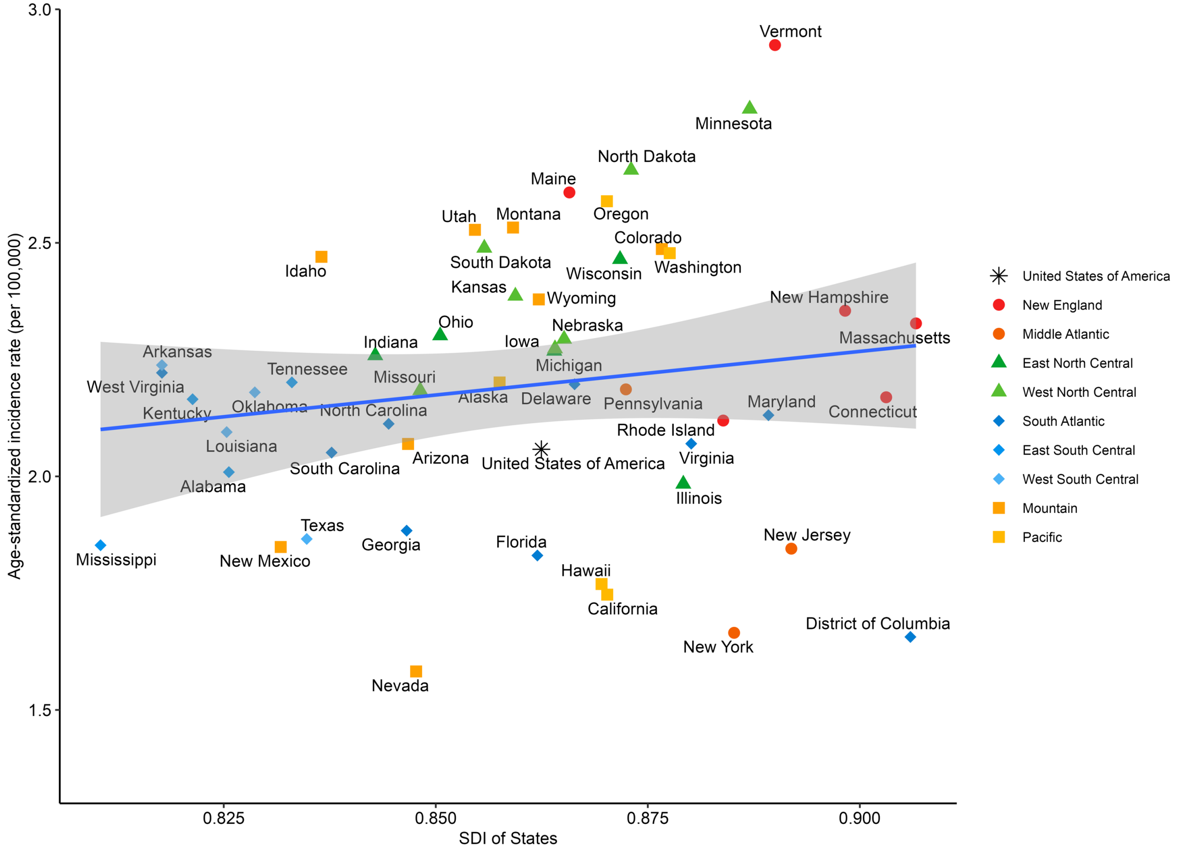

Abbreviations: SDI, sociodemographic index.

(D) YLDs

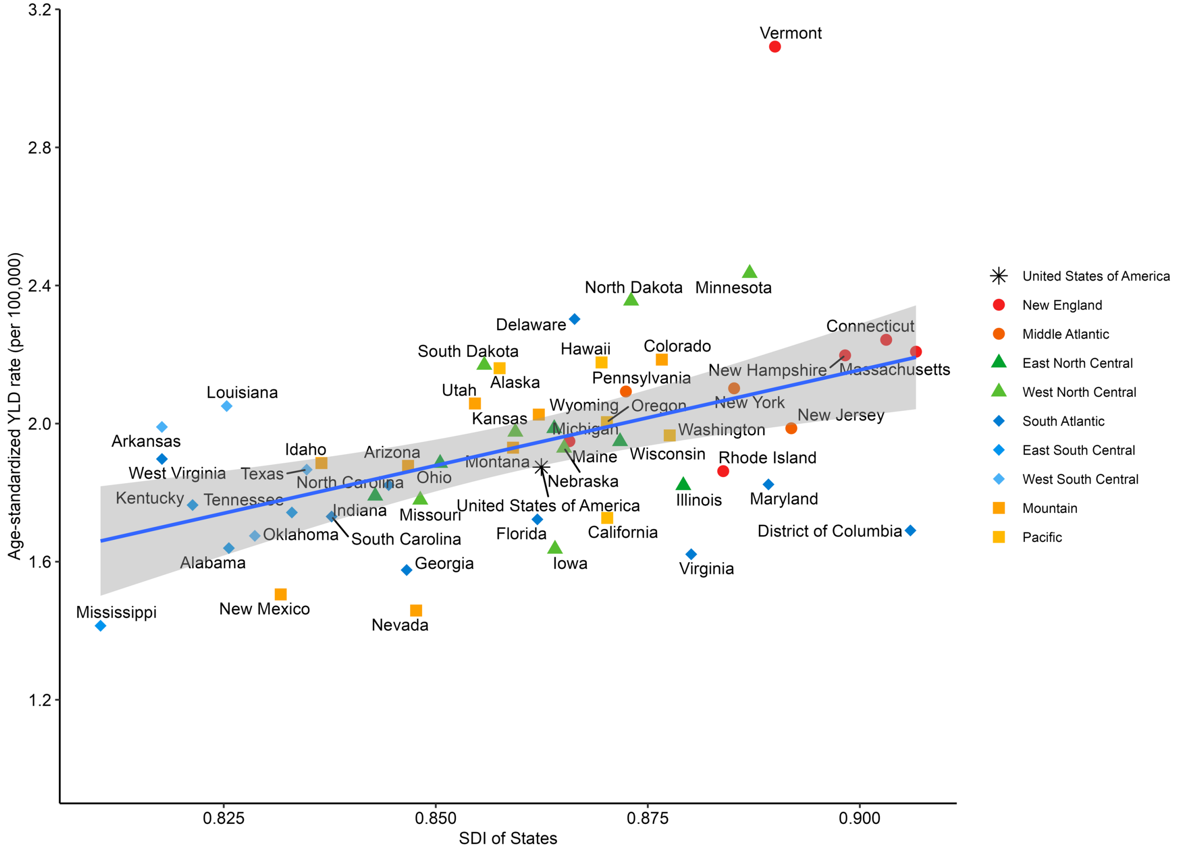

Abbreviations: SDI, sociodemographic index; YLDs, years lived with disability.

(E) YLLs

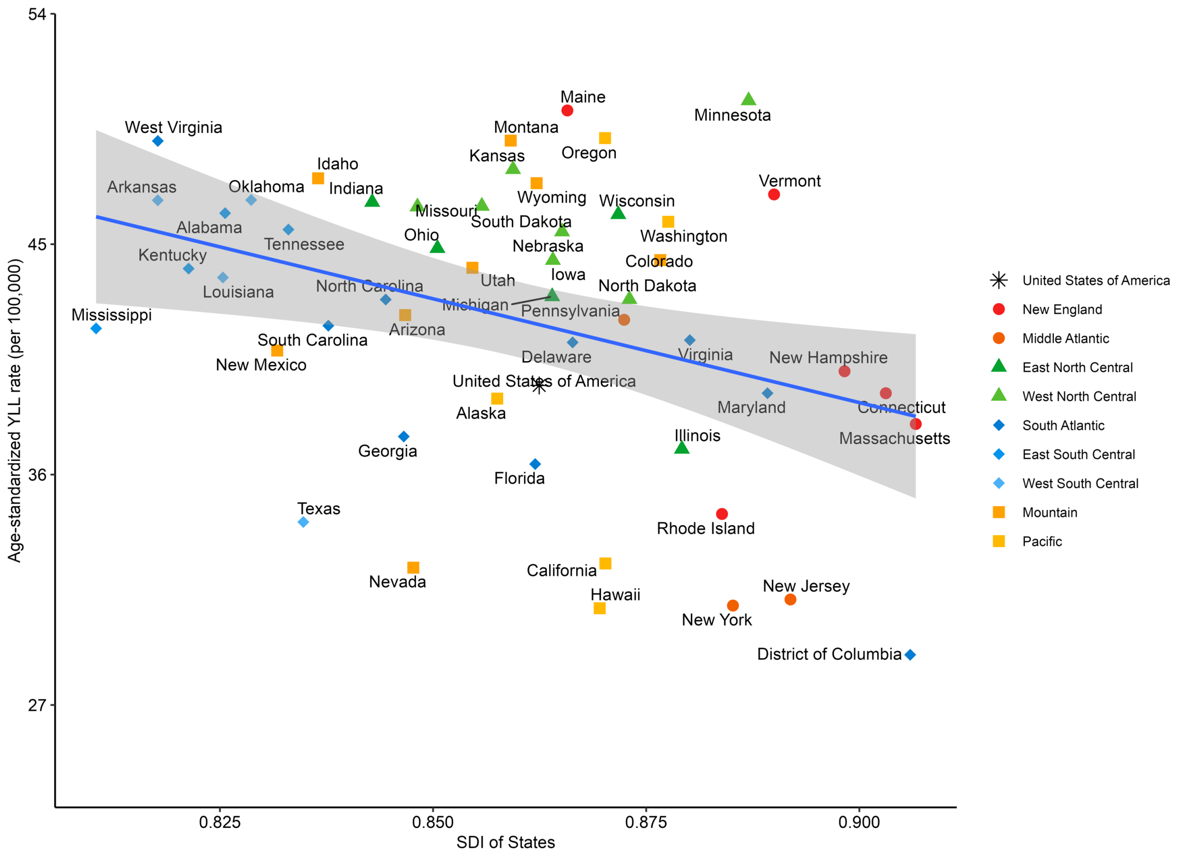

Abbreviations: SDI, sociodemographic index; YLLs, years of life lost.
