# Supplementary material for: The Burden of Motor Neuron Diseases in the United States, 1990–2021: A Systematic Analysis of the Global Burden of Disease Study 2021
Source: Muscle Nerve. 2025 Sep 10;72(5):1130–42. doi: 10.1002/mus.70023 (PMC12529030; doi:10.1002/mus.70023)
Supplement: Supplementary file 2 — Table S1: Numbers, age‐standardized rates, and percentage changes of incidence, prevalence, deaths, DALYs, YLDs, and YLLs, from 1990 to 2021, in US, global, Asia, and Europe Table S2a: Annual age‐standardized prevalence rates and percentage changes from 1990 to 2021, by sex Table S2b: Annual age‐standardized disability‐adjusted life year (DALY) rates and percentage changes from 1990 to 2021, by sex Table S2c: Annual age‐standardized mortality rates and percentage changes from 1990 to 2021, by sex Table S2d: Annual age‐standardized incidence rates and percentage changes from 1990 to 2021, by sex Table S2e: Annual age‐standardized years lived with disability (YLD) rates and percentage changes from 1990 to 2021, by sex Table S2f: Annual age‐standardized years of life lost (YLL) rates and percentage changes from 1990 to 2021, by sex Table S3: Numbers and age‐standardized rates, and percentage changes of Prevalence, DALYs, and deaths in the US. Table S4a: Numbers and age‐standardized rates of prevalence in 2021, and percentage changes from 1990 to 2021, by sex Table S4b: Numbers and age‐standardized rates of disability‐adjusted life years (DALYs) in 2021, and percentage changes from 1990 to 2021, by sex Table S4c: Numbers and age‐standardized rates of deaths in 2021, and percentage changes from 1990 to 2021, by sex Table S4d: Numbers and age‐standardized rates of incidence in 2021, and percentage changes from 1990 to 2021, by sex Table S4e: Numbers and age‐standardized rates of years lived with disability (YLDs) in 2021, and percentage changes from 1990 to 2021, by sex Table S4f: Numbers and age‐standardized rates of years of life lost (YLLs) in 2021, and percentage changes from 1990 to 2021, by sex Table S5a: Prevalence, DALYs, and mortality rates in 2021, by age groups and sex Table S5b: Incidence, YLD, and YLL rates in 2021, by age groups and sex Table S6: Rates of prevalence, DALYs, mortality, incidence, YLD, and YLL in 2021, by age groups Table S7a: Numbers and age‐ [file MUS-72-1130-s001.docx]

Supplementary Materials

[1. Supplementary Methods 3](#_Toc203344272)

[2. Supplementary Tables 15](#_Toc203344273)

[Table S1: Numbers, age-standardized rates, and percentage changes of incidence, prevalence, deaths, DALYs, YLDs, and YLLs, from 1990 to 2021, in US, global, Asia, and Europe 15](#_Toc203344274)

[Table S2a: Annual age-standardized prevalence rates and percentage changes from 1990 to 2021, by sex 17](#_Toc203344275)

[Table S2b: Annual age-standardized disability-adjusted life year (DALY) rates and percentage changes from 1990 to 2021, by sex 20](#_Toc203344276)

[Table S2c: Annual age-standardized mortality rates and percentage changes from 1990 to 2021, by sex 23](#_Toc203344277)

[Table S2d: Annual age-standardized incidence rates and percentage changes from 1990 to 2021, by sex 26](#_Toc203344278)

[Table S2e: Annual age-standardized years lived with disability (YLD) rates and percentage changes from 1990 to 2021, by sex 29](#_Toc203344279)

[Table S2f: Annual age-standardized years of life lost (YLL) rates and percentage changes from 1990 to 2021, by sex 32](#_Toc203344280)

[Table S4a: Numbers and age-standardized rates of prevalence in 2021, and percentage changes from 1990 to 2021, by sex 41](#_Toc203344281)

[Table S4b: Numbers and age-standardized rates of disability-adjusted life years (DALYs) in 2021, and percentage changes from 1990 to 2021, by sex 45](#_Toc203344282)

[Table S4c: Numbers and age-standardized rates of deaths in 2021, and percentage changes from 1990 to 2021, by sex 49](#_Toc203344283)

[Table S4d: Numbers and age-standardized rates of incidence in 2021, and percentage changes from 1990 to 2021, by sex 53](#_Toc203344284)

[Table S4e: Numbers and age-standardized rates of years lived with disability (YLDs) in 2021, and percentage changes from 1990 to 2021, by sex 57](#_Toc203344285)

[Table S4f: Numbers and age-standardized rates of years of life lost (YLLs) in 2021, and percentage changes from 1990 to 2021, by sex 61](#_Toc203344286)

[Table S5a: Prevalence, DALYs, and mortality rates in 2021, by age groups and sex 65](#_Toc203344287)

[Table S5b: Incidence, YLD, and YLL rates in 2021, by age groups and sex 67](#_Toc203344288)

[Table S6: Rates of prevalence, DALYs, mortality, incidence, YLD, and YLL in 2021, by age groups 69](#_Toc203344289)

[Table S7a: Numbers and age-standardized rates of prevalence, DALYs, and deaths in 2021, and percentage changes from 1990 to 2021, with SDI 71](#_Toc203344290)

[Table S7b: Numbers and age-standardized rates of incidence, YLDs, and YLLs in 2021, and percentage changes from 1990 to 2021, with SDI 76](#_Toc203344291)

# 1. Supplementary Methods

**1.1 Overview and Data Source**

Global burden of disease (GBD) 2021 study classifies causes into a four-level hierarchy. Level 1 causes encompass aggregates of non-communicable diseases; injuries; Other COVID-19 pandemic-related outcomes; and a combined category of maternal, and neonatal disorders and nutritional deficiencies. At Level 2, these categories break into 22 aggregated groupings of diseases and injuries, such as cardiovascular diseases, respiratory infections, tuberculosis, neurological disorders, and transport injuries. Level 3 includes specific causes like rheumatic heart disease and road injuries. Level 3 is the most detailed classification, while others have more detailed categories at Level 4, such as ischemic stroke, coal workers pneumoconiosis, and cyclist road injuries. Detailed levels of causes are explained at the GBD 2021 website (https://www.healthdata.org/research-analysis/diseases-injuries/factsheets-overview/about-disease-injury-impairment). Based on these criteria, motor neuron disease (MND) is classified as Level 3.

**1.2 Input data and estimates**

**1.2.1 Flowchart**

**
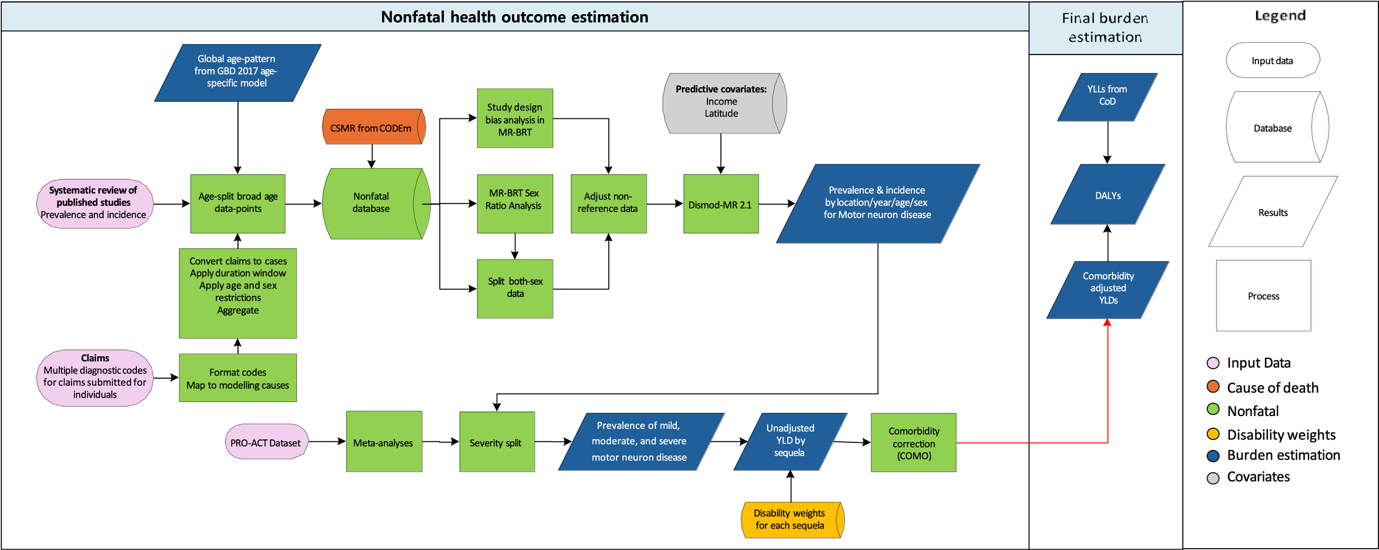
**

**1.2.2 Input data and data processing**

A full systematic review was most recently performed for GBD 2021. The following search string guided our search, which resulted in 3,146 hits with 58 sources meeting extraction criteria: (1) the study is a representative population-based study with well-defined sample, (2) reports on prevalence, incidence, remission, excess mortality, relative risk of mortality, standardized mortality ratio, or with-condition mortality rate for motor neuron diseases in aggregate or a specified motor neuron disease.

Estimates for disability adjusted life years (DALYs), years lived with disability (YLDs), years of life lost (YLLs), and deaths related to MNDs were derived from health claims data, survey microdata, registries, surveillance systems, and systematic evaluations of reports, regardless of publication status. Institute for Health Metrics and Evaluation (IHME) gathered data for the GBD Study by searching various databases, including Embase, PubMed, the System for Information on Gray Literature in Europe, CAP abstracts, Medline, CINAHL, and the World Health Organization Library Information System, without restrictions on age, language, or sex.

They individually searched for terms like (('motor neuron disease'[MeSH Terms] OR ('motor'[All Fields] AND 'neuron'[All Fields] AND 'disease'[All Fields]) OR 'motor neuron disease'[All Fields] OR ('motor'[All Fields] AND 'neuron'[All Fields] AND 'diseases'[All Fields]) OR 'motor neuron diseases'[All Fields]) OR ('amyotrophic lateral sclerosis'[MeSH Terms] OR ('amyotrophic'[All Fields] AND 'lateral'[All Fields] AND 'sclerosis'[All Fields]) OR 'amyotrophic lateral sclerosis'[All Fields]) OR ALS[All Fields] OR ('motor neuron disease'[MeSH Terms] OR ('motor'[All Fields] AND 'neuron'[All Fields] AND 'disease'[All Fields]) OR 'motor neuron disease'[All Fields] OR ('primary'[All Fields] AND 'lateral'[All Fields] AND 'sclerosis'[All Fields]) OR 'primary lateral sclerosis'[All Fields]) OR ('Politics Life Sci'[Journal] OR 'pls'[All Fields]) OR ('muscular atrophy, spinal'[MeSH Terms] OR ('muscular'[All Fields] AND 'atrophy'[All Fields] AND 'spinal'[All Fields]) OR 'spinal muscular atrophy'[All Fields] OR ('progressive'[All Fields] AND 'muscular'[All Fields] AND 'atrophy'[All Fields]) OR 'progressive muscular atrophy'[All Fields]) OR PBP[All Fields] OR ('pseudobulbar palsy'[MeSH Terms] OR ('pseudobulbar'[All Fields] AND 'palsy'[All Fields]) OR 'pseudobulbar palsy'[All Fields])) AND (('epidemiology'[Subheading] OR 'epidemiology'[All Fields] OR 'epidemiology'[MeSH Terms]) OR population-based[All Fields])

Data identified through the systematic review were manually extracted for the GBD 2021. For the GBD 2021, consistent with the approach used in 2019, datapoints corresponding to broad age groups were split based on the estimated age distribution for that specific location, derived from an initial model utilizing only age-specific data. For the GBD 2019, all previously extracted studies were evaluated and assigned a design variable to indicate if the case definition was limited to ALS only or inclusive of all MND types. This classification approach was maintained for GBD 2021.

In addition to data derived from the systematic review, as in previous rounds of the GBD, the IHME made use of claims data as obtained and processed by the GBD Clinical Informatics team. These data link claims for all inpatient and outpatient encounters for a single individual and include both primary and secondary diagnoses for all encounters. An individual was extracted from claims data as a prevalent case if they had any MND code as any diagnosis in one or more inpatient encounters or two or more outpatient encounters.

Total sources used for modelling in GBD 2021 are listed in the table below:

| Measure | Total sources | Countries with data |
| --- | --- | --- |
| All measures | 73 | 18 |
| Prevalence | 24 | 1 |
| Incidence | 48 | 18 |
| Remission | 0 | 0 |
| Other | 1 | 1 |

In GBD 2021, all sex-specific data were used to estimate a pooled sex-ratio using a MR-BRT (meta- regression—Bayesian, regularized, trimmed) model 1 (Additional information can be found in appendix 1, section 4.4.1 of the cited paper). This ratio was integrated with sex-specific population estimates for the year-age-location combinations corresponding to each datapoint reported for both sexes combined, to estimate sex-specific datapoints prior to modelling. Specifically, the male prevalence was calculated using the following method.

$${prev}_{male}={prev}_{both}* \frac{{pop}_{both}}{({pop}_{male}+ratio*{pop}_{femlae})}$$

and then calculating female prevalence:

𝑝𝑟𝑒𝑣_𝑓𝑒𝑚𝑎𝑙𝑒_ = 𝑟𝑎𝑡𝑖𝑜 ∗ 𝑝𝑟𝑒𝑣_𝑚𝑎𝑙𝑒_

(Or the equivalent equations for incidence or other epidemiological measure.)

Two pre-modelling adjustments were then made to adjust for systematic biases in some data sources: (1) data reporting on ALS only, and (2) data from the United States of America (USA) claims in the year 2000 (the U.S. Marketscan database covers a commercially insured sub-population from all U.S. states). Two studies of ALS only were found to be closely matched in year, age, sex and time with three studies of MND more broadly, and the log- ratios for all matched pairs were entered into a separate MR-BRT model.

**MR-BRT Crosswalk Adjustment Factors**

| Data input | Reference or alternative  case definition | Beta Coefficient, Log  (95% CI) * | Adjustment  factor** |
| --- | --- | --- | --- |
| Surveys of all MND using combined clinical, imaging, electrophysiology and imaging criteria  OR  Claims data from location-  years other than USA 2000 | Ref | --- | --- |
| USA claims from year 2000 | Alt | -0.026 (-1.2 to 1.1) | 0.97 (0.31 to 3.1) |
| Surveys limited to ALS only | Alt | -0.13 (-0.23 to -0.029) | 0.88 (0.79 to 0.97) |

**MR-BRT crosswalk adjustments can be interpreted as the factor the alternative case definition is adjusted by to reflect what it would have been had it been measured using the reference case definition. If the log/logit beta coefficient is negative, then the alternative is adjusted up to the reference. If the log/logit beta coefficient is positive, then the alternative is adjusted down to the reference.*

***The adjustment factor column is the exponentiated beta coefficient. For log beta coefficients, this is the relative rate between the two case definitions. For logit beta coefficients, this is the relative odds between the two case definitions.*

Following data extraction and processing, some studies were identified as outliers and were excluded on a case-by-case basis if they were inconsistent with established regional or temporal trends or if concerns about study quality were marked during extraction and processing.

**1.2.3 Claims data processing**

Administrative claims data played a key role in the process of estimating many non-fatal causes and injuries in the GBD 2021 study. Most clinical sources were coded using the International Classification of Diseases (ICD) system that the IHME mapped to GBD-defined diagnosis groups. ICD-9 and ICD-10 codes were mapped to what are termed “ICD code groups” (ICGs) with a many-to-one relationship, which simplifies the disease categorization and reduces complexity. ICGs were then mapped to a disease or injury modelling entity used by GBD modelers. ICD codes used for motor neuron disease were ICD-10 codes G12-G12.9 and ICD-9 codes 335-335.9.


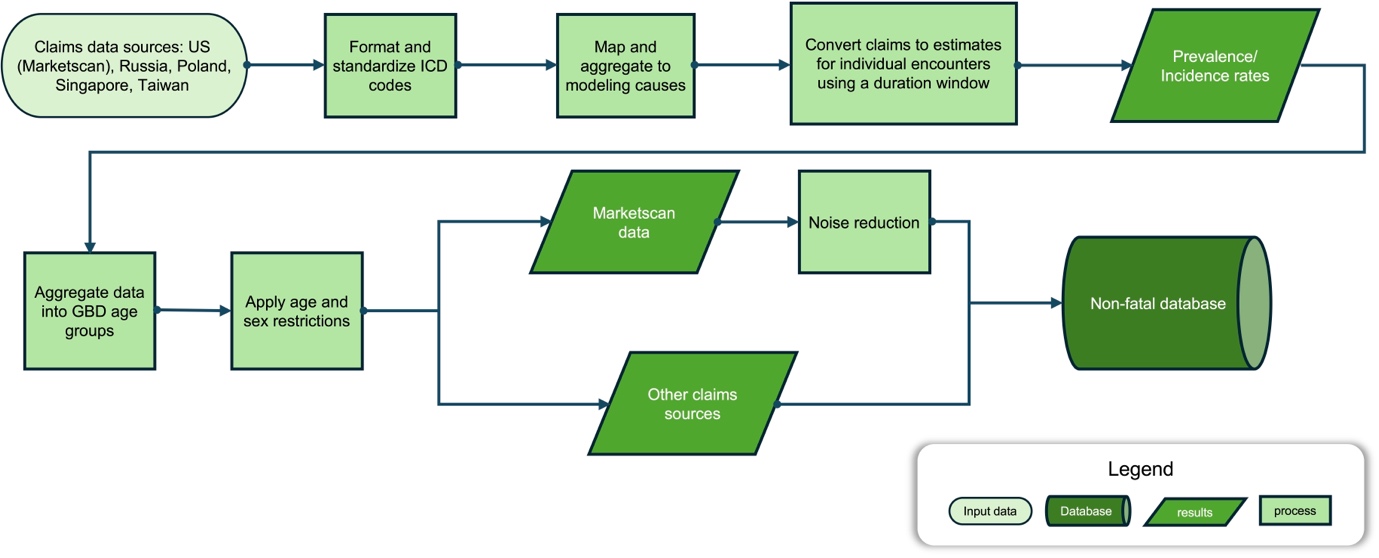


**1.3. Severity of MNDs**

**1.3.1 Severity splits**

The GBD 2021 study provided severity information only for ALS with no corresponding data available for other MND subtypes. To calculate the severity and disability due to MND, the IHME utilized data from Pooled Resource Open-access ALS Clinical Trials (PRO-ACT) database. PRO-ACT is a large ALS clinical trials dataset, with a total of 8,635 ALS patient records from multiple completed clinical trials. Among these, the IHME conducted the final analysis with n=4838 (56%) of the patients with complete ALS Function Rating Scale (ALSFRS) score with average follow-up time of 184 days (min: -22, max: 648), in which 2,999 (62%) received experimental (medication) treatments and 1,301 (27%) received placebo (in these trials, the medications tested were found to be no better than placebo with respect to their effects on ALS progressions).

The ALSFRS is an instrument for assessing the functional status of patients with ALS. It can be used to monitor functional changes in a patient over time. It measures (1) speech, (2) salivation, (3) swallowing, (4) handwriting, (5) cutting food and handling utensils (with or without gastrostomy), (6) dressing and hygiene, (7) turning in bed and adjusting bed clothes, (8) walking, (9) climbing stairs, and (10) breathing. Each task is rated on a 5-point scale from 0 = can’t do, to 4 = normal ability. Individual item scores are summed to produce a reported total score of between 0 and 40 (worst to best). ALSFRS has been revised to ALSFRS-R, which includes 12 questions (ALSFRS Q10 changes to (10) Dyspnea, (11) Orthopnea, and (12) Respiratory insufficiency), with individual item scores summed to a score between 0 and 48.

To eliminate any bias on the ALSFRS scores from treatment effects, only the first observation at the time of trial is included. If the first observation is missing at the time of trial (or prior), the next non-missing observation is selected to be included in the final analysis.

The IHME subsequently mapped ALSFRS scores into GBD severities, and sequelae into different combinations of speech problems, chronic obstructive pulmonary disease, and motor impairment using the following logic:

**Motor impairment:**

The ALSFRS assess motor function of the legs through questions on walking (Q8) and stair climbing (Q9).

| Combined score | Severity level |
| --- | --- |
| 8 | None |
| 5-7 | Mild |
| 2-4 | Moderate |
| 0-1 | Severe |

The ALSFRS also assesses motor impairment through questions on handwriting (Q4), cutting food and handling utensils (Q5), and dressing and hygiene (Q6).

| Combined score | Severity level |
| --- | --- |
| 12 | None |
| 9-11 | Mild |
| 3-8 | Moderate |
| 0-2 | Severe |

After determining case severity on these two separate metrics, the IHME aggregate by taking the most severe ranking (eg, severe + mild = a severe case).

**Respiratory problems:**

Question 10 of the ALSFRS describes breathing difficulty as a function of MND.

| ALSFRS score | Description | Severity level |
| --- | --- | --- |
| 4 | Normal | None |
| 3 | Shortness of breath with  minimal exertion | Mild |
| 2 | Shortness of breath at rest | Moderate |
| 0-1 | Intermittent ventilator assistance required/ventilator-  dependent | Severe |

**Speech problems:**

Speech impairment due to MND is derived from ALSFRS question 1, which describes speech impediments. A score of 4 on this question denotes no impairment, while all other values suggest some impairment.

**1.3.2 Creating sequelae and disability weighting**

After determining the severity status of each case for the three symptom umbrellas, the IHME subsequently estimated the relative proportion of each combination of symptom class and their respective severities. Those without any symptoms (eg, no severity) were categorized as having worry about the diagnosis for disability estimation. The following table displays the various sequelae and their associated proportions.

| Sequela | Proportion  (Mean) | Proportion  (Lower) | Proportion  (Upper) |
| --- | --- | --- | --- |
| Mild motor impairment, mild respiratory problems and  speech problems due to motor neuron disease | 0.01779 | 0.01658 | 0.01909 |
| Mild motor impairment, moderate respiratory problems and  speech problems due to motor neuron disease | 0.00270 | 0.00225 | 0.00324 |
| Mild motor impairment, severe respiratory problems and  speech problems due to motor neuron disease | 0.00082 | 0.00059 | 0.00113 |
| Mild motor impairment, and speech problems due to motor  neuron disease | 0.02052 | 0.01922 | 0.02190 |
| Moderate motor impairment, mild respiratory problems and  speech problems due to motor neuron disease | 0.03377 | 0.03210 | 0.03552 |
| Moderate motor impairment, moderate respiratory  problems and speech problems due to motor neuron disease | 0.00715 | 0.00640 | 0.00799 |
| Moderate motor impairment, severe respiratory problems  and speech problems due to motor neuron disease | 0.00286 | 0.00240 | 0.00342 |
| Moderate motor impairment, and speech problems due to  motor neuron disease | 0.03041 | 0.02883 | 0.03208 |
| Severe motor impairment, mild respiratory problems and  speech problems due to motor neuron disease | 0.05242 | 0.05035 | 0.05457 |
| Severe motor impairment, moderate respiratory problems  and speech problems due to motor neuron disease | 0.02247 | 0.02111 | 0.02392 |
| Severe motor impairment, severe respiratory problems and  speech problems due to motor neuron disease | 0.01365 | 0.01259 | 0.01479 |
| Severe motor impairment and speech problems due to  motor neuron disease | 0.04765 | 0.04567 | 0.04970 |
| Mild respiratory problems and speech problems due to  motor neuron disease | 0.01157 | 0.01060 | 0.01263 |
| Moderate respiratory problems and speech problems due to  motor neuron disease | 0.00142 | 0.00111 | 0.00182 |
| Severe respiratory problems and speech problems due to  motor neuron disease | 0.00023 | 0.00013 | 0.00043 |
| Speech problems due to motor neuron disease | 0.02457 | 0.02315 | 0.02608 |
| Mild motor impairment and mild respiratory problems due  to motor neuron disease | 0.02245 | 0.02109 | 0.02389 |
| Mild motor impairment and moderate respiratory problems  due to motor neuron disease | 0.00275 | 0.00230 | 0.00329 |
| Mild motor impairment and severe respiratory problems due  to motor neuron disease | 0.00068 | 0.00047 | 0.00097 |
| Mild motor impairment due to motor neuron disease | 0.10388 | 0.10103 | 0.10681 |
| Moderate motor impairment and mild respiratory problems  due to motor neuron disease | 0.06744 | 0.06511 | 0.06985 |
| Moderate motor impairment and moderate respiratory  problems due to motor neuron disease | 0.01302 | 0.01199 | 0.01413 |
| Moderate motor impairment and severe respiratory  problems due to motor neuron disease | 0.00412 | 0.00356 | 0.00477 |
| Moderate motor impairment due to motor neuron disease | 0.20136 | 0.19760 | 0.20518 |
| Severe motor impairment and mild respiratory problems due  to motor neuron disease | 0.06902 | 0.06666 | 0.07146 |
| Severe motor impairment and moderate respiratory  problems due to motor neuron disease | 0.02000 | 0.01872 | 0.02137 |
| Severe motor impairment and severe respiratory problems  due to motor neuron disease | 0.01062 | 0.00969 | 0.01163 |
| Severe motor impairment due to motor neuron disease | 0.15037 | 0.14702 | 0.15378 |
| Mild respiratory problems due to motor neuron disease | 0.00643 | 0.00571 | 0.00723 |
| Moderate respiratory problems due to motor neuron  disease | 0.00044 | 0.00028 | 0.00069 |
| Severe respiratory problems due to motor neuron disease | 0.00005 | 0.00001 | 0.00017 |
| Asymptomatic, but worry about diagnosis due to motor  neuron disease | 0.03738 | 0.03562 | 0.03921 |
| Moderate motor impairment, moderate respiratory  problems and speech problems due to motor neuron disease | 0.00715 | 0.00640 | 0.00799 |
| Moderate motor impairment, severe respiratory problems  and speech problems due to motor neuron disease | 0.00286 | 0.00240 | 0.00342 |
| Moderate motor impairment, and speech problems due to  motor neuron disease | 0.03041 | 0.02883 | 0.03208 |
| Severe motor impairment, mild respiratory problems and  speech problems due to motor neuron disease | 0.05242 | 0.05035 | 0.05457 |
| Severe motor impairment, moderate respiratory problems  and speech problems due to motor neuron disease | 0.02247 | 0.02111 | 0.02392 |
| Severe motor impairment, severe respiratory problems and  speech problems due to motor neuron disease | 0.01365 | 0.01259 | 0.01479 |
| Severe motor impairment and speech problems due to  motor neuron disease | 0.04765 | 0.04567 | 0.04970 |
| Mild respiratory problems and speech problems due to  motor neuron disease | 0.01157 | 0.01060 | 0.01263 |
| Moderate respiratory problems and speech problems due to  motor neuron disease | 0.00142 | 0.00111 | 0.00182 |
| Severe respiratory problems and speech problems due to  motor neuron disease | 0.00023 | 0.00013 | 0.00043 |
| Speech problems due to motor neuron disease | 0.02457 | 0.02315 | 0.02608 |
| Mild motor impairment and mild respiratory problems due  to motor neuron disease | 0.02245 | 0.02109 | 0.02389 |
| Mild motor impairment and moderate respiratory problems  due to motor neuron disease | 0.00275 | 0.00230 | 0.00329 |
| Mild motor impairment and severe respiratory problems due  to motor neuron disease | 0.00068 | 0.00047 | 0.00097 |
| Mild motor impairment due to motor neuron disease | 0.10388 | 0.10103 | 0.10681 |
| Moderate motor impairment and mild respiratory problems  due to motor neuron disease | 0.06744 | 0.06511 | 0.06985 |
| Moderate motor impairment and moderate respiratory  problems due to motor neuron disease | 0.01302 | 0.01199 | 0.01413 |
| Moderate motor impairment and severe respiratory  problems due to motor neuron disease | 0.00412 | 0.00356 | 0.00477 |
| Moderate motor impairment due to motor neuron disease | 0.20136 | 0.19760 | 0.20518 |
| Severe motor impairment and mild respiratory problems due  to motor neuron disease | 0.06902 | 0.06666 | 0.07146 |
| Severe motor impairment and moderate respiratory  problems due to motor neuron disease | 0.02000 | 0.01872 | 0.02137 |
| Severe motor impairment and severe respiratory problems  due to motor neuron disease | 0.01062 | 0.00969 | 0.01163 |
| Severe motor impairment due to motor neuron disease | 0.15037 | 0.14702 | 0.15378 |
| Mild respiratory problems due to motor neuron disease | 0.00643 | 0.00571 | 0.00723 |
| Moderate respiratory problems due to motor neuron  disease | 0.00044 | 0.00028 | 0.00069 |
| Severe respiratory problems due to motor neuron disease | 0.00005 | 0.00001 | 0.00017 |
| Asymptomatic, but worry about diagnosis due to motor  neuron disease | 0.03738 | 0.03562 | 0.03921 |

To determine disability due to these sequelae, the IHME use the standard multiplicative aggregation formula as described in the main text. The following table provides description and disability weight assigned to the sequelae as appropriate.

| Symptom  group | Severity level | Lay description | Disability Weights  (95%) |
| --- | --- | --- | --- |
| Respiratory  problems | Asymptomatic |  |  |
| Respiratory problems | Mild | Has cough and shortness of breath after heavy physical activity, but is able to  walk long distances and climb stairs. | 0.019  (0.011–0.033) |
| Respiratory problems | Moderate | Has cough, wheezing, and shortness of breath, even after light physical activity. The person feels tired and can walk only short distances or climb only a few  stairs. | 0.225  (0.153–0.31) |
| Respiratory problems | Severe | Has cough, wheezing, and shortness of breath all the time. The person has great difficulty walking even short distances or climbing any stairs, feels  tired when at rest and is anxious. | 0.408  (0.273–0.556) |
| Motor  impairment | Asymptomatic |  |  |
| Motor impairment | Mild | Has some difficulty in moving around but is able to walk without help. | 0.01  (0.005–0.019) |
| Motor impairment | Moderate | Has some difficulty in moving around and difficulty in lifting and holding objects, dressing, and sitting upright,  but is able to walk without help. | 0.061  (0.04–0.089) |
| Motor impairment | Severe | Is unable to move around without help, and is not able to lift or hold objects,  get dressed or sit upright. | 0.402  (0.268–0.545) |
| Speech  problems | No |  |  |
| Speech problems | Yes | Has difficulty speaking, and others find it difficult to understand. | 0.051  (0.032–0.078) |
| Asymptomatic, but worry | Yes | Has a disease diagnosis that causes some worry but minimal interference  with daily activities. | 0.012  (0.006–0.023) |

**1.4 Modelling strategy**

For the estimation of MND burden, DisMod-MR 2.1 [1] (disease model—Bayesian meta-regression, details on this method can be found in appendix 1, section 4.5 of the citation) were used as the primary analytical tool. Inputs included prevalence and incidence data, as described above, as well as the cause-specific mortality rate (CSMR) estimated in the GBD causes of death analysis, and excess mortality rate (EMR) obtained by dividing CSMR by prevalence datapoints. Prior settings are limited to 0 remission at all ages and maximum incidence of 0.0004. The IHME also constrain the super-region random effects for prevalence and incidence to -0.5 and 0.5 to account for spurious inflation of regional differences.

To improve model predictions, two covariates were employed:

| Covariate | Measure | Beta coefficient  (95% CI) | Exponentiated |
| --- | --- | --- | --- |
| Absolute value of average  latitude | Prevalence | 0.037 (0.036 to 0.038) | 1.04 (1.04 to 1.04) |
| LDI (I$ per capita) | Excess mortality rate | -0.5 (-0.5 to -0.5) | 0.61 (0.61 to 0.61) |

While no known cures have been established for MND, the IHME expect disease management to differ globally – largely as a function of available resources. To capture this, the IHME employ the natural log of lagged distributed income per capita as a proxy to capture this relationship in the estimation of excess mortality.

Additionally, prior studies have reported a potential association between extreme latitude with higher prevalence and incidence of MND, although the pathway to explain the association is not understood. Our operationalization of latitude is created by taking the absolute value of a population-weighted average of latitude by country. The underlying population distribution rasters are part of the Gridded Population of the World dataset.

**1.5 Model validation strategy**

To ensure the reliability of the model assumptions used in the Global Burden of Disease (GBD) 2021 study, the GBD team implemented a range of validation strategies throughout the modeling process:

(1) Comparison with external data sources: GBD results were compared with data from registries, surveys, and other independent studies (e.g., WHO, national statistics agencies, civil registration and vital statistics, NHANES, and etc.) to assess consistency. This was validated through an extensive set of 56,604 data sources, representing the most comprehensive comparison ever conducted in a modeling study to date.

(2) Expert consultation and systematic reviews: The GBD study included regular consultations with disease experts and relied on thousands of peer-reviewed studies to calibrate assumptions used in the model, particularly around natural history, severity distributions, and treatment coverage. The GBD study conducted separate systematic reviews for each of the 371 diseases, thereby providing robust and disease-specific evidence.

(3) Methodological validation:

i) Out-of-sample predictive validation: GBD employed a train-test 1-test 2 strategy where models were developed on a training dataset, optimized on a first test dataset, and then validated on a completely separate second test dataset to ensure unbiased assessment of model performance.

ii) Systematic covariate selection: all covariates included in GBD models were systematically tested to ensure they demonstrated the expected direction of effect and statistical significance, validating our assumptions about relationships between predictors and outcomes.

iii) Internal consistency checks: GBD models enforced consistency between epidemiological parameters, ensuring that relationships between incidence, prevalence, and mortality rates aligned with established epidemiological theory.

(4) Model diagnostics and transparency: The GBD team publicly shared modeling strategies and diagnostics (e.g., uncertainty intervals, trace plots, residuals) in their appendices or through the IHME GBD Compare tool, which allowed independent researchers to inspect patterns and spot anomalies.

[1] Vos T, Lim SS, Abbafati C, et al. Global burden of 369 diseases and injuries in 204 countries and territories, 1990–2019: a systematic analysis for the Global Burden of Disease Study 2019. The Lancet 2020; 396: 1204–22. doi: <https://doi.org/10.1016/S0140-6736(20)30925-9>

# 2. Supplementary Tables

## Table S1: Numbers, age-standardized rates, and percentage changes of incidence, prevalence, deaths, DALYs, YLDs, and YLLs, from 1990 to 2021, in US, global, Asia, and Europe

|  |  | **Incidence**  **(95% UI)** | **Prevalence**  **(95% UI)** | **Deaths**  **(95% UI)** | **DALYs**  **(95% UI)** | **YLDs**  **(95% UI)** | **YLLs**  **(95% UI)** |
| --- | --- | --- | --- | --- | --- | --- | --- |
| **United States**  **of America** | **Absolute number, thousands, 1990** | 5280.94 (5003.51 to 5603.93) | 22197.46 (19256.54 to 25429.67) | 3890.20 (3686.90 to 4008.21) | 111651.92 (107615.58 to 114636.27) | 4717.00 (3366.69 to 6414.58) | 106934.92 (103345.08 to 109329.44) |
|  | **Age-standardized rate, per 100 000 people, 1990** | 1.79 (1.69 to 1.91) | 7.82 (6.82 to 8.92) | 1.26 (1.20 to 1.29) | 39.71 (38.48 to 40.72) | 1.66 (1.18 to 2.24) | 38.05 (36.95 to 38.85) |
|  | **Absolute number, thousands, 2021** | 11089.01 (10633.99 to 11559.06) | 40495.43 (37645.36 to 43576.27) | 8465.56 (7799.92 to 8898.99) | 210559.45 (199250.06 to 218996.64) | 8597.05 (6259.71 to 11269.09) | 201962.40 (191037.84 to 210306.37) |
|  | **Age-standardized rate, per 100 000 people, 2021** | 2.06 (1.98 to 2.14) | 8.82 (8.26 to 9.51) | 1.49 (1.38 to 1.56) | 41.36 (39.47 to 42.94) | 1.87 (1.36 to 2.46) | 39.48 (37.75 to 40.98) |
|  | **Percentage Change, 1990-2021** | 15.03% (11.49% to 19.04%) | 12.89% (3.10% to 23.66%) | 18.34% (13.86% to 22.70%) | 4.14% (0.41% to 7.68%) | 12.80% (2.98% to 23.54%) | 3.76% (-0.25% to 7.38%) |
| **Global** | **Absolute number, thousands, 1990** | 36769.25 (33067.78 to 41299.83) | 161926.07 (137005.78 to 189262.55) | 15260.07 (14366.61 to 16042.60) | 506146.42 (462034.99 to 545049.77) | 34425.85 (23884.86 to 47211.93) | 471720.57 (427708.75 to 507715.98) |
|  | **Age-standardized rate, per 100 000 people, 1990** | 0.81 (0.72 to 0.90) | 3.36 (2.87 to 3.92) | 0.38 (0.36 to 0.40) | 11.22 (10.39 to 11.98) | 0.71 (0.50 to 0.97) | 10.51 (9.70 to 11.20) |
|  | **Absolute number, thousands, 2021** | 64177.73 (58506.16 to 70270.30) | 272732.25 (236194.50 to 313676.27) | 39081.64 (35756.51 to 42432.82) | 1040565.98 (963064.26 to 1123956.29) | 57889.10 (40686.61 to 78030.59) | 982676.88 (904588.13 to 1064643.84) |
|  | **Age-standardized rate, per 100 000 people, 2021** | 0.77 (0.70 to 0.84) | 3.31 (2.86 to 3.80) | 0.46 (0.42 to 0.49) | 12.17 (11.24 to 13.15) | 0.70 (0.49 to 0.96) | 11.46 (10.52 to 12.43) |
|  | **Percentage Change, 1990-2021** | -4.69% (-6.53% to -2.86%) | -1.36% (-3.28% to 0.87%) | 19.84% (10.79% to 30.39%) | 8.42% (-0.78% to 18.64%) | -1.49% (-3.45% to 0.70%) | 9.10% (-0.70% to 19.99%) |
| **Asia** | **Absolute number, thousands, 1990** | 15462.51 (13357.02 to 18059.19) | 64900.43 (52068.74 to 79426.79) | 3167.78 (2409.81 to 3665.06) | 143029.69 (102839.52 to 169391.83) | 13805.45 (9133.88 to 19495.70) | 129224.24 (89297.89 to 155196.80) |
|  | **Age-standardized rate, per 100 000 people, 1990** | 0.56 (0.48 to 0.65) | 2.13 (1.74 to 2.56) | 0.14 (0.11 to 0.16) | 5.18 (3.89 to 6.05) | 0.45 (0.30 to 0.63) | 4.73 (3.47 to 5.56) |
|  | **Absolute number, thousands, 2021** | 22711.92 (19411.40 to 26490.45) | 103790.47 (84253.56 to 125081.68) | 8553.49 (7010.38 to 10160.32) | 252770.72 (201243.16 to 301064.00) | 22072.20 (14839.01 to 30703.82) | 230698.52 (179178.95 to 276855.11) |
|  | **Age-standardized rate, per 100 000 people, 2021** | 0.48 (0.41 to 0.55) | 2.19 (1.78 to 2.64) | 0.17 (0.14 to 0.20) | 5.12 (4.06 to 6.04) | 0.47 (0.31 to 0.65) | 4.65 (3.60 to 5.58) |
|  | **Percentage Change, 1990-2021** | -14.85% (-16.66% to -12.85%) | 2.49% (-0.05% to 5.51%) | 23.37% (0.12% to 51.51%) | -1.29% (-23.29% to 33.49%) | 2.48% (-0.06% to 5.50%) | -1.65% (-25.59% to 36.96%) |
| **Europe** | **Absolute number, thousands, 1990** | 10882.38 (10068.60 to 11723.34) | 51836.95 (45108.23 to 59778.31) | 7201.60 (6880.05 to 7482.99) | 212503.73 (203833.69 to 224394.46) | 11013.71 (7819.57 to 14862.50) | 201490.02 (193687.20 to 213145.62) |
|  | **Age-standardized rate, per 100 000 people, 1990** | 1.17 (1.08 to 1.26) | 5.74 (4.98 to 6.61) | 0.72 (0.69 to 0.75) | 23.74 (22.67 to 25.56) | 1.22 (0.86 to 1.65) | 22.52 (21.59 to 24.17) |
|  | **Absolute number, thousands, 2021** | 19078.07 (17992.21 to 20111.73) | 83361.94 (72590.68 to 96071.27) | 16894.76 (15348.35 to 18501.86) | 422075.93 (390517.62 to 456792.65) | 17636.54 (12575.11 to 23634.31) | 404439.38 (373856.47 to 438649.23) |
|  | **Age-standardized rate, per 100 000 people, 2021** | 1.43 (1.35 to 1.53) | 6.97 (6.07 to 7.98) | 1.12 (1.03 to 1.23) | 33.06 (30.76 to 35.81) | 1.48 (1.06 to 2.00) | 31.58 (29.28 to 34.29) |
|  | **Percentage Change, 1990-2021** | 22.97% (19.82% to 26.14%) | 21.33% (17.80% to 24.87%) | 55.53% (43.77% to 71.11%) | 39.24% (28.01% to 53.73%) | 21.00% (17.50% to 24.40%) | 40.23% (28.43% to 55.68%) |

Abbreviations: DALYs, disability-adjusted life years; UI, uncertainty interval; YLDs, years lived with disability; YLLs, years of life lost.

## Table S2a: Annual age-standardized prevalence rates and percentage changes from 1990 to 2021, by sex

|  | **Both** | | **Male** | | **Female** | |
| --- | --- | --- | --- | --- | --- | --- |
|  | **Absolute number** | **Age-standardized rate, per 100,000 people** | **Absolute number** | **Age-standardized rate, per 100,000 people** | **Absolute number** | **Age-standardized rate, per 100,000 people** |
| **1990** | 22197.46 (19256.54 to 25429.67) | 7.82 (6.82 to 8.92) | 12069.74 (10460.22 to 13880.86) | 9.21 (8.01 to 10.55) | 10127.73 (8779.08 to 11581.12) | 6.62 (5.74 to 7.55) |
| **1991** | 20492.33 (17784.13 to 23438.79) | 7.12 (6.22 to 8.10) | 11091.35 (9624.24 to 12731.73) | 8.36 (7.28 to 9.55) | 9400.99 (8164.68 to 10756.37) | 6.06 (5.26 to 6.92) |
| **1992** | 19134.82 (16673.12 to 21831.96) | 6.55 (5.74 to 7.44) | 10314.05 (8966.52 to 11798.08) | 7.67 (6.70 to 8.74) | 8820.77 (7667.30 to 10107.35) | 5.61 (4.86 to 6.39) |
| **1993** | 18159.98 (15834.85 to 20718.72) | 6.14 (5.37 to 6.98) | 9757.98 (8503.08 to 11168.58) | 7.17 (6.28 to 8.18) | 8401.99 (7306.92 to 9628.08) | 5.27 (4.57 to 6.01) |
| **1994** | 17593.77 (15372.68 to 20126.42) | 5.88 (5.14 to 6.69) | 9435.82 (8234.81 to 10824.29) | 6.85 (6.02 to 7.84) | 8157.95 (7075.61 to 9343.49) | 5.06 (4.40 to 5.76) |
| **1995** | 17524.96 (15339.35 to 20056.53) | 5.79 (5.05 to 6.60) | 9399.17 (8223.41 to 10800.91) | 6.74 (5.91 to 7.72) | 8125.79 (7051.98 to 9305.19) | 4.99 (4.33 to 5.69) |
| **1996** | 18112.89 (15936.25 to 20589.94) | 5.93 (5.19 to 6.75) | 9754.33 (8580.38 to 11162.23) | 6.92 (6.09 to 7.88) | 8358.55 (7282.51 to 9541.02) | 5.09 (4.43 to 5.81) |
| **1997** | 19328.98 (17135.29 to 21891.54) | 6.27 (5.53 to 7.09) | 10492.16 (9313.86 to 11902.66) | 7.35 (6.52 to 8.30) | 8836.82 (7736.96 to 10038.21) | 5.34 (4.68 to 6.08) |
| **1998** | 20864.65 (18607.96 to 23556.80) | 6.71 (5.96 to 7.53) | 11420.07 (10225.42 to 12848.03) | 7.90 (7.06 to 8.84) | 9444.59 (8321.80 to 10696.16) | 5.67 (5.00 to 6.41) |
| **1999** | 22364.04 (20066.33 to 25018.99) | 7.12 (6.38 to 7.94) | 12316.83 (11088.44 to 13734.22) | 8.41 (7.56 to 9.33) | 10047.21 (8914.98 to 11317.82) | 5.98 (5.31 to 6.73) |
| **2000** | 23524.66 (21209.47 to 26201.35) | 7.40 (6.67 to 8.20) | 12994.47 (11762.68 to 14450.90) | 8.74 (7.90 to 9.64) | 10530.20 (9417.94 to 11775.96) | 6.21 (5.55 to 6.96) |
| **2001** | 24447.49 (22325.69 to 26996.13) | 7.58 (6.91 to 8.35) | 13508.14 (12369.60 to 14884.14) | 8.93 (8.16 to 9.81) | 10939.35 (9909.83 to 12111.09) | 6.38 (5.79 to 7.07) |
| **2002** | 25470.66 (23485.54 to 27871.47) | 7.77 (7.16 to 8.49) | 14069.11 (13016.32 to 15331.53) | 9.14 (8.43 to 9.94) | 11401.55 (10470.40 to 12482.73) | 6.56 (6.03 to 7.20) |
| **2003** | 26510.91 (24679.21 to 28740.47) | 7.94 (7.40 to 8.62) | 14641.68 (13671.33 to 15835.73) | 9.32 (8.70 to 10.07) | 11869.23 (10995.20 to 12886.06) | 6.73 (6.23 to 7.33) |
| **2004** | 27437.28 (25757.00 to 29513.30) | 8.08 (7.59 to 8.71) | 15159.61 (14277.57 to 16294.75) | 9.47 (8.91 to 10.18) | 12277.67 (11490.01 to 13223.10) | 6.86 (6.39 to 7.44) |
| **2005** | 28193.48 (26570.42 to 30257.26) | 8.17 (7.71 to 8.78) | 15600.42 (14736.64 to 16712.26) | 9.56 (9.05 to 10.26) | 12593.06 (11798.75 to 13551.74) | 6.94 (6.49 to 7.49) |
| **2006** | 28792.02 (27159.54 to 30850.64) | 8.20 (7.75 to 8.81) | 15961.43 (15082.17 to 17072.60) | 9.61 (9.09 to 10.29) | 12830.60 (12039.31 to 13796.89) | 6.96 (6.53 to 7.51) |
| **2007** | 29425.66 (27776.88 to 31486.32) | 8.24 (7.78 to 8.84) | 16344.30 (15438.05 to 17460.21) | 9.65 (9.13 to 10.33) | 13081.36 (12294.26 to 14061.22) | 6.99 (6.55 to 7.53) |
| **2008** | 30085.95 (28407.17 to 32153.69) | 8.26 (7.81 to 8.87) | 16742.67 (15804.65 to 17854.47) | 9.69 (9.16 to 10.35) | 13343.29 (12551.21 to 14335.72) | 7.00 (6.56 to 7.54) |
| **2009** | 30726.80 (28988.09 to 32807.77) | 8.29 (7.83 to 8.89) | 17128.96 (16156.15 to 18248.14) | 9.72 (9.18 to 10.38) | 13597.84 (12791.60 to 14606.56) | 7.01 (6.58 to 7.55) |
| **2010** | 31399.31 (29585.80 to 33528.44) | 8.31 (7.85 to 8.91) | 17535.04 (16524.46 to 18695.87) | 9.75 (9.20 to 10.40) | 13864.28 (13038.07 to 14890.98) | 7.03 (6.59 to 7.55) |
| **2011** | 32059.62 (30221.82 to 34258.67) | 8.34 (7.87 to 8.94) | 17932.23 (16886.13 to 19131.26) | 9.78 (9.24 to 10.43) | 14127.39 (13283.18 to 15181.50) | 7.04 (6.61 to 7.58) |
| **2012** | 32796.15 (30914.22 to 35086.77) | 8.36 (7.90 to 8.97) | 18370.83 (17278.74 to 19615.74) | 9.82 (9.27 to 10.47) | 14425.32 (13543.92 to 15517.80) | 7.06 (6.63 to 7.61) |
| **2013** | 33569.81 (31635.08 to 35954.77) | 8.39 (7.92 to 9.00) | 18828.31 (17714.86 to 20117.57) | 9.85 (9.31 to 10.52) | 14741.50 (13818.50 to 15882.63) | 7.08 (6.65 to 7.64) |
| **2014** | 34331.68 (32345.26 to 36797.60) | 8.42 (7.95 to 9.04) | 19276.48 (18150.10 to 20612.22) | 9.89 (9.33 to 10.57) | 15055.20 (14090.49 to 16234.71) | 7.10 (6.66 to 7.67) |
| **2015** | 35126.91 (33077.50 to 37669.02) | 8.45 (7.97 to 9.07) | 19744.35 (18599.58 to 21127.95) | 9.93 (9.37 to 10.62) | 15382.56 (14379.94 to 16601.30) | 7.12 (6.67 to 7.70) |
| **2016** | 35966.32 (33846.39 to 38581.08) | 8.50 (8.03 to 9.13) | 20228.30 (19022.84 to 21643.80) | 9.99 (9.43 to 10.69) | 15738.02 (14709.27 to 16973.43) | 7.16 (6.72 to 7.73) |
| **2017** | 36948.18 (34627.18 to 39610.94) | 8.57 (8.09 to 9.21) | 20782.41 (19485.48 to 22202.96) | 10.07 (9.52 to 10.79) | 16165.78 (15084.83 to 17421.59) | 7.22 (6.76 to 7.80) |
| **2018** | 37942.80 (35435.61 to 40723.21) | 8.65 (8.16 to 9.30) | 21343.42 (19981.28 to 22844.81) | 10.14 (9.58 to 10.89) | 16599.38 (15496.71 to 17883.53) | 7.29 (6.81 to 7.87) |
| **2019** | 38830.89 (36232.87 to 41743.42) | 8.69 (8.18 to 9.36) | 21848.17 (20400.73 to 23409.10) | 10.20 (9.61 to 10.94) | 16982.72 (15822.82 to 18277.59) | 7.32 (6.85 to 7.92) |
| **2020** | 39640.08 (37031.44 to 42591.42) | 8.73 (8.23 to 9.39) | 22329.61 (20885.31 to 23998.44) | 10.25 (9.68 to 10.98) | 17310.46 (16148.16 to 18618.18) | 7.34 (6.89 to 7.91) |
| **2021** | 40495.43 (37645.36 to 43576.27) | 8.82 (8.26 to 9.51) | 22755.73 (21262.86 to 24479.88) | 10.33 (9.74 to 11.13) | 17739.70 (16413.50 to 19154.57) | 7.46 (6.96 to 8.05) |
| **Percentage Change,**  **1990-2021** | 82.43% (66.27% to 99.71%) | 12.89% (3.10% to 23.66%) | 88.54% (71.29% to 107.76%) | 12.20% (2.08% to 23.37%) | 75.16% (60.07% to 92.07%) | 12.59% (2.33% to 23.78%) |
| **Statistical Analysis** | Spearman’s r = 0.973 p-value < 0.001 | Spearman’s 𝜌 = 0.953 p-value < 0.001 | Spearman’s r = 0.973 p-value < 0.001 | Spearman’s 𝜌 = 0.953 p-value < 0.001 | Spearman’s r = 0.970 p-value < 0.001 | Spearman’s 𝜌 = 0.950 p-value < 0.001 |
|  |  |  | Wilcoxon rank sum test between sex (count): p-value = 0.003 Wilcoxon rank sum test between sex (rate): p-value < 0.001 | | | |

The numbers in parentheses represent the 95% uncertainty interval.

## Table S2b: Annual age-standardized disability-adjusted life year (DALY) rates and percentage changes from 1990 to 2021, by sex

|  | **Both** | | **Male** | | **Female** | |
| --- | --- | --- | --- | --- | --- | --- |
|  | **Absolute number** | **Age-standardized rate, per 100,000 people** | **Absolute number** | **Age-standardized rate, per 100,000 people** | **Absolute number** | **Age-standardized rate, per 100,000 people** |
| **1990** | 111651.92 (107615.58 to 114636.27) | 39.71 (38.48 to 40.72) | 60845.02 (59151.86 to 62401.79) | 46.99 (45.72 to 48.22) | 50806.89 (48448.88 to 52382.55) | 33.49 (32.28 to 34.43) |
| **1991** | 114748.08 (110416.75 to 117735.35) | 40.35 (39.05 to 41.33) | 62664.18 (60933.00 to 64188.86) | 47.68 (46.38 to 48.91) | 52083.89 (49489.18 to 53698.95) | 34.12 (32.81 to 35.09) |
| **1992** | 115140.59 (111114.08 to 118104.08) | 39.94 (38.76 to 40.90) | 62955.82 (61305.48 to 64404.58) | 47.23 (46.02 to 48.33) | 52184.76 (49758.42 to 53836.18) | 33.75 (32.53 to 34.73) |
| **1993** | 115849.11 (111980.90 to 118540.05) | 39.81 (38.69 to 40.63) | 63032.14 (61481.05 to 64401.39) | 46.72 (45.60 to 47.73) | 52816.97 (50548.98 to 54331.10) | 33.96 (32.81 to 34.82) |
| **1994** | 119236.35 (115567.27 to 122091.42) | 40.54 (39.46 to 41.45) | 64857.25 (63282.91 to 66223.22) | 47.49 (46.40 to 48.46) | 54379.10 (52108.87 to 55980.91) | 34.66 (33.55 to 35.54) |
| **1995** | 122322.98 (118488.02 to 125156.28) | 41.03 (39.92 to 41.90) | 66908.14 (65350.53 to 68245.91) | 48.21 (47.11 to 49.17) | 55414.84 (53009.68 to 57071.80) | 34.86 (33.68 to 35.74) |
| **1996** | 124202.10 (120501.73 to 127022.65) | 41.14 (40.03 to 41.99) | 67149.26 (65687.57 to 68555.13) | 47.64 (46.64 to 48.65) | 57052.85 (54692.72 to 58726.13) | 35.61 (34.46 to 36.55) |
| **1997** | 125681.50 (121579.08 to 128709.26) | 41.02 (39.80 to 41.93) | 68435.01 (66797.14 to 69965.64) | 47.77 (46.64 to 48.87) | 57246.50 (54807.76 to 58989.19) | 35.20 (34.07 to 36.14) |
| **1998** | 130132.04 (125521.71 to 133392.10) | 41.65 (40.41 to 42.62) | 70744.61 (68877.79 to 72356.17) | 48.53 (47.29 to 49.65) | 59387.43 (56518.82 to 61217.07) | 35.66 (34.38 to 36.67) |
| **1999** | 141648.68 (136569.14 to 145133.83) | 43.91 (42.56 to 44.95) | 78143.39 (76038.55 to 79915.26) | 52.12 (50.75 to 53.28) | 63505.29 (60457.17 to 65416.43) | 36.81 (35.48 to 37.79) |
| **2000** | 151744.27 (146345.16 to 155539.54) | 46.08 (44.62 to 47.20) | 84424.26 (82141.60 to 86379.64) | 55.09 (53.68 to 56.32) | 67320.00 (64098.70 to 69402.72) | 38.28 (36.89 to 39.35) |
| **2001** | 155220.15 (149461.35 to 159095.82) | 46.43 (44.98 to 47.52) | 87163.18 (84749.88 to 89188.95) | 56.00 (54.43 to 57.32) | 68056.96 (64654.39 to 70197.61) | 38.12 (36.68 to 39.17) |
| **2002** | 162058.38 (156135.01 to 166136.62) | 47.71 (46.18 to 48.82) | 90390.26 (87938.33 to 92563.50) | 57.02 (55.46 to 58.33) | 71668.12 (67906.37 to 73961.39) | 39.53 (37.89 to 40.63) |
| **2003** | 166209.96 (160094.05 to 170471.57) | 48.11 (46.51 to 49.24) | 93627.75 (91144.23 to 95787.86) | 57.87 (56.35 to 59.16) | 72582.20 (68806.46 to 74992.58) | 39.43 (37.76 to 40.60) |
| **2004** | 165361.42 (158974.56 to 169610.75) | 47.11 (45.47 to 48.24) | 94220.47 (91396.86 to 96458.88) | 57.18 (55.53 to 58.52) | 71140.95 (67445.36 to 73548.07) | 38.10 (36.50 to 39.22) |
| **2005** | 169298.74 (162895.63 to 173607.56) | 47.25 (45.70 to 48.42) | 96835.98 (94128.67 to 99108.87) | 57.53 (55.95 to 58.87) | 72462.76 (68616.72 to 74954.65) | 38.04 (36.36 to 39.15) |
| **2006** | 172622.74 (165578.66 to 177131.07) | 47.29 (45.57 to 48.44) | 98470.15 (95418.10 to 100889.98) | 57.24 (55.56 to 58.62) | 74152.60 (69945.89 to 76649.82) | 38.41 (36.67 to 39.55) |
| **2007** | 176294.28 (168819.71 to 181059.91) | 47.42 (45.65 to 48.64) | 98777.42 (95627.78 to 101270.83) | 56.33 (54.60 to 57.74) | 77516.86 (73046.42 to 80164.21) | 39.42 (37.57 to 40.63) |
| **2008** | 181450.76 (173641.34 to 186413.02) | 47.84 (46.04 to 49.02) | 102140.47 (98960.39 to 104734.79) | 56.99 (55.29 to 58.38) | 79310.28 (74789.36 to 81982.22) | 39.60 (37.72 to 40.77) |
| **2009** | 187365.67 (178995.34 to 192464.81) | 48.38 (46.57 to 49.62) | 105675.68 (102519.84 to 108248.79) | 57.76 (56.16 to 59.11) | 81689.99 (76802.60 to 84449.66) | 39.88 (37.94 to 41.11) |
| **2010** | 192222.70 (183288.26 to 197608.12) | 48.61 (46.66 to 49.88) | 108086.46 (104314.95 to 110878.12) | 57.77 (55.89 to 59.15) | 84136.24 (78820.72 to 87130.75) | 40.32 (38.28 to 41.55) |
| **2011** | 197646.58 (188197.62 to 202956.41) | 48.88 (46.91 to 50.16) | 110902.78 (107218.07 to 113569.38) | 57.89 (56.12 to 59.21) | 86743.80 (81546.19 to 89704.53) | 40.72 (38.70 to 41.92) |
| **2012** | 202348.90 (192383.19 to 207958.90) | 48.91 (46.84 to 50.16) | 114058.10 (110240.25 to 116804.58) | 58.11 (56.23 to 59.43) | 88290.80 (82360.42 to 91400.31) | 40.59 (38.30 to 41.83) |
| **2013** | 202571.11 (192222.32 to 208093.74) | 47.79 (45.66 to 48.97) | 114679.96 (110455.76 to 117389.98) | 56.97 (54.98 to 58.27) | 87891.14 (82154.15 to 90767.56) | 39.48 (37.37 to 40.66) |
| **2014** | 204990.32 (194413.33 to 210633.97) | 47.11 (44.97 to 48.31) | 116326.57 (111900.90 to 119139.61) | 56.45 (54.48 to 57.76) | 88663.75 (83193.70 to 91690.76) | 38.64 (36.61 to 39.84) |
| **2015** | 206672.35 (196067.81 to 212016.82) | 46.52 (44.45 to 47.63) | 117376.55 (112576.98 to 120024.86) | 55.81 (53.79 to 57.02) | 89295.80 (83628.49 to 92233.90) | 38.04 (36.00 to 39.20) |
| **2016** | 202917.78 (192903.95 to 208204.93) | 44.77 (42.93 to 45.83) | 115737.94 (111258.63 to 118676.75) | 53.85 (51.98 to 55.10) | 87179.84 (81754.11 to 90020.16) | 36.46 (34.58 to 37.53) |
| **2017** | 202043.02 (192044.13 to 207467.23) | 43.33 (41.47 to 44.39) | 115305.42 (110796.59 to 118242.81) | 52.07 (50.23 to 53.36) | 86737.60 (81161.44 to 89826.15) | 35.32 (33.46 to 36.41) |
| **2018** | 200724.76 (190961.06 to 206233.18) | 41.92 (40.13 to 43.02) | 114085.06 (109488.58 to 117152.50) | 50.14 (48.32 to 51.45) | 86639.70 (80694.43 to 89768.26) | 34.42 (32.54 to 35.56) |
| **2019** | 202826.77 (193035.99 to 208799.43) | 41.47 (39.77 to 42.64) | 115748.11 (111213.37 to 118991.19) | 49.97 (48.23 to 51.38) | 87078.66 (80972.88 to 90337.59) | 33.69 (31.78 to 34.82) |
| **2020** | 205587.93 (195437.15 to 212840.48) | 41.17 (39.44 to 42.55) | 117439.72 (112874.98 to 121448.40) | 49.75 (47.92 to 51.42) | 88148.21 (81657.79 to 91754.28) | 33.32 (31.30 to 34.57) |
| **2021** | 210559.45 (199250.06 to 218996.64) | 41.36 (39.47 to 42.94) | 120100.60 (114052.88 to 125007.03) | 50.01 (47.66 to 51.99) | 90458.85 (83917.83 to 95383.87) | 33.45 (31.52 to 34.99) |
| **Percentage Change,**  **1990-2021** | 88.59% (81.68% to 95.19%) | 4.14% (0.41% to 7.68%) | 97.39% (88.23% to 105.44%) | 6.43% (1.68% to 10.69%) | 78.04% (70.86% to 85.98%) | -0.12% (-3.73% to 3.94%) |
| **Statistical Analysis** | Spearman’s 𝜌 = 0.979 p-value < 0.001 | Spearman’s 𝜌 = 0.459 p-value = 0.009 | Spearman’s 𝜌 = 0.988 p-value < 0.001 | Spearman’s 𝜌 = 0.451 p-value = 0.010 | Spearman’s 𝜌 = 0.960 p-value < 0.001 | Spearman’s 𝜌 = 0.170 p-value = 0.352 |
|  |  |  | Wilcoxon rank sum test between sex (count): p-value < 0.001 Wilcoxon rank sum test between sex (rate): p-value < 0.001 | | | |

The numbers in parentheses represent the 95% uncertainty interval.

## Table S2c: Annual age-standardized mortality rates and percentage changes from 1990 to 2021, by sex

|  | **Both** | | **Male** | | **Female** | |
| --- | --- | --- | --- | --- | --- | --- |
|  | **Absolute number** | **Age-standardized rate, per 100,000 people** | **Absolute number** | **Age-standardized rate, per 100,000 people** | **Absolute number** | **Age-standardized rate, per 100,000 people** |
| **1990** | 3890.20 (3686.90 to 4008.21) | 1.26 (1.20 to 1.29) | 2034.89 (1963.03 to 2087.27) | 1.52 (1.46 to 1.56) | 1855.31 (1723.94 to 1928.40) | 1.06 (1.00 to 1.10) |
| **1991** | 4033.33 (3812.24 to 4162.46) | 1.29 (1.22 to 1.32) | 2119.26 (2041.83 to 2176.00) | 1.55 (1.50 to 1.60) | 1914.08 (1777.71 to 1988.89) | 1.08 (1.02 to 1.12) |
| **1992** | 4090.25 (3874.48 to 4215.99) | 1.28 (1.22 to 1.32) | 2151.89 (2077.08 to 2205.76) | 1.55 (1.50 to 1.59) | 1938.36 (1800.75 to 2016.57) | 1.08 (1.02 to 1.12) |
| **1993** | 4132.89 (3926.17 to 4257.04) | 1.28 (1.23 to 1.32) | 2169.82 (2093.85 to 2222.79) | 1.54 (1.49 to 1.58) | 1963.07 (1825.92 to 2037.08) | 1.09 (1.02 to 1.12) |
| **1994** | 4297.23 (4081.50 to 4420.17) | 1.32 (1.26 to 1.35) | 2253.20 (2175.93 to 2309.62) | 1.58 (1.53 to 1.62) | 2044.03 (1900.63 to 2123.24) | 1.12 (1.06 to 1.16) |
| **1995** | 4421.14 (4200.69 to 4548.04) | 1.34 (1.28 to 1.37) | 2318.77 (2236.98 to 2373.51) | 1.60 (1.55 to 1.64) | 2102.37 (1950.34 to 2187.21) | 1.13 (1.07 to 1.17) |
| **1996** | 4520.15 (4286.28 to 4653.26) | 1.35 (1.29 to 1.39) | 2351.14 (2269.28 to 2409.06) | 1.60 (1.54 to 1.64) | 2169.01 (2012.46 to 2254.29) | 1.16 (1.09 to 1.20) |
| **1997** | 4585.48 (4347.06 to 4719.99) | 1.35 (1.29 to 1.39) | 2394.09 (2304.49 to 2450.36) | 1.60 (1.54 to 1.64) | 2191.39 (2027.62 to 2278.71) | 1.15 (1.09 to 1.19) |
| **1998** | 4802.08 (4545.67 to 4950.44) | 1.39 (1.32 to 1.43) | 2495.48 (2400.47 to 2557.57) | 1.64 (1.58 to 1.68) | 2306.61 (2128.18 to 2401.83) | 1.19 (1.12 to 1.23) |
| **1999** | 5401.04 (5095.36 to 5565.92) | 1.52 (1.44 to 1.56) | 2849.25 (2742.47 to 2920.01) | 1.83 (1.76 to 1.88) | 2551.79 (2345.15 to 2650.95) | 1.28 (1.19 to 1.32) |
| **2000** | 5839.28 (5504.35 to 6026.49) | 1.61 (1.53 to 1.66) | 3110.99 (2991.90 to 3189.40) | 1.96 (1.88 to 2.01) | 2728.29 (2508.72 to 2842.84) | 1.35 (1.26 to 1.39) |
| **2001** | 6003.77 (5665.22 to 6189.36) | 1.64 (1.56 to 1.68) | 3231.74 (3108.74 to 3313.33) | 2.00 (1.93 to 2.05) | 2772.03 (2548.57 to 2886.75) | 1.35 (1.26 to 1.40) |
| **2002** | 6256.27 (5896.01 to 6459.14) | 1.68 (1.60 to 1.73) | 3328.66 (3199.17 to 3411.92) | 2.03 (1.95 to 2.08) | 2927.62 (2681.34 to 3051.60) | 1.41 (1.31 to 1.46) |
| **2003** | 6386.44 (6022.94 to 6591.26) | 1.69 (1.60 to 1.74) | 3419.80 (3287.39 to 3502.70) | 2.04 (1.96 to 2.09) | 2966.64 (2723.45 to 3093.92) | 1.40 (1.31 to 1.46) |
| **2004** | 6305.84 (5944.84 to 6511.15) | 1.64 (1.56 to 1.69) | 3417.17 (3281.98 to 3506.86) | 2.00 (1.92 to 2.05) | 2888.67 (2649.80 to 3015.23) | 1.35 (1.26 to 1.40) |
| **2005** | 6472.31 (6091.90 to 6696.20) | 1.65 (1.56 to 1.70) | 3531.82 (3385.89 to 3621.71) | 2.02 (1.94 to 2.07) | 2940.50 (2687.61 to 3072.97) | 1.35 (1.25 to 1.40) |
| **2006** | 6587.55 (6174.94 to 6809.41) | 1.65 (1.56 to 1.70) | 3601.28 (3438.73 to 3704.46) | 2.02 (1.93 to 2.08) | 2986.27 (2724.60 to 3124.19) | 1.35 (1.25 to 1.41) |
| **2007** | 6723.24 (6289.78 to 6968.81) | 1.65 (1.56 to 1.71) | 3603.83 (3426.54 to 3711.05) | 1.97 (1.88 to 2.03) | 3119.41 (2844.12 to 3268.47) | 1.39 (1.29 to 1.45) |
| **2008** | 6900.71 (6454.15 to 7159.24) | 1.66 (1.56 to 1.72) | 3721.60 (3544.60 to 3830.25) | 1.99 (1.90 to 2.05) | 3179.11 (2895.04 to 3330.09) | 1.39 (1.28 to 1.45) |
| **2009** | 7131.92 (6673.71 to 7397.33) | 1.68 (1.58 to 1.74) | 3856.29 (3665.24 to 3971.58) | 2.01 (1.91 to 2.07) | 3275.64 (2984.10 to 3430.21) | 1.40 (1.30 to 1.46) |
| **2010** | 7365.13 (6875.61 to 7637.73) | 1.70 (1.59 to 1.75) | 3978.55 (3777.90 to 4097.09) | 2.02 (1.92 to 2.08) | 3386.58 (3071.85 to 3552.91) | 1.42 (1.31 to 1.48) |
| **2011** | 7630.62 (7116.42 to 7906.62) | 1.72 (1.61 to 1.78) | 4117.39 (3906.23 to 4239.45) | 2.04 (1.94 to 2.10) | 3513.23 (3182.25 to 3676.40) | 1.45 (1.33 to 1.51) |
| **2012** | 7879.97 (7338.43 to 8172.46) | 1.73 (1.62 to 1.79) | 4284.99 (4068.84 to 4415.39) | 2.07 (1.97 to 2.13) | 3594.98 (3254.75 to 3764.99) | 1.44 (1.32 to 1.51) |
| **2013** | 7906.21 (7354.85 to 8197.84) | 1.69 (1.58 to 1.75) | 4327.92 (4105.51 to 4457.37) | 2.03 (1.93 to 2.09) | 3578.29 (3225.68 to 3748.53) | 1.40 (1.28 to 1.46) |
| **2014** | 8044.95 (7464.91 to 8344.65) | 1.68 (1.56 to 1.73) | 4413.05 (4178.90 to 4547.09) | 2.02 (1.91 to 2.08) | 3631.90 (3278.62 to 3802.26) | 1.39 (1.27 to 1.44) |
| **2015** | 8093.36 (7526.64 to 8387.22) | 1.65 (1.54 to 1.70) | 4448.72 (4201.68 to 4585.22) | 1.98 (1.87 to 2.04) | 3644.65 (3286.34 to 3817.40) | 1.36 (1.24 to 1.42) |
| **2016** | 7858.42 (7328.64 to 8147.55) | 1.56 (1.47 to 1.62) | 4345.55 (4113.75 to 4486.48) | 1.89 (1.79 to 1.95) | 3512.87 (3178.02 to 3673.91) | 1.29 (1.18 to 1.34) |
| **2017** | 7856.22 (7311.31 to 8137.31) | 1.52 (1.43 to 1.57) | 4348.36 (4114.63 to 4488.93) | 1.83 (1.74 to 1.89) | 3507.86 (3173.96 to 3669.03) | 1.25 (1.15 to 1.30) |
| **2018** | 7858.47 (7300.15 to 8145.85) | 1.48 (1.39 to 1.53) | 4345.03 (4120.55 to 4481.72) | 1.78 (1.69 to 1.83) | 3513.44 (3161.47 to 3684.44) | 1.22 (1.11 to 1.28) |
| **2019** | 7998.62 (7414.53 to 8295.12) | 1.47 (1.37 to 1.52) | 4442.67 (4223.00 to 4585.82) | 1.78 (1.69 to 1.83) | 3555.95 (3196.89 to 3729.15) | 1.21 (1.10 to 1.26) |
| **2020** | 8170.70 (7546.51 to 8521.98) | 1.47 (1.37 to 1.53) | 4544.20 (4311.03 to 4718.72) | 1.78 (1.69 to 1.84) | 3626.50 (3247.87 to 3838.26) | 1.20 (1.09 to 1.26) |
| **2021** | 8465.56 (7799.92 to 8898.99) | 1.49 (1.38 to 1.56) | 4705.32 (4440.84 to 4912.11) | 1.80 (1.71 to 1.88) | 3760.24 (3366.24 to 4020.17) | 1.21 (1.10 to 1.29) |
| **Percentage Change,**  **1990-2021** | 117.61% (109.05% to 125.82%) | 18.34% (13.86% to 22.70%) | 131.23% (120.09% to 141.17%) | 19.01% (13.35% to 24.06%) | 102.67% (92.93% to 114.14%) | 14.50% (9.28% to 20.47%) |
| **Statistical Analysis** | Spearman’s 𝜌 = 0.981 p-value < 0.001 | Spearman’s 𝜌 = 0.510 p-value = 0.0032 | Spearman’s 𝜌 = 0.992 p-value < 0.001 | Spearman’s 𝜌 = 0.474 p-value = 0.007 | Spearman’s 𝜌 = 0.966 p-value < 0.001 | Spearman’s 𝜌 = 0.493 p-value = 0.005 |
|  |  |  | Wilcoxon rank sum test between sex (count): p-value = 0.009 Wilcoxon rank sum test between sex (rate): p-value < 0.001 | | | |

The numbers in parentheses represent the 95% uncertainty interval.

## Table S2d: Annual age-standardized incidence rates and percentage changes from 1990 to 2021, by sex

|  | **Both** | | **Male** | | **Female** | |
| --- | --- | --- | --- | --- | --- | --- |
|  | **Absolute number** | **Age-standardized rate, per 100,000 people** | **Absolute number** | **Age-standardized rate, per 100,000 people** | **Absolute number** | **Age-standardized rate, per 100,000 people** |
| **1990** | 5280.94 (5003.51 to 5603.93) | 1.79 (1.69 to 1.91) | 2804.43 (2652.60 to 2979.83) | 2.13 (2.01 to 2.26) | 2476.51 (2338.94 to 2627.23) | 1.52 (1.43 to 1.62) |
| **1991** | 5341.30 (5069.87 to 5649.64) | 1.78 (1.68 to 1.89) | 2838.26 (2695.18 to 3006.34) | 2.12 (2.01 to 2.24) | 2503.04 (2371.41 to 2650.88) | 1.51 (1.42 to 1.60) |
| **1992** | 5411.27 (5148.42 to 5705.87) | 1.78 (1.69 to 1.88) | 2877.56 (2739.72 to 3042.94) | 2.12 (2.01 to 2.23) | 2533.71 (2404.39 to 2676.67) | 1.50 (1.42 to 1.59) |
| **1993** | 5490.83 (5243.52 to 5773.01) | 1.77 (1.69 to 1.87) | 2922.82 (2788.01 to 3082.17) | 2.12 (2.02 to 2.23) | 2568.01 (2441.17 to 2705.61) | 1.50 (1.42 to 1.59) |
| **1994** | 5571.26 (5321.34 to 5849.51) | 1.78 (1.69 to 1.87) | 2968.65 (2831.17 to 3124.80) | 2.12 (2.02 to 2.23) | 2602.60 (2473.39 to 2738.06) | 1.50 (1.42 to 1.58) |
| **1995** | 5670.81 (5417.54 to 5948.95) | 1.78 (1.70 to 1.88) | 3025.78 (2889.73 to 3186.75) | 2.13 (2.03 to 2.24) | 2645.03 (2512.64 to 2777.14) | 1.51 (1.43 to 1.59) |
| **1996** | 5786.35 (5529.31 to 6068.87) | 1.80 (1.71 to 1.89) | 3091.78 (2958.31 to 3249.41) | 2.15 (2.05 to 2.25) | 2694.57 (2562.13 to 2828.23) | 1.52 (1.44 to 1.60) |
| **1997** | 5931.08 (5670.01 to 6220.77) | 1.82 (1.73 to 1.91) | 3174.22 (3040.63 to 3331.97) | 2.17 (2.07 to 2.27) | 2756.86 (2617.21 to 2891.56) | 1.53 (1.45 to 1.61) |
| **1998** | 6095.06 (5822.18 to 6391.59) | 1.84 (1.75 to 1.93) | 3268.14 (3128.17 to 3429.89) | 2.19 (2.09 to 2.30) | 2826.91 (2684.82 to 2966.63) | 1.55 (1.47 to 1.63) |
| **1999** | 6253.06 (5975.46 to 6550.65) | 1.86 (1.77 to 1.95) | 3360.21 (3215.48 to 3522.49) | 2.21 (2.12 to 2.32) | 2892.84 (2750.93 to 3035.34) | 1.56 (1.48 to 1.64) |
| **2000** | 6410.34 (6129.91 to 6707.38) | 1.87 (1.79 to 1.96) | 3454.59 (3305.19 to 3620.02) | 2.23 (2.14 to 2.34) | 2955.75 (2812.49 to 3098.10) | 1.57 (1.49 to 1.65) |
| **2001** | 6560.98 (6280.86 to 6860.34) | 1.89 (1.80 to 1.97) | 3547.26 (3398.14 to 3711.83) | 2.25 (2.16 to 2.35) | 3013.72 (2871.88 to 3156.87) | 1.58 (1.51 to 1.66) |
| **2002** | 6735.47 (6454.11 to 7034.83) | 1.90 (1.82 to 1.98) | 3654.63 (3509.99 to 3820.26) | 2.27 (2.19 to 2.37) | 3080.85 (2939.33 to 3225.09) | 1.59 (1.52 to 1.67) |
| **2003** | 6929.36 (6649.25 to 7233.61) | 1.91 (1.84 to 2.00) | 3773.27 (3632.36 to 3940.20) | 2.29 (2.21 to 2.39) | 3156.10 (3015.86 to 3298.51) | 1.60 (1.53 to 1.68) |
| **2004** | 7120.44 (6845.76 to 7420.97) | 1.93 (1.86 to 2.01) | 3890.11 (3748.98 to 4050.87) | 2.31 (2.23 to 2.40) | 3230.34 (3088.82 to 3373.16) | 1.61 (1.54 to 1.68) |
| **2005** | 7319.88 (7038.19 to 7624.94) | 1.94 (1.87 to 2.02) | 4011.21 (3867.46 to 4172.44) | 2.33 (2.25 to 2.42) | 3308.67 (3165.20 to 3453.06) | 1.62 (1.55 to 1.69) |
| **2006** | 7515.51 (7225.70 to 7821.48) | 1.96 (1.89 to 2.03) | 4128.25 (3983.59 to 4290.89) | 2.35 (2.27 to 2.44) | 3387.26 (3244.71 to 3534.84) | 1.63 (1.56 to 1.70) |
| **2007** | 7741.41 (7445.17 to 8048.09) | 1.97 (1.90 to 2.05) | 4261.16 (4114.13 to 4424.45) | 2.37 (2.29 to 2.45) | 3480.25 (3334.96 to 3628.39) | 1.64 (1.57 to 1.71) |
| **2008** | 7980.78 (7679.37 to 8294.83) | 1.99 (1.92 to 2.06) | 4400.97 (4248.04 to 4563.87) | 2.38 (2.31 to 2.47) | 3579.81 (3430.82 to 3728.07) | 1.65 (1.58 to 1.72) |
| **2009** | 8206.55 (7895.94 to 8516.36) | 2.00 (1.93 to 2.07) | 4532.81 (4378.16 to 4701.45) | 2.40 (2.33 to 2.48) | 3673.74 (3522.07 to 3825.85) | 1.65 (1.59 to 1.73) |
| **2010** | 8435.04 (8114.58 to 8749.56) | 2.01 (1.94 to 2.08) | 4667.18 (4505.86 to 4840.90) | 2.41 (2.34 to 2.49) | 3767.86 (3616.68 to 3923.20) | 1.66 (1.60 to 1.73) |
| **2011** | 8650.32 (8329.01 to 8974.39) | 2.02 (1.95 to 2.09) | 4793.41 (4629.88 to 4972.40) | 2.42 (2.35 to 2.50) | 3856.91 (3706.00 to 4012.48) | 1.67 (1.60 to 1.74) |
| **2012** | 8892.25 (8570.66 to 9221.43) | 2.02 (1.96 to 2.10) | 4932.75 (4766.59 to 5111.90) | 2.43 (2.35 to 2.51) | 3959.50 (3808.95 to 4114.64) | 1.67 (1.61 to 1.74) |
| **2013** | 9145.44 (8824.65 to 9482.25) | 2.03 (1.96 to 2.10) | 5077.83 (4906.63 to 5263.07) | 2.43 (2.36 to 2.52) | 4067.61 (3916.77 to 4225.67) | 1.68 (1.61 to 1.74) |
| **2014** | 9389.51 (9063.74 to 9734.74) | 2.03 (1.97 to 2.11) | 5216.48 (5045.99 to 5400.27) | 2.44 (2.36 to 2.53) | 4173.03 (4018.36 to 4335.43) | 1.68 (1.62 to 1.75) |
| **2015** | 9641.12 (9304.70 to 9997.12) | 2.04 (1.97 to 2.11) | 5359.80 (5182.76 to 5549.18) | 2.45 (2.37 to 2.53) | 4281.33 (4121.51 to 4448.46) | 1.68 (1.62 to 1.75) |
| **2016** | 9877.93 (9518.43 to 10245.00) | 2.04 (1.97 to 2.12) | 5493.04 (5310.83 to 5691.19) | 2.45 (2.37 to 2.54) | 4384.88 (4216.60 to 4560.58) | 1.69 (1.62 to 1.75) |
| **2017** | 10133.52 (9757.59 to 10521.95) | 2.04 (1.97 to 2.12) | 5635.62 (5437.26 to 5848.92) | 2.45 (2.37 to 2.54) | 4497.90 (4323.88 to 4681.17) | 1.69 (1.62 to 1.76) |
| **2018** | 10392.51 (9988.90 to 10810.74) | 2.05 (1.97 to 2.13) | 5779.32 (5565.92 to 6004.00) | 2.45 (2.37 to 2.55) | 4613.19 (4428.70 to 4806.86) | 1.69 (1.62 to 1.76) |
| **2019** | 10640.30 (10202.52 to 11087.15) | 2.05 (1.97 to 2.13) | 5915.36 (5688.02 to 6155.14) | 2.46 (2.36 to 2.55) | 4724.94 (4529.69 to 4928.48) | 1.69 (1.62 to 1.76) |
| **2020** | 10868.76 (10436.04 to 11319.27) | 2.05 (1.97 to 2.13) | 6039.46 (5809.38 to 6280.59) | 2.46 (2.36 to 2.55) | 4829.29 (4630.40 to 5039.40) | 1.69 (1.62 to 1.77) |
| **2021** | 11089.01 (10633.99 to 11559.06) | 2.06 (1.98 to 2.14) | 6154.30 (5915.24 to 6410.01) | 2.46 (2.37 to 2.57) | 4934.71 (4719.20 to 5152.02) | 1.70 (1.63 to 1.78) |
| **Percentage Change,**  **1990-2021** | 109.98% (103.15% to 117.25%) | 15.03% (11.49% to 19.04%) | 119.45% (110.81% to 127.89%) | 15.92% (12.18% to 19.91%) | 99.26% (93.25% to 105.90%) | 11.93% (8.42% to 15.89%) |
| **Statistical Analysis** | Spearman’s 𝜌 = 1.000 p-value < 0.001 | Spearman’s 𝜌 = 0.953 p-value < 0.001 | Spearman’s 𝜌 = 1.000 p-value < 0.001 | Spearman’s 𝜌 = 0.953 p-value < 0.001 | Spearman’s 𝜌 = 1.000 p-value < 0.001 | Spearman’s 𝜌 = 0.987 p-value < 0.001 |
|  |  |  | Wilcoxon rank sum test between sex (count): p-value = 0.005 Wilcoxon rank sum test between sex (rate): p-value < 0.001 | | | |

The numbers in parentheses represent the 95% uncertainty interval.

## Table S2e: Annual age-standardized years lived with disability (YLD) rates and percentage changes from 1990 to 2021, by sex

|  | **Both** | | **Male** | | **Female** | |
| --- | --- | --- | --- | --- | --- | --- |
|  | **Absolute number** | **Age-standardized rate, per 100,000 people** | **Absolute number** | **Age-standardized rate, per 100,000 people** | **Absolute number** | **Age-standardized rate, per 100,000 people** |
| **1990** | 4717.00 (3366.69 to 6414.58) | 1.66 (1.18 to 2.24) | 2565.14 (1831.97 to 3463.58) | 1.96 (1.40 to 2.63) | 2151.86 (1528.41 to 2916.58) | 1.41 (1.00 to 1.89) |
| **1991** | 4354.53 (3104.96 to 5933.89) | 1.51 (1.08 to 2.04) | 2357.12 (1682.01 to 3197.56) | 1.78 (1.27 to 2.40) | 1997.41 (1421.55 to 2705.47) | 1.29 (0.91 to 1.73) |
| **1992** | 4065.93 (2898.06 to 5536.69) | 1.39 (0.99 to 1.88) | 2191.83 (1565.10 to 2973.13) | 1.63 (1.17 to 2.21) | 1874.10 (1332.18 to 2539.22) | 1.19 (0.85 to 1.61) |
| **1993** | 3858.68 (2755.05 to 5234.00) | 1.30 (0.93 to 1.76) | 2073.58 (1483.35 to 2811.22) | 1.52 (1.09 to 2.06) | 1785.11 (1271.42 to 2409.49) | 1.12 (0.80 to 1.51) |
| **1994** | 3738.30 (2676.61 to 5054.69) | 1.25 (0.90 to 1.69) | 2005.05 (1433.27 to 2720.34) | 1.46 (1.04 to 1.97) | 1733.25 (1240.43 to 2330.05) | 1.08 (0.77 to 1.46) |
| **1995** | 3723.64 (2672.30 to 5023.17) | 1.23 (0.89 to 1.66) | 1997.22 (1427.08 to 2709.32) | 1.43 (1.03 to 1.93) | 1726.42 (1239.53 to 2315.48) | 1.06 (0.76 to 1.43) |
| **1996** | 3848.62 (2756.88 to 5189.86) | 1.26 (0.91 to 1.70) | 2072.72 (1482.98 to 2809.55) | 1.47 (1.06 to 1.99) | 1775.89 (1273.72 to 2377.23) | 1.08 (0.78 to 1.46) |
| **1997** | 4107.13 (2944.74 to 5537.08) | 1.33 (0.96 to 1.80) | 2229.57 (1596.10 to 3011.84) | 1.56 (1.12 to 2.11) | 1877.55 (1344.64 to 2512.94) | 1.14 (0.82 to 1.54) |
| **1998** | 4433.52 (3182.12 to 5965.29) | 1.43 (1.03 to 1.92) | 2426.78 (1738.47 to 3267.52) | 1.68 (1.21 to 2.26) | 2006.74 (1440.02 to 2685.03) | 1.20 (0.87 to 1.63) |
| **1999** | 4752.25 (3406.97 to 6380.68) | 1.51 (1.09 to 2.03) | 2617.41 (1877.17 to 3508.21) | 1.79 (1.29 to 2.40) | 2134.84 (1535.83 to 2860.83) | 1.27 (0.92 to 1.71) |
| **2000** | 4998.91 (3591.24 to 6689.40) | 1.57 (1.13 to 2.10) | 2761.39 (1985.40 to 3688.70) | 1.86 (1.34 to 2.48) | 2237.52 (1611.83 to 2999.67) | 1.32 (0.96 to 1.77) |
| **2001** | 5194.96 (3743.60 to 6929.31) | 1.61 (1.16 to 2.15) | 2870.48 (2071.18 to 3817.45) | 1.90 (1.37 to 2.53) | 2324.47 (1672.33 to 3099.01) | 1.35 (0.98 to 1.81) |
| **2002** | 5412.26 (3891.62 to 7149.77) | 1.65 (1.19 to 2.18) | 2989.53 (2147.04 to 3955.93) | 1.94 (1.40 to 2.56) | 2422.73 (1743.85 to 3208.04) | 1.39 (1.01 to 1.84) |
| **2003** | 5633.17 (4062.01 to 7407.11) | 1.69 (1.22 to 2.21) | 3111.02 (2243.96 to 4094.57) | 1.98 (1.43 to 2.60) | 2522.15 (1818.99 to 3318.07) | 1.43 (1.04 to 1.88) |
| **2004** | 5829.93 (4200.09 to 7648.23) | 1.72 (1.25 to 2.24) | 3220.96 (2328.96 to 4233.12) | 2.01 (1.46 to 2.63) | 2608.98 (1880.46 to 3421.45) | 1.46 (1.06 to 1.90) |
| **2005** | 5990.16 (4312.74 to 7875.58) | 1.73 (1.26 to 2.27) | 3314.16 (2393.21 to 4367.57) | 2.03 (1.47 to 2.66) | 2676.00 (1931.66 to 3508.21) | 1.47 (1.07 to 1.92) |
| **2006** | 6116.93 (4406.47 to 8041.79) | 1.74 (1.26 to 2.28) | 3390.47 (2446.15 to 4468.18) | 2.04 (1.48 to 2.68) | 2726.47 (1968.37 to 3574.09) | 1.48 (1.07 to 1.93) |
| **2007** | 6251.55 (4510.15 to 8216.53) | 1.75 (1.27 to 2.29) | 3471.81 (2503.25 to 4573.14) | 2.05 (1.48 to 2.69) | 2779.74 (2007.60 to 3643.36) | 1.48 (1.08 to 1.94) |
| **2008** | 6391.57 (4617.15 to 8395.50) | 1.76 (1.27 to 2.30) | 3556.19 (2571.48 to 4676.19) | 2.06 (1.49 to 2.70) | 2835.38 (2048.58 to 3716.18) | 1.49 (1.08 to 1.94) |
| **2009** | 6527.82 (4721.35 to 8580.93) | 1.76 (1.27 to 2.30) | 3638.39 (2633.98 to 4784.51) | 2.06 (1.49 to 2.71) | 2889.44 (2088.20 to 3788.05) | 1.49 (1.08 to 1.95) |
| **2010** | 6670.58 (4830.43 to 8766.03) | 1.77 (1.28 to 2.31) | 3724.56 (2701.40 to 4889.29) | 2.07 (1.50 to 2.72) | 2946.01 (2129.74 to 3863.12) | 1.49 (1.08 to 1.95) |
| **2011** | 6810.55 (4938.84 to 8947.26) | 1.77 (1.28 to 2.32) | 3808.65 (2765.01 to 4998.21) | 2.08 (1.50 to 2.73) | 3001.91 (2174.33 to 3938.64) | 1.50 (1.08 to 1.96) |
| **2012** | 6966.68 (5063.29 to 9152.24) | 1.78 (1.29 to 2.33) | 3901.51 (2838.55 to 5115.73) | 2.08 (1.51 to 2.74) | 3065.17 (2224.74 to 4025.43) | 1.50 (1.09 to 1.96) |
| **2013** | 7130.75 (5192.20 to 9362.01) | 1.78 (1.29 to 2.34) | 3998.43 (2913.98 to 5244.21) | 2.09 (1.52 to 2.74) | 3132.32 (2278.23 to 4119.50) | 1.50 (1.09 to 1.97) |
| **2014** | 7292.62 (5319.89 to 9574.12) | 1.79 (1.30 to 2.34) | 4093.68 (2990.19 to 5372.79) | 2.10 (1.52 to 2.75) | 3198.94 (2326.69 to 4207.05) | 1.51 (1.10 to 1.97) |
| **2015** | 7461.04 (5443.14 to 9787.19) | 1.80 (1.30 to 2.35) | 4192.58 (3061.16 to 5505.81) | 2.11 (1.53 to 2.76) | 3268.45 (2377.03 to 4297.46) | 1.51 (1.10 to 1.98) |
| **2016** | 7639.21 (5571.36 to 10016.38) | 1.81 (1.31 to 2.37) | 4295.25 (3137.57 to 5629.24) | 2.12 (1.54 to 2.78) | 3343.96 (2431.46 to 4396.78) | 1.52 (1.11 to 1.99) |
| **2017** | 7846.30 (5718.06 to 10284.37) | 1.82 (1.32 to 2.39) | 4411.47 (3220.60 to 5772.64) | 2.14 (1.55 to 2.80) | 3434.83 (2496.68 to 4516.47) | 1.53 (1.12 to 2.01) |
| **2018** | 8057.23 (5871.36 to 10555.75) | 1.84 (1.34 to 2.41) | 4530.27 (3309.44 to 5923.48) | 2.15 (1.57 to 2.83) | 3526.96 (2561.93 to 4635.25) | 1.55 (1.13 to 2.03) |
| **2019** | 8245.61 (6004.31 to 10806.98) | 1.85 (1.34 to 2.43) | 4637.21 (3386.07 to 6068.32) | 2.17 (1.58 to 2.85) | 3608.40 (2618.14 to 4738.67) | 1.56 (1.13 to 2.04) |
| **2020** | 8416.10 (6129.02 to 11022.65) | 1.85 (1.34 to 2.43) | 4738.21 (3457.13 to 6205.63) | 2.18 (1.57 to 2.87) | 3677.89 (2667.12 to 4810.87) | 1.56 (1.13 to 2.03) |
| **2021** | 8597.05 (6259.71 to 11269.09) | 1.87 (1.36 to 2.46) | 4827.99 (3512.31 to 6332.66) | 2.19 (1.59 to 2.89) | 3769.07 (2733.16 to 4946.14) | 1.58 (1.15 to 2.08) |
| **Percentage Change,**  **1990-2021** | 82.26% (66.11% to 99.53%) | 12.80% (2.98% to 23.54%) | 88.22% (71.27% to 107.49%) | 12.05% (2.03% to 23.33%) | 75.15% (60.07% to 92.07%) | 12.57% (2.33% to 23.78%) |
| **Statistical Analysis** | Spearman’s r = 0.973 p-value < 0.001 | Spearman’s 𝜌 = 0.953 p-value < 0.001 | Spearman’s r = 0.973 p-value < 0.001 | Spearman’s 𝜌 = 0.953 p-value < 0.001 | Spearman’s r = 0.970 p-value < 0.001 | Spearman’s 𝜌 = 0.950 p-value < 0.001 |
|  |  |  | Wilcoxon rank sum test between sex (count): p-value = 0.003 Wilcoxon rank sum test between sex (rate): p-value < 0.001 | | | |

The numbers in parentheses represent the 95% uncertainty interval.

## Table S2f: Annual age-standardized years of life lost (YLL) rates and percentage changes from 1990 to 2021, by sex

|  | **Both** | | **Male** | | **Female** | |
| --- | --- | --- | --- | --- | --- | --- |
|  | **Absolute number** | **Age-standardized rate, per 100,000 people** | **Absolute number** | **Age-standardized rate, per 100,000 people** | **Absolute number** | **Age-standardized rate, per 100,000 people** |
| **1990** | 106934.92 (103345.08 to 109329.44) | 38.05 (36.95 to 38.85) | 58279.89 (56785.49 to 59513.45) | 45.03 (43.88 to 45.96) | 48655.03 (46428.36 to 50154.06) | 32.08 (30.88 to 32.93) |
| **1991** | 110393.55 (106550.75 to 113067.10) | 38.84 (37.71 to 39.72) | 60307.06 (58629.94 to 61616.42) | 45.91 (44.67 to 46.91) | 50086.49 (47726.52 to 51583.60) | 32.83 (31.67 to 33.69) |
| **1992** | 111074.66 (107567.48 to 113663.69) | 38.55 (37.52 to 39.38) | 60763.99 (59286.71 to 62039.47) | 45.60 (44.54 to 46.52) | 50310.67 (48048.58 to 51817.21) | 32.56 (31.45 to 33.40) |
| **1993** | 111990.43 (108399.16 to 114453.31) | 38.50 (37.48 to 39.28) | 60958.56 (59482.33 to 62117.41) | 45.20 (44.11 to 46.06) | 51031.87 (48797.58 to 52433.31) | 32.84 (31.77 to 33.63) |
| **1994** | 115498.05 (111876.41 to 118127.54) | 39.29 (38.25 to 40.13) | 62852.20 (61300.08 to 64084.48) | 46.03 (44.94 to 46.91) | 52645.85 (50346.98 to 54145.18) | 33.58 (32.49 to 34.41) |
| **1995** | 118599.34 (114762.18 to 121126.49) | 39.80 (38.74 to 40.58) | 64910.92 (63385.18 to 66173.20) | 46.78 (45.72 to 47.66) | 53688.43 (51324.12 to 55232.38) | 33.80 (32.61 to 34.63) |
| **1996** | 120353.48 (116498.46 to 123026.44) | 39.88 (38.82 to 40.68) | 65076.53 (63601.19 to 66336.49) | 46.17 (45.13 to 47.04) | 55276.95 (52842.09 to 56785.66) | 34.53 (33.32 to 35.35) |
| **1997** | 121574.38 (117560.96 to 124258.14) | 39.68 (38.57 to 40.47) | 66205.44 (64522.77 to 67491.16) | 46.21 (45.09 to 47.05) | 55368.94 (52878.24 to 56897.38) | 34.06 (32.86 to 34.89) |
| **1998** | 125698.52 (121071.25 to 128479.39) | 40.22 (39.05 to 41.03) | 68317.83 (66585.50 to 69652.33) | 46.85 (45.70 to 47.77) | 57380.69 (54434.85 to 59056.38) | 34.46 (33.10 to 35.29) |
| **1999** | 136896.43 (131872.31 to 139833.84) | 42.40 (41.06 to 43.22) | 75525.98 (73562.69 to 77041.86) | 50.33 (49.06 to 51.31) | 61370.45 (58044.63 to 63084.19) | 35.54 (34.06 to 36.38) |
| **2000** | 146745.36 (141160.77 to 150059.36) | 44.51 (43.05 to 45.40) | 81662.87 (79518.93 to 83280.50) | 53.23 (51.93 to 54.28) | 65082.49 (61547.80 to 66999.36) | 36.97 (35.40 to 37.88) |
| **2001** | 150025.19 (144429.79 to 153343.14) | 44.82 (43.31 to 45.72) | 84292.70 (82015.00 to 85926.58) | 54.10 (52.65 to 55.11) | 65732.49 (62163.24 to 67669.26) | 36.77 (35.19 to 37.69) |
| **2002** | 156646.12 (150852.80 to 160292.01) | 46.06 (44.59 to 47.03) | 87400.73 (85138.95 to 89148.37) | 55.08 (53.67 to 56.14) | 69245.39 (65470.29 to 71383.68) | 38.14 (36.53 to 39.10) |
| **2003** | 160576.78 (154634.40 to 164339.32) | 46.42 (44.92 to 47.37) | 90516.73 (88347.03 to 92321.08) | 55.89 (54.52 to 57.00) | 70060.05 (66228.88 to 72190.14) | 38.00 (36.33 to 39.00) |
| **2004** | 159531.48 (153505.92 to 163401.81) | 45.39 (43.96 to 46.40) | 90999.51 (88753.49 to 92831.70) | 55.17 (53.85 to 56.29) | 68531.97 (64816.31 to 70617.05) | 36.64 (35.10 to 37.60) |
| **2005** | 163308.59 (156696.74 to 167376.24) | 45.51 (44.00 to 46.52) | 93521.82 (91198.63 to 95463.32) | 55.50 (54.16 to 56.64) | 69786.76 (65924.98 to 72037.39) | 36.56 (34.90 to 37.54) |
| **2006** | 166505.81 (159575.43 to 170413.98) | 45.55 (43.94 to 46.53) | 95079.68 (92333.87 to 97259.53) | 55.20 (53.66 to 56.39) | 71426.13 (67280.31 to 73715.34) | 36.93 (35.14 to 37.91) |
| **2007** | 170042.73 (162400.93 to 174347.11) | 45.67 (43.97 to 46.72) | 95305.61 (92358.92 to 97668.48) | 54.28 (52.71 to 55.55) | 74737.12 (70315.35 to 77260.54) | 37.94 (36.16 to 39.02) |
| **2008** | 175059.19 (167608.60 to 179557.08) | 46.09 (44.41 to 47.16) | 98584.28 (95419.55 to 100809.90) | 54.94 (53.31 to 56.13) | 76474.91 (71835.51 to 79010.12) | 38.11 (36.25 to 39.17) |
| **2009** | 180837.84 (173079.66 to 185624.89) | 46.62 (44.85 to 47.74) | 102037.30 (98847.83 to 104376.25) | 55.70 (54.06 to 56.88) | 78800.55 (73898.53 to 81441.39) | 38.39 (36.45 to 39.54) |
| **2010** | 185552.12 (176679.22 to 190361.24) | 46.85 (44.96 to 47.94) | 104361.89 (100885.73 to 106739.54) | 55.70 (53.96 to 56.88) | 81190.23 (75960.02 to 84008.62) | 38.82 (36.81 to 39.96) |
| **2011** | 190836.02 (181581.89 to 195834.47) | 47.11 (45.17 to 48.23) | 107094.13 (103437.76 to 109619.03) | 55.81 (54.00 to 57.05) | 83741.89 (78370.02 to 86542.39) | 39.22 (37.15 to 40.37) |
| **2012** | 195382.22 (185502.68 to 200685.48) | 47.13 (45.02 to 48.28) | 110156.59 (106184.59 to 112806.53) | 56.02 (54.13 to 57.29) | 85225.63 (79582.15 to 88252.62) | 39.09 (36.94 to 40.26) |
| **2013** | 195440.36 (185515.07 to 200756.52) | 46.00 (43.91 to 47.11) | 110681.53 (106449.32 to 113283.01) | 54.88 (52.92 to 56.08) | 84758.82 (78885.78 to 87725.93) | 37.98 (35.83 to 39.08) |
| **2014** | 197697.70 (187425.37 to 203015.24) | 45.33 (43.32 to 46.44) | 112232.89 (107557.73 to 114979.04) | 54.35 (52.25 to 55.59) | 85464.81 (79640.20 to 88377.68) | 37.13 (35.04 to 38.23) |
| **2015** | 199211.32 (188861.20 to 204840.95) | 44.72 (42.76 to 45.80) | 113183.97 (108675.84 to 116014.70) | 53.70 (51.81 to 54.90) | 86027.35 (80276.73 to 88994.15) | 36.53 (34.53 to 37.61) |
| **2016** | 195278.57 (185607.20 to 200581.50) | 42.97 (41.17 to 43.99) | 111442.68 (107147.30 to 114323.17) | 51.73 (49.94 to 52.88) | 83835.89 (78289.94 to 86662.54) | 34.94 (33.11 to 35.94) |
| **2017** | 194196.72 (184513.54 to 199422.64) | 41.51 (39.77 to 42.49) | 110893.96 (106761.75 to 113739.26) | 49.94 (48.28 to 51.11) | 83302.76 (77576.95 to 86197.36) | 33.78 (31.95 to 34.77) |
| **2018** | 192667.52 (183158.45 to 198078.21) | 40.09 (38.43 to 41.08) | 109554.79 (105689.86 to 112404.60) | 47.98 (46.49 to 49.16) | 83112.74 (77194.28 to 86137.83) | 32.88 (30.98 to 33.91) |
| **2019** | 194581.16 (184649.43 to 200282.01) | 39.62 (37.95 to 40.66) | 111110.90 (107333.09 to 114087.22) | 47.80 (46.33 to 49.02) | 83470.26 (77332.15 to 86554.99) | 32.13 (30.25 to 33.13) |
| **2020** | 197171.83 (187038.58 to 203952.18) | 39.32 (37.55 to 40.56) | 112701.51 (108029.37 to 116709.10) | 47.58 (45.76 to 49.22) | 84470.32 (78112.95 to 88111.35) | 31.76 (29.78 to 32.87) |
| **2021** | 201962.40 (191037.84 to 210306.37) | 39.48 (37.75 to 40.98) | 115272.61 (109575.75 to 120038.51) | 47.82 (45.55 to 49.73) | 86689.78 (80157.39 to 91098.92) | 31.87 (29.91 to 33.22) |
| **Percentage Change,**  **1990-2021** | 88.86% (81.59% to 95.60%) | 3.76% (-0.25% to 7.38%) | 97.79% (88.17% to 106.27%) | 6.19% (1.17% to 10.62%) | 78.17% (70.63% to 86.70%) | -0.67% (-4.43% to 3.37%) |
| **Statistical Analysis** | Spearman’s 𝜌 = 0.970 p-value < 0.001 | Spearman’s 𝜌 = 0.360 p-value = 0.044 | Spearman’s 𝜌 = 0.983 p-value < 0.001 | Spearman’s 𝜌 = 0.452 p-value = 0.010 | Spearman’s 𝜌 = 0.955 p-value < 0.001 | Spearman’s 𝜌 = 0.143 p-value = 0.435 |
|  |  |  | Wilcoxon rank sum test between sex (count): p-value < 0.001 Wilcoxon rank sum test between sex (rate): p-value < 0.001 | | | |

The numbers in parentheses represent the 95% uncertainty interval.

**Table S3: Numbers and age-standardized rates, and percentage changes of Prevalence, DALYs, and deaths in the US.**

|  | **Prevalence (95% UI)** | | | **DALYs (95% UI)** | | | **Deaths (95% UI)** | | |
| --- | --- | --- | --- | --- | --- | --- | --- | --- | --- |
|  | **Absolute number, thousands, 2021** | **Age-standardized rate,**  **per 100,000 people, 2021** | **Percentage Change,**  **1990-2021** | **Absolute number, thousands, 2021** | **Age-standardized rate,**  **per 100,000 people, 2021** | **Percentage Change,**  **1990-2021** | **Absolute number, thousands, 2021** | **Age-standardized rate,**  **per 100,000 people, 2021** | **Percentage Change,**  **1990-2021** |
| **United States of America** | **40495.43 (37645.36 to 43576.27)** | **8.82 (8.26 to 9.51)** | **12.89% (3.10% to 23.66%)** | **210559.45 (199250.06 to 218996.64)** | **41.36 (39.47 to 42.94)** | **4.14% (0.41% to 7.68%)** | **8465.56 (7799.92 to 8898.99)** | **1.49 (1.38 to 1.56)** | **18.34% (13.86% to 22.70%)** |
| **Northeast** | 8183.64 (7585.57 to 8850.32) | 10.18 (9.34 to 11.14) | 17.66% (6.48% to 30.05%) | 35531.92 (30086.29 to 41220.94) | 40.24 (34.42 to 46.48) | -1.67% (-16.04% to 13.94%) | **8465.56 (7799.92 to 8898.99)** | 1.46 (1.24 to 1.71) | 9.55% (-7.31% to 28.16%) |
| **Midwest** | 8739.43 (8027.03 to 9522.62) | 9.25 (8.42 to 10.16) | 15.17% (4.99% to 27.17%) | 49542.83 (42262.27 to 57480.71) | 46.88 (40.36 to 53.89) | 13.48% (-2.52% to 30.87%) | 1448.54 (1210.87 to 1687.98) | 1.69 (1.44 to 1.97) | 25.72% (6.89% to 46.42%) |
| **South** | 14388.31 (13313.62 to 15571.30) | 8.37 (7.69 to 9.13) | 20.43% (8.80% to 34.01%) | 78767.27 (67435.83 to 90938.09) | 44.11 (38.10 to 50.68) | 14.67% (-1.52% to 32.67%) | 2015.52 (1706.17 to 2350.38) | 1.55 (1.32 to 1.79) | 28.56% (9.47% to 49.93%) |
| **West** | 9184.04 (8481.42 to 10011.31) | 9.12 (8.27 to 10.11) | 12.89% (2.35% to 24.99%) | 46717.43 (40086.34 to 53761.52) | 43.41 (37.51 to 49.86) | 5.10% (-9.34% to 21.39%) | 3127.04 (2649.51 to 3635.53) | 1.56 (1.32 to 1.81) | 17.75% (0.45% to 37.41%) |
| **New England** | **2318.19**  **(2118.83 to 2539.97)** | **10.66 (9.60 to 11.82)** | **16.86% (7.11% to 27.82%)** | **10694.16 (8929.55 to 12502.75)** | **43.73 (37.34 to 50.55)** | **0.59% (-14.63% to 17.01%)** | **447.14 (368.72 to 524.53)** | **1.60 (1.35 to 1.87)** | **9.02% (-8.26% to 28.12%)** |
| **Connecticut** | 561.43 (522.32 to 606.48) | 10.57 (9.79 to 11.47) | 15.25% (4.27% to 27.75%) | 2467.62 (2055.40 to 2890.17) | 41.42 (34.90 to 48.25) | -0.12% (-16.69% to 17.37%) | 101.73 (82.87 to 119.94) | 1.51 (1.25 to 1.77) | 8.93% (-9.98% to 29.35%) |
| **Maine** | 199.14 (177.72 to 220.87) | 9.17 (8.21 to 10.17) | 8.78% (-1.84% to 20.14%) | 1306.36 (1119.83 to 1515.61) | 52.17 (44.85 to 60.04) | 19.69% (0.99% to 38.36%) | 54.97 (46.89 to 64.24) | 1.90 (1.62 to 2.21) | 26.63% (5.74% to 47.57%) |
| **Massachusetts** | 1051.96 (974.54 to 1143.16) | 10.40 (9.66 to 11.25) | 11.54% (2.67% to 23.17%) | 4664.18 (3834.31 to 5479.88) | 40.18 (33.19 to 47.19) | -5.15% (-21.41% to 11.36%) | 196.93 (160.56 to 231.25) | 1.53 (1.26 to 1.81) | 8.22% (-10.67% to 28.06%) |
| **New Hampshire** | 218.73 (197.69 to 241.15) | 10.35 (9.41 to 11.41) | 14.47% (4.80% to 26.00%) | 1032.05 (866.64 to 1203.14) | 42.23 (35.71 to 48.82) | -1.59% (-16.06% to 15.53%) | 42.85 (35.60 to 50.28) | 1.56 (1.30 to 1.82) | 4.77% (-11.98% to 24.71%) |
| **Rhode Island** | 137.07 (116.68 to 157.57) | 8.77 (7.49 to 10.11) | 17.37% (8.40% to 26.87%) | 655.82 (554.16 to 765.05) | 36.32 (31.22 to 42.24) | -9.32% (-22.11% to 5.15%) | 27.26 (22.82 to 32.02) | 1.34 (1.13 to 1.58) | 0.94% (-12.93% to 18.28%) |
| **Vermont** | 149.85 (129.87 to 170.74) | 14.69 (13.07 to 16.52) | 33.73% (24.36% to 42.96%) | 568.14 (499.20 to 648.90) | 50.04 (44.16 to 56.74) | 0.02% (-12.48% to 14.30%) | 23.40 (19.97 to 26.81) | 1.78 (1.54 to 2.04) | 4.62% (-9.75% to 20.77%) |
| **Middle Atlantic** | **5865.45 (5466.75 to 6310.34)** | **9.70 (9.07 to 10.45)** | **18.46% (5.84% to 32.28%)** | **24837.76 (21156.75 to 28718.19)** | **36.74 (31.51 to 42.41)** | **-3.92% (-17.44% to 10.87%)** | **1001.41 (842.15 to 1163.45)** | **1.33 (1.12 to 1.54)** | **10.07% (-6.36% to 28.19%)** |
| **New Jersey** | 1213.39 (1130.38 to 1307.44) | 9.35 (8.72 to 10.11) | 11.84% (-0.02% to 23.90%) | 4902.38 (4174.13 to 5767.31) | 33.11 (28.30 to 38.63) | -12.66% (-25.53% to 2.41%) | 199.11 (168.02 to 234.09) | 1.22 (1.03 to 1.43) | -0.22% (-15.68% to 18.61%) |
| **New York** | 2790.73 (2604.30 to 2993.38) | 9.90 (9.28 to 10.60) | 21.12% (8.49% to 35.08%) | 10477.60 (8975.21 to 12027.09) | 32.99 (28.43 to 37.69) | -8.58% (-20.86% to 4.59%) | 418.34 (350.68 to 485.01) | 1.19 (1.00 to 1.38) | 10.18% (-6.14% to 26.83%) |
| **Pennsylvania** | 1861.33 (1732.07 to 2009.52) | 9.86 (9.21 to 10.64) | 22.41% (9.06% to 37.88%) | 9457.78 (8007.41 to 10923.79) | 44.14 (37.80 to 50.92) | 9.47% (-5.94% to 25.60%) | 383.96 (323.45 to 444.35) | 1.58 (1.34 to 1.82) | 20.25% (2.74% to 39.14%) |
| **East North Central** | **5885.53 (5462.66 to 6357.55)** | **8.88 (8.28 to 9.59)** | **11.13% (0.50% to 23.82%)** | **33122.96 (28354.74 to 38312.03)** | **45.41 (39.18 to 52.17)** | **11.53% (-3.46% to 28.42%)** | **1340.25 (1139.82 to 1555.34)** | **1.64 (1.40 to 1.90)** | **24.73% (6.95% to 44.94%)** |
| **Illinois** | 1479.68 (1381.43 to 1592.21) | 8.57 (8.02 to 9.22) | 6.60% (-4.04% to 19.73%) | 7510.06 (6460.11 to 8712.85) | 38.82 (33.72 to 44.53) | -1.78% (-14.82% to 12.75%) | 302.81 (258.68 to 354.23) | 1.41 (1.21 to 1.64) | 16.00% (-0.22% to 35.64%) |
| **Indiana** | 776.28 (716.95 to 836.17) | 8.43 (7.80 to 9.09) | 11.69% (0.52% to 24.47%) | 4961.30 (4252.75 to 5683.89) | 48.43 (41.87 to 55.16) | 20.73% (4.68% to 38.83%) | 198.87 (170.50 to 228.42) | 1.75 (1.50 to 2.00) | 33.71% (14.69% to 55.07%) |
| **Michigan** | 1369.58 (1273.57 to 1479.88) | 9.35 (8.75 to 10.11) | 17.79% (8.23% to 29.62%) | 7344.97 (6289.71 to 8457.05) | 44.94 (38.78 to 51.57) | 12.19% (-3.50% to 28.68%) | 295.34 (250.62 to 340.86) | 1.59 (1.36 to 1.84) | 24.80% (7.09% to 44.58%) |
| **Ohio** | 1484.74 (1376.30 to 1605.85) | 8.87 (8.32 to 9.53) | 12.99% (2.08% to 25.78%) | 8754.95 (7515.71 to 10119.01) | 46.72 (40.57 to 53.66) | 13.96% (0.52% to 30.68%) | 352.01 (301.09 to 406.70) | 1.66 (1.42 to 1.92) | 25.65% (9.31% to 44.79%) |
| **Wisconsin** | 775.25 (714.40 to 843.43) | 9.17 (8.48 to 10.00) | 6.59% (-4.27% to 19.52%) | 4551.68 (3836.46 to 5339.23) | 48.11 (40.95 to 55.91) | 12.54% (-4.19% to 31.17%) | 191.23 (158.94 to 225.11) | 1.79 (1.49 to 2.10) | 23.49% (3.90% to 44.62%) |
| **West North Central** | **2853.91 (2564.38 to 3165.07)** | **9.62 (8.56 to 10.74)** | **19.20% (9.48% to 30.51%)** | **16419.87 (13907.53 to 19168.67)** | **48.35 (41.54 to 55.61)** | **15.43% (-1.59% to 33.31%)** | **675.27 (566.35 to 795.04)** | **1.74 (1.48 to 2.03)** | **26.72% (6.84% to 47.91%)** |
| **Iowa** | 347.38 (304.98 to 393.22) | 7.70 (6.81 to 8.67) | 3.27% (-5.69% to 12.91%) | 2302.63 (1949.06 to 2668.59) | 46.01 (39.18 to 52.88) | 21.36% (2.06% to 40.39%) | 96.44 (81.12 to 112.34) | 1.69 (1.42 to 1.96) | 34.28% (11.60% to 57.84%) |
| **Kansas** | 368.02 (330.06 to 410.28) | 9.31 (8.36 to 10.32) | 17.47% (7.12% to 30.12%) | 2180.87 (1826.32 to 2547.58) | 49.90 (42.10 to 58.10) | 18.87% (-0.47% to 38.87%) | 88.95 (74.50 to 104.12) | 1.82 (1.53 to 2.13) | 33.51% (10.31% to 57.69%) |
| **Minnesota** | 931.87 (840.87 to 1023.44) | 11.54 (10.52 to 12.62) | 16.68% (6.09% to 30.67%) | 4784.93 (4020.08 to 5645.00) | 53.05 (44.77 to 61.98) | 10.53% (-6.57% to 28.96%) | 200.53 (164.69 to 240.07) | 2.00 (1.65 to 2.39) | 21.88% (2.02% to 44.00%) |
| **Missouri** | 716.81 (666.00 to 773.68) | 8.37 (7.79 to 9.03) | 13.61% (1.00% to 27.69%) | 4616.64 (3924.87 to 5390.10) | 48.24 (41.50 to 55.86) | 20.53% (2.33% to 40.06%) | 186.78 (158.39 to 219.66) | 1.73 (1.47 to 2.02) | 33.36% (12.17% to 56.57%) |
| **Nebraska** | 243.62 (211.26 to 280.89) | 9.08 (7.87 to 10.39) | 17.08% (8.26% to 26.79%) | 1385.99 (1180.25 to 1606.26) | 47.42 (40.61 to 54.38) | 14.53% (-2.58% to 31.86%) | 56.72 (48.04 to 65.89) | 1.73 (1.47 to 2.01) | 25.90% (5.53% to 45.66%) |
| **North Dakota** | 120.77 (103.13 to 139.21) | 11.09 (9.58 to 12.69) | 32.59% (23.80% to 42.11%) | 509.27 (444.69 to 580.66) | 45.20 (39.79 to 51.10) | 7.20% (-6.36% to 22.48%) | 20.12 (17.22 to 23.18) | 1.54 (1.32 to 1.78) | 12.85% (-2.27% to 29.56%) |
| **South Dakota** | 125.45 (108.08 to 144.35) | 10.22 (9.01 to 11.43) | 33.72% (25.79% to 43.27%) | 639.54 (562.27 to 730.48) | 48.66 (42.85 to 55.00) | 14.99% (0.49% to 30.56%) | 25.74 (22.40 to 29.78) | 1.68 (1.47 to 1.94) | 25.23% (8.50% to 44.03%) |
| **South Atlantic** | **7671.55 (7092.89 to 8313.09)** | **8.47 (7.77 to 9.24)** | **17.03% (6.14% to 30.15%)** | **42671.81 (36358.40 to 49428.80)** | **41.59 (36.00 to 47.66)** | **4.07% (-10.86% to 20.32%)** | **1720.48 (1444.47 to 2007.71)** | **1.46 (1.24 to 1.70)** | **18.54% (0.55% to 38.18%)** |
| **Delaware** | 154.61 (142.00 to 168.76) | 10.85 (9.97 to 11.80) | 31.08% (19.44% to 44.66%) | 694.91 (614.77 to 789.69) | 43.47 (38.81 to 48.93) | 10.22% (-3.25% to 24.73%) | 28.02 (24.54 to 32.39) | 1.51 (1.33 to 1.73) | 17.83% (3.11% to 34.62%) |
| **District of Columbia** | 64.75 (56.68 to 74.60) | 7.95 (6.97 to 9.13) | 28.28% (18.06% to 40.21%) | 261.52 (226.11 to 302.41) | 30.65 (26.74 to 35.25) | -47.80% (-56.19% to -38.44%) | 9.35 (7.93 to 10.85) | 1.01 (0.86 to 1.17) | -24.44% (-36.84% to  -11.38%) |
| **Florida** | 2702.84 (2512.31 to 2909.89) | 8.11 (7.55 to 8.73) | 17.57% (4.77% to 32.71%) | 13864.25 (11738.85 to 16152.59) | 38.13 (32.82 to 43.86) | 0.67% (-13.04% to 16.31%) | 568.39 (473.42 to 667.00) | 1.34 (1.12 to 1.56) | 22.03% (3.55% to 42.21%) |
| **Georgia** | 1037.25 (956.83 to 1119.01) | 7.42 (6.91 to 7.99) | 8.11% (-1.02% to 20.03%) | 6081.83 (5246.13 to 7013.91) | 39.06 (33.94 to 44.57) | 6.10% (-8.13% to 22.05%) | 240.79 (204.06 to 281.32) | 1.42 (1.21 to 1.65) | 22.05% (4.11% to 41.55%) |
| **Maryland** | 719.61 (656.95 to 790.19) | 8.58 (7.77 to 9.47) | 10.52% (0.21% to 22.37%) | 3863.25 (3252.55 to 4513.68) | 41.00 (34.93 to 47.59) | 3.63% (-13.15% to 19.74%) | 152.81 (126.81 to 180.42) | 1.47 (1.22 to 1.72) | 14.65% (-4.61% to 33.58%) |
| **North Carolina** | 1254.69 (1170.09 to 1351.20) | 8.57 (8.00 to 9.20) | 13.94% (1.42% to 28.63%) | 7205.48 (6177.51 to 8288.28) | 44.66 (38.51 to 51.15) | 10.09% (-4.60% to 27.68%) | 290.66 (245.35 to 335.00) | 1.60 (1.36 to 1.85) | 24.11% (5.90% to 45.70%) |
| **South Carolina** | 606.54 (559.62 to 660.75) | 8.15 (7.59 to 8.78) | 13.53% (2.43% to 27.25%) | 3528.25 (3007.32 to 4048.40) | 43.55 (37.54 to 49.63) | 9.27% (-7.42% to 27.09%) | 140.13 (118.06 to 161.20) | 1.52 (1.29 to 1.75) | 24.31% (4.72% to 44.02%) |
| **Virginia** | 894.17 (826.23 to 975.33) | 7.63 (7.03 to 8.32) | -2.32% (-11.52% to 9.32%) | 5661.34 (4805.92 to 6575.85) | 42.87 (36.88 to 49.43) | 1.95% (-13.28% to 18.75%) | 230.29 (193.52 to 269.76) | 1.58 (1.33 to 1.84) | 14.49% (-4.18% to 35.36%) |
| **West Virginia** | 237.08 (212.18 to 263.35) | 8.93 (8.15 to 9.76) | 32.52% (21.46% to 46.21%) | 1510.99 (1289.23 to 1743.99) | 50.93 (43.81 to 58.56) | 42.50% (21.34% to 64.94%) | 60.04 (50.78 to 69.78) | 1.74 (1.48 to 2.02) | 51.87% (29.21% to 77.93%) |
| **East South Central** | **2085.02 (1912.83 to 2273.71)** | **7.72 (7.07 to 8.44)** | **19.76% (8.30% to 32.59%)** | **13730.36 (11647.31 to 15883.60)** | **46.03 (39.41 to 53.08)** | **23.82% (5.97% to 43.53%)** | **539.04 (456.79 to 625.55)** | **1.61 (1.36 to 1.87)** | **36.72% (16.18% to 59.30%)** |
| **Alabama** | 527.04 (485.12 to 572.87) | 7.71 (7.05 to 8.46) | 20.79% (8.47% to 34.14%) | 3662.79 (3105.26 to 4242.72) | 47.85 (41.16 to 55.03) | 28.53% (10.43% to 48.41%) | 144.10 (122.23 to 166.91) | 1.67 (1.43 to 1.94) | 43.00% (22.69% to 67.23%) |
| **Kentucky** | 519.10 (481.09 to 562.63) | 8.31 (7.69 to 9.01) | 20.78% (8.80% to 34.76%) | 3181.23 (2673.61 to 3705.44) | 45.81 (38.75 to 53.07) | 23.13% (4.48% to 44.24%) | 124.43 (104.41 to 146.43) | 1.61 (1.35 to 1.90) | 33.38% (11.97% to 55.82%) |
| **Mississippi** | 262.61 (232.60 to 292.73) | 6.66 (5.92 to 7.40) | 17.27% (7.16% to 28.37%) | 1855.54 (1583.34 to 2165.55) | 43.12 (37.18 to 50.05) | 21.90% (4.77% to 42.17%) | 71.47 (60.85 to 83.62) | 1.48 (1.26 to 1.72) | 36.33% (15.87% to 59.15%) |
| **Tennessee** | 776.26 (714.02 to 845.48) | 8.20 (7.62 to 8.89) | 20.22% (8.76% to 33.08%) | 5030.79 (4285.09 to 5769.89) | 47.31 (40.55 to 54.18) | 21.70% (4.19% to 39.31%) | 199.04 (169.30 to 228.60) | 1.67 (1.42 to 1.92) | 34.18% (14.21% to 54.98%) |
| **West South Central** | **4631.74 (4307.90 to 4984.50)** | **8.92 (8.23 to 9.70)** | **24.50% (11.96% to 39.29%)** | **22365.10 (19430.13 to 25625.68)** | **44.72 (38.89 to 51.29)** | **16.14% (0.34% to 34.15%)** | **867.52 (748.24 to 1002.27)** | **1.57 (1.35 to 1.82)** | **30.42% (11.69% to 52.32%)** |
| **Arkansas** | 383.61 (348.09 to 423.32) | 9.37 (8.52 to 10.35) | 31.58% (17.89% to 46.48%) | 2183.00 (1871.13 to 2521.17) | 48.71 (41.99 to 55.88) | 22.75% (4.53% to 42.69%) | 85.05 (72.15 to 99.24) | 1.67 (1.43 to 1.94) | 35.62% (14.20% to 59.55%) |
| **Louisiana** | 590.87 (548.15 to 635.08) | 9.66 (9.00 to 10.39) | 35.74% (21.55% to 54.50%) | 3087.09 (2663.16 to 3590.62) | 45.75 (39.86 to 53.01) | 15.76% (-0.50% to 34.31%) | 117.67 (100.79 to 137.82) | 1.57 (1.35 to 1.84) | 29.56% (9.80% to 51.75%) |
| **Oklahoma** | 412.00 (378.75 to 448.46) | 7.88 (7.20 to 8.65) | 15.90% (4.76% to 28.62%) | 2791.26 (2427.24 to 3207.29) | 48.40 (42.04 to 55.45) | 29.99% (13.12% to 50.24%) | 110.75 (96.02 to 128.32) | 1.72 (1.49 to 1.99) | 46.15% (27.06% to 70.34%) |
| **Texas** | 3245.26 (3032.91 to 3477.65) | 8.79 (8.20 to 9.43) | 14.78% (3.64% to 27.57%) | 14303.74 (12468.59 to 16306.60) | 36.01 (31.66 to 40.83) | -3.95% (-15.78% to 9.37%) | 554.06 (479.29 to 636.89) | 1.30 (1.13 to 1.49) | 10.34% (-4.32% to 27.63%) |
| **Mountain** | **2996.80 (2738.05 to 3285.69)** | **8.79 (7.96 to 9.74)** | **12.58% (2.09% to 24.66%)** | **16281.01 (13880.70 to 18861.89)** | **45.35 (39.14 to 52.12)** | **10.30% (-4.71% to 27.61%)** | **650.88 (547.40 to 758.84)** | **1.63 (1.39 to 1.89)** | **24.03% (5.97% to 44.72%)** |
| **Arizona** | 874.40 (810.14 to 945.52) | 8.84 (8.14 to 9.57) | 20.86% (7.73% to 36.11%) | 4781.60 (4116.17 to 5502.53) | 44.11 (38.43 to 50.53) | 11.39% (-3.74% to 28.51%) | 190.98 (161.56 to 220.82) | 1.54 (1.31 to 1.77) | 29.60% (10.33% to 50.52%) |
| **Colorado** | 819.47 (758.22 to 887.19) | 10.33 (9.61 to 11.21) | 14.90% (3.11% to 28.27%) | 4062.15 (3413.58 to 4737.90) | 46.56 (39.62 to 53.94) | 0.91% (-13.87% to 16.86%) | 161.11 (132.31 to 189.65) | 1.69 (1.39 to 1.99) | 13.54% (-4.48% to 32.45%) |
| **Idaho** | 223.45 (204.28 to 247.00) | 8.89 (8.17 to 9.71) | 4.52% (-5.92% to 17.16%) | 1364.44 (1148.28 to 1588.82) | 49.46 (41.75 to 56.97) | 8.93% (-6.49% to 26.22%) | 56.41 (47.28 to 65.41) | 1.84 (1.55 to 2.14) | 23.34% (4.31% to 43.34%) |
| **Montana** | 144.01 (128.97 to 162.19) | 9.09 (8.17 to 10.09) | 15.07% (4.61% to 27.33%) | 916.60 (785.20 to 1073.98) | 50.98 (44.09 to 59.18) | 15.55% (0.02% to 36.23%) | 37.78 (32.18 to 44.24) | 1.80 (1.54 to 2.11) | 24.68% (7.69% to 47.86%) |
| **Nevada** | 279.14 (250.10 to 311.37) | 6.86 (6.14 to 7.72) | 4.81% (-4.42% to 14.95%) | 1559.46 (1326.91 to 1798.02) | 33.82 (29.06 to 38.77) | -0.21% (-14.31% to 14.87%) | 61.73 (52.16 to 71.82) | 1.22 (1.03 to 1.41) | 13.35% (-3.59% to 32.08%) |
| **New Mexico** | 212.25 (192.32 to 234.20) | 7.08 (6.45 to 7.84) | 8.94% (-1.05% to 20.39%) | 1362.17 (1161.25 to 1588.64) | 42.35 (36.21 to 48.86) | 25.24% (7.68% to 45.79%) | 53.93 (46.22 to 63.47) | 1.45 (1.24 to 1.71) | 38.99% (18.05% to 63.93%) |
| **Utah** | 366.64 (328.19 to 408.95) | 9.70 (8.77 to 10.77) | 10.02% (-0.28% to 21.72%) | 1799.26 (1542.22 to 2080.15) | 46.14 (39.86 to 53.13) | 4.83% (-8.96% to 21.66%) | 71.35 (60.27 to 83.33) | 1.73 (1.46 to 2.02) | 21.14% (3.52% to 41.83%) |
| **Wyoming** | 77.44 (65.84 to 89.26) | 9.54 (8.21 to 10.97) | 21.52% (12.89% to 31.32%) | 435.33 (387.09 to 491.85) | 49.41 (44.06 to 55.55) | 15.73% (1.95% to 30.72%) | 17.60 (15.42 to 20.09) | 1.77 (1.56 to 2.02) | 27.56% (11.95% to 45.74%) |
| **Pacific** | **6187.24 (5743.36 to 6725.62)** | **9.45 (8.58 to 10.48)** | **13.20% (2.61% to 25.32%)** | **30436.42 (26205.64 to 34899.63)** | **41.47 (35.88 to 47.61)** | **-0.09% (-13.97% to 15.16%)** | **1223.57 (1046.27 to 1416.98)** | **1.48 (1.26 to 1.72)** | **11.47% (-5.08% to 30.10%)** |
| **Alaska** | 91.22 (79.00 to 105.06) | 10.17 (8.92 to 11.56) | 31.47% (19.32% to 43.94%) | 398.65 (347.85 to 455.05) | 41.13 (36.26 to 46.87) | 1.71% (-10.69% to 16.27%) | 14.54 (12.62 to 16.74) | 1.39 (1.21 to 1.59) | 8.73% (-6.12% to 27.07%) |
| **California** | 4300.56 (4028.65 to 4639.75) | 8.13 (7.65 to 8.72) | 4.04% (-7.28% to 16.85%) | 19817.21 (17190.89 to 22569.75) | 34.26 (29.89 to 38.77) | -13.10% (-23.80% to  -2.24%) | 789.01 (678.90 to 908.49) | 1.24 (1.07 to 1.43) | 1.10% (-12.80% to 14.40%) |
| **Hawaii** | 204.28 (180.20 to 233.29) | 10.25 (9.14 to 11.60) | 30.38% (20.91% to 41.65%) | 747.96 (630.79 to 874.09) | 32.96 (28.29 to 38.00) | 3.77% (-12.04% to 21.40%) | 29.62 (24.74 to 34.86) | 1.11 (0.93 to 1.30) | 16.70% (-2.78% to 38.36%) |
| **Oregon** | 603.50 (539.02 to 672.42) | 9.44 (8.66 to 10.39) | 4.34% (-6.15% to 16.58%) | 3654.14 (3134.65 to 4312.72) | 51.15 (44.29 to 59.66) | 8.21% (-7.01% to 27.22%) | 153.47 (129.91 to 182.08) | 1.90 (1.62 to 2.25) | 21.82% (4.55% to 44.85%) |
| **Washington** | 987.69 (916.49 to 1075.09) | 9.26 (8.54 to 10.11) | -4.22% (-13.76% to 7.58%) | 5818.47 (4901.46 to 6688.01) | 47.84 (40.68 to 54.75) | -1.02% (-16.33% to 13.15%) | 236.93 (200.10 to 274.80) | 1.76 (1.49 to 2.04) | 9.00% (-8.25% to 25.81%) |

Abbreviations: UI, uncertainty interval; DALYs, disability-adjusted life years

## Table S4a: Numbers and age-standardized rates of prevalence in 2021, and percentage changes from 1990 to 2021, by sex

|  | **Male** | | | **Female** | | |
| --- | --- | --- | --- | --- | --- | --- |
|  | **Absolute number, 2021** | **Age-standardized rate, per 100,000 people, 2021** | **Percentage Change, 1990-2021** | **Absolute number, thousands, 2021** | **Age-standardized rate, per 100,000 people, 2021** | **Percentage Change, 1990-2021** |
| **United States of America** | 22755.73 (21262.86 to 24479.88) | 10.33 (9.74 to 11.13) | 12.20% (2.08% to 23.37%) | 17739.70 (16413.50 to 19154.57) | 7.46 (6.96 to 8.05) | 12.59% (2.33% to 23.78%) |
| **Alabama** | 286.73 (264.10 to 313.01) | 8.77 (8.01 to 9.65) | 18.75% (6.32% to 33.23%) | 240.32 (219.97 to 263.70) | 6.79 (6.17 to 7.53) | 22.51% (8.16% to 38.00%) |
| **Alaska** | 48.18 (41.21 to 55.87) | 10.34 (8.98 to 11.86) | 26.57% (13.05% to 41.34%) | 43.03 (36.88 to 49.69) | 10.00 (8.62 to 11.34) | 37.52% (24.86% to 50.42%) |
| **Arizona** | 505.86 (469.98 to 546.93) | 10.56 (9.77 to 11.42) | 22.29% (8.33% to 38.49%) | 368.54 (334.87 to 404.19) | 7.25 (6.56 to 7.96) | 18.05% (3.96% to 35.51%) |
| **Arkansas** | 212.07 (192.45 to 233.66) | 10.80 (9.83 to 11.85) | 26.97% (11.69% to 43.48%) | 171.54 (153.98 to 193.45) | 8.06 (7.20 to 9.09) | 35.35% (20.16% to 51.94%) |
| **California** | 2500.75 (2333.95 to 2702.63) | 9.81 (9.18 to 10.55) | 4.64% (-8.34% to 17.79%) | 1799.81 (1670.16 to 1954.56) | 6.59 (6.16 to 7.13) | 2.56% (-8.48% to 16.56%) |
| **Colorado** | 532.70 (492.64 to 579.28) | 13.78 (12.83 to 14.98) | 25.69% (11.04% to 43.71%) | 286.76 (260.23 to 313.75) | 7.08 (6.43 to 7.75) | -2.43% (-13.12% to 10.12%) |
| **Connecticut** | 313.26 (289.09 to 338.77) | 12.38 (11.52 to 13.35) | 14.03% (1.84% to 28.71%) | 248.17 (226.51 to 270.02) | 8.94 (8.20 to 9.80) | 15.61% (4.22% to 28.82%) |
| **Delaware** | 99.73 (91.38 to 109.16) | 14.47 (13.35 to 15.69) | 40.72% (27.10% to 58.98%) | 54.89 (49.37 to 61.32) | 7.68 (6.84 to 8.53) | 16.67% (5.93% to 29.52%) |
| **District of Columbia** | 35.09 (30.58 to 40.54) | 9.27 (8.14 to 10.68) | 27.63% (16.04% to 40.51%) | 29.66 (25.49 to 34.45) | 6.75 (5.90 to 7.84) | 25.09% (13.60% to 39.28%) |
| **Florida** | 1461.38 (1348.23 to 1581.73) | 9.13 (8.45 to 9.83) | 13.99% (0.26% to 28.42%) | 1241.46 (1147.55 to 1349.25) | 7.20 (6.66 to 7.82) | 21.41% (7.35% to 37.54%) |
| **Georgia** | 614.21 (566.06 to 667.28) | 9.31 (8.62 to 10.05) | 11.20% (0.79% to 24.74%) | 423.04 (389.66 to 458.56) | 5.76 (5.29 to 6.25) | 2.89% (-6.72% to 13.93%) |
| **Hawaii** | 97.58 (85.39 to 111.24) | 10.04 (8.82 to 11.37) | 25.31% (15.43% to 37.29%) | 106.70 (93.64 to 121.99) | 10.47 (9.22 to 11.95) | 34.93% (20.90% to 50.73%) |
| **Idaho** | 144.97 (132.54 to 158.35) | 11.72 (10.77 to 12.79) | 14.25% (0.32% to 32.24%) | 78.48 (69.58 to 89.45) | 6.15 (5.45 to 7.00) | -10.51% (-19.27% to 0.04%) |
| **Illinois** | 768.97 (715.25 to 827.75) | 9.28 (8.61 to 9.97) | 1.27% (-10.06% to 14.47%) | 710.71 (658.61 to 775.77) | 7.96 (7.41 to 8.64) | 12.23% (-0.47% to 26.45%) |
| **Indiana** | 441.31 (406.07 to 476.46) | 9.94 (9.21 to 10.72) | 10.50% (-1.83% to 25.10%) | 334.96 (307.69 to 364.76) | 7.06 (6.47 to 7.67) | 11.85% (0.76% to 25.89%) |
| **Iowa** | 184.86 (163.02 to 210.35) | 8.47 (7.41 to 9.65) | -0.91% (-11.25% to 10.03%) | 162.52 (142.18 to 184.70) | 7.00 (6.18 to 7.88) | 7.26% (-3.43% to 18.02%) |
| **Kansas** | 220.07 (198.88 to 244.98) | 11.51 (10.43 to 12.76) | 18.98% (7.55% to 33.91%) | 147.95 (130.18 to 166.78) | 7.24 (6.41 to 8.12) | 13.32% (2.41% to 26.68%) |
| **Kentucky** | 324.78 (300.47 to 350.53) | 10.75 (9.99 to 11.63) | 27.23% (12.23% to 44.53%) | 194.32 (175.70 to 211.93) | 6.06 (5.51 to 6.66) | 9.55% (-1.91% to 23.81%) |
| **Louisiana** | 316.29 (289.49 to 343.08) | 10.68 (9.86 to 11.50) | 30.76% (15.87% to 49.07%) | 274.58 (254.51 to 296.91) | 8.76 (8.08 to 9.45) | 40.20% (24.44% to 60.93%) |
| **Maine** | 109.24 (98.19 to 121.52) | 10.46 (9.30 to 11.53) | 7.28% (-4.71% to 20.21%) | 89.91 (78.97 to 100.91) | 7.99 (7.03 to 8.91) | 9.04% (-2.50% to 21.21%) |
| **Maryland** | 375.70 (342.68 to 413.86) | 9.45 (8.56 to 10.42) | 7.53% (-3.73% to 21.53%) | 343.91 (311.41 to 381.86) | 7.86 (7.07 to 8.80) | 13.53% (2.45% to 26.69%) |
| **Massachusetts** | 562.59 (519.91 to 607.83) | 11.77 (10.93 to 12.73) | 8.47% (-0.87% to 20.63%) | 489.37 (448.95 to 536.82) | 9.23 (8.47 to 10.03) | 14.31% (4.09% to 26.94%) |
| **Michigan** | 795.04 (739.44 to 862.97) | 11.08 (10.34 to 11.98) | 15.61% (5.39% to 28.14%) | 574.54 (532.10 to 622.23) | 7.82 (7.27 to 8.52) | 20.19% (8.81% to 34.87%) |
| **Minnesota** | 562.28 (510.01 to 616.37) | 14.36 (13.18 to 15.67) | 21.06% (8.78% to 37.48%) | 369.59 (323.47 to 416.75) | 8.87 (7.89 to 9.91) | 8.88% (-2.28% to 21.44%) |
| **Mississippi** | 165.25 (147.10 to 183.63) | 8.88 (7.95 to 9.82) | 22.66% (10.17% to 37.59%) | 97.36 (84.54 to 110.04) | 4.66 (4.06 to 5.30) | 7.06% (-4.63% to 18.09%) |
| **Missouri** | 387.07 (355.84 to 420.06) | 9.39 (8.64 to 10.18) | 10.28% (-2.61% to 25.92%) | 329.74 (305.12 to 358.21) | 7.45 (6.89 to 8.12) | 16.45% (3.91% to 31.32%) |
| **Montana** | 80.39 (71.66 to 90.78) | 10.24 (9.17 to 11.26) | 12.25% (-0.27% to 26.57%) | 63.62 (56.24 to 72.93) | 7.95 (7.02 to 9.06) | 17.18% (4.60% to 31.09%) |
| **Nebraska** | 135.69 (117.86 to 158.79) | 10.37 (8.97 to 11.95) | 12.68% (2.32% to 24.69%) | 107.93 (93.34 to 124.14) | 7.90 (6.89 to 9.06) | 21.40% (9.06% to 33.70%) |
| **Nevada** | 154.13 (137.92 to 172.94) | 7.66 (6.83 to 8.59) | 1.76% (-8.47% to 13.73%) | 125.01 (110.85 to 140.74) | 6.11 (5.41 to 6.91) | 8.90% (-1.28% to 20.95%) |
| **New Hampshire** | 124.64 (113.06 to 137.16) | 12.08 (10.97 to 13.20) | 12.09% (0.71% to 24.66%) | 94.09 (84.44 to 104.63) | 8.76 (7.85 to 9.74) | 15.81% (4.96% to 29.35%) |
| **New Jersey** | 603.87 (558.25 to 653.69) | 9.70 (9.00 to 10.48) | 3.66% (-7.86% to 15.30%) | 609.53 (569.16 to 661.20) | 9.08 (8.46 to 9.92) | 20.29% (7.68% to 35.98%) |
| **New Mexico** | 124.28 (112.53 to 137.21) | 8.55 (7.77 to 9.41) | 9.58% (-1.58% to 23.07%) | 87.97 (78.06 to 98.86) | 5.73 (5.12 to 6.40) | 7.35% (-2.86% to 18.42%) |
| **New York** | 1540.08 (1428.62 to 1665.87) | 11.60 (10.80 to 12.49) | 21.09% (8.53% to 35.45%) | 1250.65 (1163.30 to 1347.70) | 8.42 (7.85 to 9.11) | 19.94% (6.62% to 35.79%) |
| **North Carolina** | 656.84 (609.67 to 709.30) | 9.54 (8.89 to 10.25) | 8.76% (-3.14% to 23.21%) | 597.85 (552.98 to 647.11) | 7.74 (7.19 to 8.39) | 19.26% (5.97% to 35.60%) |
| **North Dakota** | 61.45 (51.72 to 72.27) | 11.44 (9.83 to 13.31) | 31.82% (19.62% to 44.76%) | 59.32 (50.34 to 68.69) | 10.73 (9.34 to 12.18) | 32.18% (21.01% to 45.14%) |
| **Ohio** | 800.59 (739.03 to 864.30) | 9.93 (9.17 to 10.70) | 8.27% (-2.09% to 20.78%) | 684.16 (633.76 to 739.57) | 7.94 (7.37 to 8.59) | 17.57% (4.57% to 33.12%) |
| **Oklahoma** | 214.39 (195.65 to 235.88) | 8.51 (7.77 to 9.32) | 9.49% (-2.72% to 23.51%) | 197.61 (180.90 to 217.71) | 7.30 (6.64 to 8.13) | 22.37% (8.83% to 36.42%) |
| **Oregon** | 338.45 (304.67 to 378.22) | 10.97 (9.97 to 11.99) | 4.23% (-7.34% to 18.36%) | 265.04 (235.20 to 297.06) | 8.02 (7.24 to 8.92) | 3.77% (-7.37% to 15.50%) |
| **Pennsylvania** | 1109.73 (1025.98 to 1206.31) | 12.30 (11.46 to 13.30) | 25.43% (10.87% to 42.81%) | 751.60 (691.83 to 814.99) | 7.59 (7.01 to 8.25) | 15.17% (1.50% to 30.19%) |
| **Rhode Island** | 73.68 (62.76 to 85.01) | 9.89 (8.53 to 11.30) | 13.92% (3.53% to 25.40%) | 63.39 (53.86 to 73.70) | 7.80 (6.55 to 8.96) | 20.51% (9.54% to 31.32%) |
| **South Carolina** | 334.66 (306.70 to 363.66) | 9.50 (8.82 to 10.29) | 12.01% (-0.10% to 24.90%) | 271.88 (248.08 to 298.95) | 6.95 (6.43 to 7.54) | 13.97% (2.46% to 30.11%) |
| **South Dakota** | 74.19 (64.03 to 85.69) | 12.18 (10.73 to 13.73) | 29.75% (19.26% to 42.92%) | 51.26 (43.57 to 59.09) | 8.32 (7.23 to 9.44) | 37.44% (24.75% to 51.70%) |
| **Tennessee** | 396.49 (364.41 to 433.71) | 8.84 (8.16 to 9.59) | 14.99% (3.05% to 27.74%) | 379.77 (349.85 to 416.29) | 7.65 (7.05 to 8.37) | 24.94% (10.75% to 40.71%) |
| **Texas** | 1890.70 (1759.27 to 2033.99) | 10.68 (9.94 to 11.51) | 18.07% (5.54% to 31.57%) | 1354.55 (1255.38 to 1458.25) | 7.05 (6.57 to 7.56) | 9.28% (-2.00% to 23.02%) |
| **Utah** | 215.55 (193.85 to 240.09) | 11.69 (10.55 to 12.99) | 10.79% (-1.10% to 24.40%) | 151.09 (133.12 to 170.38) | 7.80 (6.91 to 8.73) | 7.37% (-3.99% to 20.01%) |
| **Vermont** | 90.02 (77.75 to 104.54) | 17.79 (15.77 to 19.97) | 35.69% (23.07% to 47.72%) | 59.83 (51.90 to 68.96) | 11.82 (10.46 to 13.31) | 28.99% (17.71% to 41.11%) |
| **Virginia** | 507.24 (466.62 to 554.43) | 9.00 (8.28 to 9.81) | -2.81% (-12.42% to 8.87%) | 386.94 (355.17 to 424.32) | 6.41 (5.83 to 7.12) | -2.72% (-14.12% to 10.95%) |
| **Washington** | 559.88 (518.25 to 608.07) | 10.82 (10.04 to 11.75) | -6.07% (-16.25% to 7.16%) | 427.80 (389.93 to 473.17) | 7.78 (7.12 to 8.55) | -2.24% (-12.32% to 10.71%) |
| **West Virginia** | 134.26 (121.60 to 149.61) | 10.46 (9.55 to 11.42) | 29.65% (17.38% to 44.51%) | 102.82 (91.22 to 115.44) | 7.48 (6.65 to 8.37) | 32.80% (19.43% to 47.88%) |
| **Wisconsin** | 423.64 (389.32 to 463.92) | 10.30 (9.48 to 11.22) | 2.21% (-9.10% to 15.42%) | 351.61 (320.69 to 385.03) | 8.12 (7.47 to 8.90) | 11.21% (-1.33% to 25.62%) |
| **Wyoming** | 44.91 (38.06 to 51.80) | 10.98 (9.36 to 12.61) | 18.62% (8.57% to 31.98%) | 32.53 (27.44 to 37.87) | 8.06 (6.93 to 9.35) | 22.87% (11.71% to 33.57%) |

The numbers in parentheses represent the 95% uncertainty interval.

## Table S4b: Numbers and age-standardized rates of disability-adjusted life years (DALYs) in 2021, and percentage changes from 1990 to 2021, by sex

|  | **Male** | | | **Female** | | |
| --- | --- | --- | --- | --- | --- | --- |
|  | **Absolute number, 2021** | **Age-standardized rate, per 100,000 people, 2021** | **Percentage Change, 1990-2021** | **Absolute number, thousands, 2021** | **Age-standardized rate, per 100,000 people, 2021** | **Percentage Change, 1990-2021** |
| **United States of America** | 120100.60 (114052.88 to 125007.03) | 50.01 (47.66 to 51.99) | 6.43% (1.68% to 10.69%) | 90458.85 (83917.83 to 95383.87) | 33.45 (31.52 to 34.99) | -0.12% (-3.73% to 3.94%) |
| **Alabama** | 2062.61 (1632.11 to 2535.12) | 57.70 (46.37 to 70.18) | 31.86% (5.37% to 61.58%) | 1600.18 (1308.87 to 1907.81) | 38.95 (32.37 to 46.06) | 23.18% (2.15% to 48.65%) |
| **Alaska** | 246.27 (202.98 to 296.47) | 49.11 (40.52 to 58.46) | 4.09% (-14.60% to 25.32%) | 152.38 (132.34 to 175.36) | 32.67 (28.72 to 37.23) | -2.16% (-13.99% to 12.65%) |
| **Arizona** | 2834.10 (2304.33 to 3426.31) | 54.74 (44.82 to 65.58) | 15.57% (-5.58% to 40.43%) | 1947.50 (1585.55 to 2334.93) | 34.35 (28.44 to 40.74) | 5.48% (-13.07% to 27.43%) |
| **Arkansas** | 1275.22 (1016.92 to 1572.43) | 59.78 (47.92 to 72.64) | 21.71% (-2.49% to 51.19%) | 907.78 (738.94 to 1095.64) | 38.52 (31.56 to 46.08) | 21.93% (-0.71% to 46.15%) |
| **California** | 11561.78 (9487.91 to 13957.26) | 41.99 (34.80 to 50.60) | -9.59% (-25.85% to 8.94%) | 8255.42 (6800.11 to 9828.91) | 27.08 (22.77 to 31.83) | -18.65% (-31.60% to -4.04%) |
| **Colorado** | 2285.17 (1781.19 to 2850.49) | 55.20 (43.56 to 68.01) | 2.47% (-19.84% to 26.86%) | 1776.98 (1426.63 to 2152.01) | 38.34 (31.17 to 45.95) | -2.49% (-20.73% to 15.75%) |
| **Connecticut** | 1445.40 (1143.75 to 1802.88) | 51.75 (41.25 to 64.40) | 4.06% (-19.44% to 30.02%) | 1022.22 (812.19 to 1264.02) | 32.14 (25.88 to 39.02) | -6.79% (-23.74% to 14.33%) |
| **Delaware** | 398.98 (331.33 to 478.52) | 53.88 (45.46 to 64.07) | 15.09% (-5.17% to 37.54%) | 295.93 (258.50 to 344.89) | 34.30 (30.47 to 39.37) | 3.47% (-10.94% to 19.46%) |
| **District of Columbia** | 145.73 (117.00 to 178.43) | 36.04 (29.21 to 43.88) | -46.93% (-57.22% to -34.59%) | 115.79 (98.47 to 137.23) | 25.83 (22.09 to 30.31) | -49.25% (-58.26% to -36.26%) |
| **Florida** | 8012.20 (6440.49 to 9791.42) | 46.96 (38.29 to 56.66) | 1.91% (-18.20% to 23.26%) | 5852.05 (4796.83 to 6997.01) | 30.12 (25.24 to 35.73) | -1.87% (-17.72% to 16.06%) |
| **Georgia** | 3348.54 (2664.70 to 4096.42) | 46.44 (37.16 to 56.27) | 7.77% (-14.00% to 30.89%) | 2733.28 (2245.69 to 3277.28) | 32.72 (27.16 to 38.70) | 3.42% (-14.33% to 22.79%) |
| **Hawaii** | 459.72 (360.44 to 573.72) | 41.71 (33.26 to 51.05) | 8.79% (-14.56% to 35.34%) | 288.24 (241.74 to 339.57) | 24.55 (20.93 to 28.37) | -3.71% (-16.45% to 10.80%) |
| **Idaho** | 783.47 (606.55 to 976.65) | 58.93 (46.55 to 72.98) | 13.48% (-10.48% to 41.65%) | 580.97 (498.90 to 685.10) | 40.41 (35.04 to 47.24) | 2.51% (-11.98% to 20.38%) |
| **Illinois** | 4359.08 (3521.99 to 5313.49) | 47.75 (39.04 to 57.82) | 1.56% (-18.04% to 22.49%) | 3150.98 (2505.22 to 3840.28) | 30.75 (25.01 to 36.92) | -7.69% (-24.50% to 12.36%) |
| **Indiana** | 2808.11 (2261.46 to 3453.21) | 58.10 (47.50 to 70.47) | 18.98% (-3.43% to 45.18%) | 2153.19 (1778.90 to 2609.84) | 39.62 (32.79 to 47.37) | 20.68% (1.12% to 43.71%) |
| **Iowa** | 1309.42 (1016.78 to 1607.84) | 54.77 (42.99 to 66.64) | 18.27% (-6.95% to 44.07%) | 993.21 (792.88 to 1209.46) | 37.73 (30.60 to 45.08) | 22.78% (-1.58% to 49.79%) |
| **Kansas** | 1217.38 (972.46 to 1495.63) | 58.70 (47.60 to 71.48) | 14.22% (-8.97% to 40.95%) | 963.49 (778.20 to 1170.61) | 41.68 (34.00 to 50.61) | 23.27% (0.38% to 51.38%) |
| **Kentucky** | 1799.16 (1439.55 to 2206.16) | 54.92 (44.10 to 67.16) | 22.39% (-3.51% to 51.05%) | 1382.08 (1123.21 to 1698.72) | 37.31 (30.69 to 45.35) | 22.01% (-0.23% to 50.08%) |
| **Louisiana** | 1744.84 (1402.26 to 2143.31) | 54.71 (44.65 to 66.63) | 17.98% (-4.62% to 44.47%) | 1342.25 (1082.23 to 1622.88) | 37.49 (30.66 to 44.92) | 11.55% (-8.83% to 33.47%) |
| **Maine** | 725.66 (566.01 to 904.29) | 62.32 (49.53 to 76.99) | 22.66% (-4.21% to 51.50%) | 580.70 (483.04 to 696.04) | 42.60 (35.75 to 50.25) | 13.33% (-4.88% to 33.41%) |
| **Maryland** | 2124.79 (1677.81 to 2597.72) | 48.81 (39.16 to 59.21) | 6.40% (-16.33% to 31.02%) | 1738.46 (1407.69 to 2122.87) | 34.17 (28.25 to 41.33) | -0.35% (-17.68% to 20.47%) |
| **Massachusetts** | 2609.34 (2015.43 to 3298.65) | 48.83 (38.29 to 61.41) | -1.29% (-21.54% to 24.62%) | 2054.84 (1619.17 to 2531.20) | 32.48 (25.94 to 39.41) | -11.08% (-28.58% to 9.42%) |
| **Michigan** | 4289.15 (3437.83 to 5222.46) | 55.35 (44.76 to 66.86) | 16.93% (-5.90% to 40.06%) | 3055.82 (2522.98 to 3629.46) | 35.48 (29.68 to 42.08) | 4.86% (-12.70% to 24.44%) |
| **Minnesota** | 2780.71 (2175.42 to 3467.39) | 64.11 (50.54 to 78.91) | 14.51% (-9.48% to 42.30%) | 2004.22 (1627.57 to 2449.47) | 42.63 (35.10 to 51.50) | 4.14% (-14.52% to 26.01%) |
| **Mississippi** | 1070.48 (844.66 to 1308.15) | 53.52 (43.03 to 64.79) | 25.87% (1.66% to 51.29%) | 785.07 (643.42 to 944.86) | 33.87 (28.04 to 40.44) | 15.16% (-4.50% to 38.53%) |
| **Missouri** | 2651.60 (2096.88 to 3289.07) | 58.96 (47.33 to 71.83) | 27.61% (1.54% to 58.22%) | 1965.05 (1617.20 to 2353.59) | 38.37 (31.92 to 45.64) | 10.40% (-8.21% to 32.99%) |
| **Montana** | 542.96 (425.78 to 680.99) | 61.66 (49.07 to 76.28) | 21.48% (-1.72% to 52.22%) | 373.64 (319.87 to 431.29) | 40.44 (35.27 to 46.19) | 6.22% (-7.85% to 24.01%) |
| **Nebraska** | 815.51 (651.24 to 1007.78) | 57.78 (46.47 to 71.07) | 13.22% (-9.72% to 39.78%) | 570.47 (475.05 to 667.64) | 37.73 (31.83 to 43.94) | 13.56% (-3.59% to 33.93%) |
| **Nevada** | 913.85 (723.13 to 1129.61) | 40.78 (32.57 to 49.94) | 2.67% (-17.23% to 27.14%) | 645.60 (560.79 to 745.37) | 27.09 (23.70 to 31.05) | -4.03% (-16.65% to 10.86%) |
| **New Hampshire** | 610.20 (474.63 to 766.79) | 52.51 (41.39 to 64.87) | 1.38% (-20.50% to 27.74%) | 421.84 (348.32 to 510.02) | 32.65 (27.25 to 38.89) | -7.39% (-22.26% to 11.70%) |
| **New Jersey** | 2636.34 (2082.34 to 3303.93) | 38.53 (30.73 to 47.62) | -13.30% (-31.23% to 8.01%) | 2266.04 (1805.28 to 2753.96) | 28.33 (22.93 to 34.30) | -12.67% (-29.46% to 6.20%) |
| **New Mexico** | 788.10 (622.04 to 966.20) | 51.82 (41.46 to 63.50) | 30.22% (2.35% to 61.29%) | 574.07 (464.80 to 691.42) | 33.39 (27.30 to 39.55) | 17.51% (-3.64% to 41.41%) |
| **New York** | 5878.81 (4737.64 to 7097.34) | 40.02 (32.53 to 47.62) | -6.71% (-24.57% to 13.03%) | 4598.79 (3748.06 to 5534.53) | 26.80 (21.86 to 32.02) | -11.90% (-26.94% to 6.07%) |
| **North Carolina** | 4036.25 (3227.52 to 4927.60) | 54.07 (43.66 to 65.60) | 6.35% (-15.24% to 29.96%) | 3169.23 (2558.21 to 3811.19) | 36.39 (29.81 to 43.17) | 13.66% (-6.14% to 36.31%) |
| **North Dakota** | 300.69 (246.94 to 357.35) | 53.63 (44.33 to 63.16) | 7.54% (-11.63% to 28.39%) | 208.58 (177.36 to 243.75) | 36.79 (31.51 to 42.56) | 4.31% (-11.03% to 21.52%) |
| **Ohio** | 4777.07 (3888.96 to 5808.60) | 54.40 (44.66 to 65.64) | 10.36% (-8.98% to 33.94%) | 3977.87 (3283.28 to 4799.87) | 39.76 (33.06 to 47.18) | 16.78% (-3.55% to 39.94%) |
| **Oklahoma** | 1604.93 (1292.55 to 1972.30) | 58.56 (47.77 to 71.21) | 34.02% (9.32% to 64.06%) | 1186.34 (991.07 to 1413.74) | 38.83 (32.59 to 45.98) | 22.59% (2.10% to 45.88%) |
| **Oregon** | 2118.41 (1672.26 to 2611.07) | 62.46 (49.93 to 76.52) | 16.84% (-6.12% to 45.77%) | 1535.73 (1234.64 to 1898.84) | 40.58 (32.94 to 49.75) | -2.58% (-20.80% to 19.76%) |
| **Pennsylvania** | 5521.67 (4440.26 to 6891.81) | 54.71 (44.37 to 67.32) | 16.36% (-7.13% to 43.56%) | 3936.10 (3187.87 to 4767.52) | 34.44 (28.10 to 41.56) | -1.00% (-18.59% to 18.87%) |
| **Rhode Island** | 357.16 (279.57 to 448.61) | 42.69 (33.85 to 53.02) | -10.61% (-28.74% to 11.58%) | 298.66 (244.28 to 360.39) | 30.69 (25.54 to 36.40) | -9.33% (-23.93% to 8.12%) |
| **South Carolina** | 2010.44 (1594.68 to 2461.98) | 53.47 (42.99 to 64.70) | 15.06% (-7.50% to 39.59%) | 1517.81 (1234.52 to 1822.44) | 34.65 (28.65 to 41.40) | 0.57% (-18.04% to 21.41%) |
| **South Dakota** | 372.91 (312.83 to 444.19) | 58.04 (49.31 to 68.64) | 17.00% (-2.18% to 40.29%) | 266.63 (230.70 to 305.12) | 39.50 (34.78 to 45.05) | 10.42% (-4.65% to 28.16%) |
| **Tennessee** | 2818.80 (2246.96 to 3491.10) | 56.73 (46.26 to 69.64) | 23.86% (-0.76% to 52.16%) | 2211.99 (1817.76 to 2663.77) | 38.85 (32.33 to 46.38) | 17.46% (-3.57% to 43.12%) |
| **Texas** | 8181.91 (6625.33 to 9838.08) | 43.33 (35.50 to 51.74) | -0.20% (-18.68% to 18.54%) | 6121.83 (5094.40 to 7308.40) | 29.36 (24.72 to 34.59) | -9.49% (-24.31% to 8.35%) |
| **Utah** | 1032.74 (822.88 to 1281.41) | 54.88 (43.97 to 67.70) | 5.50% (-15.52% to 29.69%) | 766.52 (654.58 to 884.84) | 37.84 (32.58 to 43.60) | 2.49% (-11.46% to 19.84%) |
| **Vermont** | 320.16 (261.88 to 387.88) | 59.15 (49.20 to 70.91) | 4.25% (-14.03% to 25.99%) | 247.98 (213.99 to 289.11) | 41.50 (35.94 to 47.49) | -6.79% (-19.45% to 6.77%) |
| **Virginia** | 3209.21 (2562.29 to 3926.38) | 51.49 (41.57 to 62.46) | 2.24% (-17.74% to 23.35%) | 2452.13 (1986.67 to 2951.78) | 35.18 (28.87 to 41.68) | 0.49% (-17.14% to 22.22%) |
| **Washington** | 3266.31 (2616.46 to 3930.77) | 56.22 (45.43 to 67.56) | 1.05% (-19.37% to 22.09%) | 2552.16 (2072.21 to 3114.84) | 39.87 (32.74 to 48.08) | -4.74% (-20.82% to 14.53%) |
| **West Virginia** | 902.62 (700.88 to 1100.64) | 63.43 (50.55 to 76.48) | 39.02% (10.70% to 68.25%) | 608.36 (491.36 to 746.63) | 38.97 (31.77 to 47.11) | 42.41% (15.21% to 73.59%) |
| **Wisconsin** | 2487.41 (1967.12 to 3113.15) | 54.89 (44.11 to 67.77) | 14.92% (-10.44% to 40.29%) | 2064.28 (1678.97 to 2504.49) | 41.73 (34.49 to 50.09) | 8.61% (-10.41% to 31.31%) |
| **Wyoming** | 247.24 (208.59 to 300.99) | 56.33 (47.67 to 67.75) | 16.73% (-2.88% to 42.63%) | 188.09 (160.90 to 216.75) | 42.22 (36.36 to 48.34) | 12.38% (-1.98% to 29.08%) |

The numbers in parentheses represent the 95% uncertainty interval.

## Table S4c: Numbers and age-standardized rates of deaths in 2021, and percentage changes from 1990 to 2021, by sex

|  | **Male** | | | **Female** | | |
| --- | --- | --- | --- | --- | --- | --- |
|  | **Absolute number, 2021** | **Age-standardized rate, per 100,000 people, 2021** | **Percentage Change, 1990-2021** | **Absolute number, thousands, 2021** | **Age-standardized rate, per 100,000 people, 2021** | **Percentage Change, 1990-2021** |
| **United States of America** | 4705.32 (4440.84 to 4912.11) | 1.80 (1.71 to 1.88) | 19.01% (13.35% to 24.06%) | 3760.24 (3366.24 to 4020.17) | 1.21 (1.10 to 1.29) | 14.50% (9.28% to 20.47%) |
| **Alabama** | 78.37 (61.66 to 96.28) | 2.01 (1.59 to 2.46) | 46.01% (14.70% to 79.57%) | 65.74 (52.82 to 78.66) | 1.38 (1.11 to 1.66) | 36.55% (11.76% to 66.18%) |
| **Alaska** | 8.97 (7.36 to 10.95) | 1.70 (1.41 to 2.06) | 13.13% (-8.11% to 38.40%) | 5.57 (4.70 to 6.47) | 1.07 (0.91 to 1.24) | 1.38% (-12.55% to 18.17%) |
| **Arizona** | 111.17 (89.59 to 135.64) | 1.92 (1.56 to 2.33) | 33.50% (6.16% to 64.57%) | 79.81 (63.99 to 96.49) | 1.20 (0.96 to 1.44) | 22.84% (-0.57% to 49.73%) |
| **Arkansas** | 48.74 (38.20 to 60.91) | 2.07 (1.63 to 2.58) | 32.58% (3.29% to 66.24%) | 36.31 (29.27 to 43.94) | 1.32 (1.07 to 1.60) | 35.45% (8.47% to 65.00%) |
| **California** | 446.49 (360.10 to 548.53) | 1.52 (1.23 to 1.87) | 2.48% (-17.17% to 25.82%) | 342.52 (275.12 to 413.23) | 1.00 (0.81 to 1.20) | -3.12% (-20.32% to 15.55%) |
| **Colorado** | 86.67 (67.06 to 108.71) | 1.96 (1.52 to 2.46) | 11.65% (-13.08% to 39.29%) | 74.45 (58.88 to 90.20) | 1.45 (1.15 to 1.75) | 13.05% (-8.93% to 36.89%) |
| **Connecticut** | 58.01 (45.47 to 72.52) | 1.90 (1.49 to 2.38) | 12.73% (-12.73% to 42.07%) | 43.72 (34.47 to 54.77) | 1.18 (0.92 to 1.47) | 1.57% (-18.57% to 27.56%) |
| **Delaware** | 15.60 (12.85 to 18.89) | 1.87 (1.55 to 2.26) | 21.14% (-1.67% to 46.89%) | 12.42 (10.49 to 14.64) | 1.21 (1.04 to 1.42) | 11.20% (-5.18% to 31.04%) |
| **District of Columbia** | 5.06 (4.04 to 6.24) | 1.20 (0.96 to 1.47) | -25.07% (-41.27% to -5.78%) | 4.28 (3.53 to 5.16) | 0.85 (0.72 to 1.02) | -25.38% (-38.56% to -8.81%) |
| **Florida** | 321.15 (254.74 to 394.22) | 1.65 (1.31 to 2.01) | 20.46% (-5.11% to 46.86%) | 247.24 (195.03 to 300.90) | 1.06 (0.86 to 1.28) | 22.04% (-0.11% to 47.81%) |
| **Georgia** | 129.37 (101.62 to 159.15) | 1.69 (1.33 to 2.07) | 22.84% (-2.84% to 50.77%) | 111.42 (90.33 to 134.68) | 1.19 (0.97 to 1.43) | 18.39% (-4.76% to 44.03%) |
| **Hawaii** | 18.10 (14.23 to 22.73) | 1.44 (1.13 to 1.80) | 22.88% (-4.79% to 55.51%) | 11.52 (9.29 to 13.84) | 0.81 (0.66 to 0.97) | 8.12% (-8.62% to 28.46%) |
| **Idaho** | 31.79 (24.87 to 40.21) | 2.19 (1.72 to 2.76) | 27.21% (-1.77% to 61.49%) | 24.62 (20.82 to 29.15) | 1.52 (1.29 to 1.80) | 16.84% (-1.29% to 38.47%) |
| **Illinois** | 171.56 (136.80 to 211.24) | 1.75 (1.40 to 2.14) | 17.00% (-6.74% to 42.70%) | 131.25 (101.36 to 162.93) | 1.11 (0.87 to 1.37) | 10.82% (-11.38% to 37.40%) |
| **Indiana** | 109.86 (87.38 to 136.44) | 2.11 (1.69 to 2.60) | 29.65% (3.76% to 60.92%) | 89.00 (72.72 to 108.20) | 1.44 (1.18 to 1.75) | 34.30% (11.31% to 64.59%) |
| **Iowa** | 53.47 (41.54 to 65.95) | 2.02 (1.57 to 2.48) | 28.48% (-1.17% to 58.61%) | 42.97 (33.58 to 52.69) | 1.40 (1.10 to 1.71) | 37.46% (6.80% to 70.23%) |
| **Kansas** | 48.36 (37.73 to 60.05) | 2.14 (1.68 to 2.65) | 25.57% (-1.47% to 57.16%) | 40.59 (32.54 to 49.54) | 1.54 (1.25 to 1.89) | 40.30% (11.38% to 74.52%) |
| **Kentucky** | 67.71 (53.64 to 83.81) | 1.91 (1.52 to 2.37) | 30.73% (2.00% to 62.41%) | 56.72 (45.79 to 69.73) | 1.35 (1.09 to 1.66) | 32.99% (6.11% to 66.95%) |
| **Louisiana** | 64.85 (51.07 to 80.15) | 1.89 (1.50 to 2.34) | 32.31% (4.62% to 64.74%) | 52.82 (42.32 to 64.36) | 1.30 (1.04 to 1.58) | 23.85% (-0.82% to 51.97%) |
| **Maine** | 29.45 (22.87 to 36.74) | 2.22 (1.72 to 2.76) | 24.38% (-2.63% to 54.77%) | 25.52 (20.90 to 30.65) | 1.60 (1.32 to 1.92) | 24.91% (4.60% to 50.46%) |
| **Maryland** | 82.06 (63.83 to 100.94) | 1.75 (1.38 to 2.15) | 16.25% (-9.43% to 45.85%) | 70.75 (55.91 to 87.14) | 1.23 (0.98 to 1.50) | 10.66% (-9.87% to 36.29%) |
| **Massachusetts** | 105.75 (81.91 to 133.34) | 1.85 (1.43 to 2.32) | 10.30% (-13.59% to 40.63%) | 91.18 (70.42 to 113.08) | 1.27 (0.99 to 1.57) | 3.11% (-18.13% to 28.23%) |
| **Michigan** | 168.66 (133.96 to 205.00) | 1.97 (1.58 to 2.38) | 28.99% (2.45% to 56.98%) | 126.68 (102.33 to 150.72) | 1.26 (1.03 to 1.51) | 15.96% (-5.55% to 40.45%) |
| **Minnesota** | 113.76 (86.10 to 143.83) | 2.42 (1.85 to 3.05) | 25.73% (-2.87% to 59.14%) | 86.77 (68.88 to 107.62) | 1.62 (1.29 to 2.01) | 14.84% (-7.26% to 42.33%) |
| **Mississippi** | 40.02 (31.42 to 49.27) | 1.84 (1.46 to 2.24) | 39.07% (10.06% to 69.95%) | 31.45 (25.61 to 38.12) | 1.18 (0.96 to 1.41) | 29.78% (6.04% to 57.27%) |
| **Missouri** | 104.76 (82.54 to 129.98) | 2.11 (1.67 to 2.61) | 40.26% (11.78% to 74.64%) | 82.02 (66.80 to 99.24) | 1.39 (1.14 to 1.67) | 22.20% (-1.02% to 49.43%) |
| **Montana** | 21.90 (16.92 to 27.61) | 2.17 (1.69 to 2.71) | 28.71% (2.11% to 64.11%) | 15.88 (13.36 to 18.65) | 1.44 (1.23 to 1.68) | 16.20% (-1.60% to 37.10%) |
| **Nebraska** | 32.82 (26.08 to 40.87) | 2.15 (1.71 to 2.67) | 22.23% (-4.13% to 53.24%) | 23.90 (19.60 to 28.33) | 1.37 (1.13 to 1.62) | 25.63% (4.37% to 49.49%) |
| **Nevada** | 35.38 (27.84 to 44.19) | 1.47 (1.16 to 1.82) | 15.27% (-9.57% to 45.22%) | 26.34 (22.45 to 30.74) | 0.99 (0.84 to 1.14) | 9.62% (-6.45% to 28.43%) |
| **New Hampshire** | 24.72 (19.00 to 31.11) | 1.94 (1.50 to 2.42) | 6.83% (-18.32% to 36.73%) | 18.13 (14.70 to 22.44) | 1.22 (1.00 to 1.50) | -1.46% (-20.38% to 22.50%) |
| **New Jersey** | 104.33 (81.61 to 131.80) | 1.43 (1.11 to 1.80) | -2.00% (-23.28% to 23.11%) | 94.77 (73.51 to 117.16) | 1.04 (0.82 to 1.28) | -0.14% (-20.50% to 24.48%) |
| **New Mexico** | 30.43 (23.76 to 37.33) | 1.77 (1.39 to 2.17) | 43.13% (11.52% to 78.94%) | 23.50 (18.70 to 28.81) | 1.17 (0.94 to 1.42) | 31.20% (5.83% to 59.06%) |
| **New York** | 228.77 (181.31 to 279.07) | 1.45 (1.15 to 1.76) | 10.22% (-13.37% to 35.37%) | 189.57 (151.71 to 231.79) | 0.97 (0.78 to 1.18) | 7.36% (-13.90% to 32.89%) |
| **North Carolina** | 158.68 (126.38 to 194.20) | 1.95 (1.55 to 2.37) | 17.47% (-8.29% to 47.09%) | 131.98 (104.78 to 160.59) | 1.32 (1.05 to 1.59) | 29.32% (3.75% to 57.14%) |
| **North Dakota** | 11.72 (9.43 to 14.07) | 1.87 (1.51 to 2.23) | 11.69% (-10.31% to 34.72%) | 8.40 (6.98 to 10.05) | 1.24 (1.04 to 1.46) | 11.18% (-7.68% to 31.89%) |
| **Ohio** | 187.60 (151.26 to 228.98) | 1.95 (1.58 to 2.37) | 20.08% (-2.38% to 46.60%) | 164.40 (134.08 to 199.67) | 1.42 (1.16 to 1.72) | 28.96% (4.09% to 56.49%) |
| **Oklahoma** | 61.93 (49.78 to 76.10) | 2.08 (1.68 to 2.54) | 48.12% (19.62% to 82.20%) | 48.81 (40.40 to 58.72) | 1.40 (1.16 to 1.68) | 39.37% (14.61% to 69.33%) |
| **Oregon** | 86.54 (67.28 to 107.42) | 2.31 (1.80 to 2.85) | 31.11% (3.53% to 64.48%) | 66.93 (52.75 to 83.83) | 1.53 (1.22 to 1.92) | 9.66% (-12.29% to 36.04%) |
| **Pennsylvania** | 218.18 (172.78 to 271.58) | 1.96 (1.57 to 2.43) | 26.26% (-1.12% to 57.96%) | 165.78 (134.23 to 200.24) | 1.25 (1.01 to 1.51) | 9.76% (-10.65% to 35.28%) |
| **Rhode Island** | 14.31 (11.22 to 18.02) | 1.57 (1.24 to 1.98) | -3.05% (-24.28% to 21.54%) | 12.95 (10.51 to 15.81) | 1.15 (0.93 to 1.39) | 2.30% (-15.08% to 24.29%) |
| **South Carolina** | 78.39 (61.56 to 95.98) | 1.88 (1.49 to 2.30) | 28.13% (2.07% to 56.90%) | 61.74 (50.03 to 75.17) | 1.22 (1.00 to 1.48) | 15.72% (-6.28% to 40.91%) |
| **South Dakota** | 14.70 (12.15 to 17.93) | 2.03 (1.68 to 2.44) | 26.35% (4.27% to 54.70%) | 11.04 (9.26 to 12.74) | 1.37 (1.16 to 1.58) | 20.55% (2.37% to 41.70%) |
| **Tennessee** | 108.59 (85.50 to 135.27) | 2.00 (1.59 to 2.47) | 34.61% (5.21% to 66.06%) | 90.45 (73.81 to 110.73) | 1.38 (1.13 to 1.68) | 30.47% (4.92% to 62.27%) |
| **Texas** | 309.46 (246.89 to 374.58) | 1.57 (1.26 to 1.90) | 12.80% (-9.63% to 35.95%) | 244.60 (199.93 to 294.88) | 1.06 (0.88 to 1.28) | 4.93% (-13.43% to 27.38%) |
| **Utah** | 39.88 (31.27 to 50.13) | 2.05 (1.62 to 2.57) | 18.95% (-7.06% to 47.76%) | 31.47 (26.55 to 36.75) | 1.44 (1.22 to 1.68) | 20.71% (2.63% to 40.67%) |
| **Vermont** | 12.77 (10.38 to 15.54) | 2.09 (1.72 to 2.53) | 5.57% (-15.44% to 29.26%) | 10.63 (8.96 to 12.56) | 1.50 (1.28 to 1.76) | 0.37% (-14.62% to 17.52%) |
| **Virginia** | 129.00 (102.32 to 158.84) | 1.92 (1.53 to 2.36) | 14.85% (-9.14% to 40.64%) | 101.29 (80.94 to 124.17) | 1.28 (1.03 to 1.56) | 11.46% (-10.48% to 37.88%) |
| **Washington** | 128.87 (103.22 to 156.33) | 2.05 (1.65 to 2.48) | 9.05% (-14.55% to 34.96%) | 108.06 (85.92 to 133.23) | 1.50 (1.20 to 1.84) | 6.26% (-13.57% to 31.10%) |
| **West Virginia** | 35.15 (27.22 to 43.34) | 2.17 (1.68 to 2.66) | 45.46% (13.33% to 78.83%) | 24.90 (20.16 to 30.47) | 1.35 (1.08 to 1.65) | 52.88% (20.63% to 88.77%) |
| **Wisconsin** | 101.71 (80.15 to 126.97) | 2.05 (1.62 to 2.55) | 25.32% (-3.46% to 55.75%) | 89.51 (72.13 to 108.98) | 1.56 (1.27 to 1.90) | 19.08% (-3.16% to 45.13%) |
| **Wyoming** | 9.75 (8.15 to 11.89) | 2.01 (1.69 to 2.44) | 26.71% (4.47% to 56.04%) | 7.84 (6.59 to 9.22) | 1.54 (1.30 to 1.80) | 25.21% (6.94% to 46.46%) |

The numbers in parentheses represent the 95% uncertainty interval.

## Table S4d: Numbers and age-standardized rates of incidence in 2021, and percentage changes from 1990 to 2021, by sex

|  | **Male** | | | **Female** | | |
| --- | --- | --- | --- | --- | --- | --- |
|  | **Absolute number, 2021** | **Age-standardized rate, per 100,000 people, 2021** | **Percentage Change, 1990-2021** | **Absolute number, thousands, 2021** | **Age-standardized rate, per 100,000 people, 2021** | **Percentage Change, 1990-2021** |
| **United States of America** | 6154.30 (5915.24 to 6410.01) | 2.46 (2.37 to 2.57) | 15.92% (12.18% to 19.91%) | 4934.71 (4719.20 to 5152.02) | 1.70 (1.63 to 1.78) | 11.93% (8.42% to 15.89%) |
| **Alabama** | 88.03 (84.14 to 92.06) | 2.36 (2.26 to 2.47) | 29.05% (22.69% to 35.57%) | 74.99 (71.15 to 78.94) | 1.70 (1.62 to 1.79) | 25.61% (18.69% to 32.88%) |
| **Alaska** | 12.42 (11.67 to 13.29) | 2.40 (2.28 to 2.55) | 14.94% (10.06% to 20.36%) | 9.88 (9.23 to 10.52) | 1.99 (1.88 to 2.12) | 13.08% (8.01% to 17.86%) |
| **Arizona** | 135.54 (129.92 to 141.59) | 2.48 (2.38 to 2.59) | 17.84% (12.41% to 23.04%) | 105.91 (100.91 to 111.58) | 1.70 (1.62 to 1.79) | 11.78% (5.94% to 17.71%) |
| **Arkansas** | 60.03 (56.88 to 62.91) | 2.70 (2.56 to 2.83) | 26.36% (20.51% to 32.88%) | 46.93 (44.33 to 49.82) | 1.82 (1.73 to 1.93) | 30.62% (23.25% to 38.31%) |
| **California** | 595.22 (567.84 to 622.47) | 2.11 (2.01 to 2.20) | 0.59% (-3.86% to 5.34%) | 462.44 (440.60 to 486.64) | 1.43 (1.36 to 1.50) | -4.08% (-8.85% to 0.95%) |
| **Colorado** | 130.73 (124.61 to 136.68) | 3.03 (2.90 to 3.16) | 25.93% (19.67% to 31.96%) | 97.87 (92.84 to 103.03) | 1.99 (1.89 to 2.08) | 13.05% (7.39% to 18.83%) |
| **Connecticut** | 76.26 (72.39 to 80.05) | 2.64 (2.50 to 2.76) | 16.48% (10.93% to 22.36%) | 60.68 (57.43 to 64.09) | 1.77 (1.67 to 1.86) | 9.35% (4.19% to 14.88%) |
| **Delaware** | 21.61 (20.53 to 22.82) | 2.80 (2.66 to 2.93) | 23.33% (17.35% to 28.68%) | 15.42 (14.53 to 16.30) | 1.68 (1.58 to 1.77) | 7.68% (2.89% to 13.04%) |
| **District of Columbia** | 7.27 (6.78 to 7.79) | 1.82 (1.71 to 1.94) | -2.71% (-6.36% to 0.59%) | 6.99 (6.54 to 7.43) | 1.50 (1.41 to 1.59) | 9.58% (4.83% to 14.66%) |
| **Florida** | 400.98 (382.04 to 417.88) | 2.19 (2.09 to 2.28) | 10.95% (5.70% to 16.60%) | 321.37 (305.07 to 337.80) | 1.52 (1.44 to 1.60) | 6.13% (0.46% to 11.94%) |
| **Georgia** | 171.01 (163.47 to 179.39) | 2.33 (2.23 to 2.43) | 19.25% (13.76% to 24.97%) | 134.22 (127.49 to 141.61) | 1.51 (1.43 to 1.59) | 9.63% (4.77% to 15.46%) |
| **Hawaii** | 24.31 (22.89 to 25.80) | 2.10 (1.98 to 2.23) | 22.73% (17.47% to 28.27%) | 18.06 (16.86 to 19.33) | 1.47 (1.38 to 1.58) | 13.81% (7.98% to 20.63%) |
| **Idaho** | 40.67 (38.65 to 42.68) | 2.94 (2.80 to 3.07) | 21.42% (15.85% to 27.79%) | 31.47 (29.69 to 33.18) | 2.03 (1.92 to 2.14) | 12.23% (7.60% to 17.55%) |
| **Illinois** | 223.88 (213.96 to 234.26) | 2.37 (2.27 to 2.48) | 14.11% (8.96% to 19.16%) | 181.91 (171.73 to 190.52) | 1.65 (1.56 to 1.73) | 7.36% (2.12% to 12.53%) |
| **Indiana** | 137.95 (131.55 to 144.60) | 2.74 (2.62 to 2.86) | 24.76% (19.32% to 30.77%) | 107.29 (101.97 to 112.48) | 1.84 (1.75 to 1.93) | 20.41% (14.77% to 27.24%) |
| **Iowa** | 69.86 (66.35 to 73.61) | 2.72 (2.58 to 2.85) | 25.63% (19.68% to 31.24%) | 54.19 (51.30 to 57.21) | 1.87 (1.78 to 1.97) | 19.38% (14.29% to 25.78%) |
| **Kansas** | 62.61 (59.50 to 65.84) | 2.90 (2.77 to 3.04) | 28.79% (23.40% to 34.87%) | 47.45 (44.86 to 50.02) | 1.92 (1.82 to 2.03) | 26.27% (19.34% to 32.61%) |
| **Kentucky** | 92.67 (87.98 to 96.49) | 2.70 (2.58 to 2.82) | 32.82% (26.39% to 39.88%) | 67.40 (63.96 to 70.89) | 1.69 (1.60 to 1.77) | 22.46% (15.75% to 29.95%) |
| **Louisiana** | 82.07 (77.76 to 86.17) | 2.49 (2.37 to 2.61) | 24.16% (17.96% to 30.91%) | 65.81 (62.35 to 69.31) | 1.74 (1.66 to 1.84) | 18.03% (11.93% to 25.23%) |
| **Maine** | 38.98 (36.97 to 41.11) | 3.02 (2.87 to 3.16) | 21.14% (15.62% to 26.63%) | 33.78 (31.98 to 35.81) | 2.24 (2.13 to 2.36) | 24.63% (19.27% to 30.57%) |
| **Maryland** | 113.45 (108.12 to 118.99) | 2.52 (2.40 to 2.63) | 19.77% (14.44% to 25.93%) | 97.20 (92.49 to 101.96) | 1.81 (1.71 to 1.89) | 13.12% (7.62% to 18.23%) |
| **Massachusetts** | 153.76 (146.38 to 161.06) | 2.79 (2.66 to 2.92) | 16.93% (12.05% to 21.99%) | 127.24 (120.78 to 133.71) | 1.94 (1.84 to 2.04) | 13.73% (8.61% to 19.21%) |
| **Michigan** | 223.10 (213.23 to 233.33) | 2.72 (2.62 to 2.84) | 19.13% (14.34% to 25.08%) | 177.62 (169.21 to 186.32) | 1.87 (1.78 to 1.96) | 18.03% (12.71% to 23.89%) |
| **Minnesota** | 149.27 (142.21 to 156.38) | 3.32 (3.17 to 3.46) | 26.38% (20.57% to 32.03%) | 116.86 (111.02 to 123.58) | 2.30 (2.18 to 2.42) | 21.67% (15.99% to 27.93%) |
| **Mississippi** | 48.56 (45.89 to 51.17) | 2.32 (2.20 to 2.43) | 25.91% (19.71% to 32.03%) | 37.06 (34.77 to 39.12) | 1.45 (1.37 to 1.53) | 18.57% (12.28% to 25.60%) |
| **Missouri** | 122.71 (117.45 to 128.43) | 2.58 (2.47 to 2.69) | 25.13% (19.31% to 32.04%) | 101.80 (96.68 to 106.86) | 1.84 (1.75 to 1.92) | 21.76% (15.27% to 28.20%) |
| **Montana** | 27.87 (26.25 to 29.40) | 2.90 (2.75 to 3.04) | 22.29% (17.00% to 27.48%) | 22.50 (21.26 to 23.79) | 2.18 (2.07 to 2.30) | 25.22% (19.76% to 30.78%) |
| **Nebraska** | 40.84 (38.76 to 43.18) | 2.77 (2.63 to 2.92) | 24.99% (19.95% to 30.68%) | 30.59 (28.86 to 32.42) | 1.87 (1.77 to 1.98) | 26.46% (20.74% to 32.49%) |
| **Nevada** | 41.56 (39.25 to 43.93) | 1.83 (1.73 to 1.93) | 1.77% (-2.57% to 6.18%) | 33.31 (31.39 to 35.34) | 1.35 (1.27 to 1.44) | 3.98% (-0.91% to 9.16%) |
| **New Hampshire** | 35.13 (33.30 to 37.08) | 2.88 (2.74 to 3.02) | 17.49% (12.08% to 22.87%) | 25.96 (24.50 to 27.38) | 1.89 (1.79 to 1.98) | 15.18% (10.10% to 20.83%) |
| **New Jersey** | 153.30 (145.60 to 160.31) | 2.18 (2.08 to 2.28) | 6.49% (2.06% to 11.61%) | 130.05 (123.68 to 137.09) | 1.56 (1.48 to 1.65) | 3.96% (-0.94% to 9.23%) |
| **New Mexico** | 36.84 (34.98 to 38.99) | 2.24 (2.13 to 2.35) | 20.51% (14.64% to 26.24%) | 28.62 (26.99 to 30.47) | 1.50 (1.41 to 1.59) | 16.41% (10.87% to 22.40%) |
| **New York** | 298.38 (284.01 to 311.62) | 2.00 (1.91 to 2.09) | 6.35% (1.45% to 11.84%) | 244.00 (231.28 to 256.43) | 1.37 (1.30 to 1.45) | 0.30% (-4.51% to 5.85%) |
| **North Carolina** | 194.59 (186.39 to 203.49) | 2.49 (2.38 to 2.60) | 16.49% (10.99% to 21.85%) | 168.91 (161.19 to 176.94) | 1.80 (1.71 to 1.88) | 18.86% (12.62% to 25.57%) |
| **North Dakota** | 17.99 (16.95 to 19.16) | 2.99 (2.82 to 3.16) | 34.91% (29.03% to 41.14%) | 14.81 (13.94 to 15.68) | 2.34 (2.21 to 2.47) | 35.58% (29.98% to 42.23%) |
| **Ohio** | 258.73 (248.43 to 270.41) | 2.77 (2.67 to 2.90) | 24.02% (18.36% to 29.61%) | 206.60 (196.04 to 215.79) | 1.89 (1.80 to 1.99) | 19.61% (13.77% to 26.26%) |
| **Oklahoma** | 73.41 (69.92 to 76.94) | 2.57 (2.45 to 2.68) | 28.86% (22.42% to 35.59%) | 59.93 (56.92 to 63.12) | 1.83 (1.75 to 1.92) | 26.10% (19.42% to 33.38%) |
| **Oregon** | 106.65 (101.48 to 112.33) | 2.97 (2.84 to 3.11) | 19.09% (14.29% to 23.63%) | 93.05 (88.40 to 97.72) | 2.24 (2.14 to 2.35) | 18.06% (12.74% to 24.17%) |
| **Pennsylvania** | 280.49 (268.47 to 293.72) | 2.67 (2.56 to 2.79) | 22.16% (15.73% to 28.51%) | 218.37 (207.40 to 228.99) | 1.76 (1.67 to 1.84) | 17.93% (12.08% to 24.26%) |
| **Rhode Island** | 21.58 (20.41 to 23.00) | 2.49 (2.36 to 2.64) | 20.86% (14.55% to 26.36%) | 18.56 (17.40 to 19.81) | 1.81 (1.69 to 1.92) | 19.36% (14.67% to 24.89%) |
| **South Carolina** | 98.17 (93.19 to 103.12) | 2.45 (2.33 to 2.56) | 15.34% (9.99% to 21.03%) | 81.90 (77.51 to 86.12) | 1.71 (1.62 to 1.80) | 15.84% (10.98% to 22.17%) |
| **South Dakota** | 20.67 (19.52 to 22.01) | 2.98 (2.82 to 3.14) | 31.42% (26.15% to 37.63%) | 15.13 (14.23 to 16.00) | 2.03 (1.92 to 2.14) | 32.02% (25.59% to 39.10%) |
| **Tennessee** | 134.72 (128.53 to 141.23) | 2.57 (2.46 to 2.69) | 28.19% (22.15% to 34.44%) | 115.79 (109.80 to 121.36) | 1.88 (1.79 to 1.98) | 28.01% (21.27% to 35.20%) |
| **Texas** | 421.54 (401.88 to 442.47) | 2.21 (2.12 to 2.32) | 9.69% (4.95% to 14.53%) | 341.03 (325.29 to 357.49) | 1.55 (1.49 to 1.63) | 6.93% (1.58% to 12.85%) |
| **Utah** | 56.71 (53.58 to 59.92) | 2.98 (2.83 to 3.14) | 18.20% (13.18% to 23.61%) | 44.25 (41.80 to 46.65) | 2.11 (2.00 to 2.22) | 18.10% (12.85% to 23.98%) |
| **Vermont** | 20.66 (19.49 to 21.98) | 3.54 (3.35 to 3.75) | 23.97% (18.89% to 29.34%) | 15.25 (14.32 to 16.25) | 2.36 (2.22 to 2.51) | 15.32% (10.71% to 20.32%) |
| **Virginia** | 159.97 (152.32 to 167.18) | 2.49 (2.37 to 2.60) | 10.48% (5.74% to 16.18%) | 128.48 (121.47 to 135.15) | 1.71 (1.62 to 1.79) | 8.10% (2.56% to 13.56%) |
| **Washington** | 179.12 (170.73 to 186.60) | 2.96 (2.83 to 3.07) | 11.97% (6.66% to 16.58%) | 141.11 (134.18 to 148.14) | 2.04 (1.95 to 2.14) | 7.28% (2.40% to 12.78%) |
| **West Virginia** | 41.26 (39.15 to 43.49) | 2.70 (2.57 to 2.82) | 33.56% (27.23% to 40.16%) | 31.19 (29.34 to 33.01) | 1.78 (1.68 to 1.88) | 32.73% (26.15% to 39.27%) |
| **Wisconsin** | 138.64 (132.28 to 145.41) | 2.87 (2.73 to 3.00) | 21.08% (15.70% to 26.43%) | 113.48 (108.12 to 119.02) | 2.10 (2.01 to 2.20) | 22.85% (17.37% to 29.23%) |
| **Wyoming** | 12.57 (11.81 to 13.43) | 2.71 (2.55 to 2.87) | 17.99% (13.59% to 23.28%) | 9.95 (9.35 to 10.55) | 2.05 (1.93 to 2.17) | 23.77% (18.23% to 29.61%) |

The numbers in parentheses represent the 95% uncertainty interval.

## Table S4e: Numbers and age-standardized rates of years lived with disability (YLDs) in 2021, and percentage changes from 1990 to 2021, by sex

|  | **Male** | | | **Female** | | |
| --- | --- | --- | --- | --- | --- | --- |
|  | **Absolute number, 2021** | **Age-standardized rate, per 100,000 people, 2021** | **Percentage Change, 1990-2021** | **Absolute number, thousands, 2021** | **Age-standardized rate, per 100,000 people, 2021** | **Percentage Change, 1990-2021** |
| **United States of America** | 4827.99 (3512.31 to 6332.66) | 2.19 (1.59 to 2.89) | 12.05% (2.03% to 23.33%) | 3769.07 (2733.16 to 4946.14) | 1.58 (1.15 to 2.08) | 12.57% (2.33% to 23.78%) |
| **Alabama** | 60.93 (44.51 to 81.41) | 1.86 (1.37 to 2.48) | 18.74% (6.32% to 33.23%) | 51.07 (37.05 to 67.85) | 1.44 (1.04 to 1.91) | 22.58% (8.16% to 38.00%) |
| **Alaska** | 10.23 (7.30 to 14.00) | 2.20 (1.56 to 2.97) | 26.45% (13.05% to 41.34%) | 9.14 (6.48 to 12.54) | 2.12 (1.52 to 2.90) | 37.54% (24.86% to 50.42%) |
| **Arizona** | 107.49 (77.46 to 141.43) | 2.24 (1.63 to 2.95) | 22.26% (8.33% to 38.49%) | 78.30 (55.75 to 103.70) | 1.54 (1.11 to 2.03) | 18.03% (3.96% to 35.51%) |
| **Arkansas** | 45.05 (32.28 to 58.91) | 2.29 (1.65 to 2.99) | 26.91% (11.69% to 43.48%) | 36.44 (26.08 to 48.89) | 1.71 (1.22 to 2.30) | 35.37% (20.16% to 51.94%) |
| **California** | 530.92 (382.27 to 708.06) | 2.08 (1.50 to 2.76) | 4.49% (-8.15% to 18.05%) | 382.49 (277.43 to 502.55) | 1.40 (1.02 to 1.83) | 2.53% (-8.48% to 16.56%) |
| **Colorado** | 112.13 (80.70 to 147.28) | 2.91 (2.11 to 3.77) | 24.71% (10.40% to 43.13%) | 60.94 (44.14 to 80.94) | 1.51 (1.09 to 2.01) | -2.41% (-13.12% to 10.12%) |
| **Connecticut** | 66.39 (47.83 to 87.80) | 2.63 (1.89 to 3.46) | 13.79% (1.28% to 28.17%) | 52.72 (37.69 to 68.85) | 1.90 (1.38 to 2.50) | 15.62% (4.22% to 28.82%) |
| **Delaware** | 21.11 (15.44 to 27.93) | 3.07 (2.21 to 4.06) | 40.38% (26.71% to 58.70%) | 11.66 (8.38 to 15.46) | 1.63 (1.17 to 2.18) | 16.62% (5.93% to 29.52%) |
| **District of Columbia** | 7.46 (5.27 to 10.03) | 1.97 (1.39 to 2.65) | 27.65% (16.04% to 40.51%) | 6.30 (4.45 to 8.63) | 1.43 (1.03 to 1.96) | 25.07% (13.60% to 39.28%) |
| **Florida** | 310.49 (223.16 to 406.98) | 1.94 (1.41 to 2.54) | 13.93% (0.26% to 28.42%) | 263.76 (191.61 to 346.87) | 1.53 (1.11 to 2.01) | 21.34% (7.35% to 37.54%) |
| **Georgia** | 130.51 (94.61 to 172.53) | 1.98 (1.44 to 2.61) | 11.19% (0.79% to 24.74%) | 89.91 (65.00 to 118.32) | 1.22 (0.89 to 1.60) | 2.88% (-6.72% to 13.93%) |
| **Hawaii** | 20.73 (14.90 to 28.10) | 2.13 (1.55 to 2.93) | 25.27% (15.43% to 37.29%) | 22.66 (15.88 to 30.63) | 2.22 (1.57 to 2.96) | 34.90% (20.90% to 50.73%) |
| **Idaho** | 30.72 (22.08 to 40.63) | 2.49 (1.80 to 3.25) | 13.99% (0.13% to 32.18%) | 16.67 (11.87 to 22.17) | 1.31 (0.92 to 1.73) | -10.51% (-19.27% to 0.04%) |
| **Illinois** | 163.41 (117.28 to 212.27) | 1.97 (1.42 to 2.57) | 1.23% (-10.06% to 14.47%) | 151.00 (109.83 to 198.74) | 1.69 (1.23 to 2.22) | 12.17% (-0.47% to 26.45%) |
| **Indiana** | 93.79 (68.39 to 123.75) | 2.11 (1.53 to 2.78) | 10.51% (-1.83% to 25.10%) | 71.16 (52.21 to 93.95) | 1.50 (1.10 to 1.98) | 11.83% (0.76% to 25.89%) |
| **Iowa** | 39.26 (28.39 to 53.39) | 1.80 (1.30 to 2.41) | -0.95% (-11.25% to 10.03%) | 34.51 (24.92 to 46.23) | 1.49 (1.06 to 2.00) | 7.23% (-3.43% to 18.02%) |
| **Kansas** | 46.65 (34.06 to 61.92) | 2.44 (1.76 to 3.19) | 18.78% (7.39% to 34.03%) | 31.43 (22.50 to 41.67) | 1.54 (1.10 to 2.06) | 13.35% (2.41% to 26.68%) |
| **Kentucky** | 68.85 (49.67 to 91.27) | 2.28 (1.65 to 3.01) | 27.02% (12.01% to 44.55%) | 41.27 (29.86 to 54.29) | 1.29 (0.93 to 1.68) | 9.54% (-1.91% to 23.81%) |
| **Louisiana** | 67.05 (48.79 to 88.08) | 2.27 (1.64 to 3.00) | 30.53% (15.60% to 48.80%) | 58.33 (42.86 to 77.00) | 1.86 (1.37 to 2.45) | 40.17% (24.44% to 60.93%) |
| **Maine** | 23.21 (16.59 to 31.04) | 2.22 (1.58 to 2.96) | 7.24% (-4.71% to 20.21%) | 19.09 (13.72 to 25.48) | 1.70 (1.23 to 2.27) | 9.02% (-2.50% to 21.21%) |
| **Maryland** | 79.83 (58.06 to 105.94) | 2.01 (1.46 to 2.69) | 7.52% (-3.73% to 21.53%) | 73.07 (52.40 to 97.00) | 1.67 (1.21 to 2.21) | 13.54% (2.45% to 26.69%) |
| **Massachusetts** | 119.24 (86.43 to 154.99) | 2.50 (1.81 to 3.27) | 8.25% (-1.22% to 20.24%) | 103.96 (74.67 to 137.30) | 1.96 (1.42 to 2.56) | 14.27% (4.09% to 26.94%) |
| **Michigan** | 168.66 (123.57 to 222.84) | 2.35 (1.72 to 3.08) | 15.51% (5.07% to 27.73%) | 122.09 (89.07 to 160.49) | 1.66 (1.20 to 2.17) | 20.18% (8.81% to 34.87%) |
| **Minnesota** | 117.54 (85.21 to 153.54) | 3.01 (2.18 to 3.93) | 19.64% (6.08% to 37.57%) | 78.49 (56.66 to 105.28) | 1.88 (1.36 to 2.48) | 8.84% (-2.28% to 21.44%) |
| **Mississippi** | 35.11 (25.45 to 46.53) | 1.89 (1.36 to 2.50) | 22.64% (10.17% to 37.59%) | 20.68 (14.87 to 28.25) | 0.99 (0.70 to 1.34) | 7.05% (-4.63% to 18.09%) |
| **Missouri** | 82.25 (59.92 to 107.47) | 1.99 (1.45 to 2.62) | 10.29% (-2.61% to 25.92%) | 70.07 (51.16 to 92.52) | 1.58 (1.14 to 2.08) | 16.51% (3.91% to 31.32%) |
| **Montana** | 17.07 (12.44 to 22.63) | 2.17 (1.58 to 2.87) | 12.18% (-0.27% to 26.57%) | 13.51 (9.68 to 18.12) | 1.69 (1.22 to 2.26) | 17.13% (4.60% to 31.09%) |
| **Nebraska** | 28.79 (20.45 to 38.45) | 2.20 (1.56 to 2.95) | 12.54% (2.36% to 24.38%) | 22.93 (16.42 to 30.66) | 1.68 (1.20 to 2.26) | 21.40% (9.06% to 33.70%) |
| **Nevada** | 32.75 (23.51 to 43.67) | 1.63 (1.17 to 2.18) | 1.79% (-8.47% to 13.73%) | 26.56 (18.85 to 35.27) | 1.30 (0.93 to 1.75) | 8.92% (-1.28% to 20.95%) |
| **New Hampshire** | 26.40 (18.66 to 34.79) | 2.56 (1.83 to 3.37) | 11.85% (0.68% to 24.46%) | 19.99 (14.35 to 26.38) | 1.86 (1.35 to 2.48) | 15.84% (4.96% to 29.35%) |
| **New Jersey** | 128.33 (93.22 to 168.03) | 2.06 (1.51 to 2.70) | 3.65% (-7.86% to 15.30%) | 129.52 (92.91 to 169.40) | 1.93 (1.40 to 2.52) | 20.28% (7.68% to 35.98%) |
| **New Mexico** | 26.40 (18.83 to 34.90) | 1.82 (1.31 to 2.40) | 9.56% (-1.58% to 23.07%) | 18.68 (13.47 to 24.71) | 1.22 (0.88 to 1.61) | 7.34% (-2.86% to 18.42%) |
| **New York** | 326.87 (237.26 to 430.20) | 2.46 (1.81 to 3.25) | 20.93% (8.51% to 35.41%) | 265.74 (193.32 to 350.70) | 1.79 (1.31 to 2.37) | 19.92% (6.62% to 35.79%) |
| **North Carolina** | 139.57 (100.29 to 185.42) | 2.03 (1.46 to 2.68) | 8.75% (-3.14% to 23.21%) | 127.05 (92.65 to 168.62) | 1.65 (1.19 to 2.18) | 19.21% (5.97% to 35.60%) |
| **North Dakota** | 13.04 (9.21 to 17.76) | 2.43 (1.73 to 3.26) | 31.77% (19.62% to 44.37%) | 12.59 (8.93 to 17.09) | 2.28 (1.64 to 3.08) | 32.14% (21.22% to 44.89%) |
| **Ohio** | 170.09 (124.86 to 223.56) | 2.11 (1.55 to 2.78) | 8.26% (-2.09% to 20.78%) | 145.36 (105.37 to 191.26) | 1.69 (1.23 to 2.22) | 17.54% (4.57% to 33.12%) |
| **Oklahoma** | 45.55 (32.74 to 60.44) | 1.81 (1.31 to 2.40) | 9.48% (-2.72% to 23.51%) | 41.99 (29.89 to 55.80) | 1.55 (1.11 to 2.05) | 22.37% (8.83% to 36.42%) |
| **Oregon** | 71.82 (52.39 to 95.74) | 2.33 (1.67 to 3.10) | 4.14% (-7.43% to 18.49%) | 56.29 (39.87 to 75.28) | 1.70 (1.23 to 2.23) | 3.76% (-7.37% to 15.50%) |
| **Pennsylvania** | 235.28 (173.66 to 309.40) | 2.61 (1.92 to 3.46) | 25.23% (10.19% to 42.88%) | 159.72 (115.72 to 210.38) | 1.61 (1.16 to 2.12) | 15.16% (1.50% to 30.19%) |
| **Rhode Island** | 15.65 (10.97 to 20.80) | 2.10 (1.48 to 2.87) | 13.91% (3.53% to 25.40%) | 13.46 (9.52 to 18.27) | 1.66 (1.17 to 2.24) | 20.46% (9.54% to 31.32%) |
| **South Carolina** | 71.10 (51.11 to 93.14) | 2.02 (1.45 to 2.62) | 12.04% (-0.10% to 24.90%) | 57.75 (41.72 to 76.42) | 1.48 (1.07 to 1.95) | 13.93% (2.46% to 30.11%) |
| **South Dakota** | 15.72 (11.19 to 21.15) | 2.58 (1.87 to 3.49) | 29.55% (19.13% to 42.77%) | 10.88 (7.58 to 14.85) | 1.77 (1.25 to 2.38) | 37.41% (24.75% to 51.70%) |
| **Tennessee** | 84.23 (61.15 to 110.55) | 1.88 (1.37 to 2.48) | 14.96% (3.05% to 27.74%) | 80.68 (58.35 to 106.55) | 1.62 (1.18 to 2.13) | 24.92% (10.75% to 40.71%) |
| **Texas** | 401.40 (292.82 to 525.38) | 2.27 (1.65 to 2.97) | 17.95% (5.46% to 31.38%) | 287.83 (207.31 to 376.00) | 1.50 (1.08 to 1.97) | 9.28% (-2.00% to 23.02%) |
| **Utah** | 45.68 (32.74 to 61.00) | 2.48 (1.78 to 3.29) | 10.51% (-0.92% to 24.10%) | 32.09 (23.09 to 42.82) | 1.66 (1.20 to 2.21) | 7.30% (-3.99% to 20.01%) |
| **Vermont** | 18.66 (13.42 to 24.65) | 3.72 (2.68 to 4.88) | 33.60% (19.86% to 48.31%) | 12.70 (9.06 to 17.24) | 2.51 (1.79 to 3.40) | 28.88% (17.95% to 40.89%) |
| **Virginia** | 107.77 (78.04 to 142.46) | 1.91 (1.38 to 2.53) | -2.82% (-12.42% to 8.87%) | 82.23 (58.95 to 110.39) | 1.36 (0.98 to 1.82) | -2.70% (-14.12% to 10.95%) |
| **Washington** | 118.80 (85.99 to 158.68) | 2.30 (1.67 to 3.05) | -6.21% (-16.39% to 7.05%) | 90.87 (65.88 to 119.25) | 1.65 (1.20 to 2.17) | -2.23% (-12.32% to 10.71%) |
| **West Virginia** | 28.52 (20.30 to 37.51) | 2.22 (1.59 to 2.96) | 29.63% (17.38% to 44.51%) | 21.83 (15.77 to 29.12) | 1.59 (1.16 to 2.11) | 32.76% (19.43% to 47.88%) |
| **Wisconsin** | 89.95 (65.82 to 120.18) | 2.19 (1.60 to 2.89) | 2.15% (-9.17% to 15.59%) | 74.70 (54.32 to 98.36) | 1.73 (1.25 to 2.28) | 11.20% (-1.33% to 25.62%) |
| **Wyoming** | 9.53 (6.85 to 12.54) | 2.33 (1.66 to 3.12) | 18.53% (8.42% to 32.04%) | 6.91 (4.91 to 9.42) | 1.71 (1.22 to 2.31) | 22.84% (11.71% to 33.57%) |

The numbers in parentheses represent the 95% uncertainty interval.

## Table S4f: Numbers and age-standardized rates of years of life lost (YLLs) in 2021, and percentage changes from 1990 to 2021, by sex

|  | **Male** | | | **Female** | | |
| --- | --- | --- | --- | --- | --- | --- |
|  | **Absolute number, 2021** | **Age-standardized rate, per 100,000 people, 2021** | **Percentage Change, 1990-2021** | **Absolute number, thousands, 2021** | **Age-standardized rate, per 100,000 people, 2021** | **Percentage Change, 1990-2021** |
| **United States of America** | 115272.61 (109575.75 to 120038.51) | 47.82 (45.55 to 49.73) | 6.19% (1.17% to 10.62%) | 86689.78 (80157.39 to 91098.92) | 31.87 (29.91 to 33.22) | -0.67% (-4.43% to 3.37%) |
| **Alabama** | 2001.68 (1567.57 to 2473.29) | 55.83 (44.39 to 68.13) | 32.34% (4.80% to 63.46%) | 1549.11 (1258.71 to 1862.91) | 37.51 (30.89 to 44.60) | 23.20% (1.30% to 48.88%) |
| **Alaska** | 236.04 (192.59 to 287.93) | 46.91 (38.49 to 56.35) | 3.23% (-16.05% to 25.46%) | 143.24 (123.87 to 164.52) | 30.54 (26.44 to 35.13) | -4.09% (-16.56% to 11.43%) |
| **Arizona** | 2726.61 (2202.03 to 3325.17) | 52.49 (42.80 to 63.39) | 15.30% (-6.63% to 41.13%) | 1869.20 (1505.20 to 2250.76) | 32.81 (26.93 to 39.11) | 4.96% (-14.53% to 27.81%) |
| **Arkansas** | 1230.17 (976.22 to 1531.62) | 57.48 (45.97 to 70.52) | 21.51% (-3.64% to 51.91%) | 871.34 (698.57 to 1052.07) | 36.81 (29.86 to 44.33) | 21.37% (-2.12% to 46.41%) |
| **California** | 11030.86 (8971.71 to 13445.69) | 39.90 (32.66 to 48.40) | -10.22% (-27.08% to 9.05%) | 7872.94 (6406.67 to 9422.26) | 25.68 (21.35 to 30.47) | -19.55% (-32.89% to -4.26%) |
| **Colorado** | 2173.04 (1670.02 to 2732.23) | 52.29 (40.56 to 64.98) | 1.47% (-21.94% to 26.80%) | 1716.04 (1369.52 to 2088.32) | 36.84 (29.83 to 44.40) | -2.50% (-21.30% to 16.71%) |
| **Connecticut** | 1379.01 (1072.10 to 1741.93) | 49.12 (38.53 to 61.73) | 3.59% (-20.80% to 30.66%) | 969.50 (759.23 to 1210.66) | 30.24 (23.90 to 37.11) | -7.91% (-25.67% to 14.28%) |
| **Delaware** | 377.86 (312.20 to 456.68) | 50.81 (42.07 to 60.99) | 13.86% (-7.22% to 37.24%) | 284.28 (246.62 to 332.14) | 32.67 (28.68 to 37.68) | 2.89% (-11.92% to 19.56%) |
| **District of Columbia** | 138.27 (110.56 to 170.03) | 34.07 (27.32 to 41.65) | -48.66% (-59.14% to -35.97%) | 109.49 (92.11 to 130.43) | 24.40 (20.61 to 28.78) | -50.96% (-59.98% to -37.68%) |
| **Florida** | 7701.71 (6134.50 to 9451.56) | 45.02 (36.19 to 54.71) | 1.45% (-19.79% to 23.74%) | 5588.29 (4542.04 to 6738.28) | 28.59 (23.77 to 34.26) | -2.87% (-19.15% to 15.94%) |
| **Georgia** | 3218.04 (2517.00 to 3952.88) | 44.47 (34.93 to 54.17) | 7.63% (-14.87% to 31.86%) | 2643.37 (2152.67 to 3181.51) | 31.49 (26.00 to 37.65) | 3.44% (-14.92% to 23.62%) |
| **Hawaii** | 438.99 (341.07 to 551.35) | 39.58 (30.98 to 48.90) | 8.03% (-16.24% to 35.62%) | 265.58 (220.44 to 314.96) | 22.32 (18.91 to 26.17) | -6.38% (-19.87% to 8.98%) |
| **Idaho** | 752.75 (579.78 to 950.21) | 56.44 (44.01 to 71.26) | 13.45% (-11.37% to 42.96%) | 564.30 (481.25 to 667.13) | 39.11 (33.69 to 45.80) | 3.01% (-12.09% to 21.64%) |
| **Illinois** | 4195.67 (3343.11 to 5167.25) | 45.77 (36.87 to 55.99) | 1.58% (-18.89% to 23.45%) | 2999.98 (2340.53 to 3694.18) | 29.06 (23.27 to 35.34) | -8.63% (-26.33% to 12.10%) |
| **Indiana** | 2714.33 (2166.39 to 3345.34) | 55.98 (45.54 to 68.33) | 19.33% (-3.95% to 46.67%) | 2082.03 (1714.19 to 2525.99) | 38.12 (31.42 to 45.67) | 21.06% (0.87% to 45.57%) |
| **Iowa** | 1270.16 (981.54 to 1568.74) | 52.97 (41.25 to 64.77) | 19.06% (-7.05% to 46.05%) | 958.70 (758.18 to 1174.68) | 36.24 (29.22 to 43.55) | 23.51% (-2.21% to 51.97%) |
| **Kansas** | 1170.72 (916.26 to 1451.47) | 56.26 (44.67 to 69.09) | 14.03% (-10.20% to 41.94%) | 932.07 (748.49 to 1140.79) | 40.14 (32.44 to 49.11) | 23.69% (-0.36% to 53.21%) |
| **Kentucky** | 1730.30 (1371.13 to 2149.13) | 52.64 (42.04 to 64.86) | 22.20% (-4.54% to 52.02%) | 1340.80 (1083.75 to 1654.22) | 36.02 (29.46 to 43.99) | 22.51% (-0.92% to 51.79%) |
| **Louisiana** | 1677.79 (1332.44 to 2076.82) | 52.44 (42.27 to 64.30) | 17.49% (-5.92% to 44.91%) | 1283.91 (1026.38 to 1559.74) | 35.63 (28.83 to 43.15) | 10.38% (-10.82% to 32.79%) |
| **Maine** | 702.45 (543.47 to 882.26) | 60.10 (47.14 to 74.75) | 23.31% (-4.50% to 53.88%) | 561.61 (463.68 to 677.75) | 40.91 (34.01 to 48.67) | 13.52% (-5.59% to 34.59%) |
| **Maryland** | 2044.96 (1585.62 to 2521.55) | 46.80 (36.99 to 57.21) | 6.35% (-17.23% to 32.19%) | 1665.40 (1332.29 to 2047.55) | 32.50 (26.31 to 39.57) | -0.97% (-19.16% to 20.85%) |
| **Massachusetts** | 2490.10 (1904.69 to 3173.12) | 46.34 (35.57 to 58.73) | -1.76% (-22.89% to 25.23%) | 1950.88 (1531.08 to 2417.35) | 30.52 (24.30 to 37.45) | -12.33% (-30.65% to 9.16%) |
| **Michigan** | 4120.49 (3275.84 to 5041.90) | 53.00 (42.60 to 64.27) | 16.99% (-6.98% to 41.24%) | 2933.73 (2398.31 to 3511.54) | 33.82 (28.17 to 40.31) | 4.21% (-13.92% to 25.02%) |
| **Minnesota** | 2663.17 (2041.06 to 3340.64) | 61.10 (47.33 to 75.76) | 14.27% (-10.66% to 43.66%) | 1925.73 (1550.64 to 2376.29) | 40.75 (33.32 to 49.68) | 3.93% (-15.49% to 27.09%) |
| **Mississippi** | 1035.37 (812.39 to 1274.12) | 51.63 (41.31 to 62.87) | 25.99% (0.97% to 52.26%) | 764.38 (626.47 to 924.09) | 32.88 (27.23 to 39.57) | 15.42% (-4.69% to 39.56%) |
| **Missouri** | 2569.35 (2018.73 to 3205.06) | 56.97 (45.38 to 69.82) | 28.31% (1.40% to 59.84%) | 1894.98 (1550.92 to 2282.72) | 36.79 (30.59 to 44.07) | 10.15% (-9.29% to 33.69%) |
| **Montana** | 525.89 (409.40 to 663.45) | 59.49 (47.20 to 74.05) | 21.85% (-2.16% to 54.25%) | 360.13 (307.47 to 418.84) | 38.76 (33.62 to 44.69) | 5.79% (-8.89% to 24.44%) |
| **Nebraska** | 786.72 (619.07 to 980.66) | 55.58 (44.08 to 68.74) | 13.24% (-10.89% to 41.03%) | 547.54 (452.73 to 646.01) | 36.05 (30.04 to 42.29) | 13.22% (-4.75% to 34.32%) |
| **Nevada** | 881.11 (691.69 to 1097.19) | 39.15 (31.09 to 48.35) | 2.71% (-17.92% to 28.15%) | 619.05 (532.35 to 717.17) | 25.79 (22.39 to 29.80) | -4.60% (-17.68% to 11.41%) |
| **New Hampshire** | 583.80 (447.50 to 745.19) | 49.95 (38.75 to 62.46) | 0.89% (-21.68% to 28.23%) | 401.85 (329.68 to 489.60) | 30.78 (25.70 to 36.97) | -8.50% (-24.01% to 11.53%) |
| **New Jersey** | 2508.01 (1947.18 to 3176.66) | 36.47 (28.73 to 45.72) | -14.09% (-32.83% to 8.01%) | 2136.52 (1663.88 to 2625.97) | 26.40 (20.93 to 32.39) | -14.39% (-32.15% to 5.66%) |
| **New Mexico** | 761.70 (598.75 to 937.59) | 50.01 (39.86 to 61.60) | 31.12% (2.01% to 63.93%) | 555.38 (443.64 to 678.74) | 32.17 (26.13 to 38.44) | 17.93% (-4.01% to 42.67%) |
| **New York** | 5551.93 (4388.97 to 6785.28) | 37.56 (29.90 to 45.44) | -8.09% (-26.99% to 12.48%) | 4333.06 (3523.68 to 5244.73) | 25.01 (20.44 to 30.02) | -13.54% (-29.86% to 5.59%) |
| **North Carolina** | 3896.68 (3079.23 to 4793.28) | 52.04 (41.55 to 63.67) | 6.26% (-16.17% to 30.97%) | 3042.18 (2431.23 to 3672.12) | 34.75 (28.18 to 41.45) | 13.41% (-7.57% to 36.74%) |
| **North Dakota** | 287.65 (233.22 to 343.02) | 51.20 (42.10 to 60.70) | 6.61% (-12.92% to 28.25%) | 195.99 (165.84 to 229.40) | 34.51 (29.34 to 39.84) | 2.88% (-13.05% to 20.77%) |
| **Ohio** | 4606.98 (3723.49 to 5648.53) | 52.29 (42.75 to 63.74) | 10.45% (-9.73% to 34.50%) | 3832.51 (3099.71 to 4634.07) | 38.07 (31.18 to 45.45) | 16.75% (-4.29% to 40.77%) |
| **Oklahoma** | 1559.37 (1247.24 to 1929.83) | 56.75 (45.87 to 69.50) | 34.98% (9.54% to 65.78%) | 1144.35 (950.17 to 1369.77) | 37.28 (31.17 to 44.40) | 22.59% (1.24% to 46.96%) |
| **Oregon** | 2046.59 (1606.44 to 2542.63) | 60.13 (47.36 to 74.17) | 17.39% (-6.75% to 47.26%) | 1479.44 (1178.35 to 1847.78) | 38.88 (31.34 to 47.94) | -2.84% (-21.69% to 20.82%) |
| **Pennsylvania** | 5286.39 (4170.68 to 6624.47) | 52.10 (41.46 to 64.45) | 15.95% (-8.89% to 44.38%) | 3776.39 (3025.15 to 4606.73) | 32.83 (26.54 to 39.97) | -1.68% (-20.09% to 18.94%) |
| **Rhode Island** | 341.52 (264.82 to 432.50) | 40.59 (31.92 to 50.96) | -11.60% (-30.14% to 11.37%) | 285.20 (231.89 to 346.05) | 29.03 (24.07 to 34.78) | -10.59% (-25.47% to 7.81%) |
| **South Carolina** | 1939.33 (1532.90 to 2388.64) | 51.45 (41.10 to 62.62) | 15.18% (-8.52% to 40.57%) | 1460.06 (1183.05 to 1761.67) | 33.17 (27.26 to 39.95) | 0.05% (-19.10% to 21.56%) |
| **South Dakota** | 357.18 (296.22 to 429.54) | 55.46 (46.63 to 65.83) | 16.48% (-3.71% to 41.03%) | 255.75 (218.45 to 295.08) | 37.73 (32.93 to 43.20) | 9.41% (-6.45% to 27.82%) |
| **Tennessee** | 2734.57 (2154.68 to 3406.46) | 54.86 (44.09 to 67.82) | 24.19% (-1.23% to 53.31%) | 2131.31 (1744.92 to 2572.47) | 37.22 (30.74 to 44.54) | 17.15% (-4.52% to 43.70%) |
| **Texas** | 7780.51 (6211.82 to 9411.60) | 41.07 (33.08 to 49.38) | -1.04% (-20.67% to 18.68%) | 5834.00 (4803.25 to 7035.18) | 27.86 (23.19 to 33.10) | -10.32% (-26.06% to 8.35%) |
| **Utah** | 987.06 (776.38 to 1236.21) | 52.40 (41.44 to 65.10) | 5.28% (-16.78% to 30.33%) | 734.43 (626.31 to 858.09) | 36.18 (31.10 to 42.03) | 2.28% (-12.13% to 20.05%) |
| **Vermont** | 301.49 (245.43 to 368.59) | 55.43 (45.77 to 66.82) | 2.74% (-16.45% to 25.58%) | 235.28 (201.21 to 275.60) | 38.99 (33.67 to 45.04) | -8.43% (-21.63% to 5.72%) |
| **Virginia** | 3101.44 (2462.39 to 3819.17) | 49.58 (39.79 to 60.57) | 2.45% (-18.42% to 24.18%) | 2369.90 (1913.76 to 2879.59) | 33.82 (27.58 to 40.50) | 0.62% (-17.84% to 23.13%) |
| **Washington** | 3147.51 (2498.12 to 3813.09) | 53.92 (43.21 to 65.14) | 1.39% (-20.11% to 23.88%) | 2461.29 (1993.38 to 3027.19) | 38.22 (31.20 to 46.59) | -4.84% (-21.57% to 15.29%) |
| **West Virginia** | 874.11 (674.25 to 1073.12) | 61.21 (48.09 to 74.48) | 39.39% (9.61% to 69.90%) | 586.53 (470.11 to 723.16) | 37.38 (30.20 to 45.60) | 42.85% (14.18% to 74.93%) |
| **Wisconsin** | 2397.46 (1887.13 to 3019.48) | 52.70 (42.01 to 65.36) | 15.51% (-11.02% to 42.34%) | 1989.58 (1611.23 to 2426.65) | 40.00 (32.82 to 48.39) | 8.50% (-11.32% to 32.62%) |
| **Wyoming** | 237.71 (199.23 to 292.27) | 54.00 (45.48 to 65.41) | 16.65% (-3.66% to 43.47%) | 181.18 (154.33 to 210.25) | 40.51 (34.73 to 46.56) | 11.98% (-2.72% to 29.26%) |

The numbers in parentheses represent the 95% uncertainty interval.

## Table S5a: Prevalence, DALYs, and mortality rates in 2021, by age groups and sex

|  | **Prevalence (95% UI)** | | **DALYs (95% UI)** | | **Deaths (95% UI)** | |
| --- | --- | --- | --- | --- | --- | --- |
|  | **Male** | **Female** | **Male** | **Female** | **Male** | **Female** |
| **<5 years** | 3.08 (2.67 to 3.56) | 2.87 (2.50 to 3.32) | 27.43 (24.01 to 31.02) | 27.10 (24.35 to 29.85) | 0.30 (0.26 to 0.34) | 0.30 (0.27 to 0.33) |
| **5 to 9** | 4.95 (4.25 to 5.74) | 4.34 (3.75 to 5.04) | 3.61 (3.25 to 4.03) | 3.73 (3.40 to 4.12) | 0.03 (0.03 to 0.03) | 0.03 (0.03 to 0.04) |
| **10 to 14** | 5.03 (4.28 to 5.85) | 4.36 (3.74 to 5.09) | 2.94 (2.63 to 3.36) | 3.18 (2.86 to 3.55) | 0.02 (0.02 to 0.03) | 0.03 (0.03 to 0.03) |
| **15 to 19** | 4.98 (4.23 to 5.80) | 4.31 (3.69 to 5.03) | 3.40 (3.07 to 3.80) | 2.48 (2.19 to 2.83) | 0.03 (0.03 to 0.03) | 0.02 (0.02 to 0.02) |
| **20 to 24** | 4.87 (4.14 to 5.65) | 4.24 (3.62 to 4.95) | 4.25 (3.81 to 4.71) | 3.12 (2.79 to 3.58) | 0.05 (0.04 to 0.05) | 0.03 (0.03 to 0.04) |
| **25 to 29** | 4.75 (4.06 to 5.51) | 4.13 (3.56 to 4.76) | 6.73 (6.22 to 7.25) | 3.32 (3.00 to 3.68) | 0.09 (0.08 to 0.10) | 0.04 (0.04 to 0.04) |
| **30 to 34** | 4.72 (4.02 to 5.51) | 4.03 (3.48 to 4.66) | 11.60 (10.82 to 12.35) | 5.41 (5.02 to 5.81) | 0.18 (0.17 to 0.20) | 0.08 (0.07 to 0.08) |
| **35 to 39** | 5.34 (4.33 to 6.65) | 4.21 (3.57 to 5.05) | 19.46 (18.09 to 20.71) | 8.88 (8.32 to 9.52) | 0.35 (0.32 to 0.37) | 0.15 (0.14 to 0.16) |
| **40 to 44** | 7.61 (6.08 to 9.79) | 5.03 (4.08 to 6.28) | 34.34 (32.11 to 36.57) | 16.87 (16.00 to 17.90) | 0.68 (0.64 to 0.73) | 0.33 (0.31 to 0.35) |
| **45 to 49** | 10.90 (8.70 to 13.55) | 6.43 (4.95 to 8.29) | 64.05 (59.94 to 67.97) | 29.55 (28.06 to 31.08) | 1.44 (1.35 to 1.53) | 0.66 (0.62 to 0.69) |
| **50 to 54** | 14.15 (11.34 to 17.18) | 8.93 (7.08 to 10.99) | 97.72 (92.37 to 103.16) | 59.14 (56.58 to 61.88) | 2.48 (2.34 to 2.62) | 1.50 (1.43 to 1.57) |
| **55 to 59** | 18.69 (15.64 to 22.33) | 12.31 (10.07 to 14.94) | 144.92 (135.80 to 152.89) | 90.19 (86.68 to 93.80) | 4.21 (3.94 to 4.44) | 2.62 (2.52 to 2.72) |
| **60 to 64** | 25.99 (22.04 to 30.85) | 16.45 (13.64 to 19.80) | 188.61 (176.84 to 199.83) | 126.27 (119.77 to 132.05) | 6.34 (5.93 to 6.72) | 4.25 (4.04 to 4.45) |
| **65 to 69** | 35.73 (29.15 to 42.85) | 24.11 (19.67 to 29.16) | 218.88 (205.31 to 231.06) | 160.70 (149.19 to 169.08) | 8.68 (8.15 to 9.16) | 6.40 (5.91 to 6.74) |
| **70 to 74** | 45.16 (37.57 to 54.40) | 31.61 (26.14 to 38.39) | 245.58 (229.88 to 258.43) | 186.95 (167.78 to 203.36) | 11.83 (10.99 to 12.44) | 9.05 (8.06 to 9.85) |
| **75 to 79** | 49.52 (40.82 to 59.58) | 34.14 (28.04 to 41.29) | 257.94 (237.14 to 274.36) | 188.80 (163.35 to 210.34) | 15.52 (14.29 to 16.55) | 11.41 (9.85 to 12.72) |
| **80 to 84** | 44.64 (36.27 to 54.10) | 29.57 (23.99 to 35.74) | 204.42 (178.63 to 222.91) | 137.64 (108.59 to 157.22) | 15.63 (13.68 to 17.06) | 10.56 (8.14 to 12.13) |
| **85 to 89** | 38.03 (29.67 to 47.99) | 26.57 (20.87 to 32.79) | 141.05 (118.54 to 155.61) | 78.27 (59.60 to 90.49) | 13.45 (11.24 to 14.93) | 7.38 (5.47 to 8.64) |
| **90 to 94** | 30.82 (23.05 to 40.18) | 20.57 (15.21 to 26.65) | 87.80 (70.70 to 98.70) | 47.91 (35.99 to 56.36) | 9.44 (7.42 to 10.66) | 5.06 (3.65 to 5.94) |
| **95 plus** | 27.99 (16.89 to 41.56) | 19.37 (12.02 to 28.20) | 74.07 (54.87 to 85.64) | 33.50 (24.63 to 39.60) | 8.35 (6.05 to 9.66) | 3.62 (2.50 to 4.30) |

Abbreviations: DALYs, disability-adjusted life years; UI, uncertainty interval

## Table S5b: Incidence, YLD, and YLL rates in 2021, by age groups and sex

|  | **Incidence (95% UI)** | | **YLDs (95% UI)** | | **YLLs (95% UI)** | |
| --- | --- | --- | --- | --- | --- | --- |
|  | **Male** | **Female** | **Male** | **Female** | **Male** | **Female** |
| **<5 years** | 1.58 (1.39 to 1.81) | 1.41 (1.24 to 1.62) | 0.65 (0.46 to 0.89) | 0.61 (0.43 to 0.82) | 26.77 (23.36 to 30.36) | 26.49 (23.84 to 29.26) |
| **5 to 9** | 0.10 (0.05 to 0.15) | 0.08 (0.04 to 0.13) | 1.05 (0.75 to 1.42) | 0.92 (0.66 to 1.23) | 2.56 (2.31 to 2.78) | 2.81 (2.64 to 3.00) |
| **10 to 14** | 0.03 (0.01 to 0.06) | 0.02 (0.01 to 0.05) | 1.07 (0.77 to 1.45) | 0.93 (0.66 to 1.24) | 1.87 (1.74 to 2.01) | 2.26 (2.11 to 2.41) |
| **15 to 19** | 0.02 (0.01 to 0.05) | 0.01 (0.00 to 0.04) | 1.06 (0.76 to 1.44) | 0.92 (0.66 to 1.24) | 2.34 (2.18 to 2.51) | 1.57 (1.47 to 1.71) |
| **20 to 24** | 0.03 (0.01 to 0.08) | 0.02 (0.00 to 0.04) | 1.03 (0.74 to 1.39) | 0.90 (0.65 to 1.21) | 3.21 (2.92 to 3.52) | 2.22 (2.04 to 2.59) |
| **25 to 29** | 0.08 (0.04 to 0.16) | 0.04 (0.01 to 0.08) | 1.01 (0.72 to 1.39) | 0.88 (0.63 to 1.19) | 5.72 (5.32 to 6.10) | 2.44 (2.27 to 2.61) |
| **30 to 34** | 0.25 (0.13 to 0.42) | 0.10 (0.05 to 0.19) | 1.00 (0.71 to 1.35) | 0.86 (0.61 to 1.16) | 10.60 (9.89 to 11.29) | 4.55 (4.31 to 4.80) |
| **35 to 39** | 0.73 (0.47 to 1.12) | 0.28 (0.16 to 0.47) | 1.14 (0.79 to 1.59) | 0.89 (0.62 to 1.24) | 18.32 (17.03 to 19.41) | 7.98 (7.51 to 8.55) |
| **40 to 44** | 1.49 (1.06 to 1.98) | 0.62 (0.37 to 0.93) | 1.62 (1.09 to 2.35) | 1.07 (0.71 to 1.55) | 32.72 (30.56 to 34.76) | 15.80 (15.00 to 16.74) |
| **45 to 49** | 2.25 (1.65 to 2.91) | 1.19 (0.77 to 1.63) | 2.32 (1.56 to 3.26) | 1.37 (0.91 to 1.96) | 61.73 (57.83 to 65.67) | 28.18 (26.72 to 29.70) |
| **50 to 54** | 3.39 (2.66 to 4.17) | 2.18 (1.63 to 2.74) | 3.01 (2.11 to 4.09) | 1.90 (1.28 to 2.69) | 94.71 (89.56 to 100.05) | 57.24 (54.68 to 59.91) |
| **55 to 59** | 5.44 (4.46 to 6.50) | 3.55 (2.86 to 4.33) | 3.97 (2.73 to 5.42) | 2.62 (1.78 to 3.62) | 140.95 (131.95 to 148.65) | 87.57 (84.14 to 91.02) |
| **60 to 64** | 8.45 (7.03 to 9.88) | 6.06 (4.98 to 7.06) | 5.52 (3.83 to 7.46) | 3.50 (2.37 to 4.74) | 183.08 (171.27 to 194.16) | 122.78 (116.49 to 128.30) |
| **65 to 69** | 11.88 (10.06 to 13.69) | 9.11 (7.77 to 10.42) | 7.58 (5.27 to 10.52) | 5.12 (3.55 to 7.13) | 211.29 (198.40 to 222.98) | 155.58 (143.50 to 163.83) |
| **70 to 74** | 15.33 (13.20 to 17.33) | 11.41 (9.85 to 12.93) | 9.56 (6.55 to 13.10) | 6.71 (4.56 to 9.10) | 236.02 (219.21 to 248.05) | 180.24 (160.57 to 196.05) |
| **75 to 79** | 17.35 (14.86 to 19.60) | 12.57 (10.81 to 14.11) | 10.44 (7.27 to 14.54) | 7.25 (5.10 to 10.10) | 247.50 (227.84 to 263.91) | 181.55 (156.72 to 202.39) |
| **80 to 84** | 17.16 (14.86 to 19.64) | 11.83 (10.26 to 13.45) | 9.46 (6.70 to 12.80) | 6.28 (4.40 to 8.58) | 194.96 (170.73 to 212.83) | 131.36 (101.31 to 150.87) |
| **85 to 89** | 14.16 (12.12 to 16.39) | 8.32 (6.99 to 9.77) | 8.07 (5.59 to 11.23) | 5.65 (3.84 to 7.70) | 132.98 (111.10 to 147.59) | 72.62 (53.84 to 85.08) |
| **90 to 94** | 10.07 (8.15 to 12.16) | 4.90 (3.68 to 6.09) | 6.54 (4.37 to 9.56) | 4.37 (2.96 to 6.24) | 81.26 (63.89 to 91.83) | 43.55 (31.39 to 51.13) |
| **95 plus** | 6.97 (4.45 to 10.03) | 3.30 (1.97 to 5.09) | 5.95 (3.42 to 9.49) | 4.12 (2.37 to 6.30) | 68.12 (49.33 to 78.78) | 29.38 (20.30 to 34.87) |

Abbreviations: UI, uncertainty interval; YLDs, years lived with disability; YLLs, years of life lost

## Table S6: Rates of prevalence, DALYs, mortality, incidence, YLD, and YLL in 2021, by age groups

|  | **Prevalence (95% UI)** | **DALYs**  **(95% UI)** | **Deaths**  **(95% UI)** | **Incidence (95% UI)** | **YLDs**  **(95% UI)** | **YLLs**  **(95% UI)** |
| --- | --- | --- | --- | --- | --- | --- |
| **<5 years** | 2.98 (2.59 to 3.44) | 27.27 (24.53 to 30.25) | 0.30 (0.27 to 0.33) | 1.50 (1.32 to 1.72) | 0.63 (0.45 to 0.85) | 26.64 (23.97 to 29.58) |
| **5 to 9** | 4.65 (4.01 to 5.39) | 3.67 (3.33 to 4.05) | 0.03 (0.03 to 0.03) | 0.09 (0.04 to 0.14) | 0.99 (0.71 to 1.33) | 2.68 (2.49 to 2.86) |
| **10 to 14** | 4.70 (4.00 to 5.48) | 3.06 (2.74 to 3.45) | 0.03 (0.03 to 0.03) | 0.02 (0.01 to 0.05) | 1.00 (0.71 to 1.34) | 2.06 (1.94 to 2.19) |
| **15 to 19** | 4.66 (3.96 to 5.41) | 2.95 (2.66 to 3.31) | 0.03 (0.03 to 0.03) | 0.02 (0.01 to 0.04) | 0.99 (0.71 to 1.34) | 1.96 (1.85 to 2.08) |
| **20 to 24** | 4.56 (3.89 to 5.29) | 3.69 (3.32 to 4.09) | 0.04 (0.04 to 0.04) | 0.03 (0.01 to 0.06) | 0.97 (0.69 to 1.30) | 2.72 (2.50 to 2.93) |
| **25 to 29** | 4.44 (3.82 to 5.14) | 5.04 (4.67 to 5.45) | 0.07 (0.06 to 0.07) | 0.06 (0.03 to 0.12) | 0.94 (0.67 to 1.29) | 4.10 (3.85 to 4.32) |
| **30 to 34** | 4.38 (3.75 to 5.09) | 8.51 (7.99 to 9.05) | 0.13 (0.12 to 0.14) | 0.18 (0.09 to 0.31) | 0.93 (0.66 to 1.26) | 7.58 (7.12 to 7.97) |
| **35 to 39** | 4.77 (3.97 to 5.77) | 14.14 (13.26 to 14.95) | 0.25 (0.23 to 0.26) | 0.50 (0.32 to 0.80) | 1.01 (0.70 to 1.43) | 13.12 (12.32 to 13.77) |
| **40 to 44** | 6.31 (5.11 to 8.00) | 25.51 (24.13 to 26.92) | 0.50 (0.48 to 0.53) | 1.05 (0.72 to 1.44) | 1.34 (0.91 to 1.94) | 24.17 (22.89 to 25.49) |
| **45 to 49** | 8.63 (6.83 to 10.80) | 46.56 (44.16 to 49.04) | 1.04 (0.99 to 1.10) | 1.72 (1.22 to 2.26) | 1.84 (1.24 to 2.61) | 44.73 (42.39 to 47.04) |
| **50 to 54** | 11.50 (9.24 to 14.02) | 78.14 (74.75 to 81.44) | 1.98 (1.90 to 2.06) | 2.77 (2.15 to 3.45) | 2.44 (1.70 to 3.39) | 75.70 (72.49 to 78.80) |
| **55 to 59** | 15.42 (12.87 to 18.38) | 116.90 (111.47 to 121.96) | 3.40 (3.25 to 3.54) | 4.48 (3.63 to 5.39) | 3.28 (2.24 to 4.48) | 113.62 (108.69 to 118.44) |
| **60 to 64** | 21.04 (17.74 to 25.08) | 156.26 (148.30 to 163.20) | 5.26 (4.99 to 5.50) | 7.21 (5.96 to 8.40) | 4.47 (3.07 to 6.06) | 151.79 (144.12 to 158.72) |
| **65 to 69** | 29.59 (24.16 to 35.65) | 188.13 (177.43 to 195.69) | 7.48 (7.03 to 7.79) | 10.42 (8.86 to 11.95) | 6.28 (4.36 to 8.72) | 181.85 (171.05 to 189.50) |
| **70 to 74** | 37.89 (31.54 to 45.66) | 214.10 (199.28 to 226.43) | 10.34 (9.61 to 10.96) | 13.23 (11.40 to 14.97) | 8.03 (5.48 to 10.97) | 206.06 (191.57 to 218.40) |
| **75 to 79** | 41.04 (33.84 to 49.52) | 219.84 (196.06 to 235.37) | 13.26 (11.79 to 14.21) | 14.72 (12.62 to 16.57) | 8.68 (6.07 to 12.09) | 211.16 (187.82 to 226.34) |
| **80 to 84** | 35.99 (29.39 to 43.53) | 166.11 (137.95 to 181.89) | 12.72 (10.45 to 13.94) | 14.10 (12.20 to 16.08) | 7.64 (5.39 to 10.40) | 158.47 (130.20 to 173.69) |
| **85 to 89** | 31.05 (24.28 to 38.72) | 102.78 (82.16 to 114.39) | 9.75 (7.72 to 10.89) | 10.60 (8.99 to 12.30) | 6.59 (4.52 to 9.06) | 96.19 (76.14 to 107.46) |
| **90 to 94** | 24.02 (18.11 to 31.04) | 61.36 (47.80 to 69.73) | 6.54 (4.90 to 7.46) | 6.64 (5.20 to 8.10) | 5.10 (3.50 to 7.39) | 56.26 (42.19 to 64.20) |
| **95 plus** | 21.66 (13.39 to 31.62) | 44.29 (32.73 to 51.35) | 4.88 (3.46 to 5.69) | 4.28 (2.64 to 6.35) | 4.60 (2.62 to 7.18) | 39.69 (28.14 to 46.33) |

Abbreviations: DALYs, disability-adjusted life years; UI, uncertainty interval; YLDs, years lived with disability; YLLs, years of life lost

## Table S7a: Numbers and age-standardized rates of prevalence, DALYs, and deaths in 2021, and percentage changes from 1990 to 2021, with SDI

|  | **Prevalence (95% UI)** | | | **DALYs (95% UI)** | | | **Deaths (95% UI)** | | |  |
| --- | --- | --- | --- | --- | --- | --- | --- | --- | --- | --- |
|  | **Absolute number, thousands, 2021** | **Age-standardized rate,**  **per 100,000 people, 2021** | **Percentage Change, 1990-2021** | **Absolute number, thousands, 2021** | **Age-standardized rate,**  **per 100,000 people, 2021** | **Percentage Change, 1990-2021** | **Absolute number, thousands, 2021** | **Age-standardized rate,**  **per 100,000 people, 2021** | **Percentage Change, 1990-2021** | **SDI** |
| **United States of America** | 40495.43 (37645.36 to 43576.27) | 8.82 (8.26 to 9.51) | 12.89% (3.10% to 23.66%) | 210559.45 (199250.06 to 218996.64) | 41.36 (39.47 to 42.94) | 4.14% (0.41% to 7.68%) | 8465.56 (7799.92 to 8898.99) | 1.49 (1.38 to 1.56) | 18.34% (13.86% to 22.70%) | 0.862 |
| **Alabama** | 527.04 (485.12 to 572.87) | 7.71 (7.05 to 8.46) | 20.79% (8.47% to 34.14%) | 3662.79 (3105.26 to 4242.72) | 47.85 (41.16 to 55.03) | 28.53% (10.43% to 48.41%) | 144.10 (122.23 to 166.91) | 1.67 (1.43 to 1.94) | 43.00% (22.69% to 67.23%) | 0.826 |
| **Alaska** | 91.22 (79.00 to 105.06) | 10.17 (8.92 to 11.56) | 31.47% (19.32% to 43.94%) | 398.65 (347.85 to 455.05) | 41.13 (36.26 to 46.87) | 1.71% (-10.69% to 16.27%) | 14.54 (12.62 to 16.74) | 1.39 (1.21 to 1.59) | 8.73% (-6.12% to 27.07%) | 0.858 |
| **Arizona** | 874.40 (810.14 to 945.52) | 8.84 (8.14 to 9.57) | 20.86% (7.73% to 36.11%) | 4781.60 (4116.17 to 5502.53) | 44.11 (38.43 to 50.53) | 11.39% (-3.74% to 28.51%) | 190.98 (161.56 to 220.82) | 1.54 (1.31 to 1.77) | 29.60% (10.33% to 50.52%) | 0.847 |
| **Arkansas** | 383.61 (348.09 to 423.32) | 9.37 (8.52 to 10.35) | 31.58% (17.89% to 46.48%) | 2183.00 (1871.13 to 2521.17) | 48.71 (41.99 to 55.88) | 22.75% (4.53% to 42.69%) | 85.05 (72.15 to 99.24) | 1.67 (1.43 to 1.94) | 35.62% (14.20% to 59.55%) | 0.818 |
| **California** | 4300.56 (4028.65 to 4639.75) | 8.13 (7.65 to 8.72) | 4.04% (-7.28% to 16.85%) | 19817.21 (17190.89 to 22569.75) | 34.26 (29.89 to 38.77) | -13.10% (-23.80% to  -2.24%) | 789.01 (678.90 to 908.49) | 1.24 (1.07 to 1.43) | 1.10% (-12.80% to 14.40%) | 0.870 |
| **Colorado** | 819.47 (758.22 to 887.19) | 10.33 (9.61 to 11.21) | 14.90% (3.11% to 28.27%) | 4062.15 (3413.58 to 4737.90) | 46.56 (39.62 to 53.94) | 0.91% (-13.87% to 16.86%) | 161.11 (132.31 to 189.65) | 1.69 (1.39 to 1.99) | 13.54% (-4.48% to 32.45%) | 0.877 |
| **Connecticut** | 561.43 (522.32 to 606.48) | 10.57 (9.79 to 11.47) | 15.25% (4.27% to 27.75%) | 2467.62 (2055.40 to 2890.17) | 41.42 (34.90 to 48.25) | -0.12% (-16.69% to 17.37%) | 101.73 (82.87 to 119.94) | 1.51 (1.25 to 1.77) | 8.93% (-9.98% to 29.35%) | 0.903 |
| **Delaware** | 154.61 (142.00 to 168.76) | 10.85 (9.97 to 11.80) | 31.08% (19.44% to 44.66%) | 694.91 (614.77 to 789.69) | 43.47 (38.81 to 48.93) | 10.22% (-3.25% to 24.73%) | 28.02 (24.54 to 32.39) | 1.51 (1.33 to 1.73) | 17.83% (3.11% to 34.62%) | 0.866 |
| **District of Columbia** | 64.75 (56.68 to 74.60) | 7.95 (6.97 to 9.13) | 28.28% (18.06% to 40.21%) | 261.52 (226.11 to 302.41) | 30.65 (26.74 to 35.25) | -47.80% (-56.19% to  -38.44%) | 9.35 (7.93 to 10.85) | 1.01 (0.86 to 1.17) | -24.44% (-36.84% to  -11.38%) | 0.906 |
| **Florida** | 2702.84 (2512.31 to 2909.89) | 8.11 (7.55 to 8.73) | 17.57% (4.77% to 32.71%) | 13864.25 (11738.85 to 16152.59) | 38.13 (32.82 to 43.86) | 0.67% (-13.04% to 16.31%) | 568.39 (473.42 to 667.00) | 1.34 (1.12 to 1.56) | 22.03% (3.55% to 42.21%) | 0.862 |
| **Georgia** | 1037.25 (956.83 to 1119.01) | 7.42 (6.91 to 7.99) | 8.11% (-1.02% to 20.03%) | 6081.83 (5246.13 to 7013.91) | 39.06 (33.94 to 44.57) | 6.10% (-8.13% to 22.05%) | 240.79 (204.06 to 281.32) | 1.42 (1.21 to 1.65) | 22.05% (4.11% to 41.55%) | 0.847 |
| **Hawaii** | 204.28 (180.20 to 233.29) | 10.25 (9.14 to 11.60) | 30.38% (20.91% to 41.65%) | 747.96 (630.79 to 874.09) | 32.96 (28.29 to 38.00) | 3.77% (-12.04% to 21.40%) | 29.62 (24.74 to 34.86) | 1.11 (0.93 to 1.30) | 16.70% (-2.78% to 38.36%) | 0.870 |
| **Idaho** | 223.45 (204.28 to 247.00) | 8.89 (8.17 to 9.71) | 4.52% (-5.92% to 17.16%) | 1364.44 (1148.28 to 1588.82) | 49.46 (41.75 to 56.97) | 8.93% (-6.49% to 26.22%) | 56.41 (47.28 to 65.41) | 1.84 (1.55 to 2.14) | 23.34% (4.31% to 43.34%) | 0.837 |
| **Illinois** | 1479.68 (1381.43 to 1592.21) | 8.57 (8.02 to 9.22) | 6.60% (-4.04% to 19.73%) | 7510.06 (6460.11 to 8712.85) | 38.82 (33.72 to 44.53) | -1.78% (-14.82% to 12.75%) | 302.81 (258.68 to 354.23) | 1.41 (1.21 to 1.64) | 16.00% (-0.22% to 35.64%) | 0.879 |
| **Indiana** | 776.28 (716.95 to 836.17) | 8.43 (7.80 to 9.09) | 11.69% (0.52% to 24.47%) | 4961.30 (4252.75 to 5683.89) | 48.43 (41.87 to 55.16) | 20.73% (4.68% to 38.83%) | 198.87 (170.50 to 228.42) | 1.75 (1.50 to 2.00) | 33.71% (14.69% to 55.07%) | 0.843 |
| **Iowa** | 347.38 (304.98 to 393.22) | 7.70 (6.81 to 8.67) | 3.27% (-5.69% to 12.91%) | 2302.63 (1949.06 to 2668.59) | 46.01 (39.18 to 52.88) | 21.36% (2.06% to 40.39%) | 96.44 (81.12 to 112.34) | 1.69 (1.42 to 1.96) | 34.28% (11.60% to 57.84%) | 0.864 |
| **Kansas** | 368.02 (330.06 to 410.28) | 9.31 (8.36 to 10.32) | 17.47% (7.12% to 30.12%) | 2180.87 (1826.32 to 2547.58) | 49.90 (42.10 to 58.10) | 18.87% (-0.47% to 38.87%) | 88.95 (74.50 to 104.12) | 1.82 (1.53 to 2.13) | 33.51% (10.31% to 57.69%) | 0.859 |
| **Kentucky** | 519.10 (481.09 to 562.63) | 8.31 (7.69 to 9.01) | 20.78% (8.80% to 34.76%) | 3181.23 (2673.61 to 3705.44) | 45.81 (38.75 to 53.07) | 23.13% (4.48% to 44.24%) | 124.43 (104.41 to 146.43) | 1.61 (1.35 to 1.90) | 33.38% (11.97% to 55.82%) | 0.821 |
| **Louisiana** | 590.87 (548.15 to 635.08) | 9.66 (9.00 to 10.39) | 35.74% (21.55% to 54.50%) | 3087.09 (2663.16 to 3590.62) | 45.75 (39.86 to 53.01) | 15.76% (-0.50% to 34.31%) | 117.67 (100.79 to 137.82) | 1.57 (1.35 to 1.84) | 29.56% (9.80% to 51.75%) | 0.825 |
| **Maine** | 199.14 (177.72 to 220.87) | 9.17 (8.21 to 10.17) | 8.78% (-1.84% to 20.14%) | 1306.36 (1119.83 to 1515.61) | 52.17 (44.85 to 60.04) | 19.69% (0.99% to 38.36%) | 54.97 (46.89 to 64.24) | 1.90 (1.62 to 2.21) | 26.63% (5.74% to 47.57%) | 0.866 |
| **Maryland** | 719.61 (656.95 to 790.19) | 8.58 (7.77 to 9.47) | 10.52% (0.21% to 22.37%) | 3863.25 (3252.55 to 4513.68) | 41.00 (34.93 to 47.59) | 3.63% (-13.15% to 19.74%) | 152.81 (126.81 to 180.42) | 1.47 (1.22 to 1.72) | 14.65% (-4.61% to 33.58%) | 0.889 |
| **Massachusetts** | 1051.96 (974.54 to 1143.16) | 10.40 (9.66 to 11.25) | 11.54% (2.67% to 23.17%) | 4664.18 (3834.31 to 5479.88) | 40.18 (33.19 to 47.19) | -5.15% (-21.41% to 11.36%) | 196.93 (160.56 to 231.25) | 1.53 (1.26 to 1.81) | 8.22% (-10.67% to 28.06%) | 0.907 |
| **Michigan** | 1369.58 (1273.57 to 1479.88) | 9.35 (8.75 to 10.11) | 17.79% (8.23% to 29.62%) | 7344.97 (6289.71 to 8457.05) | 44.94 (38.78 to 51.57) | 12.19% (-3.50% to 28.68%) | 295.34 (250.62 to 340.86) | 1.59 (1.36 to 1.84) | 24.80% (7.09% to 44.58%) | 0.864 |
| **Minnesota** | 931.87 (840.87 to 1023.44) | 11.54 (10.52 to 12.62) | 16.68% (6.09% to 30.67%) | 4784.93 (4020.08 to 5645.00) | 53.05 (44.77 to 61.98) | 10.53% (-6.57% to 28.96%) | 200.53 (164.69 to 240.07) | 2.00 (1.65 to 2.39) | 21.88% (2.02% to 44.00%) | 0.887 |
| **Mississippi** | 262.61 (232.60 to 292.73) | 6.66 (5.92 to 7.40) | 17.27% (7.16% to 28.37%) | 1855.54 (1583.34 to 2165.55) | 43.12 (37.18 to 50.05) | 21.90% (4.77% to 42.17%) | 71.47 (60.85 to 83.62) | 1.48 (1.26 to 1.72) | 36.33% (15.87% to 59.15%) | 0.810 |
| **Missouri** | 716.81 (666.00 to 773.68) | 8.37 (7.79 to 9.03) | 13.61% (1.00% to 27.69%) | 4616.64 (3924.87 to 5390.10) | 48.24 (41.50 to 55.86) | 20.53% (2.33% to 40.06%) | 186.78 (158.39 to 219.66) | 1.73 (1.47 to 2.02) | 33.36% (12.17% to 56.57%) | 0.848 |
| **Montana** | 144.01 (128.97 to 162.19) | 9.09 (8.17 to 10.09) | 15.07% (4.61% to 27.33%) | 916.60 (785.20 to 1073.98) | 50.98 (44.09 to 59.18) | 15.55% (0.02% to 36.23%) | 37.78 (32.18 to 44.24) | 1.80 (1.54 to 2.11) | 24.68% (7.69% to 47.86%) | 0.859 |
| **Nebraska** | 243.62 (211.26 to 280.89) | 9.08 (7.87 to 10.39) | 17.08% (8.26% to 26.79%) | 1385.99 (1180.25 to 1606.26) | 47.42 (40.61 to 54.38) | 14.53% (-2.58% to 31.86%) | 56.72 (48.04 to 65.89) | 1.73 (1.47 to 2.01) | 25.90% (5.53% to 45.66%) | 0.865 |
| **Nevada** | 279.14 (250.10 to 311.37) | 6.86 (6.14 to 7.72) | 4.81% (-4.42% to 14.95%) | 1559.46 (1326.91 to 1798.02) | 33.82 (29.06 to 38.77) | -0.21% (-14.31% to 14.87%) | 61.73 (52.16 to 71.82) | 1.22 (1.03 to 1.41) | 13.35% (-3.59% to 32.08%) | 0.848 |
| **New Hampshire** | 218.73 (197.69 to 241.15) | 10.35 (9.41 to 11.41) | 14.47% (4.80% to 26.00%) | 1032.05 (866.64 to 1203.14) | 42.23 (35.71 to 48.82) | -1.59% (-16.06% to 15.53%) | 42.85 (35.60 to 50.28) | 1.56 (1.30 to 1.82) | 4.77% (-11.98% to 24.71%) | 0.898 |
| **New Jersey** | 1213.39 (1130.38 to 1307.44) | 9.35 (8.72 to 10.11) | 11.84% (-0.02% to 23.90%) | 4902.38 (4174.13 to 5767.31) | 33.11 (28.30 to 38.63) | -12.66% (-25.53% to 2.41%) | 199.11 (168.02 to 234.09) | 1.22 (1.03 to 1.43) | -0.22% (-15.68% to 18.61%) | 0.892 |
| **New Mexico** | 212.25 (192.32 to 234.20) | 7.08 (6.45 to 7.84) | 8.94% (-1.05% to 20.39%) | 1362.17 (1161.25 to 1588.64) | 42.35 (36.21 to 48.86) | 25.24% (7.68% to 45.79%) | 53.93 (46.22 to 63.47) | 1.45 (1.24 to 1.71) | 38.99% (18.05% to 63.93%) | 0.832 |
| **New York** | 2790.73 (2604.30 to 2993.38) | 9.90 (9.28 to 10.60) | 21.12% (8.49% to 35.08%) | 10477.60 (8975.21 to 12027.09) | 32.99 (28.43 to 37.69) | -8.58% (-20.86% to 4.59%) | 418.34 (350.68 to 485.01) | 1.19 (1.00 to 1.38) | 10.18% (-6.14% to 26.83%) | 0.885 |
| **North Carolina** | 1254.69 (1170.09 to 1351.20) | 8.57 (8.00 to 9.20) | 13.94% (1.42% to 28.63%) | 7205.48 (6177.51 to 8288.28) | 44.66 (38.51 to 51.15) | 10.09% (-4.60% to 27.68%) | 290.66 (245.35 to 335.00) | 1.60 (1.36 to 1.85) | 24.11% (5.90% to 45.70%) | 0.844 |
| **North Dakota** | 120.77 (103.13 to 139.21) | 11.09 (9.58 to 12.69) | 32.59% (23.80% to 42.11%) | 509.27 (444.69 to 580.66) | 45.20 (39.79 to 51.10) | 7.20% (-6.36% to 22.48%) | 20.12 (17.22 to 23.18) | 1.54 (1.32 to 1.78) | 12.85% (-2.27% to 29.56%) | 0.873 |
| **Ohio** | 1484.74 (1376.30 to 1605.85) | 8.87 (8.32 to 9.53) | 12.99% (2.08% to 25.78%) | 8754.95 (7515.71 to 10119.01) | 46.72 (40.57 to 53.66) | 13.96% (0.52% to 30.68%) | 352.01 (301.09 to 406.70) | 1.66 (1.42 to 1.92) | 25.65% (9.31% to 44.79%) | 0.851 |
| **Oklahoma** | 412.00 (378.75 to 448.46) | 7.88 (7.20 to 8.65) | 15.90% (4.76% to 28.62%) | 2791.26 (2427.24 to 3207.29) | 48.40 (42.04 to 55.45) | 29.99% (13.12% to 50.24%) | 110.75 (96.02 to 128.32) | 1.72 (1.49 to 1.99) | 46.15% (27.06% to 70.34%) | 0.829 |
| **Oregon** | 603.50 (539.02 to 672.42) | 9.44 (8.66 to 10.39) | 4.34% (-6.15% to 16.58%) | 3654.14 (3134.65 to 4312.72) | 51.15 (44.29 to 59.66) | 8.21% (-7.01% to 27.22%) | 153.47 (129.91 to 182.08) | 1.90 (1.62 to 2.25) | 21.82% (4.55% to 44.85%) | 0.870 |
| **Pennsylvania** | 1861.33 (1732.07 to 2009.52) | 9.86 (9.21 to 10.64) | 22.41% (9.06% to 37.88%) | 9457.78 (8007.41 to 10923.79) | 44.14 (37.80 to 50.92) | 9.47% (-5.94% to 25.60%) | 383.96 (323.45 to 444.35) | 1.58 (1.34 to 1.82) | 20.25% (2.74% to 39.14%) | 0.872 |
| **Rhode Island** | 137.07 (116.68 to 157.57) | 8.77 (7.49 to 10.11) | 17.37% (8.40% to 26.87%) | 655.82 (554.16 to 765.05) | 36.32 (31.22 to 42.24) | -9.32% (-22.11% to 5.15%) | 27.26 (22.82 to 32.02) | 1.34 (1.13 to 1.58) | 0.94% (-12.93% to 18.28%) | 0.884 |
| **South Carolina** | 606.54 (559.62 to 660.75) | 8.15 (7.59 to 8.78) | 13.53% (2.43% to 27.25%) | 3528.25 (3007.32 to 4048.40) | 43.55 (37.54 to 49.63) | 9.27% (-7.42% to 27.09%) | 140.13 (118.06 to 161.20) | 1.52 (1.29 to 1.75) | 24.31% (4.72% to 44.02%) | 0.838 |
| **South Dakota** | 125.45 (108.08 to 144.35) | 10.22 (9.01 to 11.43) | 33.72% (25.79% to 43.27%) | 639.54 (562.27 to 730.48) | 48.66 (42.85 to 55.00) | 14.99% (0.49% to 30.56%) | 25.74 (22.40 to 29.78) | 1.68 (1.47 to 1.94) | 25.23% (8.50% to 44.03%) | 0.856 |
| **Tennessee** | 776.26 (714.02 to 845.48) | 8.20 (7.62 to 8.89) | 20.22% (8.76% to 33.08%) | 5030.79 (4285.09 to 5769.89) | 47.31 (40.55 to 54.18) | 21.70% (4.19% to 39.31%) | 199.04 (169.30 to 228.60) | 1.67 (1.42 to 1.92) | 34.18% (14.21% to 54.98%) | 0.833 |
| **Texas** | 3245.26 (3032.91 to 3477.65) | 8.79 (8.20 to 9.43) | 14.78% (3.64% to 27.57%) | 14303.74 (12468.59 to 16306.60) | 36.01 (31.66 to 40.83) | -3.95% (-15.78% to 9.37%) | 554.06 (479.29 to 636.89) | 1.30 (1.13 to 1.49) | 10.34% (-4.32% to 27.63%) | 0.835 |
| **Utah** | 366.64 (328.19 to 408.95) | 9.70 (8.77 to 10.77) | 10.02% (-0.28% to 21.72%) | 1799.26 (1542.22 to 2080.15) | 46.14 (39.86 to 53.13) | 4.83% (-8.96% to 21.66%) | 71.35 (60.27 to 83.33) | 1.73 (1.46 to 2.02) | 21.14% (3.52% to 41.83%) | 0.855 |
| **Vermont** | 149.85 (129.87 to 170.74) | 14.69 (13.07 to 16.52) | 33.73% (24.36% to 42.96%) | 568.14 (499.20 to 648.90) | 50.04 (44.16 to 56.74) | 0.02% (-12.48% to 14.30%) | 23.40 (19.97 to 26.81) | 1.78 (1.54 to 2.04) | 4.62% (-9.75% to 20.77%) | 0.890 |
| **Virginia** | 894.17 (826.23 to 975.33) | 7.63 (7.03 to 8.32) | -2.32% (-11.52% to 9.32%) | 5661.34 (4805.92 to 6575.85) | 42.87 (36.88 to 49.43) | 1.95% (-13.28% to 18.75%) | 230.29 (193.52 to 269.76) | 1.58 (1.33 to 1.84) | 14.49% (-4.18% to 35.36%) | 0.880 |
| **Washington** | 987.69 (916.49 to 1075.09) | 9.26 (8.54 to 10.11) | -4.22% (-13.76% to 7.58%) | 5818.47 (4901.46 to 6688.01) | 47.84 (40.68 to 54.75) | -1.02% (-16.33% to 13.15%) | 236.93 (200.10 to 274.80) | 1.76 (1.49 to 2.04) | 9.00% (-8.25% to 25.81%) | 0.878 |
| **West Virginia** | 237.08 (212.18 to 263.35) | 8.93 (8.15 to 9.76) | 32.52% (21.46% to 46.21%) | 1510.99 (1289.23 to 1743.99) | 50.93 (43.81 to 58.56) | 42.50% (21.34% to 64.94%) | 60.04 (50.78 to 69.78) | 1.74 (1.48 to 2.02) | 51.87% (29.21% to 77.93%) | 0.818 |
| **Wisconsin** | 775.25 (714.40 to 843.43) | 9.17 (8.48 to 10.00) | 6.59% (-4.27% to 19.52%) | 4551.68 (3836.46 to 5339.23) | 48.11 (40.95 to 55.91) | 12.54% (-4.19% to 31.17%) | 191.23 (158.94 to 225.11) | 1.79 (1.49 to 2.10) | 23.49% (3.90% to 44.62%) | 0.872 |
| **Wyoming** | 77.44 (65.84 to 89.26) | 9.54 (8.21 to 10.97) | 21.52% (12.89% to 31.32%) | 435.33 (387.09 to 491.85) | 49.41 (44.06 to 55.55) | 15.73% (1.95% to 30.72%) | 17.60 (15.42 to 20.09) | 1.77 (1.56 to 2.02) | 27.56% (11.95% to 45.74%) | 0.862 |
| **Statistical Test for SDI** | Spearman's r : 𝜌 = 0.064 p-value = 0.652 | Spearman's r : 𝜌 = 0.466 p-value < 0.001 | Spearman’s r : 𝜌 = -0.150  p-value = 0.287 | Spearman's r : 𝜌 = -0.007 p-value = 0.960 | Spearman's r : 𝜌 = -0.291 p-value = 0.037 | Spearman's r : 𝜌 = -0.716  p-value < 0.001 | Spearman's r : 𝜌 = 0.018 p-value = 0.901 | Spearman’s r : 𝜌 = -0.206  p-value = 0.144 | Spearman’s r : 𝜌 = -0.780,  p-value < 0.001 |  |

Abbreviations: DALYs, disability-adjusted life years; SDI, sociodemographic index; UI, uncertainty interval

## Table S7b: Numbers and age-standardized rates of incidence, YLDs, and YLLs in 2021, and percentage changes from 1990 to 2021, with SDI

|  | **Incidence (95% UI)** | | | **YLDs (95% UI)** | | | **YLLs (95% UI)** | | |  |
| --- | --- | --- | --- | --- | --- | --- | --- | --- | --- | --- |
|  | **Absolute number, thousands, 2021** | **Age-standardized rate,**  **per 100,000 people, 2021** | **Percentage Change, 1990-2021** | **Absolute number, thousands, 2021** | **Age-standardized rate,**  **per 100,000 people, 2021** | **Percentage Change, 1990-2021** | **Absolute number, thousands, 2021** | **Age-standardized rate,**  **per 100,000 people, 2021** | **Percentage Change, 1990-2021** | **SDI** |
| **United States of America** | 11089.01 (10633.99 to 11559.06) | 2.06 (1.98 to 2.14) | 15.03% (11.49% to 19.04%) | 8597.05 (6259.71 to 11269.09) | 1.87 (1.36 to 2.46) | 12.80% (2.98% to 23.54%) | 201962.40 (191037.84 to 210306.37) | 39.48 (37.75 to 40.98) | 3.76% (-0.25% to 7.38%) | 0.862 |
| **Alabama** | 163.02 (155.86 to 170.38) | 2.01 (1.92 to 2.09) | 28.17% (22.09% to 34.02%) | 112.00 (80.97 to 149.71) | 1.64 (1.20 to 2.18) | 20.81% (8.47% to 34.14%) | 3550.79 (3015.44 to 4123.96) | 46.21 (39.68 to 53.35) | 28.83% (10.39% to 49.60%) | 0.826 |
| **Alaska** | 22.30 (21.05 to 23.78) | 2.20 (2.09 to 2.33) | 14.36% (10.04% to 18.36%) | 19.37 (13.85 to 26.50) | 2.16 (1.55 to 2.92) | 31.42% (19.32% to 43.94%) | 379.28 (330.47 to 437.66) | 38.97 (34.18 to 44.76) | 0.45% (-12.68% to 15.91%) | 0.858 |
| **Arizona** | 241.45 (231.24 to 252.35) | 2.07 (1.99 to 2.16) | 15.65% (10.54% to 20.30%) | 185.79 (133.54 to 244.00) | 1.88 (1.36 to 2.47) | 20.83% (7.73% to 36.11%) | 4595.81 (3936.96 to 5318.27) | 42.23 (36.72 to 48.70) | 11.01% (-4.77% to 28.80%) | 0.847 |
| **Arkansas** | 106.95 (101.67 to 112.20) | 2.24 (2.13 to 2.35) | 29.50% (24.27% to 35.04%) | 81.49 (58.28 to 107.59) | 1.99 (1.43 to 2.62) | 31.55% (17.89% to 46.48%) | 2101.51 (1791.36 to 2437.11) | 46.72 (39.95 to 53.99) | 22.40% (3.53% to 42.94%) | 0.818 |
| **California** | 1057.66 (1013.15 to 1104.78) | 1.75 (1.67 to 1.83) | -0.88% (-4.74% to 3.07%) | 913.41 (660.61 to 1203.65) | 1.73 (1.25 to 2.26) | 3.94% (-7.34% to 16.61%) | 18903.80 (16268.38 to 21631.36) | 32.53 (28.27 to 37.09) | -13.85% (-24.95% to  -2.35%) | 0.870 |
| **Colorado** | 228.60 (217.86 to 239.04) | 2.49 (2.38 to 2.59) | 21.36% (16.14% to 26.07%) | 173.07 (124.38 to 227.84) | 2.19 (1.58 to 2.85) | 14.36% (2.53% to 27.52%) | 3889.08 (3241.45 to 4563.76) | 44.38 (37.35 to 51.59) | 0.33% (-15.39% to 16.59%) | 0.877 |
| **Connecticut** | 136.94 (130.35 to 143.70) | 2.17 (2.06 to 2.27) | 14.11% (9.64% to 19.17%) | 119.11 (85.48 to 156.65) | 2.24 (1.63 to 2.95) | 15.13% (4.04% to 27.55%) | 2348.51 (1939.31 to 2769.84) | 39.18 (32.35 to 45.83) | -0.87% (-18.29% to 17.55%) | 0.903 |
| **Delaware** | 37.04 (35.20 to 39.07) | 2.20 (2.10 to 2.31) | 17.44% (13.13% to 21.75%) | 32.77 (23.93 to 43.18) | 2.30 (1.68 to 3.05) | 30.88% (19.43% to 44.45%) | 662.14 (582.83 to 757.60) | 41.16 (36.63 to 46.33) | 9.26% (-4.73% to 24.46%) | 0.866 |
| **District of Columbia** | 14.25 (13.35 to 15.22) | 1.66 (1.56 to 1.76) | 4.43% (1.14% to 7.71%) | 13.76 (9.82 to 18.51) | 1.69 (1.22 to 2.27) | 28.28% (18.06% to 40.21%) | 247.76 (212.94 to 288.25) | 28.96 (25.10 to 33.55) | -49.54% (-57.79% to  -40.24%) | 0.906 |
| **Florida** | 722.35 (690.79 to 754.46) | 1.83 (1.75 to 1.92) | 9.26% (4.90% to 14.08%) | 574.26 (412.92 to 751.09) | 1.72 (1.25 to 2.25) | 17.50% (4.77% to 32.71%) | 13289.99 (11134.86 to 15556.94) | 36.41 (30.89 to 42.16) | -0.01% (-14.35% to 16.16%) | 0.862 |
| **Georgia** | 305.23 (292.44 to 320.70) | 1.88 (1.81 to 1.97) | 15.67% (11.31% to 20.98%) | 220.41 (160.01 to 290.27) | 1.58 (1.15 to 2.07) | 8.10% (-1.02% to 20.03%) | 5861.41 (5044.31 to 6797.53) | 37.49 (32.48 to 43.07) | 6.01% (-8.65% to 22.66%) | 0.847 |
| **Hawaii** | 42.38 (39.86 to 45.18) | 1.77 (1.67 to 1.88) | 18.32% (13.63% to 23.36%) | 43.39 (30.81 to 59.25) | 2.18 (1.56 to 2.94) | 30.35% (20.91% to 41.65%) | 704.57 (590.34 to 826.14) | 30.78 (26.09 to 35.54) | 2.29% (-14.45% to 20.81%) | 0.870 |
| **Idaho** | 72.15 (68.62 to 75.61) | 2.47 (2.36 to 2.59) | 17.99% (13.50% to 22.81%) | 47.39 (34.15 to 62.36) | 1.89 (1.36 to 2.48) | 4.37% (-6.32% to 17.12%) | 1317.05 (1102.11 to 1533.59) | 47.57 (40.02 to 55.12) | 9.12% (-7.05% to 27.03%) | 0.837 |
| **Illinois** | 405.79 (388.69 to 423.47) | 1.98 (1.90 to 2.07) | 11.84% (7.41% to 16.58%) | 314.41 (226.05 to 410.13) | 1.82 (1.32 to 2.37) | 6.54% (-4.04% to 19.73%) | 7195.65 (6176.72 to 8382.69) | 37.00 (31.88 to 42.74) | -2.16% (-15.64% to 13.12%) | 0.879 |
| **Indiana** | 245.24 (234.07 to 256.28) | 2.26 (2.16 to 2.36) | 23.96% (18.94% to 29.54%) | 164.95 (120.43 to 216.58) | 1.79 (1.29 to 2.36) | 11.68% (0.52% to 24.47%) | 4796.35 (4094.40 to 5506.22) | 46.64 (40.17 to 53.38) | 21.11% (4.58% to 40.03%) | 0.843 |
| **Iowa** | 124.05 (118.09 to 130.30) | 2.27 (2.17 to 2.39) | 24.10% (19.59% to 29.04%) | 73.77 (52.75 to 98.59) | 1.64 (1.17 to 2.22) | 3.23% (-5.69% to 12.91%) | 2228.86 (1876.07 to 2591.74) | 44.37 (37.59 to 51.32) | 22.15% (2.20% to 41.99%) | 0.864 |
| **Kansas** | 110.06 (104.64 to 115.66) | 2.39 (2.27 to 2.51) | 28.99% (24.20% to 34.39%) | 78.08 (56.99 to 103.48) | 1.98 (1.43 to 2.60) | 17.36% (7.00% to 29.83%) | 2102.79 (1750.12 to 2474.43) | 47.92 (40.11 to 56.16) | 18.93% (-1.18% to 39.83%) | 0.859 |
| **Kentucky** | 160.06 (152.58 to 167.02) | 2.17 (2.07 to 2.26) | 29.48% (23.59% to 35.53%) | 110.13 (79.69 to 145.02) | 1.76 (1.28 to 2.33) | 20.66% (8.63% to 34.72%) | 3071.11 (2563.14 to 3619.54) | 44.05 (37.02 to 51.50) | 23.23% (3.79% to 45.17%) | 0.821 |
| **Louisiana** | 147.89 (140.59 to 155.19) | 2.09 (2.00 to 2.19) | 22.48% (16.57% to 27.77%) | 125.39 (91.17 to 163.75) | 2.05 (1.49 to 2.69) | 35.61% (21.44% to 54.37%) | 2961.70 (2532.90 to 3466.77) | 43.70 (37.79 to 50.87) | 14.97% (-1.89% to 34.12%) | 0.825 |
| **Maine** | 72.76 (69.34 to 76.69) | 2.61 (2.49 to 2.73) | 23.95% (18.94% to 28.51%) | 42.30 (30.37 to 56.40) | 1.95 (1.40 to 2.60) | 8.75% (-1.84% to 20.14%) | 1264.06 (1080.39 to 1474.76) | 50.22 (42.97 to 57.84) | 20.16% (0.67% to 39.68%) | 0.866 |
| **Maryland** | 210.65 (200.64 to 221.02) | 2.13 (2.04 to 2.23) | 17.34% (12.80% to 21.89%) | 152.90 (109.86 to 202.34) | 1.82 (1.33 to 2.42) | 10.52% (0.21% to 22.37%) | 3710.35 (3101.42 to 4358.16) | 39.18 (32.99 to 45.81) | 3.33% (-14.11% to 20.06%) | 0.889 |
| **Massachusetts** | 281.00 (268.58 to 293.81) | 2.33 (2.22 to 2.43) | 16.46% (12.26% to 21.02%) | 223.20 (162.08 to 291.93) | 2.21 (1.60 to 2.89) | 11.40% (2.33% to 23.37%) | 4440.98 (3631.56 to 5257.87) | 37.98 (31.23 to 45.03) | -5.96% (-22.81% to 11.22%) | 0.907 |
| **Michigan** | 400.72 (383.58 to 417.74) | 2.27 (2.18 to 2.36) | 19.61% (15.16% to 24.63%) | 290.75 (213.41 to 382.45) | 1.99 (1.45 to 2.60) | 17.73% (8.37% to 29.86%) | 7054.22 (6001.43 to 8140.11) | 42.96 (36.85 to 49.48) | 11.94% (-4.37% to 29.30%) | 0.864 |
| **Minnesota** | 266.13 (254.35 to 279.18) | 2.79 (2.67 to 2.91) | 25.31% (20.88% to 30.05%) | 196.03 (142.79 to 256.07) | 2.44 (1.77 to 3.19) | 15.86% (5.46% to 30.61%) | 4588.90 (3826.85 to 5450.82) | 50.61 (42.48 to 59.51) | 10.29% (-7.69% to 29.60%) | 0.887 |
| **Mississippi** | 85.62 (81.28 to 89.99) | 1.85 (1.76 to 1.94) | 23.55% (18.64% to 29.37%) | 55.79 (40.23 to 74.30) | 1.41 (1.01 to 1.87) | 17.25% (7.16% to 28.37%) | 1799.75 (1535.28 to 2109.83) | 41.71 (35.98 to 48.63) | 22.07% (4.42% to 43.09%) | 0.810 |
| **Missouri** | 224.51 (215.14 to 234.57) | 2.18 (2.09 to 2.27) | 24.57% (18.71% to 29.99%) | 152.32 (111.11 to 199.14) | 1.78 (1.29 to 2.33) | 13.65% (1.00% to 27.69%) | 4464.33 (3773.41 to 5244.59) | 46.46 (39.60 to 54.18) | 20.81% (1.83% to 41.34%) | 0.848 |
| **Montana** | 50.37 (47.65 to 53.06) | 2.53 (2.41 to 2.65) | 24.68% (20.00% to 28.97%) | 30.58 (21.86 to 40.69) | 1.93 (1.39 to 2.54) | 15.01% (4.61% to 27.33%) | 886.02 (751.67 to 1043.37) | 49.05 (41.92 to 57.06) | 15.57% (-0.50% to 37.15%) | 0.859 |
| **Nebraska** | 71.43 (67.87 to 75.39) | 2.29 (2.18 to 2.43) | 26.98% (22.73% to 31.76%) | 51.72 (37.04 to 69.04) | 1.93 (1.37 to 2.58) | 17.00% (8.33% to 26.64%) | 1334.26 (1127.12 to 1552.46) | 45.49 (38.63 to 52.44) | 14.43% (-3.39% to 32.43%) | 0.865 |
| **Nevada** | 74.87 (70.82 to 79.01) | 1.58 (1.50 to 1.67) | 3.06% (-1.05% to 6.93%) | 59.30 (42.27 to 79.20) | 1.46 (1.06 to 1.96) | 4.84% (-4.42% to 14.95%) | 1500.15 (1268.58 to 1743.04) | 32.36 (27.62 to 37.30) | -0.43% (-15.02% to 15.17%) | 0.848 |
| **New Hampshire** | 61.09 (57.90 to 64.25) | 2.35 (2.25 to 2.47) | 17.92% (13.72% to 22.69%) | 46.39 (32.99 to 61.24) | 2.20 (1.57 to 2.90) | 14.35% (4.71% to 25.83%) | 985.65 (822.36 to 1159.14) | 40.04 (33.55 to 46.64) | -2.34% (-17.30% to 15.68%) | 0.898 |
| **New Jersey** | 283.35 (269.82 to 296.32) | 1.85 (1.76 to 1.93) | 6.01% (2.32% to 10.23%) | 257.85 (185.94 to 336.29) | 1.99 (1.45 to 2.60) | 11.83% (-0.02% to 23.90%) | 4644.54 (3930.04 to 5513.66) | 31.12 (26.39 to 36.68) | -13.87% (-27.26% to 1.84%) | 0.892 |
| **New Mexico** | 65.46 (62.20 to 69.14) | 1.85 (1.76 to 1.95) | 19.34% (14.74% to 24.41%) | 45.09 (32.28 to 59.01) | 1.51 (1.08 to 1.99) | 8.92% (-1.05% to 20.39%) | 1317.08 (1119.55 to 1548.48) | 40.85 (34.78 to 47.47) | 25.94% (7.70% to 47.56%) | 0.832 |
| **New York** | 542.39 (517.74 to 566.89) | 1.67 (1.59 to 1.74) | 4.39% (0.14% to 9.09%) | 592.61 (430.72 to 783.14) | 2.10 (1.53 to 2.77) | 21.04% (8.26% to 34.95%) | 9884.99 (8382.74 to 11486.40) | 30.88 (26.40 to 35.65) | -10.07% (-23.21% to 3.61%) | 0.885 |
| **North Carolina** | 363.50 (348.01 to 378.64) | 2.11 (2.03 to 2.20) | 18.42% (13.61% to 23.75%) | 266.62 (191.76 to 349.38) | 1.82 (1.32 to 2.40) | 13.91% (1.42% to 28.63%) | 6938.86 (5914.60 to 8035.18) | 42.84 (36.76 to 49.43) | 9.93% (-5.44% to 28.07%) | 0.844 |
| **North Dakota** | 32.79 (31.01 to 34.65) | 2.66 (2.52 to 2.80) | 36.54% (31.88% to 41.71%) | 25.63 (18.12 to 35.06) | 2.36 (1.67 to 3.19) | 32.55% (23.87% to 41.93%) | 483.64 (417.81 to 554.86) | 42.85 (37.44 to 48.72) | 6.09% (-7.90% to 22.04%) | 0.873 |
| **Ohio** | 465.33 (446.39 to 483.69) | 2.30 (2.21 to 2.40) | 23.09% (18.48% to 28.40%) | 315.45 (229.15 to 414.17) | 1.89 (1.38 to 2.50) | 12.97% (2.08% to 25.78%) | 8439.50 (7231.51 to 9818.22) | 44.84 (38.69 to 51.85) | 14.01% (0.05% to 31.29%) | 0.851 |
| **Oklahoma** | 133.34 (127.49 to 139.54) | 2.18 (2.09 to 2.28) | 28.74% (23.00% to 34.15%) | 87.54 (62.50 to 116.10) | 1.67 (1.21 to 2.21) | 15.89% (4.76% to 28.62%) | 2703.72 (2340.20 to 3134.26) | 46.73 (40.57 to 54.17) | 30.56% (12.99% to 51.74%) | 0.829 |
| **Oregon** | 199.70 (190.07 to 209.64) | 2.59 (2.48 to 2.71) | 19.26% (14.97% to 23.51%) | 128.11 (92.24 to 170.28) | 2.00 (1.43 to 2.64) | 4.28% (-6.19% to 16.41%) | 3526.03 (3007.19 to 4178.33) | 49.14 (42.33 to 57.55) | 8.38% (-7.48% to 28.47%) | 0.870 |
| **Pennsylvania** | 498.86 (477.38 to 520.88) | 2.19 (2.09 to 2.29) | 21.77% (16.37% to 27.91%) | 395.00 (289.64 to 520.89) | 2.09 (1.53 to 2.78) | 22.29% (8.85% to 37.85%) | 9062.78 (7654.32 to 10476.13) | 42.05 (35.80 to 48.45) | 8.90% (-6.87% to 25.68%) | 0.872 |
| **Rhode Island** | 40.14 (37.94 to 42.72) | 2.12 (2.00 to 2.24) | 21.21% (16.62% to 26.18%) | 29.11 (20.60 to 38.78) | 1.86 (1.32 to 2.53) | 17.34% (8.40% to 26.87%) | 626.72 (529.92 to 736.64) | 34.46 (29.60 to 40.35) | -10.42% (-23.47% to 4.59%) | 0.884 |
| **South Carolina** | 180.07 (171.31 to 188.75) | 2.05 (1.96 to 2.14) | 16.65% (12.09% to 21.81%) | 128.85 (93.74 to 169.34) | 1.73 (1.25 to 2.27) | 13.53% (2.43% to 27.25%) | 3399.40 (2874.14 to 3914.63) | 41.82 (35.79 to 47.87) | 9.10% (-8.12% to 28.06%) | 0.838 |
| **South Dakota** | 35.80 (33.83 to 37.93) | 2.49 (2.36 to 2.61) | 32.92% (28.55% to 38.42%) | 26.61 (18.81 to 35.77) | 2.17 (1.55 to 2.92) | 33.59% (25.86% to 43.38%) | 612.93 (536.10 to 703.13) | 46.49 (40.80 to 52.94) | 14.25% (-0.39% to 30.33%) | 0.856 |
| **Tennessee** | 250.51 (239.40 to 261.01) | 2.20 (2.11 to 2.30) | 29.28% (23.87% to 35.12%) | 164.91 (119.90 to 216.57) | 1.74 (1.26 to 2.28) | 20.19% (8.76% to 33.08%) | 4865.88 (4135.68 to 5615.15) | 45.57 (38.88 to 52.40) | 21.75% (3.81% to 39.89%) | 0.833 |
| **Texas** | 762.57 (729.84 to 795.83) | 1.87 (1.79 to 1.95) | 9.30% (5.05% to 13.73%) | 689.23 (502.17 to 897.62) | 1.87 (1.35 to 2.44) | 14.73% (3.54% to 27.46%) | 13614.51 (11774.77 to 15574.73) | 34.15 (29.74 to 38.84) | -4.80% (-17.26% to 9.61%) | 0.835 |
| **Utah** | 100.96 (95.87 to 106.39) | 2.53 (2.40 to 2.66) | 19.18% (15.28% to 23.50%) | 77.77 (56.12 to 103.16) | 2.06 (1.51 to 2.72) | 9.83% (-0.53% to 21.52%) | 1721.49 (1460.78 to 1995.05) | 44.08 (37.68 to 50.87) | 4.60% (-9.82% to 22.45%) | 0.855 |
| **Vermont** | 35.91 (33.89 to 38.11) | 2.92 (2.77 to 3.08) | 21.48% (17.61% to 24.88%) | 31.36 (22.70 to 41.79) | 3.09 (2.23 to 4.11) | 32.52% (22.39% to 42.92%) | 536.78 (465.62 to 613.88) | 46.94 (40.93 to 53.50) | -1.57% (-14.46% to 13.55%) | 0.890 |
| **Virginia** | 288.45 (275.50 to 301.78) | 2.07 (1.98 to 2.17) | 10.39% (5.95% to 15.01%) | 190.00 (136.55 to 250.82) | 1.62 (1.16 to 2.14) | -2.32% (-11.52% to 9.32%) | 5471.34 (4614.30 to 6408.75) | 41.25 (35.29 to 48.00) | 2.12% (-13.66% to 19.60%) | 0.880 |
| **Washington** | 320.24 (305.48 to 334.46) | 2.48 (2.38 to 2.59) | 10.51% (6.36% to 14.72%) | 209.67 (150.62 to 278.34) | 1.97 (1.42 to 2.59) | -4.30% (-13.80% to 7.40%) | 5608.80 (4694.36 to 6486.49) | 45.88 (38.69 to 52.76) | -0.88% (-16.68% to 13.89%) | 0.878 |
| **West Virginia** | 72.45 (68.74 to 76.31) | 2.22 (2.11 to 2.33) | 35.31% (29.80% to 41.55%) | 50.35 (36.01 to 66.39) | 1.90 (1.37 to 2.52) | 32.49% (21.46% to 46.21%) | 1460.64 (1239.61 to 1696.54) | 49.03 (41.90 to 56.88) | 42.92% (20.96% to 66.16%) | 0.818 |
| **Wisconsin** | 252.12 (241.39 to 263.03) | 2.46 (2.36 to 2.57) | 22.82% (18.03% to 28.05%) | 164.64 (120.65 to 217.54) | 1.95 (1.42 to 2.57) | 6.54% (-4.36% to 19.42%) | 4387.04 (3678.70 to 5180.84) | 46.16 (39.08 to 54.20) | 12.81% (-4.81% to 31.92%) | 0.872 |
| **Wyoming** | 22.52 (21.26 to 23.89) | 2.38 (2.26 to 2.51) | 22.04% (18.37% to 26.32%) | 16.44 (11.86 to 22.06) | 2.03 (1.45 to 2.73) | 21.45% (12.86% to 31.32%) | 418.90 (370.53 to 475.63) | 47.38 (42.15 to 53.52) | 15.50% (1.16% to 31.22%) | 0.862 |
| **Statistical Test for SDI** | Spearman’s r: 𝜌 = 0.045 p-value = 0.751 | Spearman’s r: 𝜌 = 0.154  p-value = 0.276 | Spearman’s r: 𝜌 = -0.419  p-value = 0.002 | Spearman’s r: 𝜌 = 0.064 p-value = 0.652 | Spearman's r: 𝜌 = 0.468 p-value < 0.001 | Spearman’s r: 𝜌 = -0.419  p-value = 0.002 | Spearman's r: 𝜌 = -0.011 p-value = 0.939 | Spearman's r: 𝜌 = -0.308 p-value = 0.027 | Spearman's r: 𝜌 = -0.721  p-value < 0.001 |  |

Abbreviations: SDI, sociodemographic index; UI, uncertainty interval; YLDs, years lived with disability; YLLs, years of life lost
